# Supplementary figures and images for: A novel long non-coding RNA, AC012456.4, as a valuable and independent prognostic biomarker of survival in oral squamous cell carcinoma (part 1 of 3)
Source: PeerJ. 2018 Aug 13;6:e5307. doi: 10.7717/peerj.5307 (PMC6095106; doi:10.7717/peerj.5307)

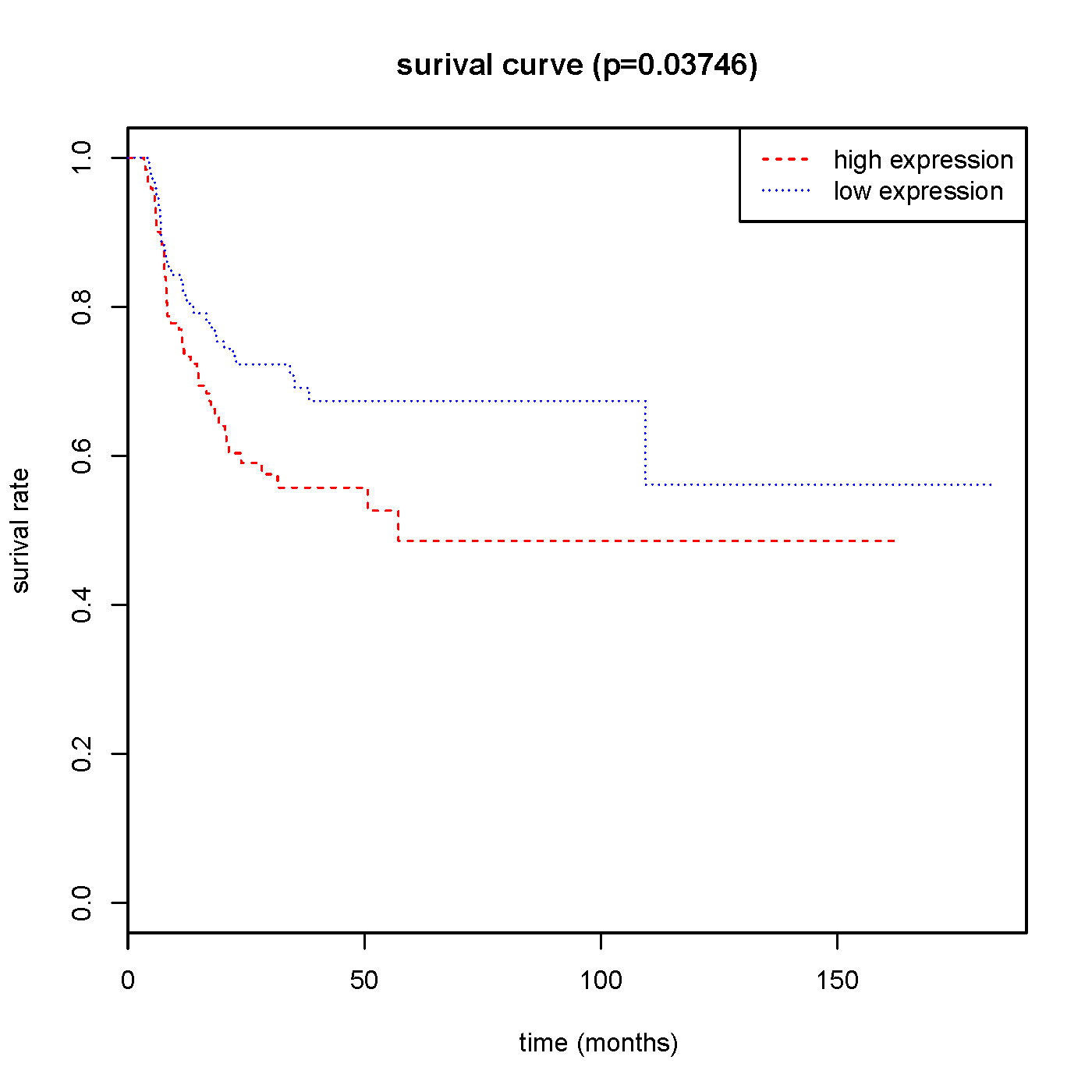

Supplement: Dataset S1 — Kaplan–Meier survival analysis with the log-rank test was used to identify relationships between the above 2493 lncRNA signatures and OSCC patient survival. Then, we determined the levels of 126 lncRNA signatures that were significantly related to DFS. [file peerj-06-5307-s005.zip › The result of Kaplan–Meier survival analyses and log-rank tests for DFS in OSCC/AC002306.1.jpg]

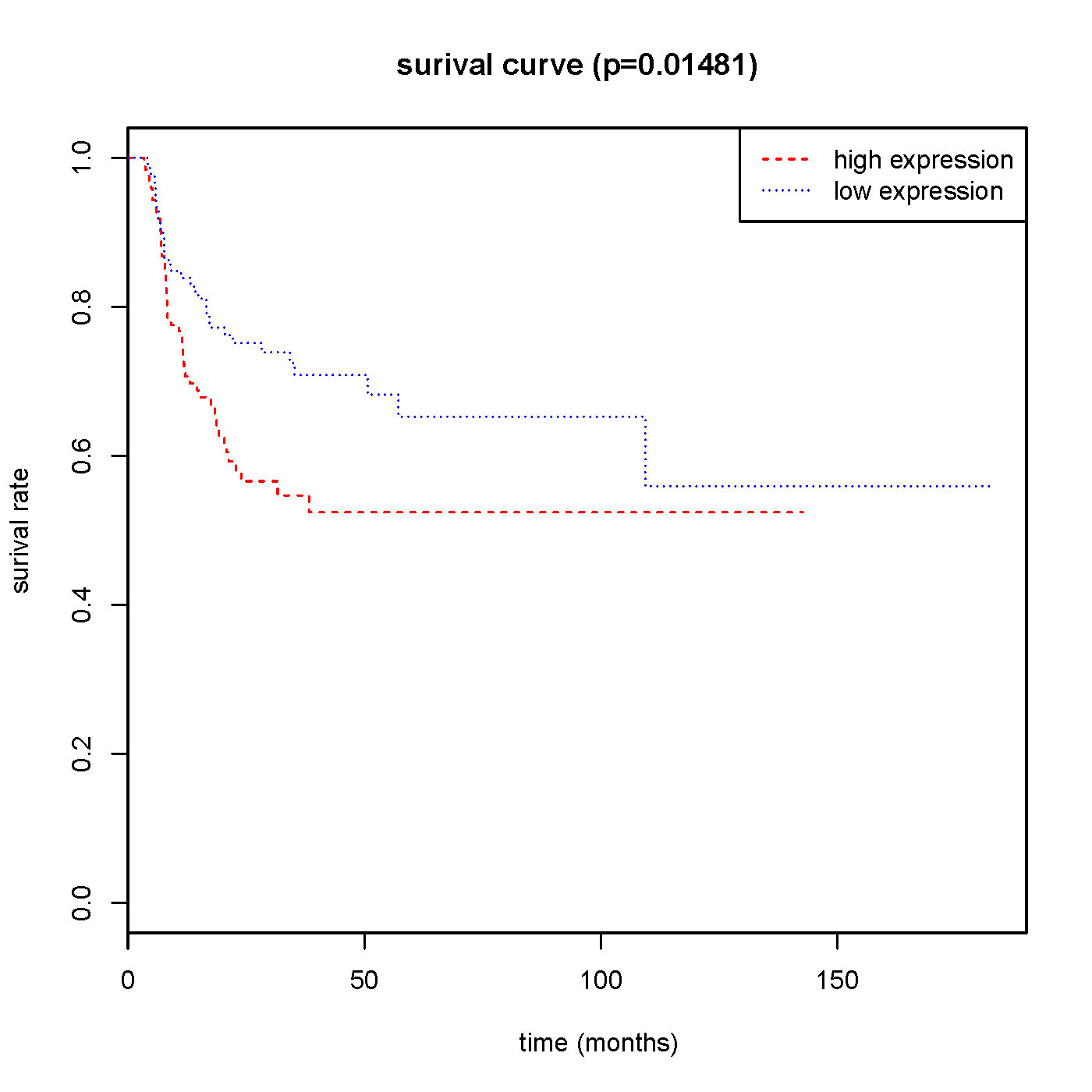

Supplement: Dataset S1 — Kaplan–Meier survival analysis with the log-rank test was used to identify relationships between the above 2493 lncRNA signatures and OSCC patient survival. Then, we determined the levels of 126 lncRNA signatures that were significantly related to DFS. [file peerj-06-5307-s005.zip › The result of Kaplan–Meier survival analyses and log-rank tests for DFS in OSCC/AC005387.2.jpg]

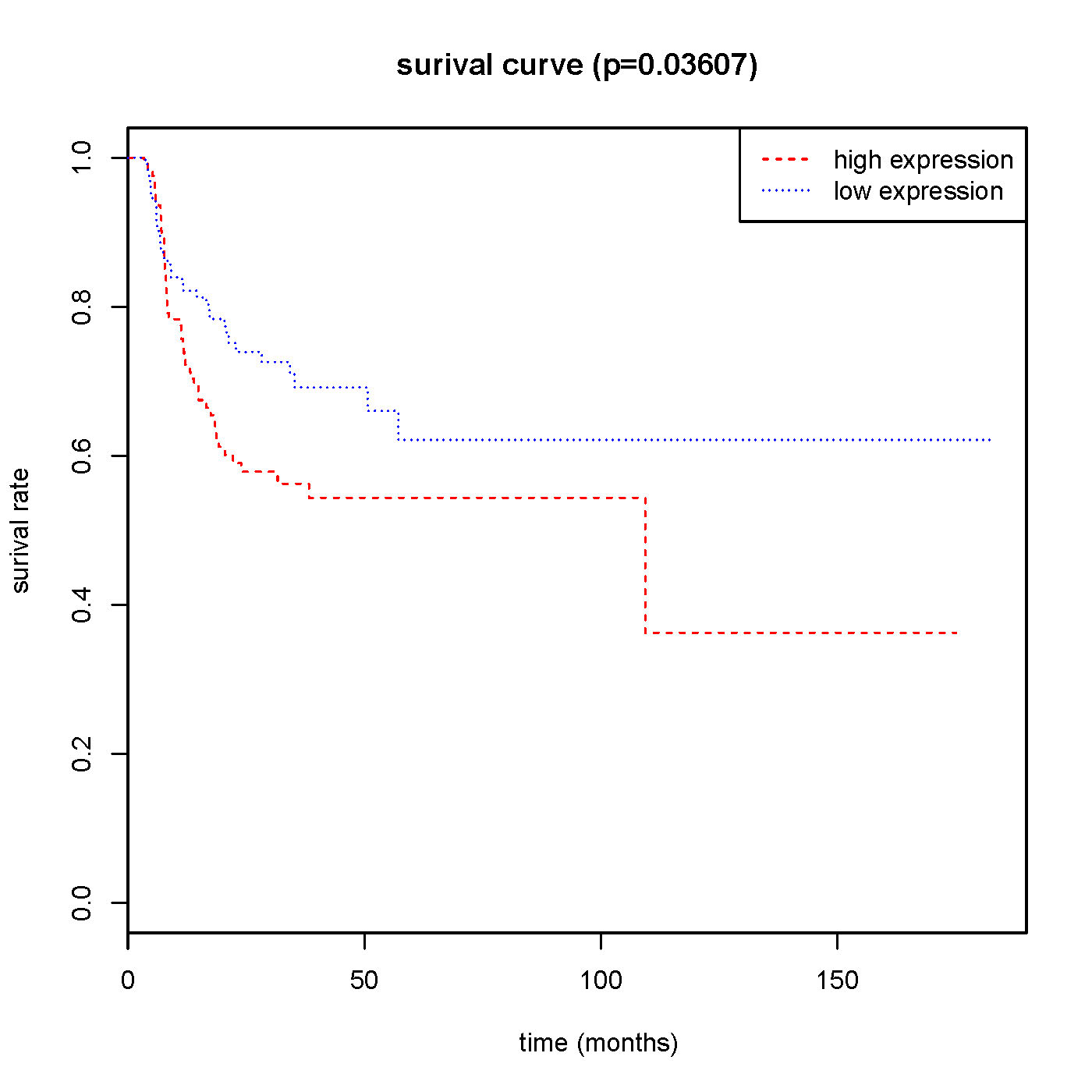

Supplement: Dataset S1 — Kaplan–Meier survival analysis with the log-rank test was used to identify relationships between the above 2493 lncRNA signatures and OSCC patient survival. Then, we determined the levels of 126 lncRNA signatures that were significantly related to DFS. [file peerj-06-5307-s005.zip › The result of Kaplan–Meier survival analyses and log-rank tests for DFS in OSCC/AC007879.2.jpg]

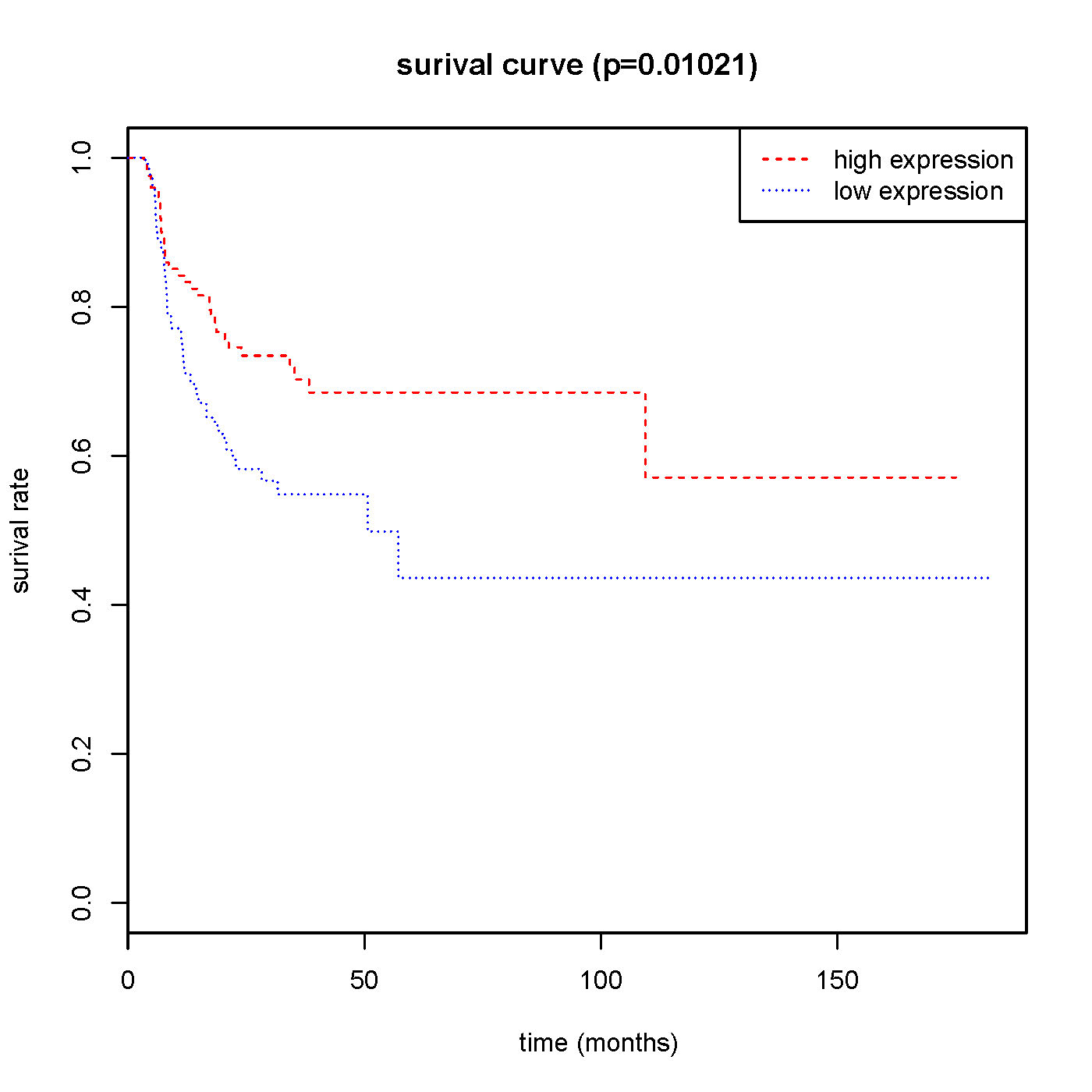

Supplement: Dataset S1 — Kaplan–Meier survival analysis with the log-rank test was used to identify relationships between the above 2493 lncRNA signatures and OSCC patient survival. Then, we determined the levels of 126 lncRNA signatures that were significantly related to DFS. [file peerj-06-5307-s005.zip › The result of Kaplan–Meier survival analyses and log-rank tests for DFS in OSCC/AC009950.2.jpg]

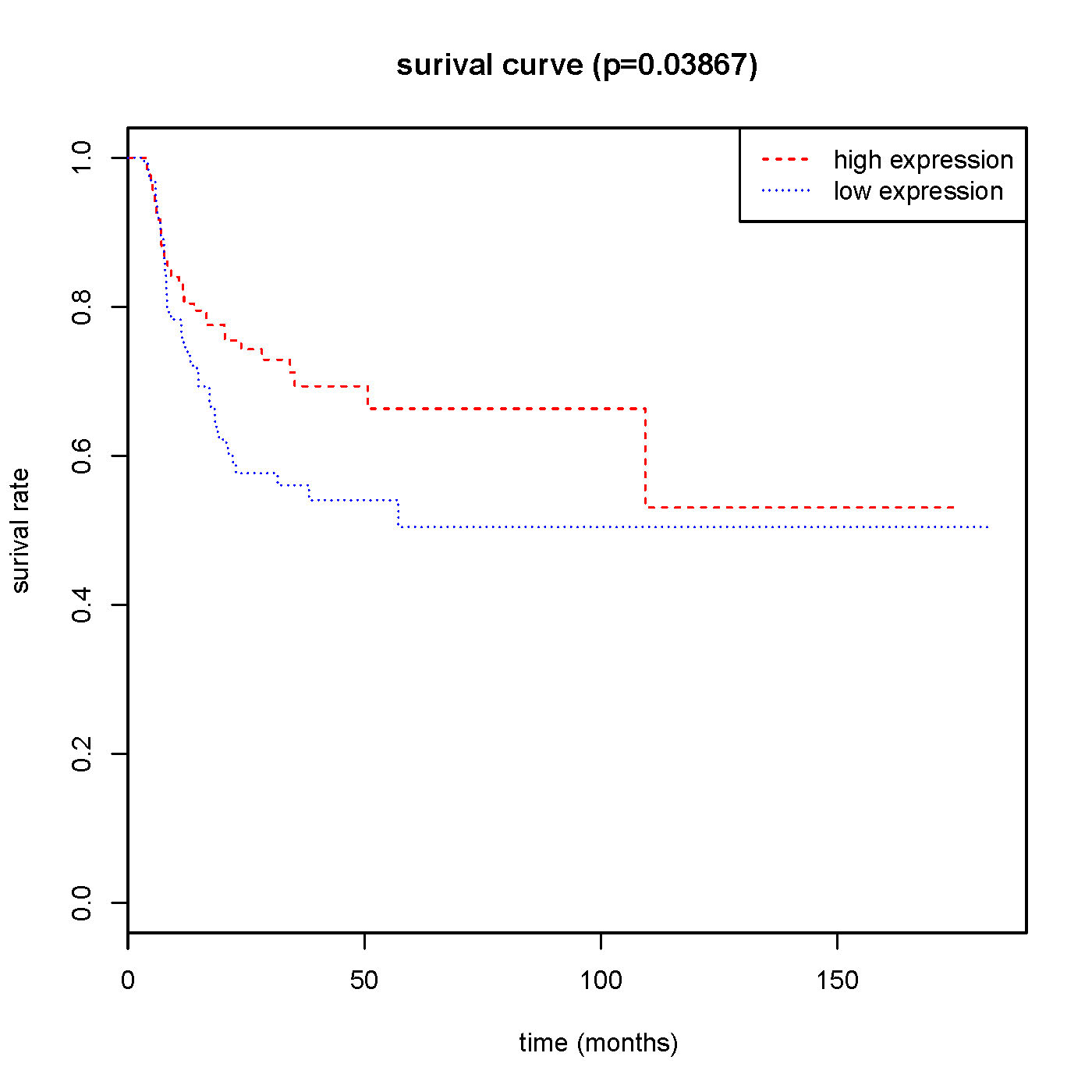

Supplement: Dataset S1 — Kaplan–Meier survival analysis with the log-rank test was used to identify relationships between the above 2493 lncRNA signatures and OSCC patient survival. Then, we determined the levels of 126 lncRNA signatures that were significantly related to DFS. [file peerj-06-5307-s005.zip › The result of Kaplan–Meier survival analyses and log-rank tests for DFS in OSCC/AC010226.4.jpg]

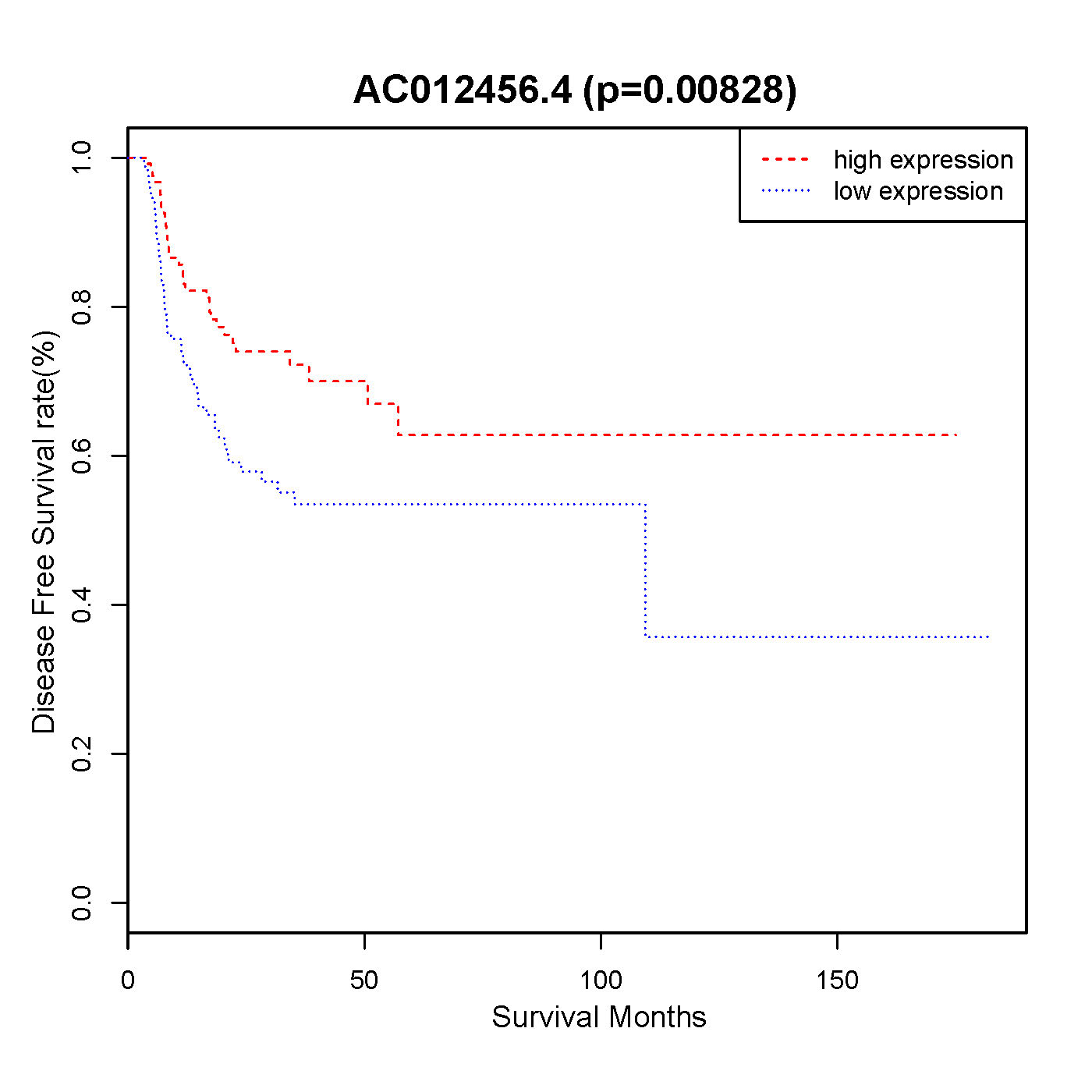

Supplement: Dataset S1 — Kaplan–Meier survival analysis with the log-rank test was used to identify relationships between the above 2493 lncRNA signatures and OSCC patient survival. Then, we determined the levels of 126 lncRNA signatures that were significantly related to DFS. [file peerj-06-5307-s005.zip › The result of Kaplan–Meier survival analyses and log-rank tests for DFS in OSCC/AC012456.4.jpg]

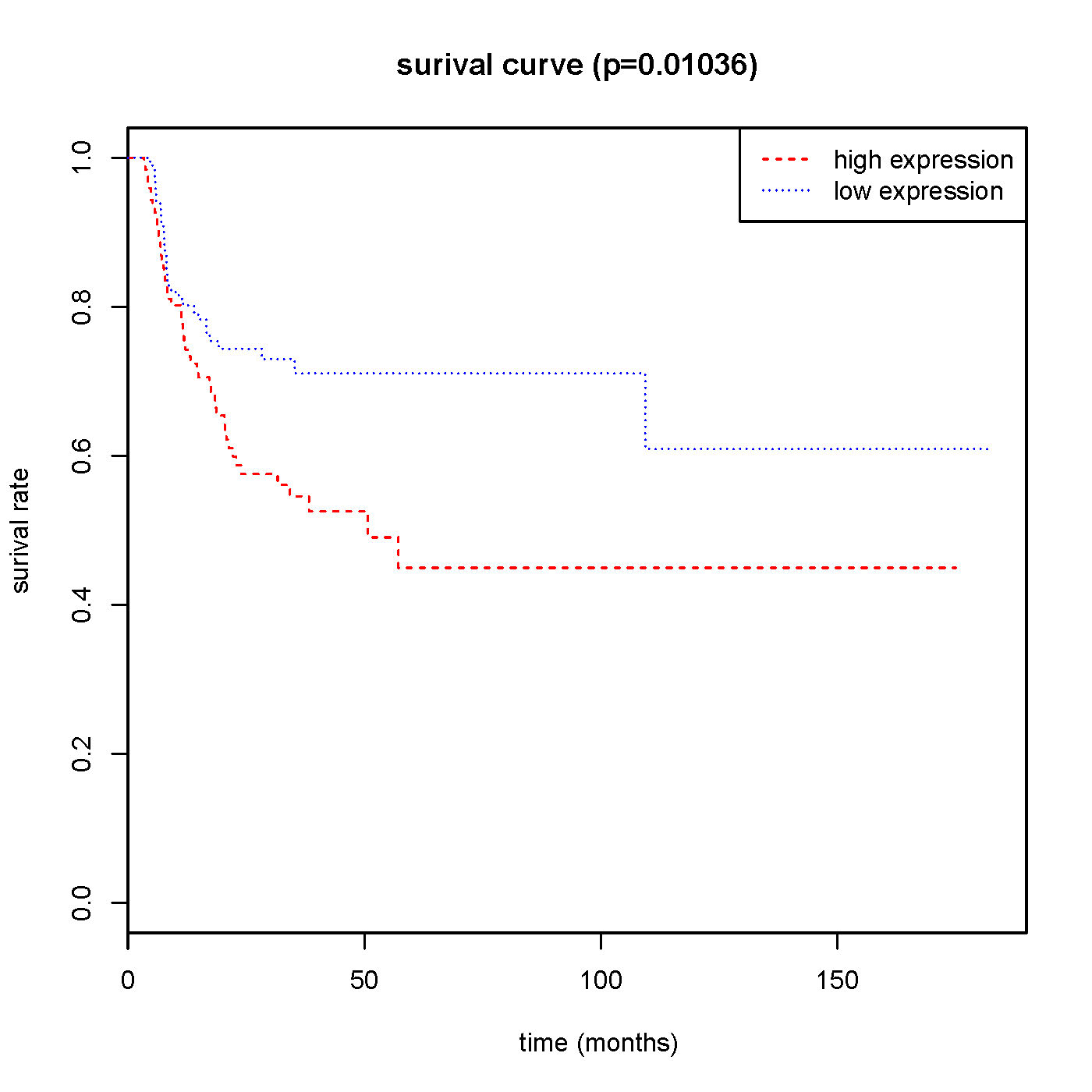

Supplement: Dataset S1 — Kaplan–Meier survival analysis with the log-rank test was used to identify relationships between the above 2493 lncRNA signatures and OSCC patient survival. Then, we determined the levels of 126 lncRNA signatures that were significantly related to DFS. [file peerj-06-5307-s005.zip › The result of Kaplan–Meier survival analyses and log-rank tests for DFS in OSCC/AC025165.8.jpg]

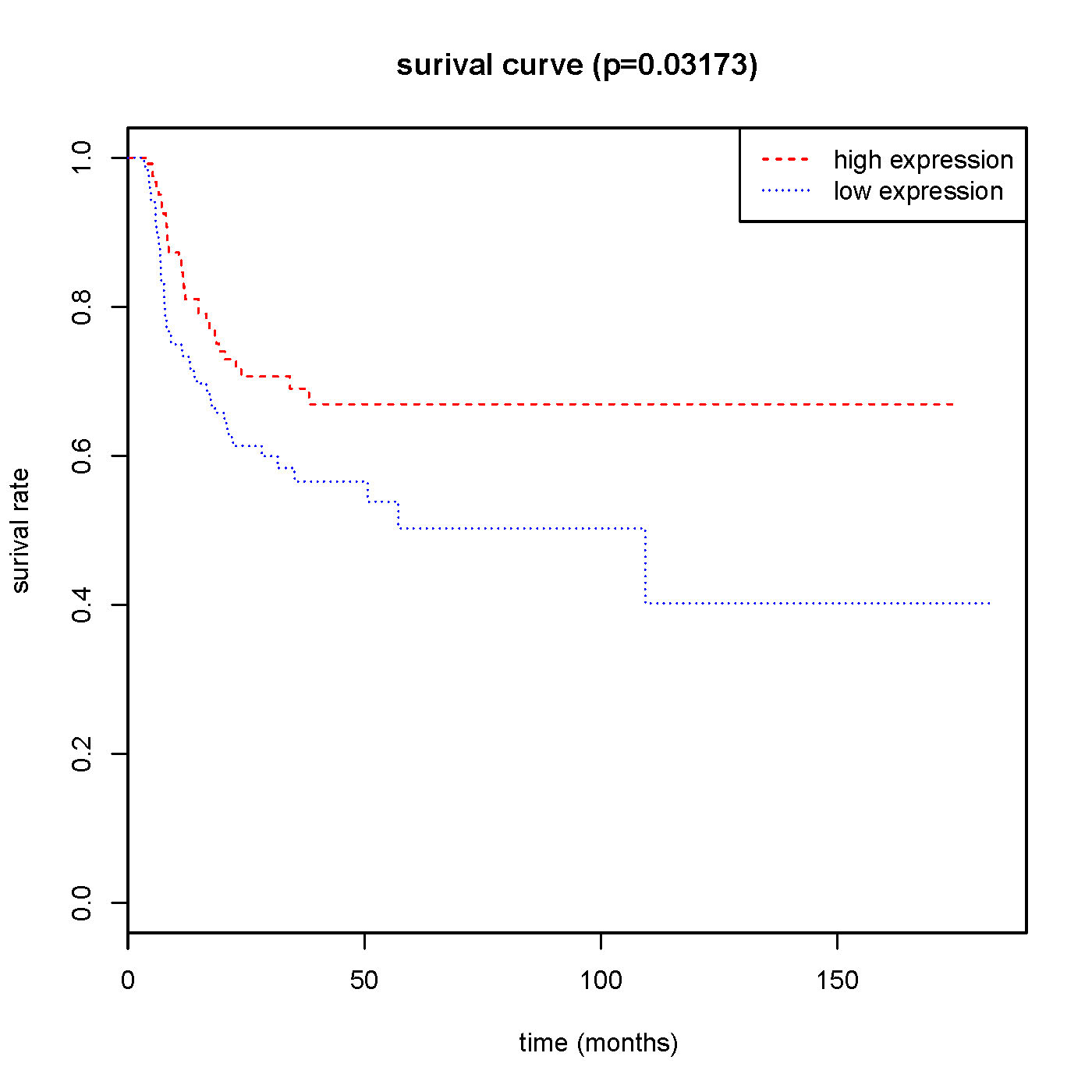

Supplement: Dataset S1 — Kaplan–Meier survival analysis with the log-rank test was used to identify relationships between the above 2493 lncRNA signatures and OSCC patient survival. Then, we determined the levels of 126 lncRNA signatures that were significantly related to DFS. [file peerj-06-5307-s005.zip › The result of Kaplan–Meier survival analyses and log-rank tests for DFS in OSCC/AC091814.3.jpg]

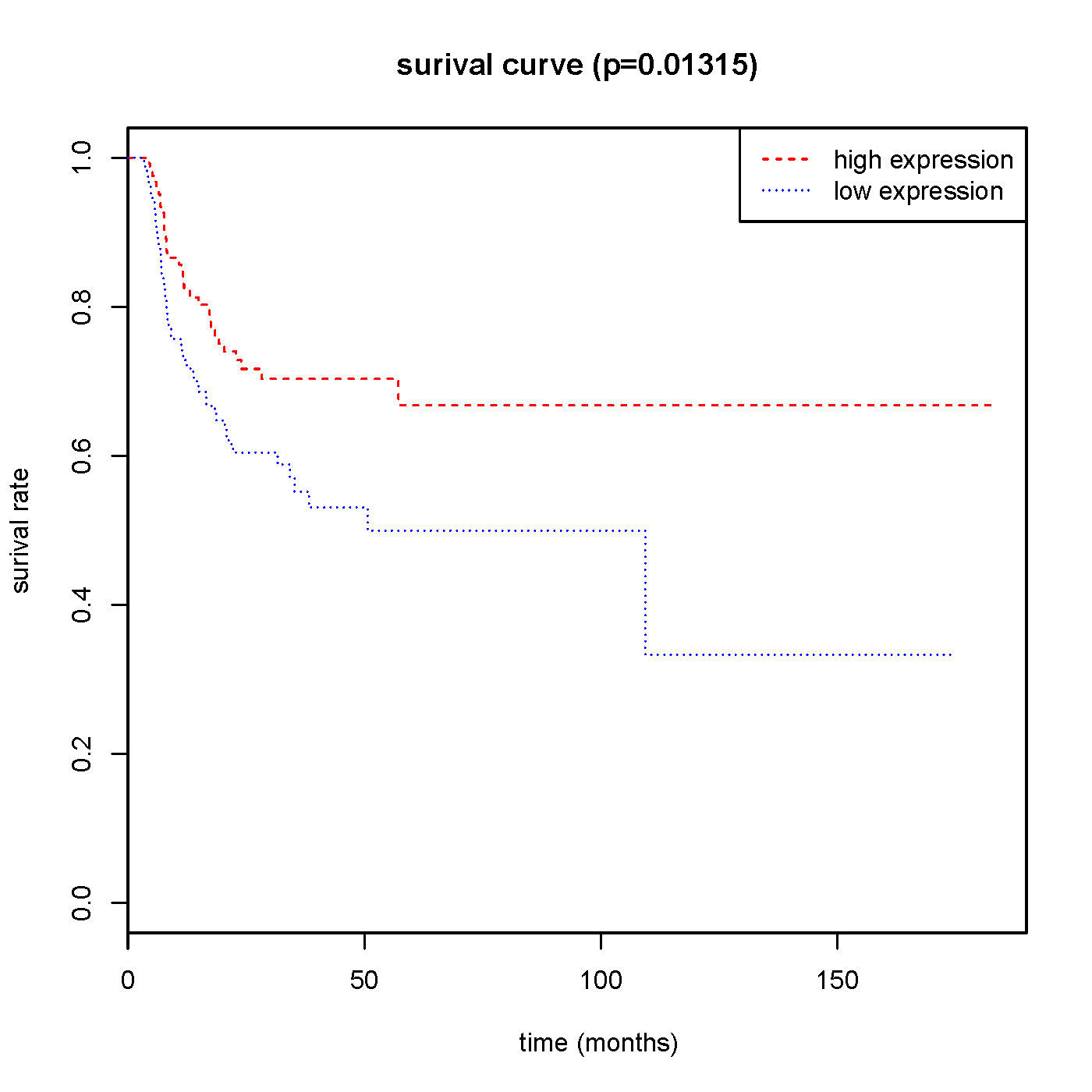

Supplement: Dataset S1 — Kaplan–Meier survival analysis with the log-rank test was used to identify relationships between the above 2493 lncRNA signatures and OSCC patient survival. Then, we determined the levels of 126 lncRNA signatures that were significantly related to DFS. [file peerj-06-5307-s005.zip › The result of Kaplan–Meier survival analyses and log-rank tests for DFS in OSCC/AC127904.2.jpg]

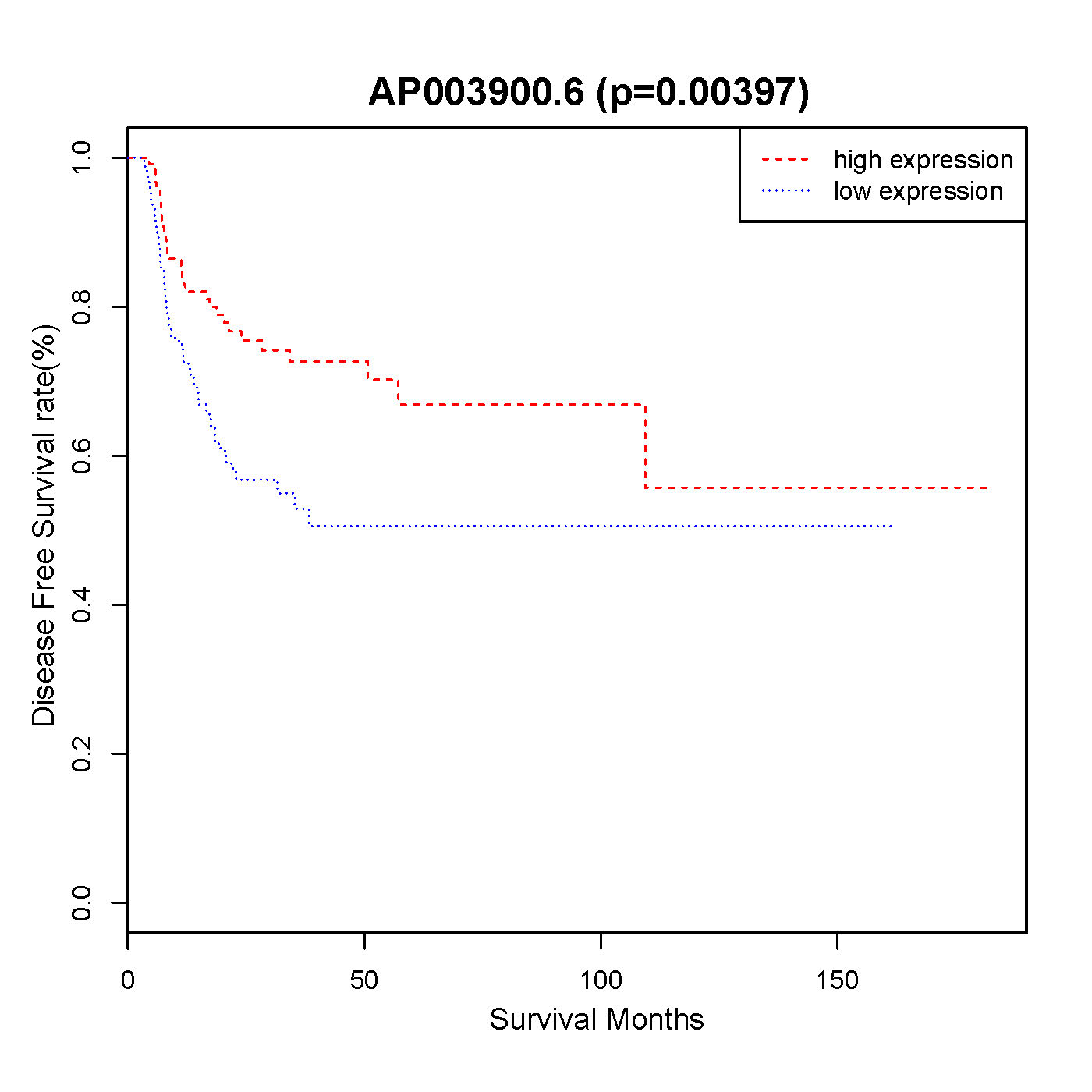

Supplement: Dataset S1 — Kaplan–Meier survival analysis with the log-rank test was used to identify relationships between the above 2493 lncRNA signatures and OSCC patient survival. Then, we determined the levels of 126 lncRNA signatures that were significantly related to DFS. [file peerj-06-5307-s005.zip › The result of Kaplan–Meier survival analyses and log-rank tests for DFS in OSCC/AP003900.6.jpg]

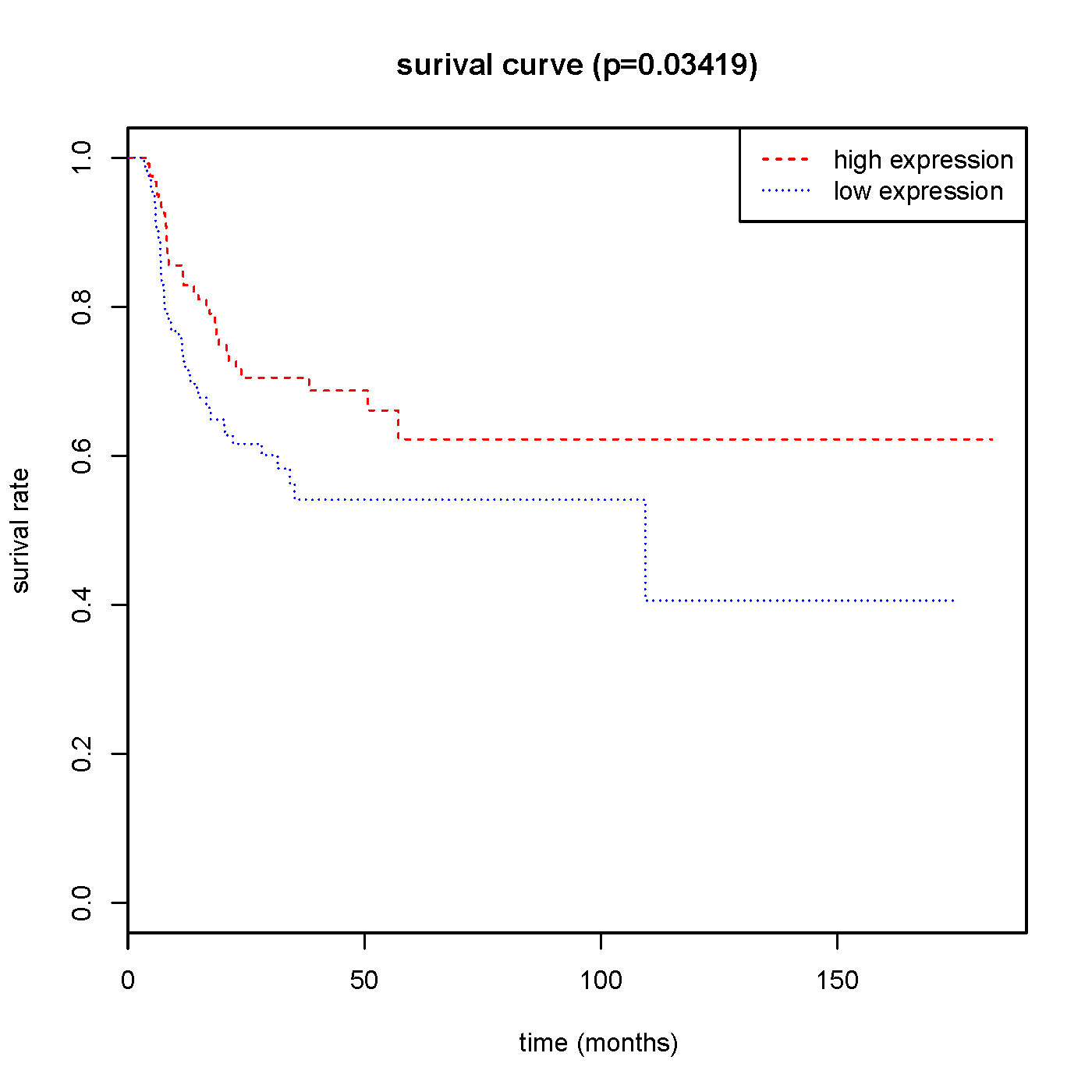

Supplement: Dataset S1 — Kaplan–Meier survival analysis with the log-rank test was used to identify relationships between the above 2493 lncRNA signatures and OSCC patient survival. Then, we determined the levels of 126 lncRNA signatures that were significantly related to DFS. [file peerj-06-5307-s005.zip › The result of Kaplan–Meier survival analyses and log-rank tests for DFS in OSCC/BLACAT1.jpg]

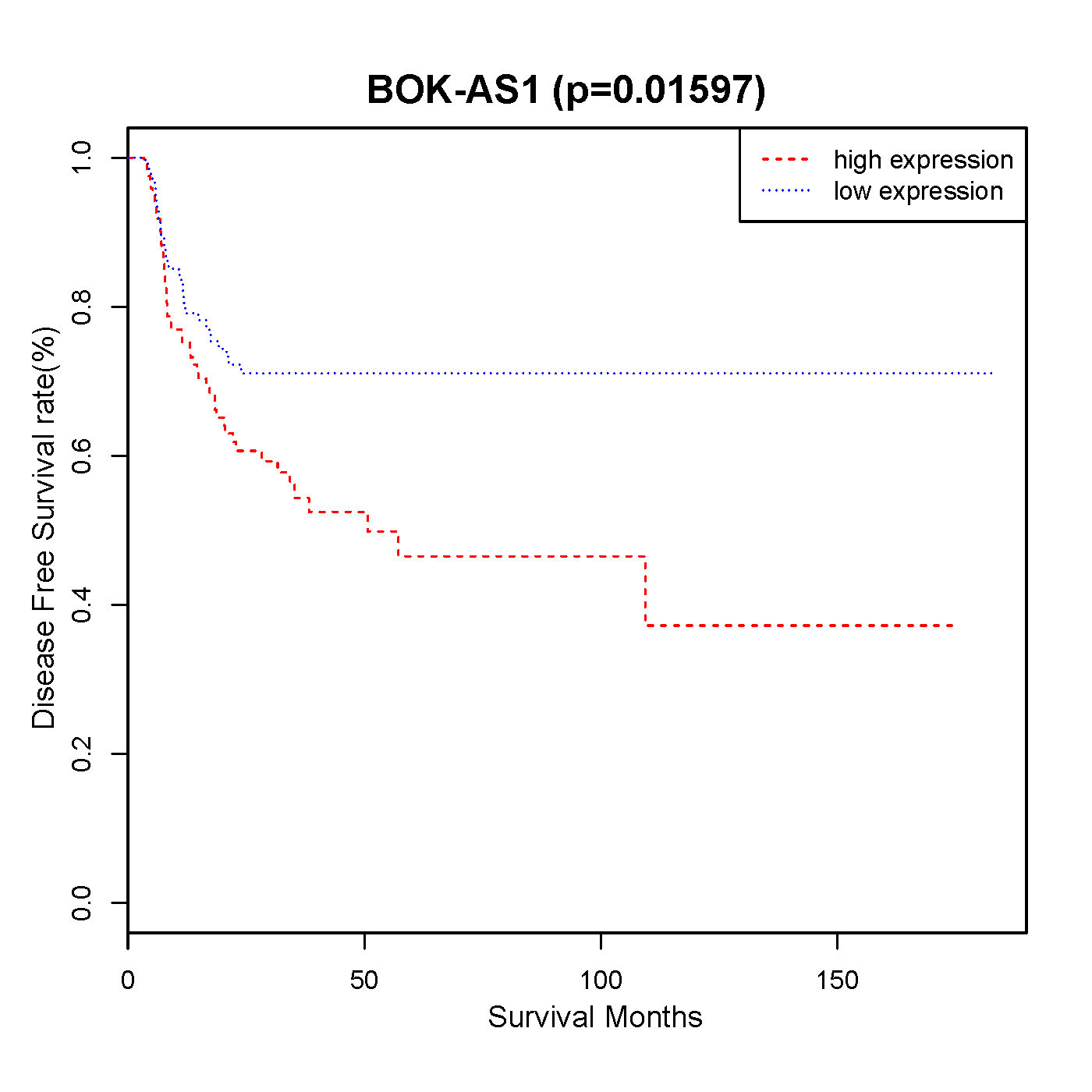

Supplement: Dataset S1 — Kaplan–Meier survival analysis with the log-rank test was used to identify relationships between the above 2493 lncRNA signatures and OSCC patient survival. Then, we determined the levels of 126 lncRNA signatures that were significantly related to DFS. [file peerj-06-5307-s005.zip › The result of Kaplan–Meier survival analyses and log-rank tests for DFS in OSCC/BOK-AS1.jpg]

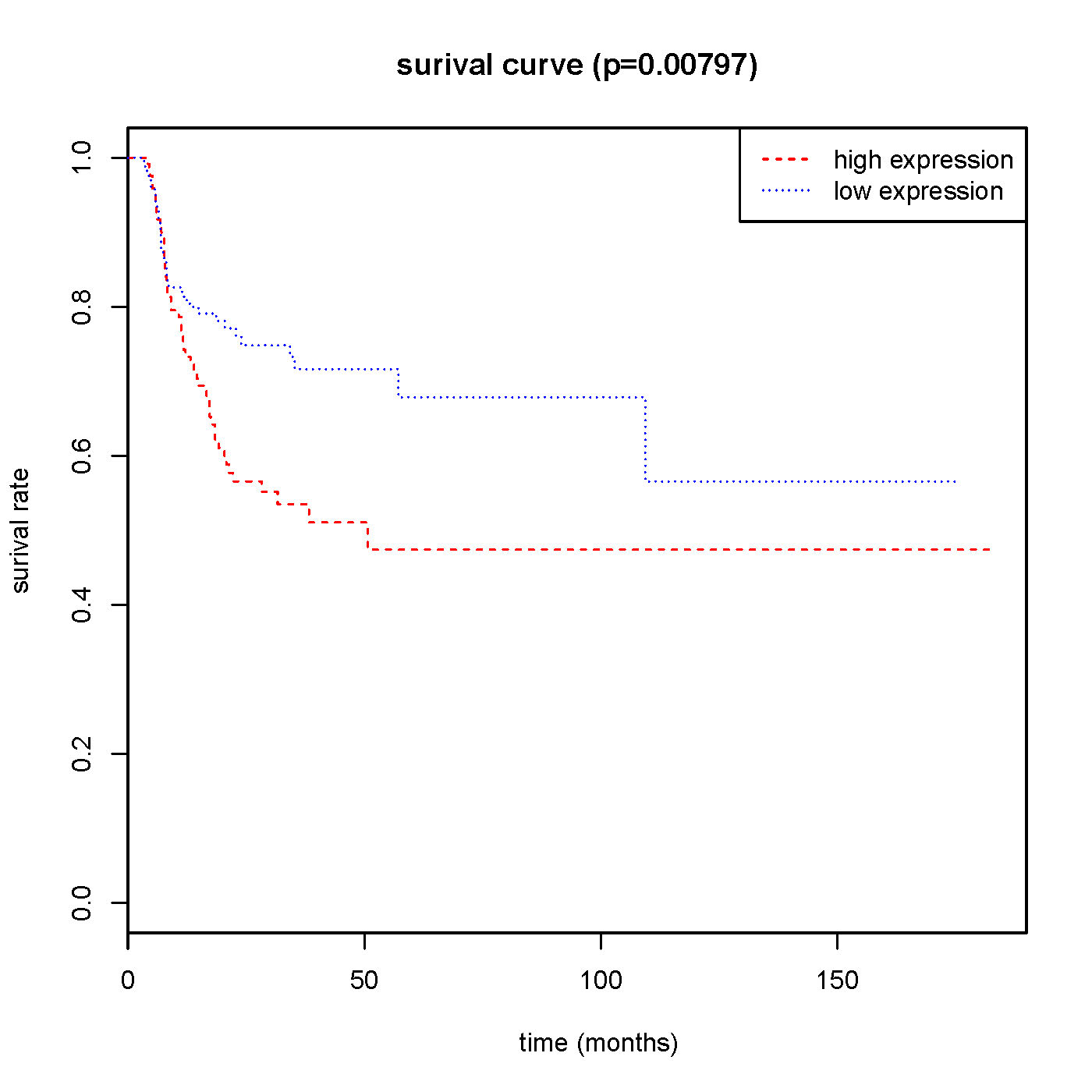

Supplement: Dataset S1 — Kaplan–Meier survival analysis with the log-rank test was used to identify relationships between the above 2493 lncRNA signatures and OSCC patient survival. Then, we determined the levels of 126 lncRNA signatures that were significantly related to DFS. [file peerj-06-5307-s005.zip › The result of Kaplan–Meier survival analyses and log-rank tests for DFS in OSCC/BX470102.3.jpg]

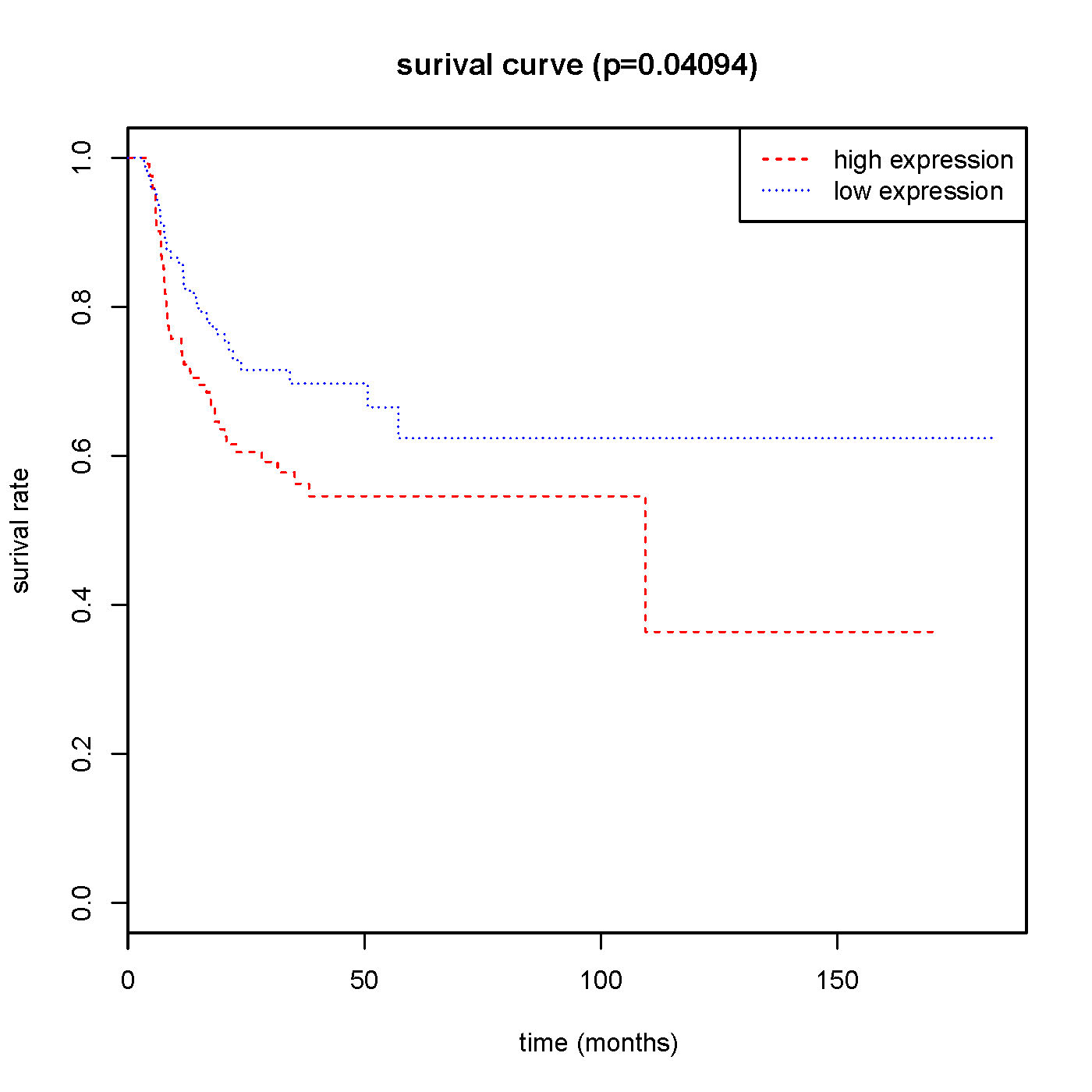

Supplement: Dataset S1 — Kaplan–Meier survival analysis with the log-rank test was used to identify relationships between the above 2493 lncRNA signatures and OSCC patient survival. Then, we determined the levels of 126 lncRNA signatures that were significantly related to DFS. [file peerj-06-5307-s005.zip › The result of Kaplan–Meier survival analyses and log-rank tests for DFS in OSCC/CASC11.jpg]

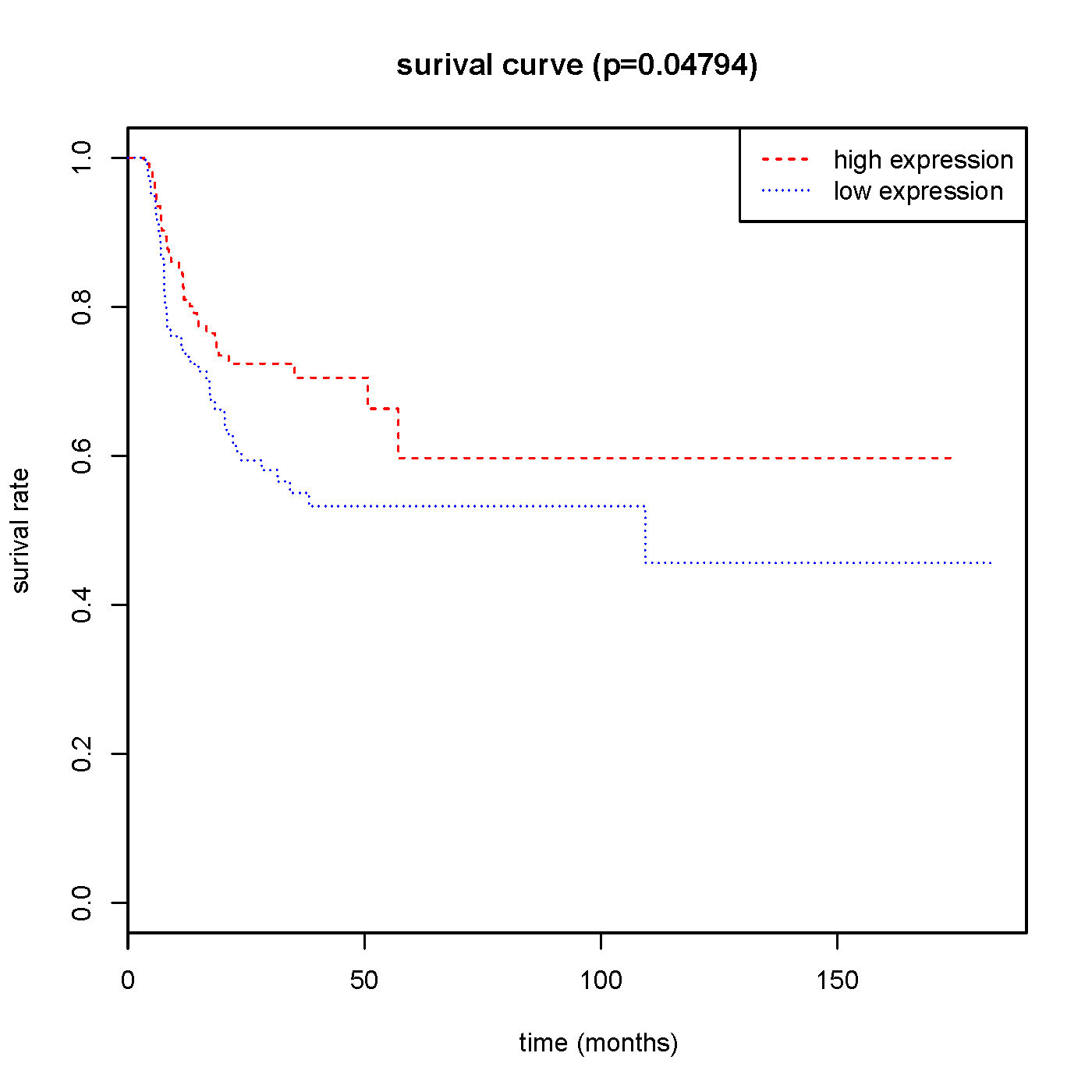

Supplement: Dataset S1 — Kaplan–Meier survival analysis with the log-rank test was used to identify relationships between the above 2493 lncRNA signatures and OSCC patient survival. Then, we determined the levels of 126 lncRNA signatures that were significantly related to DFS. [file peerj-06-5307-s005.zip › The result of Kaplan–Meier survival analyses and log-rank tests for DFS in OSCC/CH507-42P11.6.jpg]

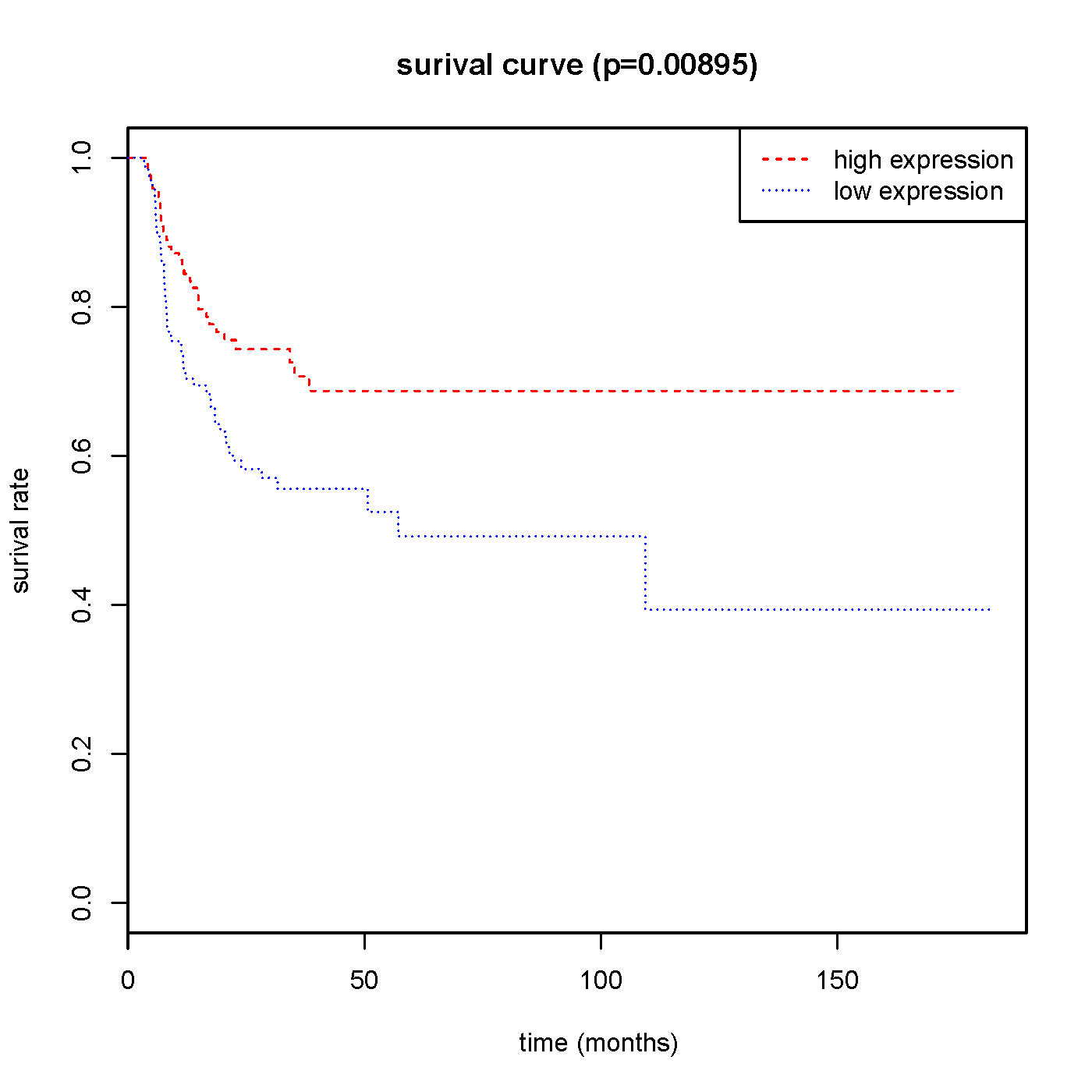

Supplement: Dataset S1 — Kaplan–Meier survival analysis with the log-rank test was used to identify relationships between the above 2493 lncRNA signatures and OSCC patient survival. Then, we determined the levels of 126 lncRNA signatures that were significantly related to DFS. [file peerj-06-5307-s005.zip › The result of Kaplan–Meier survival analyses and log-rank tests for DFS in OSCC/CTA-384D8.31.jpg]

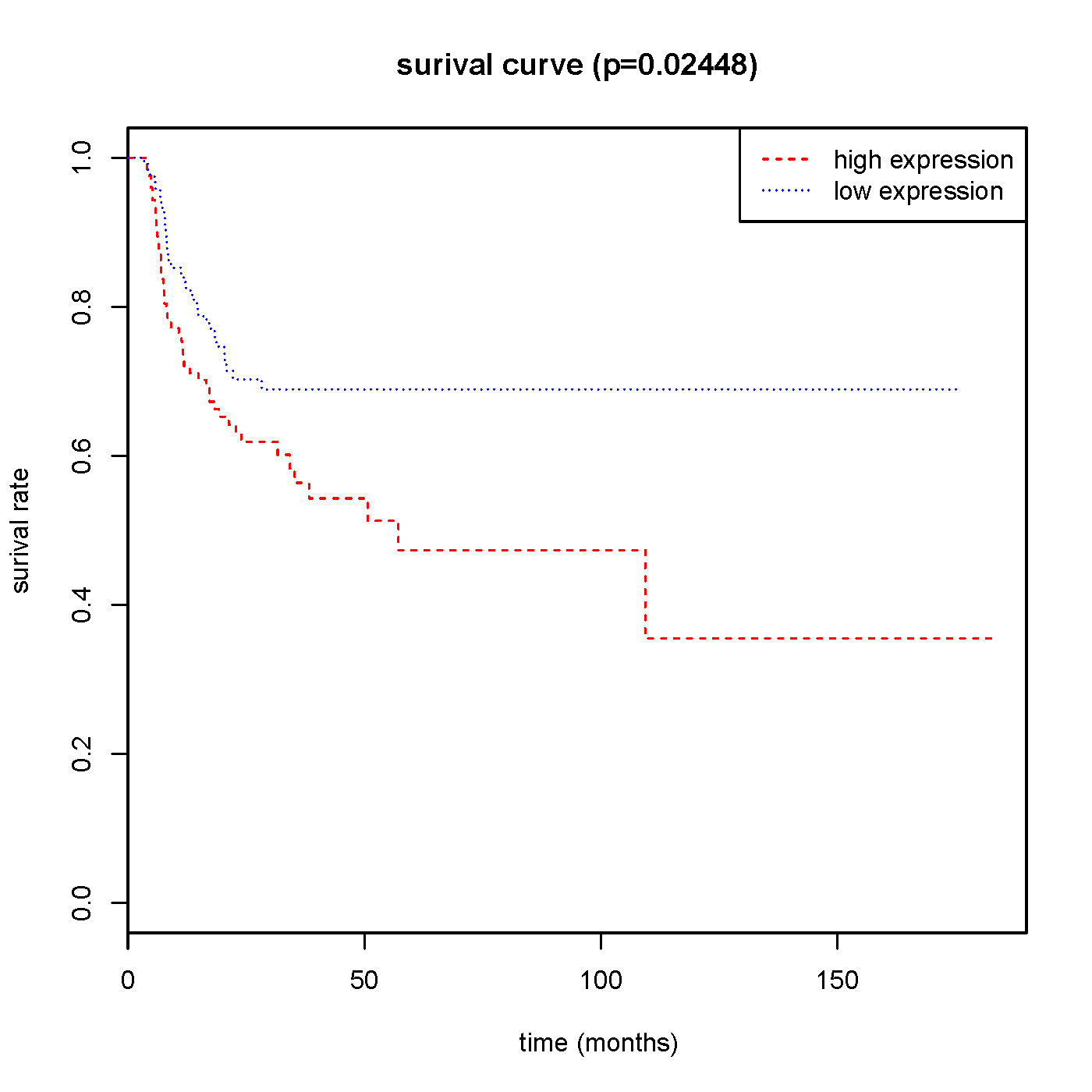

Supplement: Dataset S1 — Kaplan–Meier survival analysis with the log-rank test was used to identify relationships between the above 2493 lncRNA signatures and OSCC patient survival. Then, we determined the levels of 126 lncRNA signatures that were significantly related to DFS. [file peerj-06-5307-s005.zip › The result of Kaplan–Meier survival analyses and log-rank tests for DFS in OSCC/CTB-113D17.1.jpg]

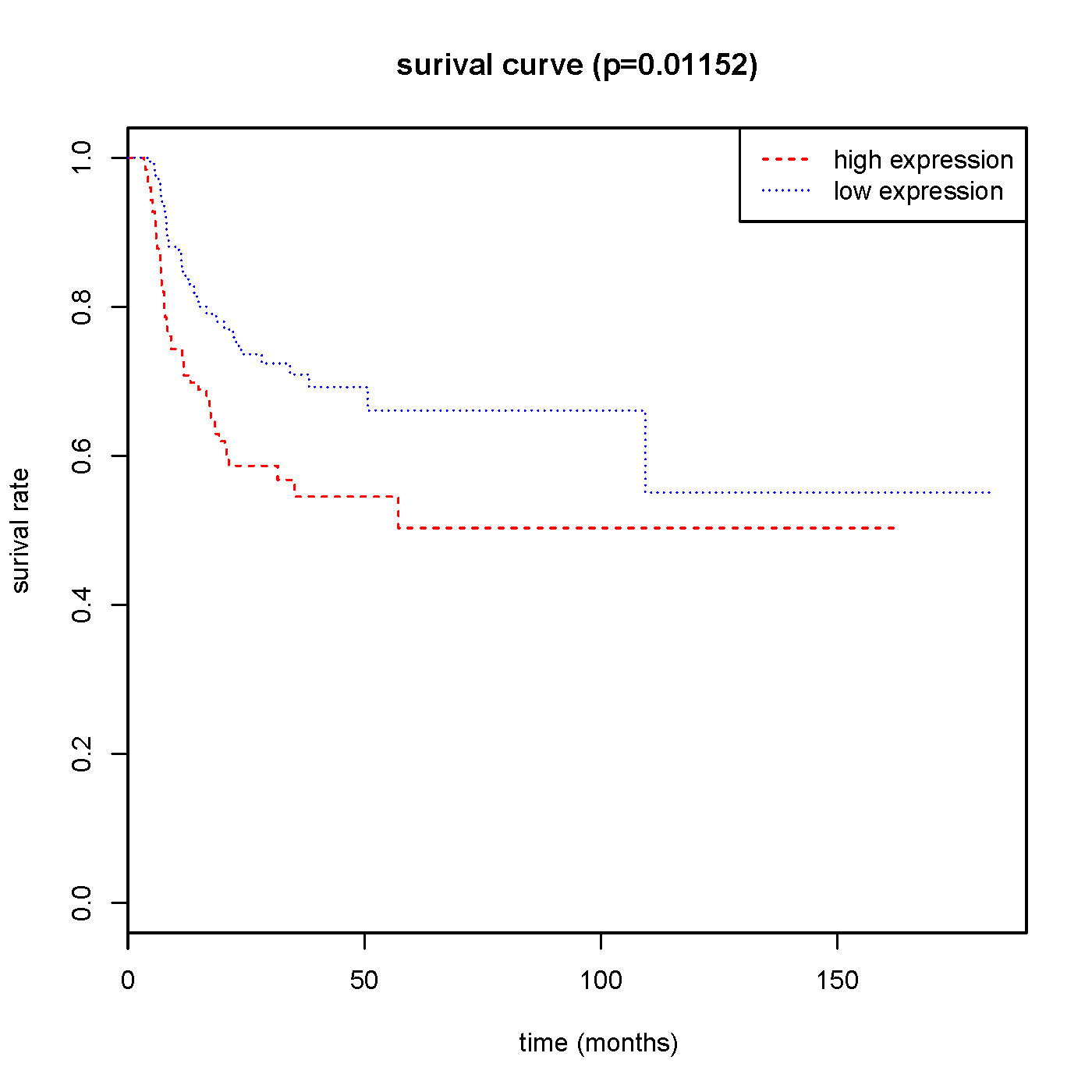

Supplement: Dataset S1 — Kaplan–Meier survival analysis with the log-rank test was used to identify relationships between the above 2493 lncRNA signatures and OSCC patient survival. Then, we determined the levels of 126 lncRNA signatures that were significantly related to DFS. [file peerj-06-5307-s005.zip › The result of Kaplan–Meier survival analyses and log-rank tests for DFS in OSCC/CTB-161M19.4.jpg]

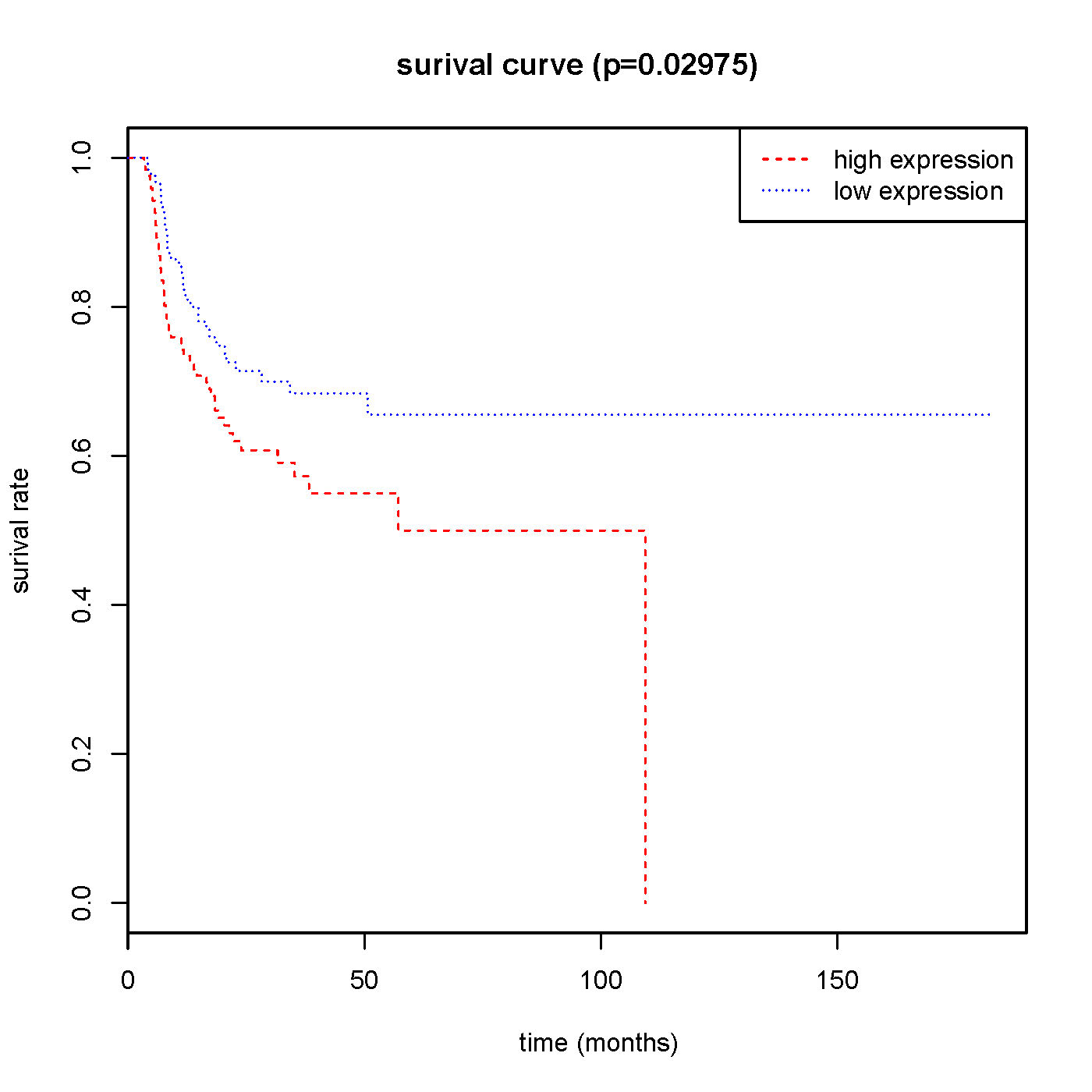

Supplement: Dataset S1 — Kaplan–Meier survival analysis with the log-rank test was used to identify relationships between the above 2493 lncRNA signatures and OSCC patient survival. Then, we determined the levels of 126 lncRNA signatures that were significantly related to DFS. [file peerj-06-5307-s005.zip › The result of Kaplan–Meier survival analyses and log-rank tests for DFS in OSCC/CTB-40H15.4.jpg]

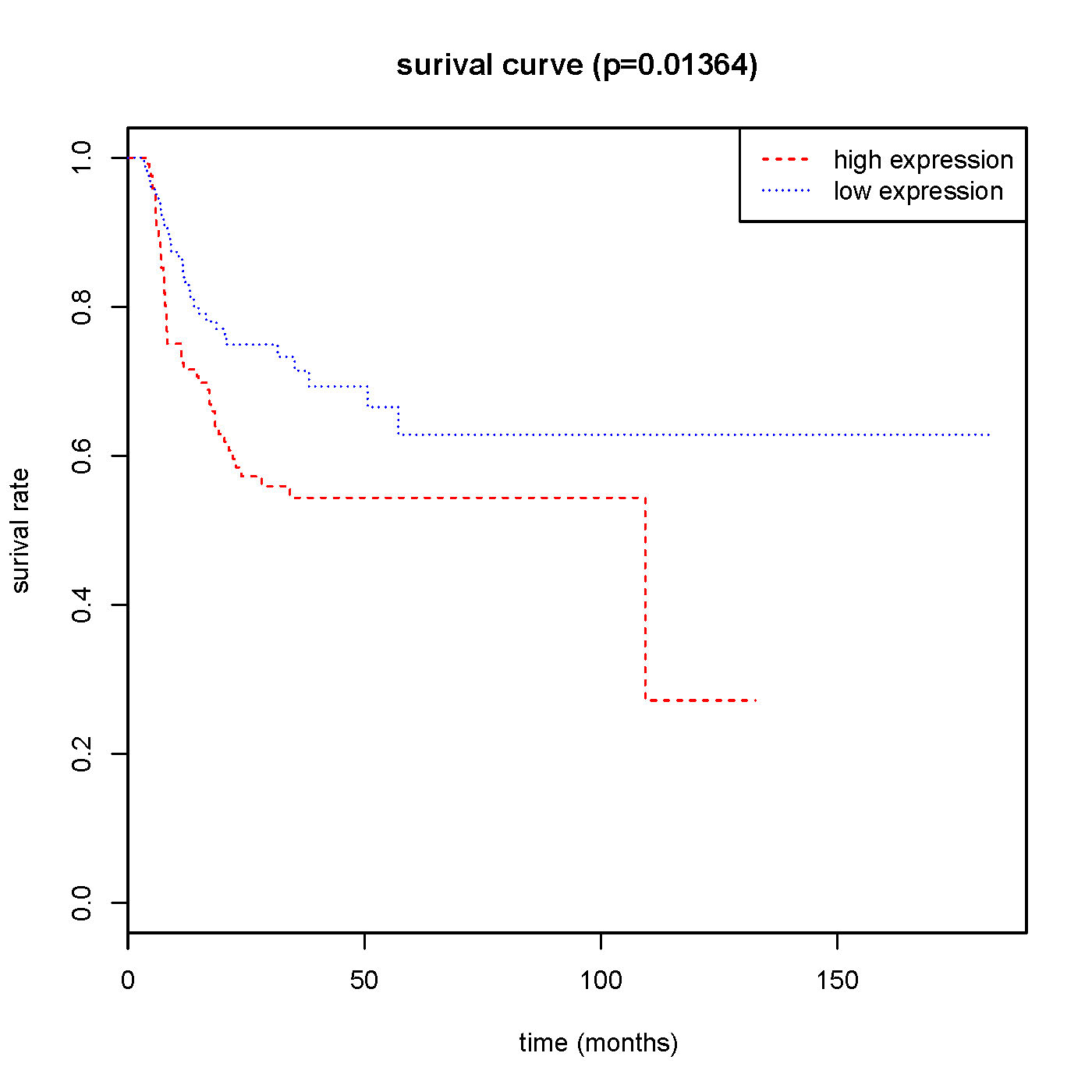

Supplement: Dataset S1 — Kaplan–Meier survival analysis with the log-rank test was used to identify relationships between the above 2493 lncRNA signatures and OSCC patient survival. Then, we determined the levels of 126 lncRNA signatures that were significantly related to DFS. [file peerj-06-5307-s005.zip › The result of Kaplan–Meier survival analyses and log-rank tests for DFS in OSCC/CTC-527H23.3.jpg]

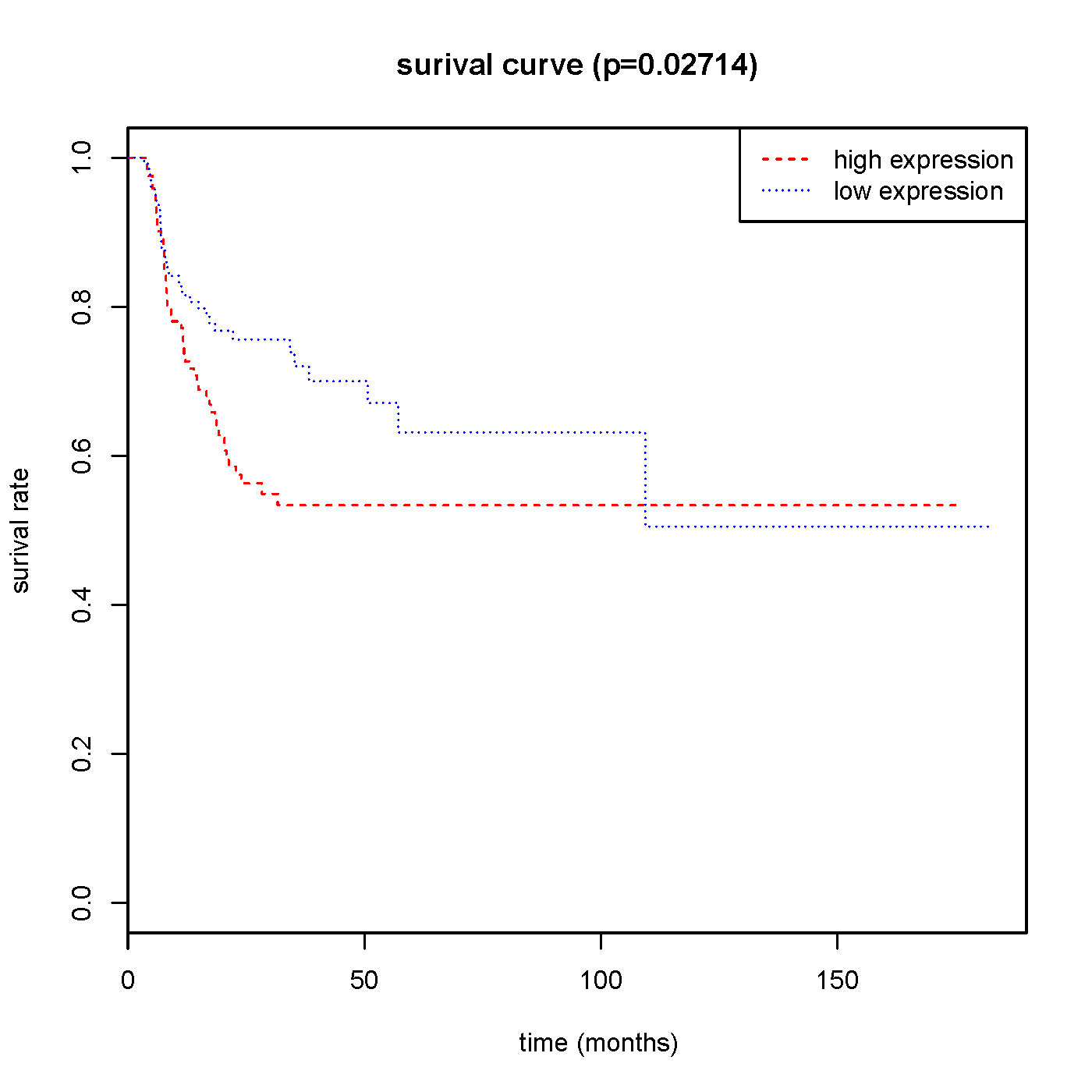

Supplement: Dataset S1 — Kaplan–Meier survival analysis with the log-rank test was used to identify relationships between the above 2493 lncRNA signatures and OSCC patient survival. Then, we determined the levels of 126 lncRNA signatures that were significantly related to DFS. [file peerj-06-5307-s005.zip › The result of Kaplan–Meier survival analyses and log-rank tests for DFS in OSCC/CTD-2033A16.3.jpg]

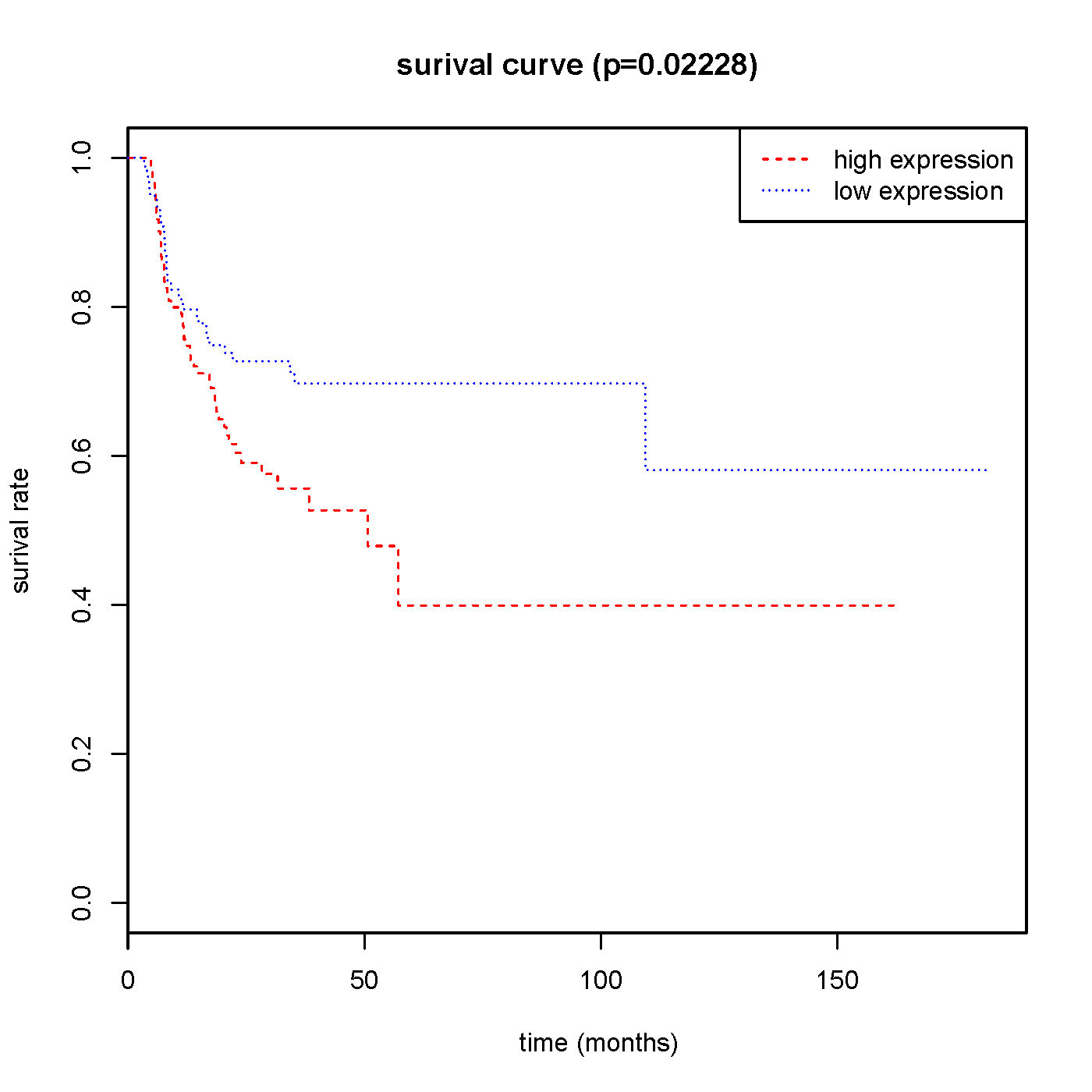

Supplement: Dataset S1 — Kaplan–Meier survival analysis with the log-rank test was used to identify relationships between the above 2493 lncRNA signatures and OSCC patient survival. Then, we determined the levels of 126 lncRNA signatures that were significantly related to DFS. [file peerj-06-5307-s005.zip › The result of Kaplan–Meier survival analyses and log-rank tests for DFS in OSCC/CTD-2227E11.1.jpg]

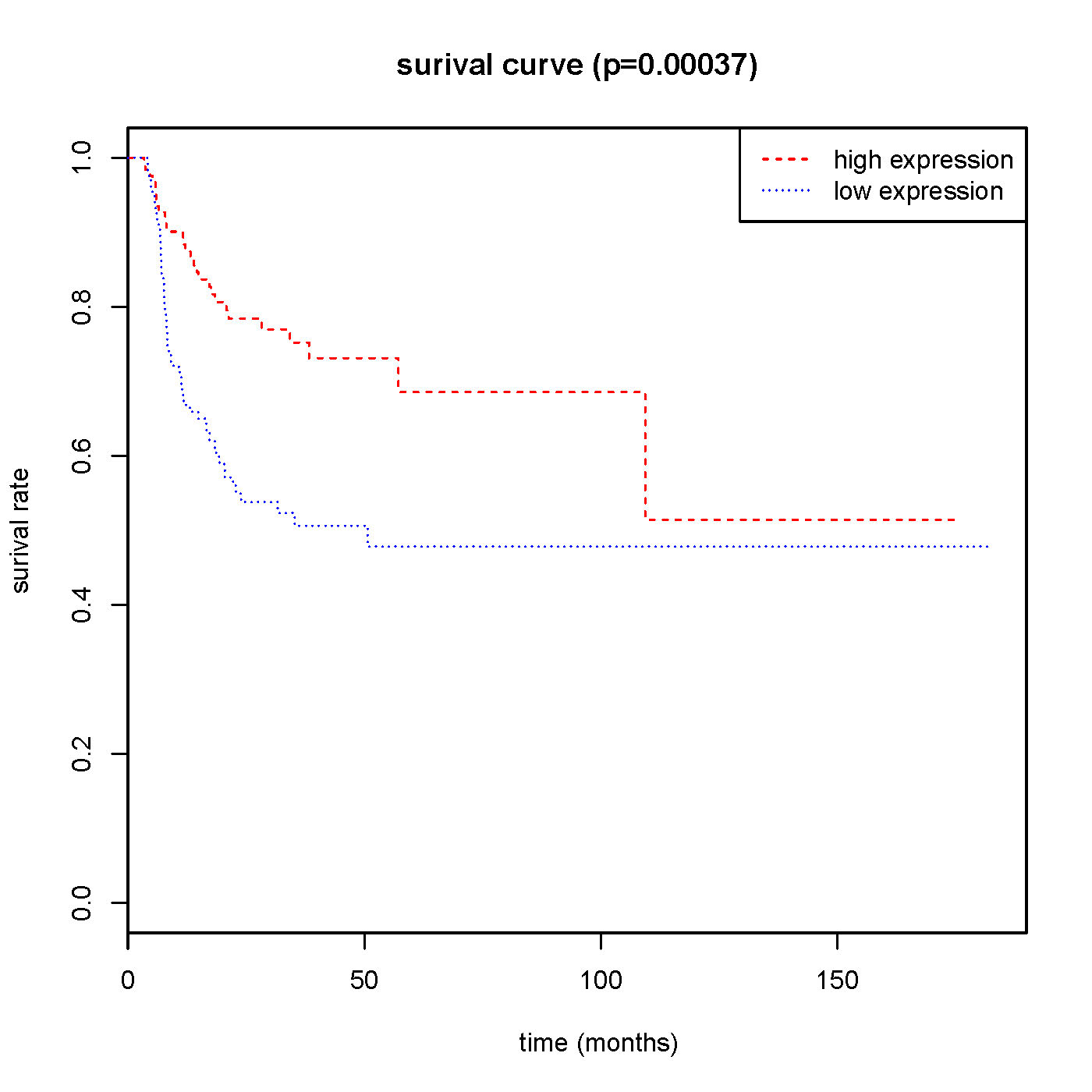

Supplement: Dataset S1 — Kaplan–Meier survival analysis with the log-rank test was used to identify relationships between the above 2493 lncRNA signatures and OSCC patient survival. Then, we determined the levels of 126 lncRNA signatures that were significantly related to DFS. [file peerj-06-5307-s005.zip › The result of Kaplan–Meier survival analyses and log-rank tests for DFS in OSCC/CTD-2330K9.3.jpg]

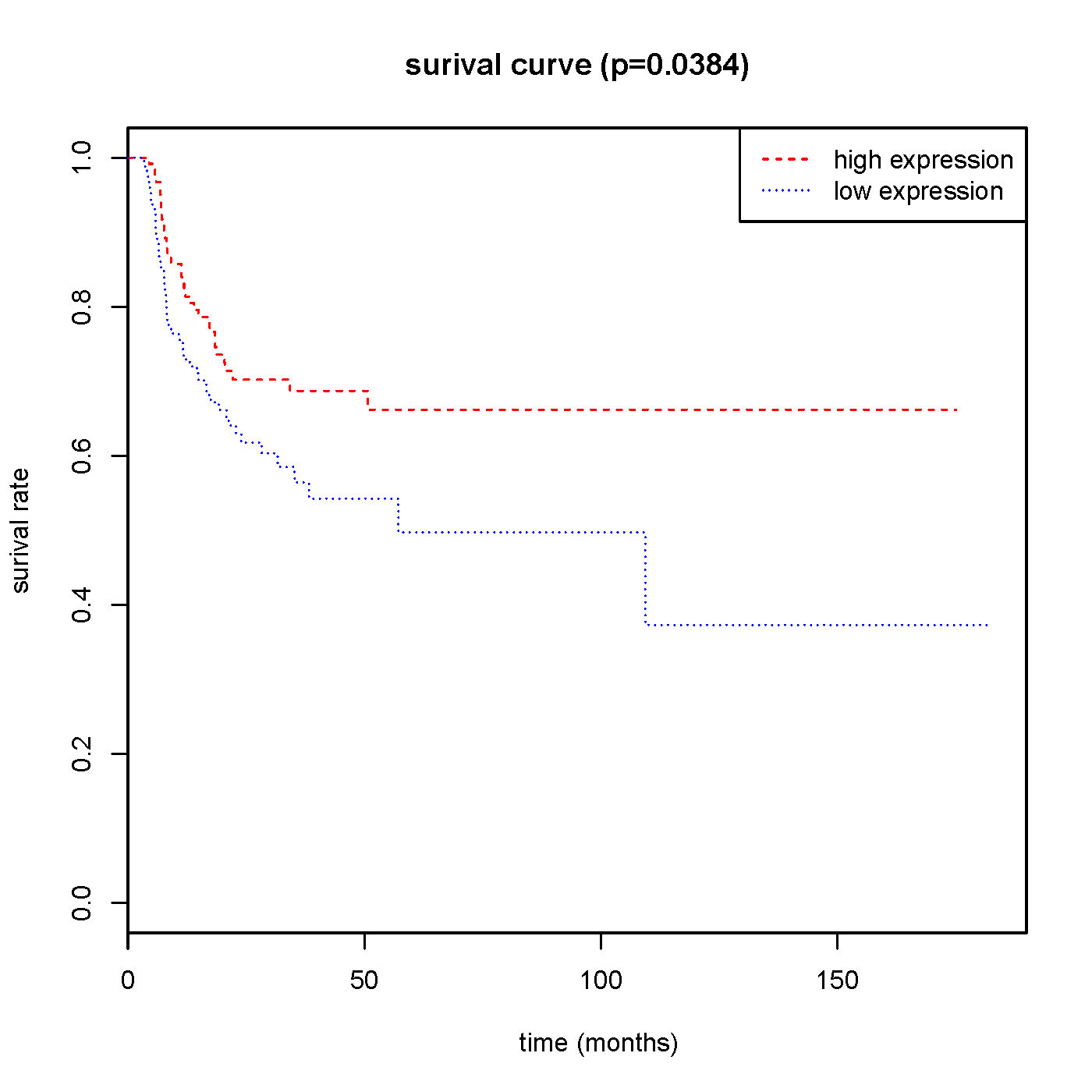

Supplement: Dataset S1 — Kaplan–Meier survival analysis with the log-rank test was used to identify relationships between the above 2493 lncRNA signatures and OSCC patient survival. Then, we determined the levels of 126 lncRNA signatures that were significantly related to DFS. [file peerj-06-5307-s005.zip › The result of Kaplan–Meier survival analyses and log-rank tests for DFS in OSCC/CTD-2535I10.1.jpg]

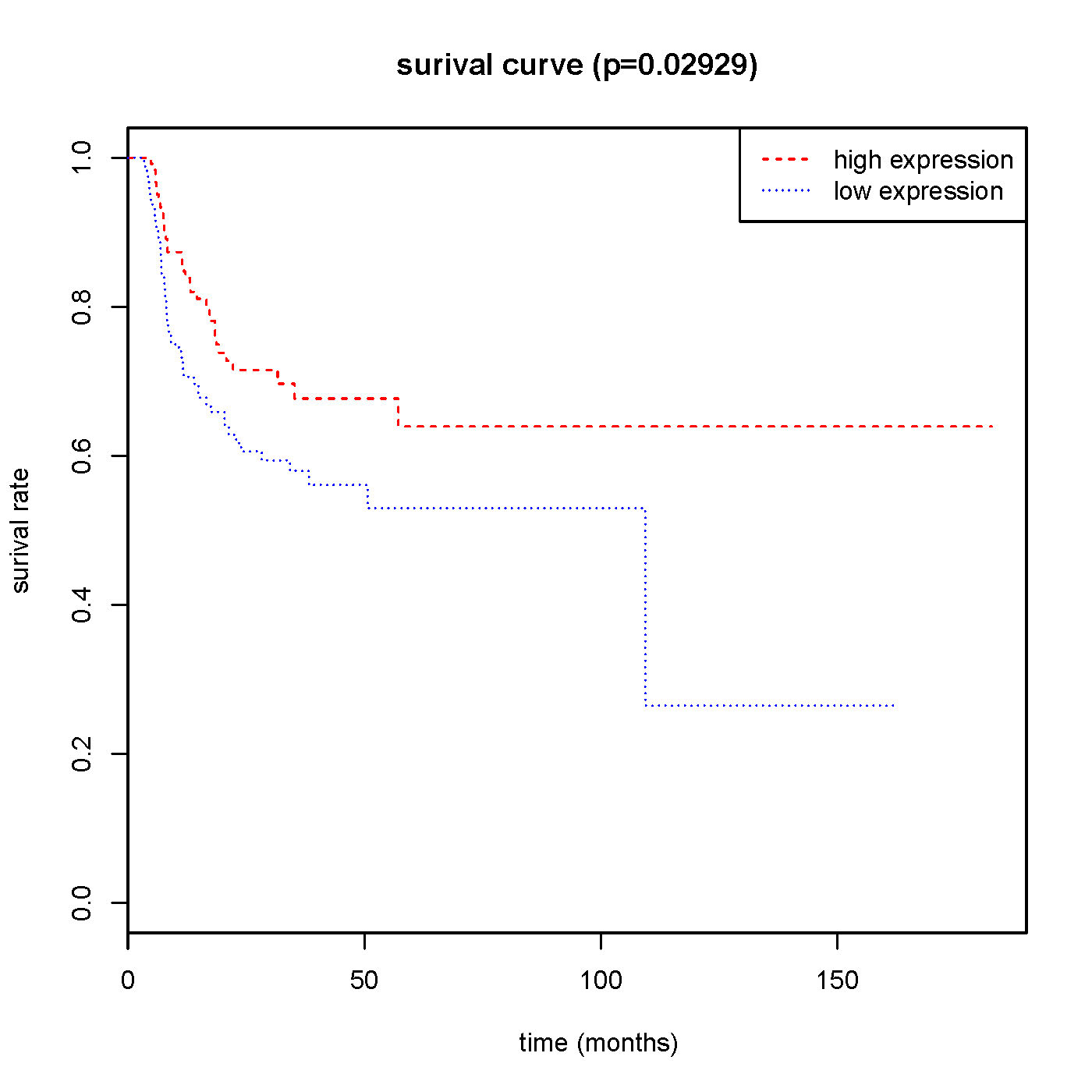

Supplement: Dataset S1 — Kaplan–Meier survival analysis with the log-rank test was used to identify relationships between the above 2493 lncRNA signatures and OSCC patient survival. Then, we determined the levels of 126 lncRNA signatures that were significantly related to DFS. [file peerj-06-5307-s005.zip › The result of Kaplan–Meier survival analyses and log-rank tests for DFS in OSCC/CTD-2545H1.2.jpg]

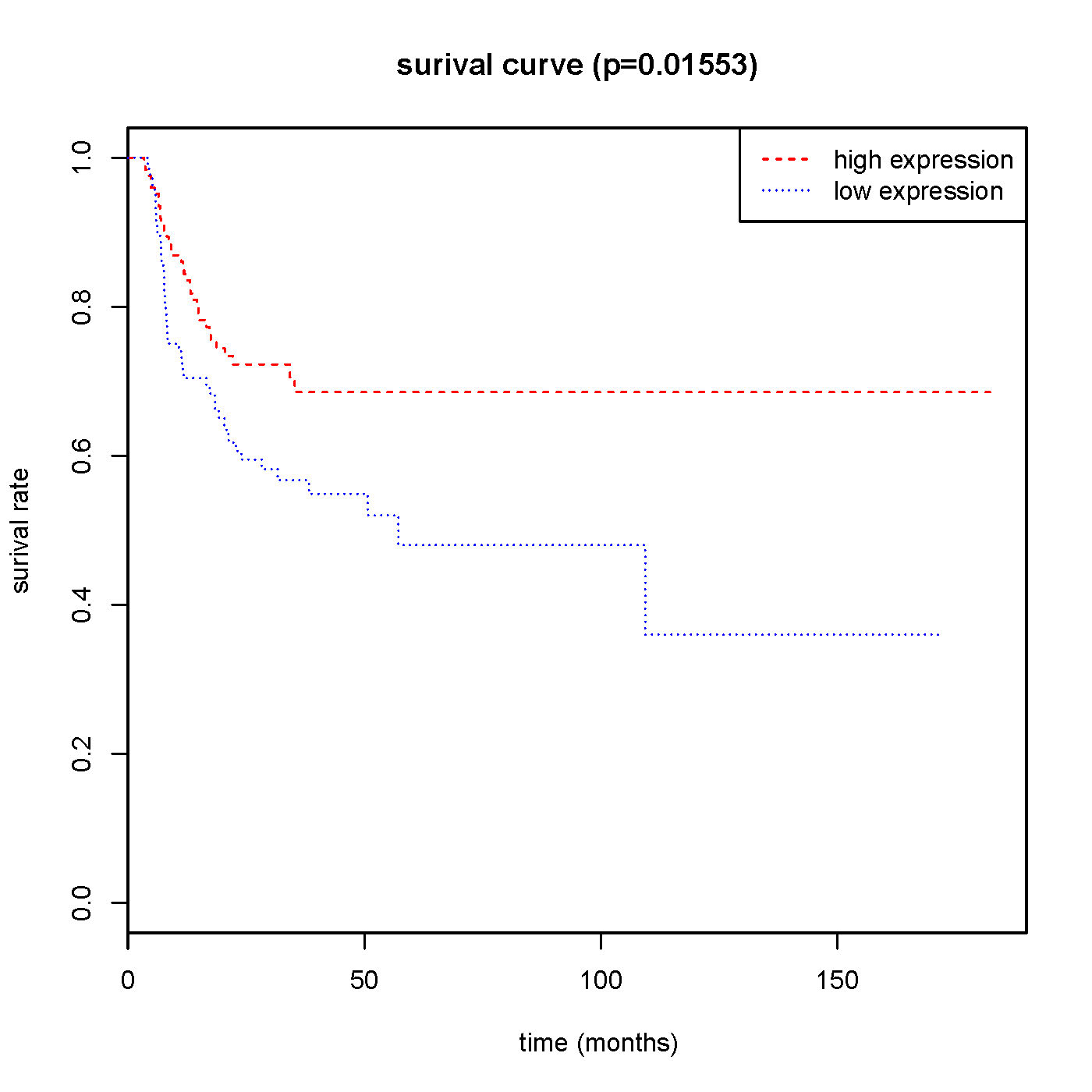

Supplement: Dataset S1 — Kaplan–Meier survival analysis with the log-rank test was used to identify relationships between the above 2493 lncRNA signatures and OSCC patient survival. Then, we determined the levels of 126 lncRNA signatures that were significantly related to DFS. [file peerj-06-5307-s005.zip › The result of Kaplan–Meier survival analyses and log-rank tests for DFS in OSCC/CTD-2619J13.17.jpg]

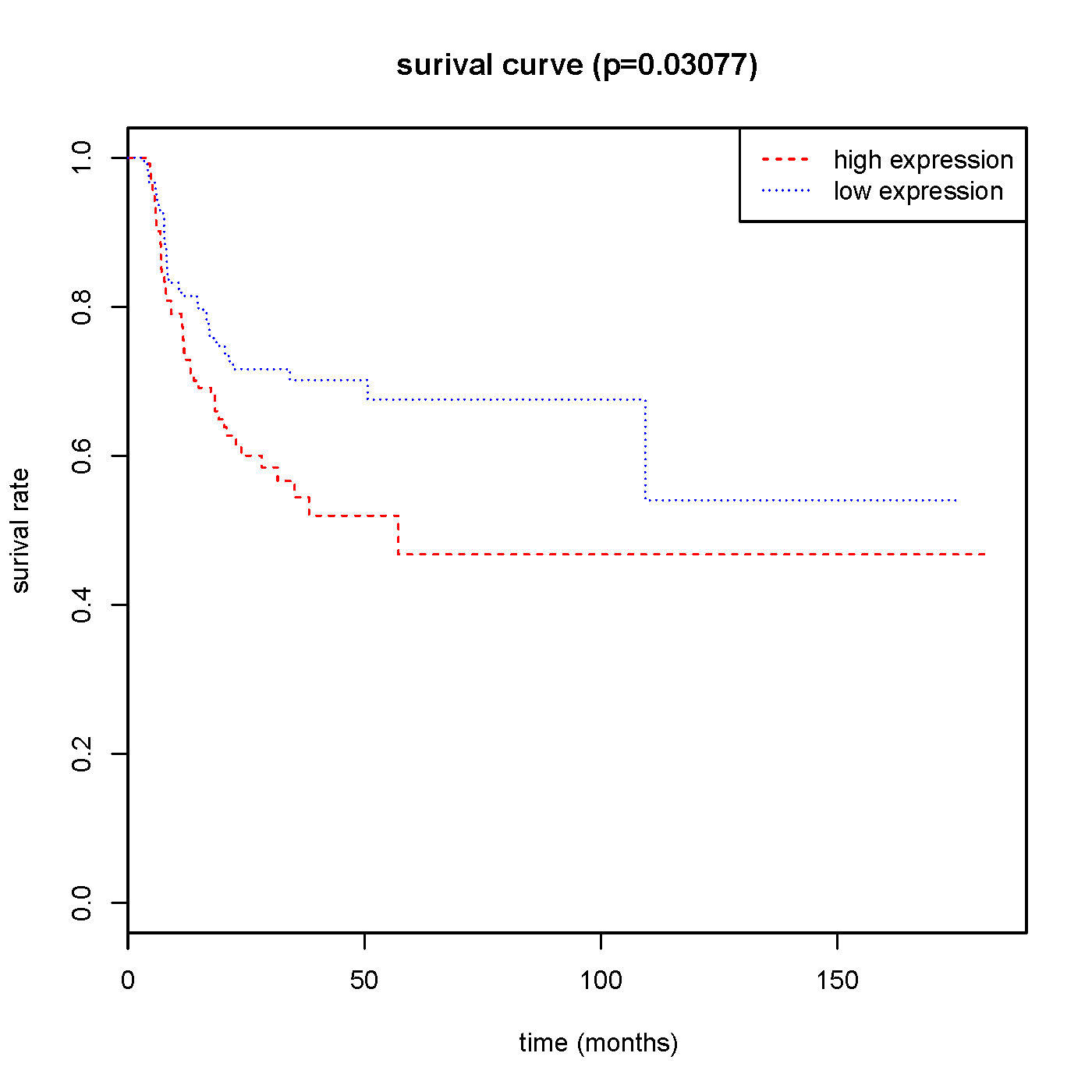

Supplement: Dataset S1 — Kaplan–Meier survival analysis with the log-rank test was used to identify relationships between the above 2493 lncRNA signatures and OSCC patient survival. Then, we determined the levels of 126 lncRNA signatures that were significantly related to DFS. [file peerj-06-5307-s005.zip › The result of Kaplan–Meier survival analyses and log-rank tests for DFS in OSCC/CTD-2649C14.2.jpg]

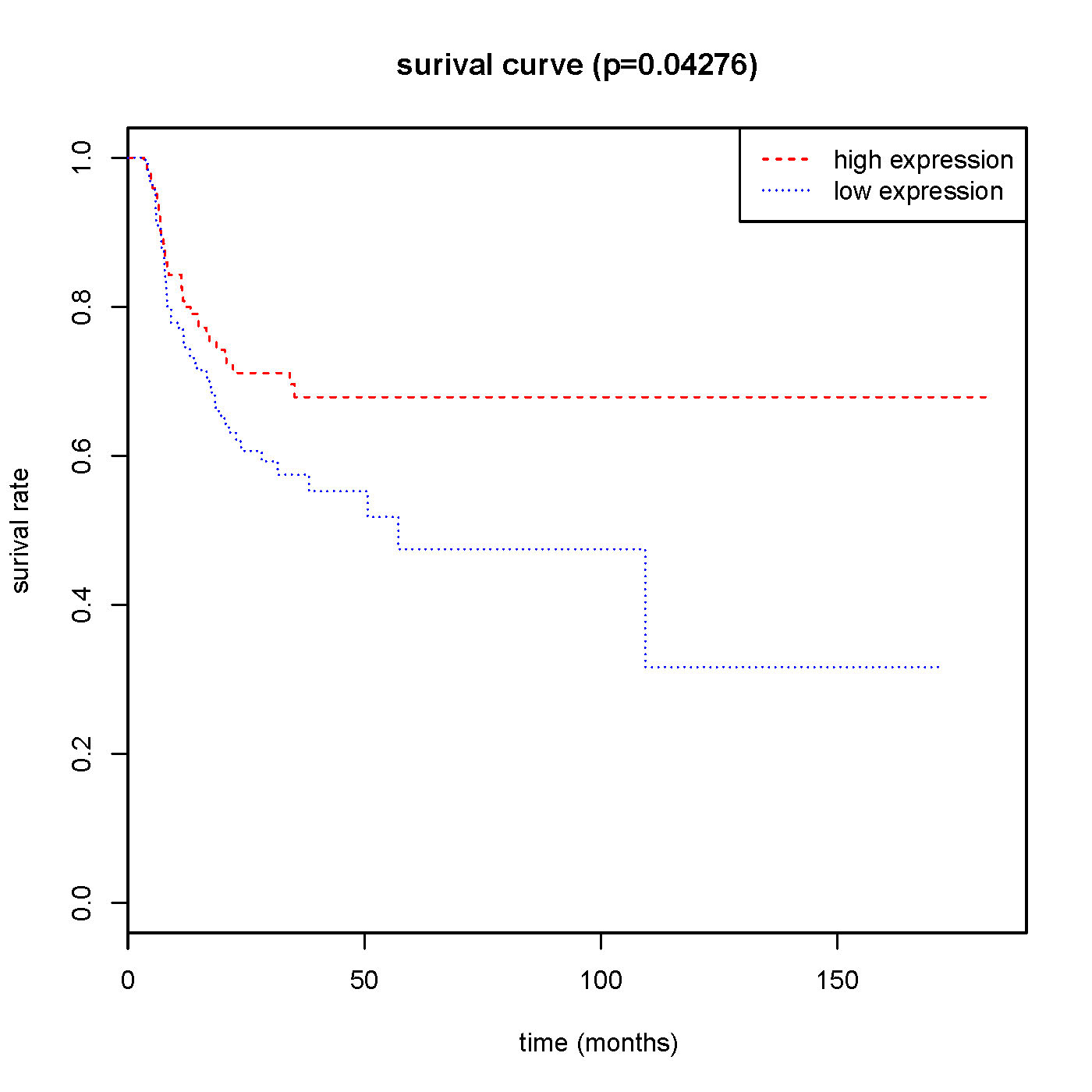

Supplement: Dataset S1 — Kaplan–Meier survival analysis with the log-rank test was used to identify relationships between the above 2493 lncRNA signatures and OSCC patient survival. Then, we determined the levels of 126 lncRNA signatures that were significantly related to DFS. [file peerj-06-5307-s005.zip › The result of Kaplan–Meier survival analyses and log-rank tests for DFS in OSCC/CTD-3157E16.1.jpg]

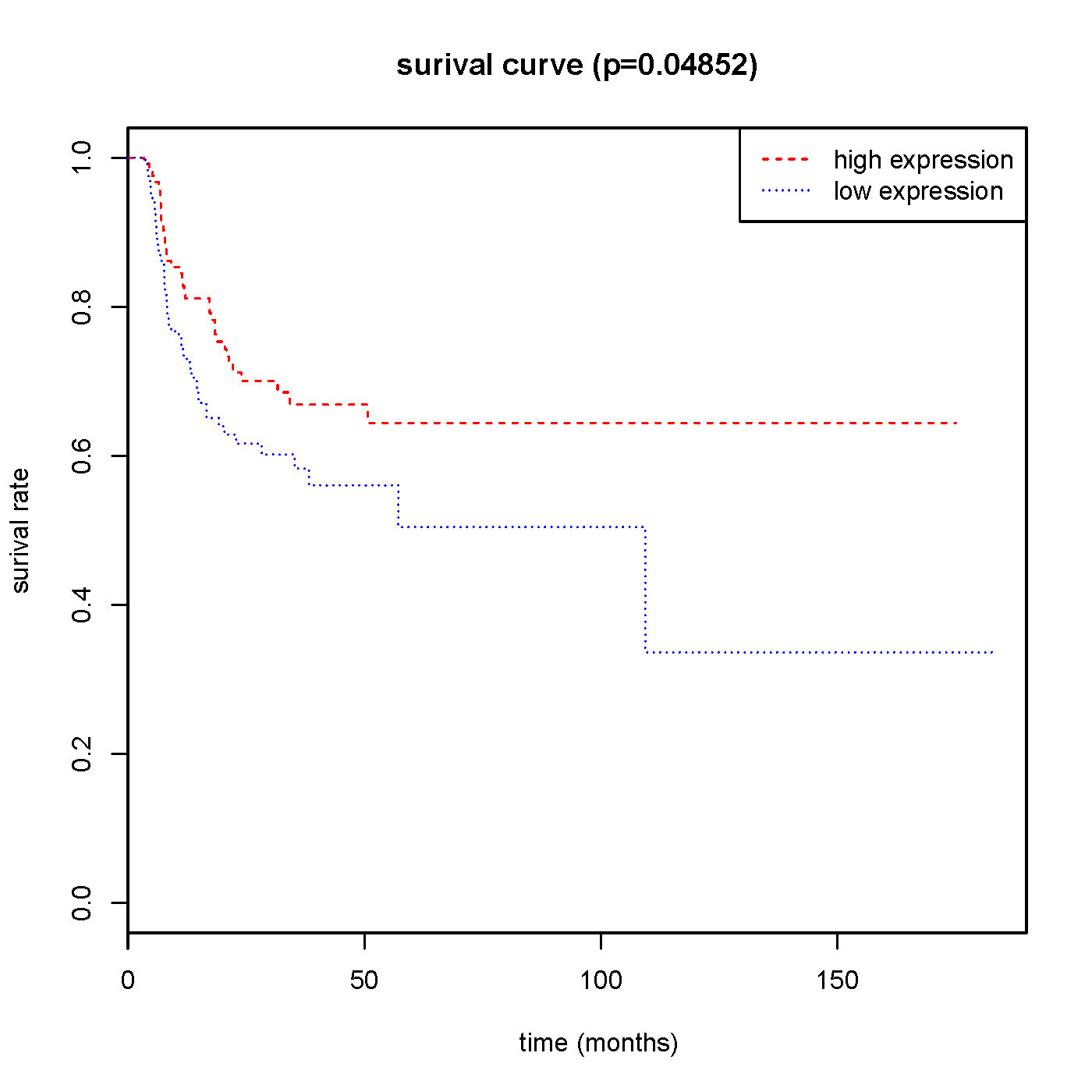

Supplement: Dataset S1 — Kaplan–Meier survival analysis with the log-rank test was used to identify relationships between the above 2493 lncRNA signatures and OSCC patient survival. Then, we determined the levels of 126 lncRNA signatures that were significantly related to DFS. [file peerj-06-5307-s005.zip › The result of Kaplan–Meier survival analyses and log-rank tests for DFS in OSCC/DISC1-IT1.jpg]

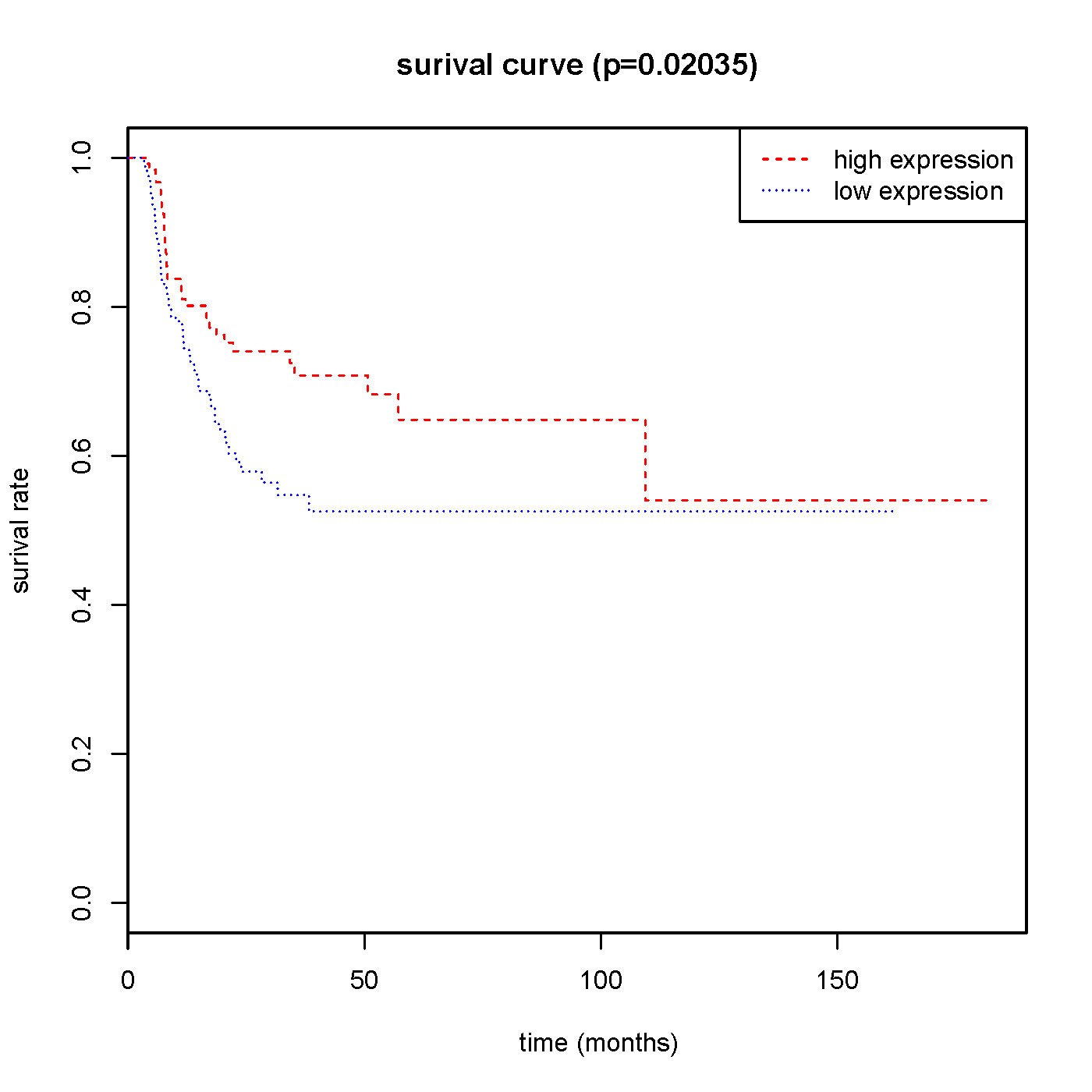

Supplement: Dataset S1 — Kaplan–Meier survival analysis with the log-rank test was used to identify relationships between the above 2493 lncRNA signatures and OSCC patient survival. Then, we determined the levels of 126 lncRNA signatures that were significantly related to DFS. [file peerj-06-5307-s005.zip › The result of Kaplan–Meier survival analyses and log-rank tests for DFS in OSCC/FAM230C.jpg]

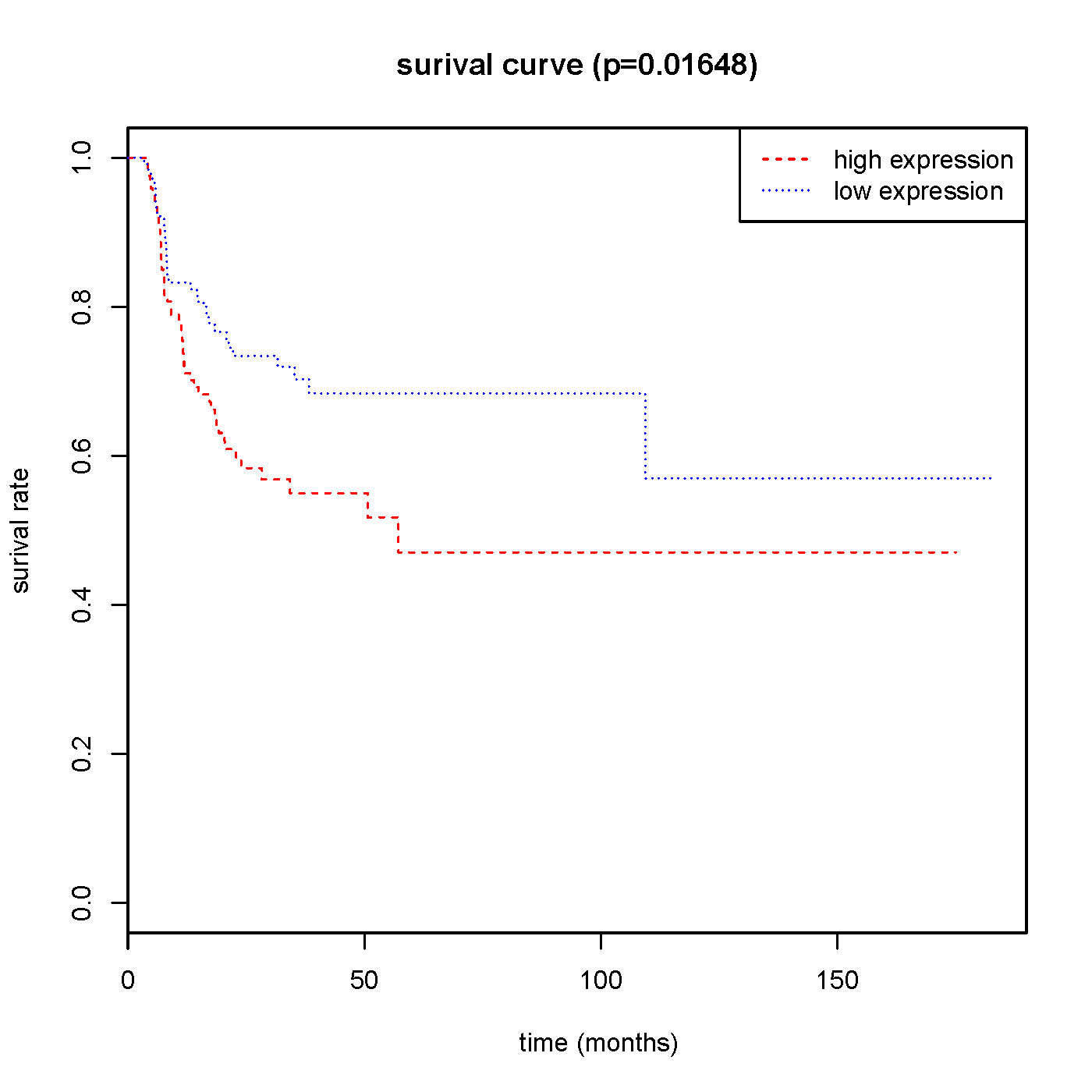

Supplement: Dataset S1 — Kaplan–Meier survival analysis with the log-rank test was used to identify relationships between the above 2493 lncRNA signatures and OSCC patient survival. Then, we determined the levels of 126 lncRNA signatures that were significantly related to DFS. [file peerj-06-5307-s005.zip › The result of Kaplan–Meier survival analyses and log-rank tests for DFS in OSCC/FAM95B1.jpg]

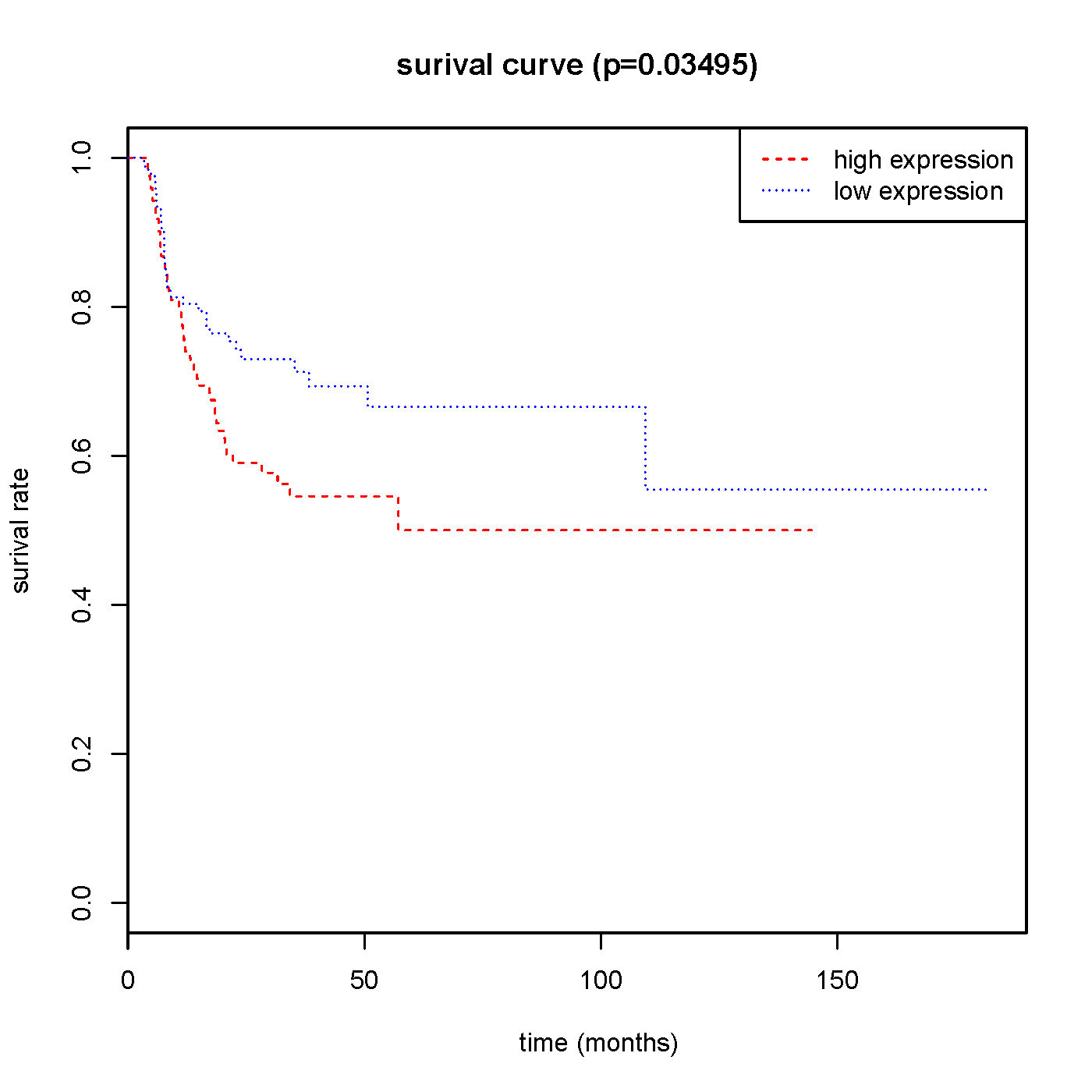

Supplement: Dataset S1 — Kaplan–Meier survival analysis with the log-rank test was used to identify relationships between the above 2493 lncRNA signatures and OSCC patient survival. Then, we determined the levels of 126 lncRNA signatures that were significantly related to DFS. [file peerj-06-5307-s005.zip › The result of Kaplan–Meier survival analyses and log-rank tests for DFS in OSCC/FNDC1-IT1.jpg]

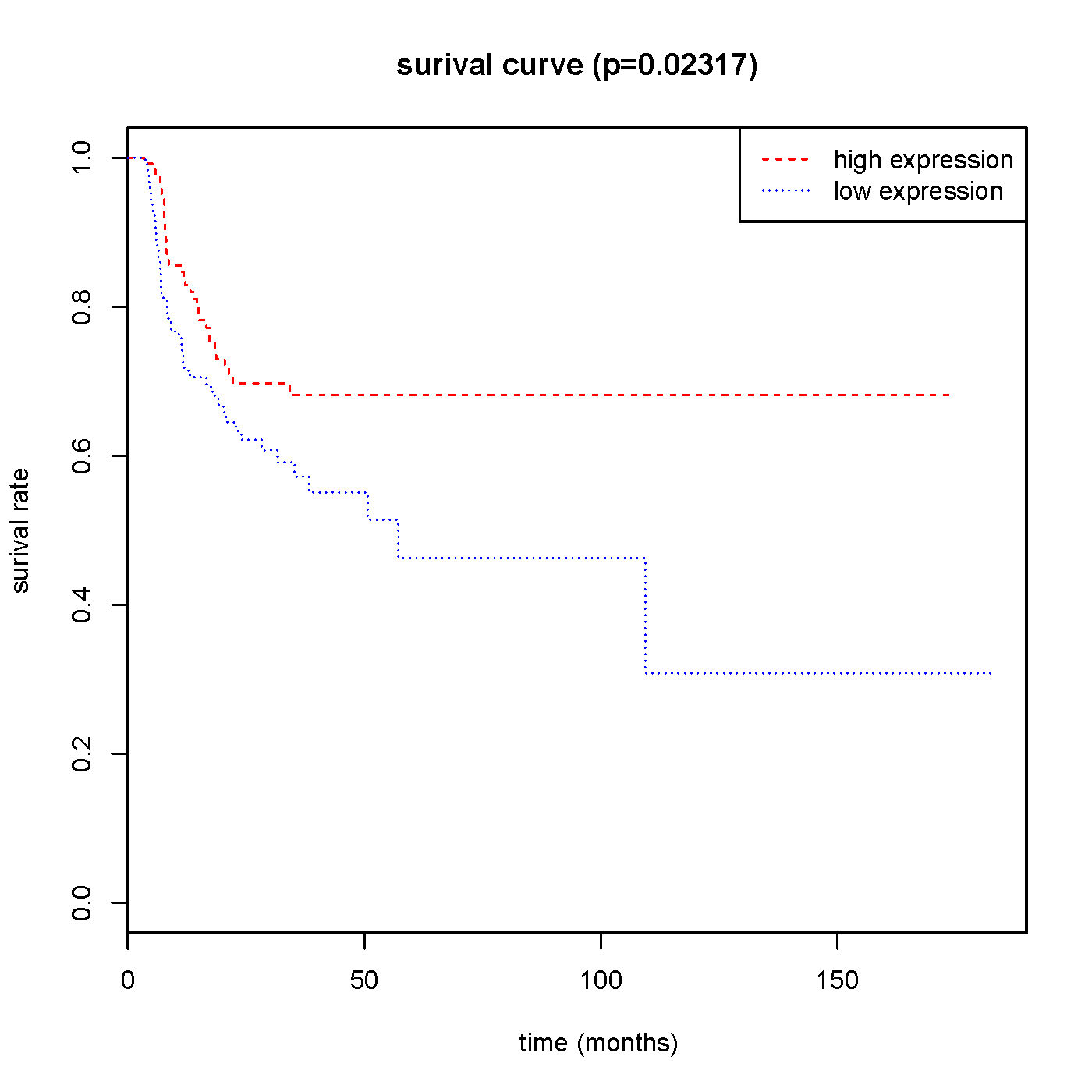

Supplement: Dataset S1 — Kaplan–Meier survival analysis with the log-rank test was used to identify relationships between the above 2493 lncRNA signatures and OSCC patient survival. Then, we determined the levels of 126 lncRNA signatures that were significantly related to DFS. [file peerj-06-5307-s005.zip › The result of Kaplan–Meier survival analyses and log-rank tests for DFS in OSCC/HS1BP3-IT1.jpg]

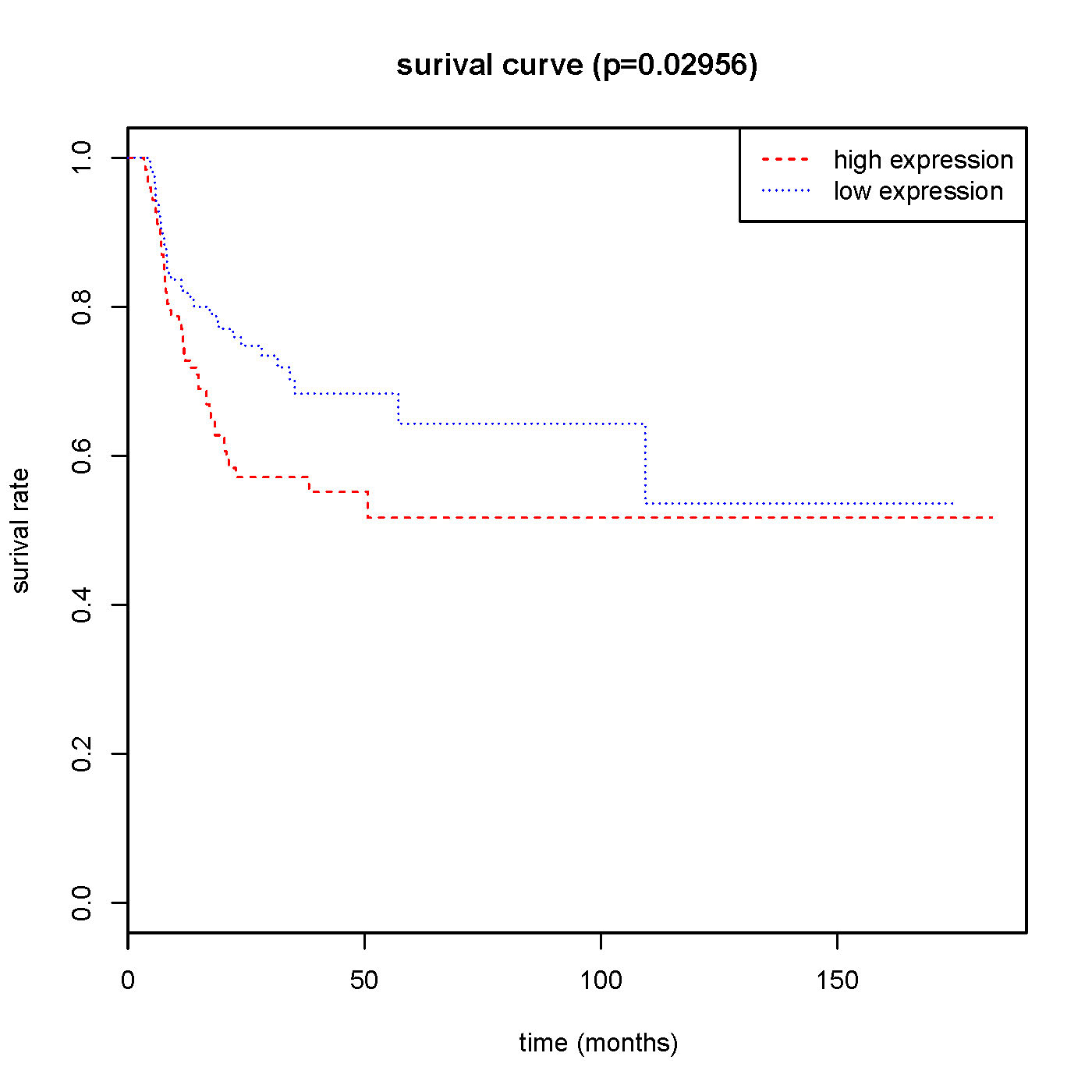

Supplement: Dataset S1 — Kaplan–Meier survival analysis with the log-rank test was used to identify relationships between the above 2493 lncRNA signatures and OSCC patient survival. Then, we determined the levels of 126 lncRNA signatures that were significantly related to DFS. [file peerj-06-5307-s005.zip › The result of Kaplan–Meier survival analyses and log-rank tests for DFS in OSCC/KB-431C1.5.jpg]

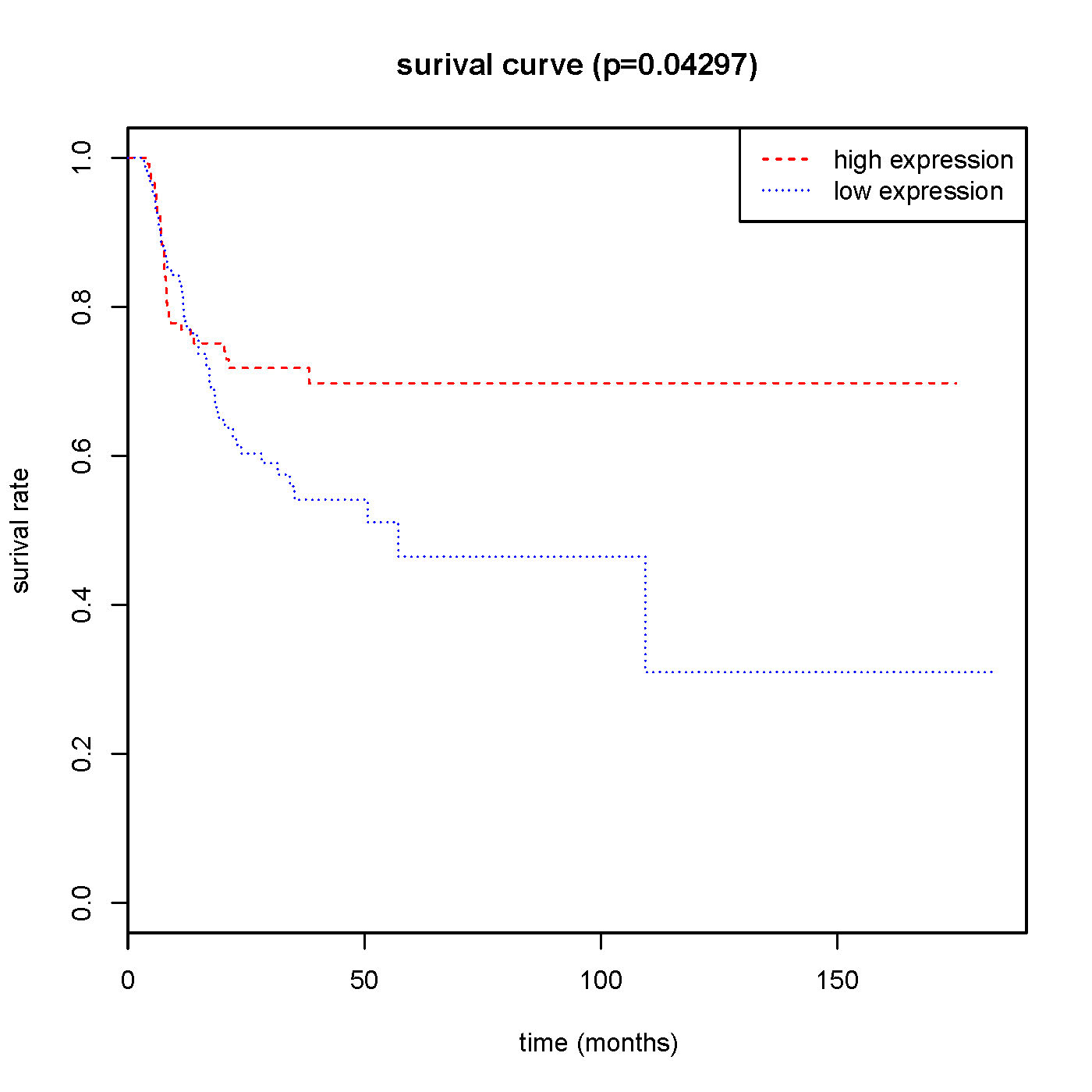

Supplement: Dataset S1 — Kaplan–Meier survival analysis with the log-rank test was used to identify relationships between the above 2493 lncRNA signatures and OSCC patient survival. Then, we determined the levels of 126 lncRNA signatures that were significantly related to DFS. [file peerj-06-5307-s005.zip › The result of Kaplan–Meier survival analyses and log-rank tests for DFS in OSCC/KIRREL-IT1.jpg]

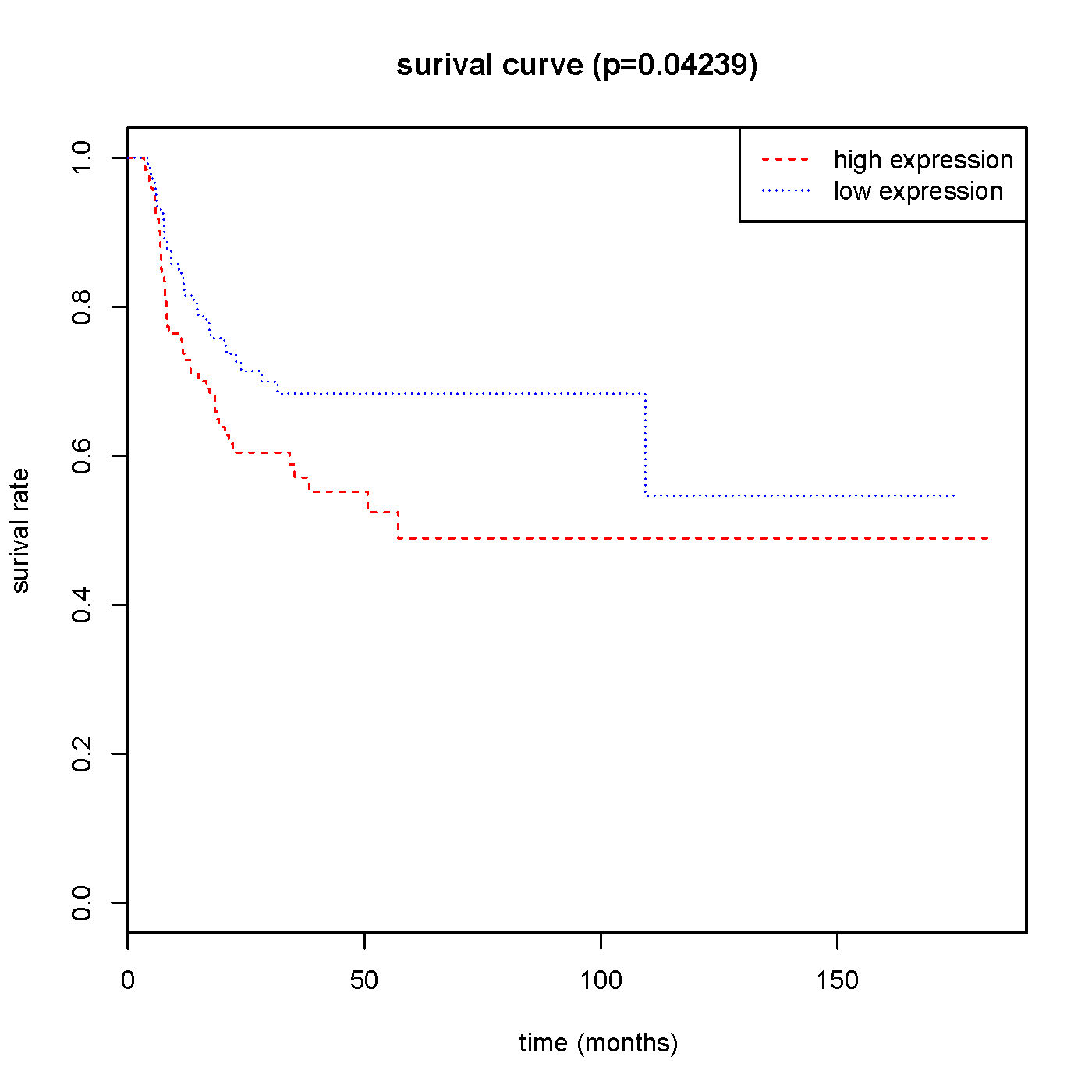

Supplement: Dataset S1 — Kaplan–Meier survival analysis with the log-rank test was used to identify relationships between the above 2493 lncRNA signatures and OSCC patient survival. Then, we determined the levels of 126 lncRNA signatures that were significantly related to DFS. [file peerj-06-5307-s005.zip › The result of Kaplan–Meier survival analyses and log-rank tests for DFS in OSCC/LINC00052.jpg]

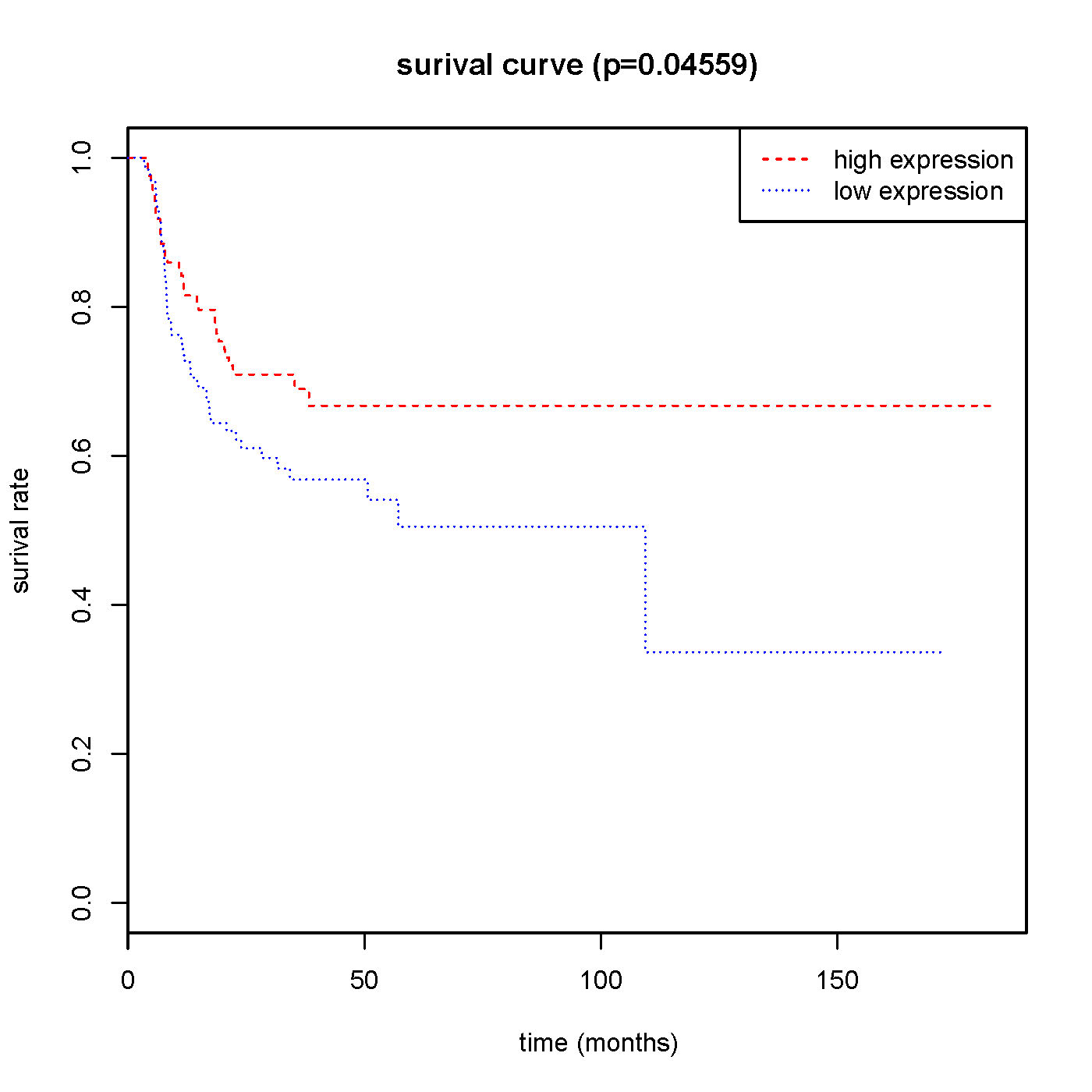

Supplement: Dataset S1 — Kaplan–Meier survival analysis with the log-rank test was used to identify relationships between the above 2493 lncRNA signatures and OSCC patient survival. Then, we determined the levels of 126 lncRNA signatures that were significantly related to DFS. [file peerj-06-5307-s005.zip › The result of Kaplan–Meier survival analyses and log-rank tests for DFS in OSCC/LINC00174.jpg]

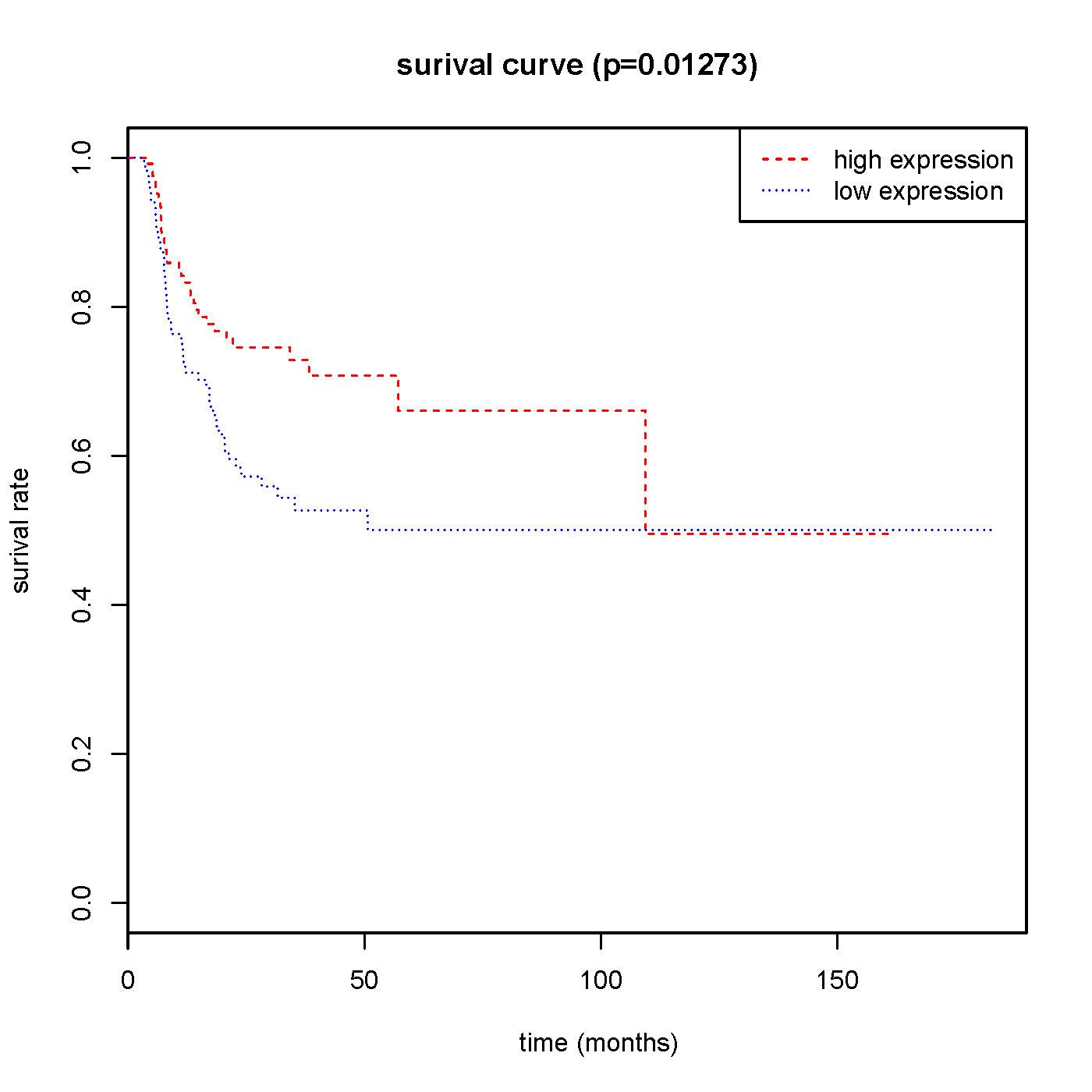

Supplement: Dataset S1 — Kaplan–Meier survival analysis with the log-rank test was used to identify relationships between the above 2493 lncRNA signatures and OSCC patient survival. Then, we determined the levels of 126 lncRNA signatures that were significantly related to DFS. [file peerj-06-5307-s005.zip › The result of Kaplan–Meier survival analyses and log-rank tests for DFS in OSCC/LINC00184.jpg]

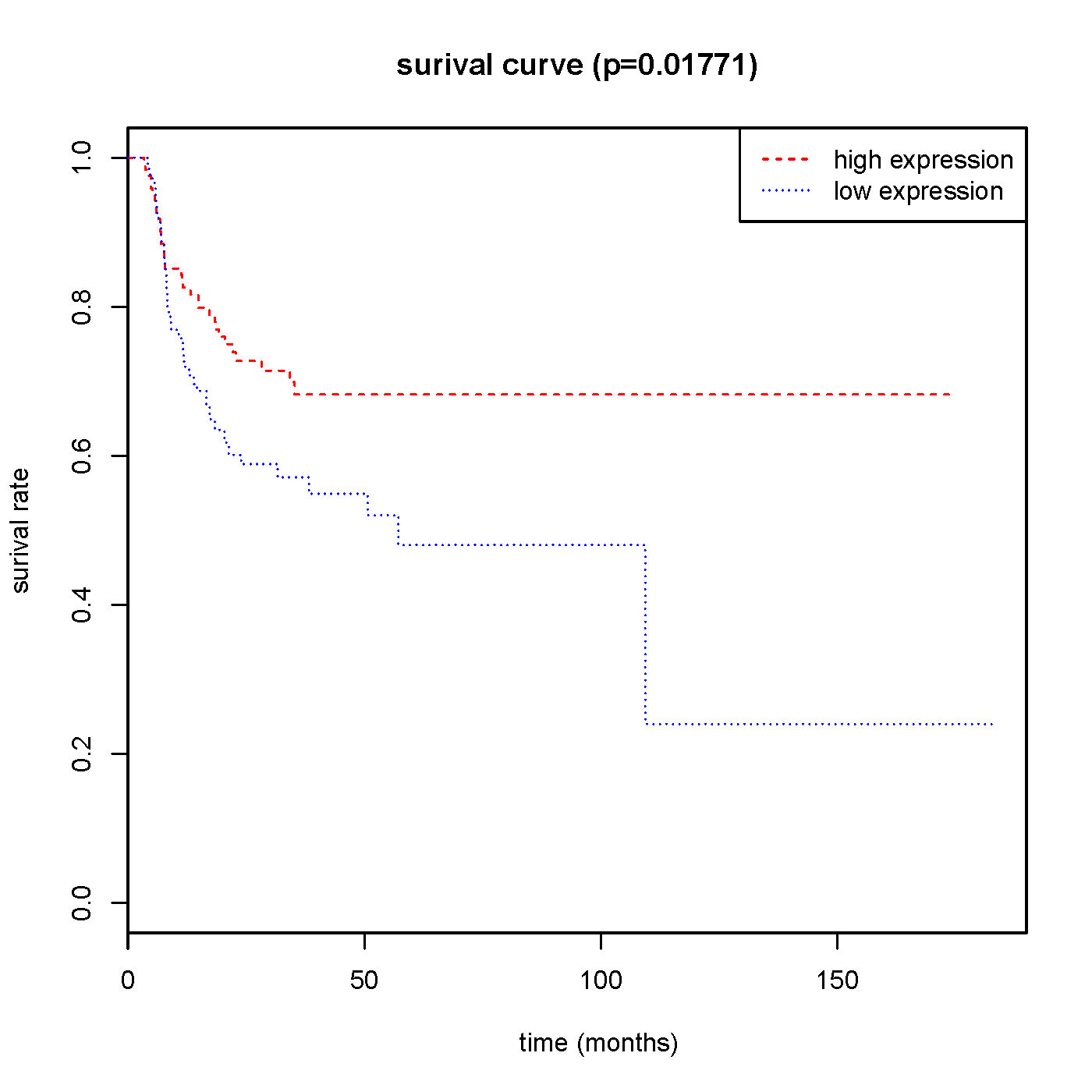

Supplement: Dataset S1 — Kaplan–Meier survival analysis with the log-rank test was used to identify relationships between the above 2493 lncRNA signatures and OSCC patient survival. Then, we determined the levels of 126 lncRNA signatures that were significantly related to DFS. [file peerj-06-5307-s005.zip › The result of Kaplan–Meier survival analyses and log-rank tests for DFS in OSCC/LINC00327.jpg]

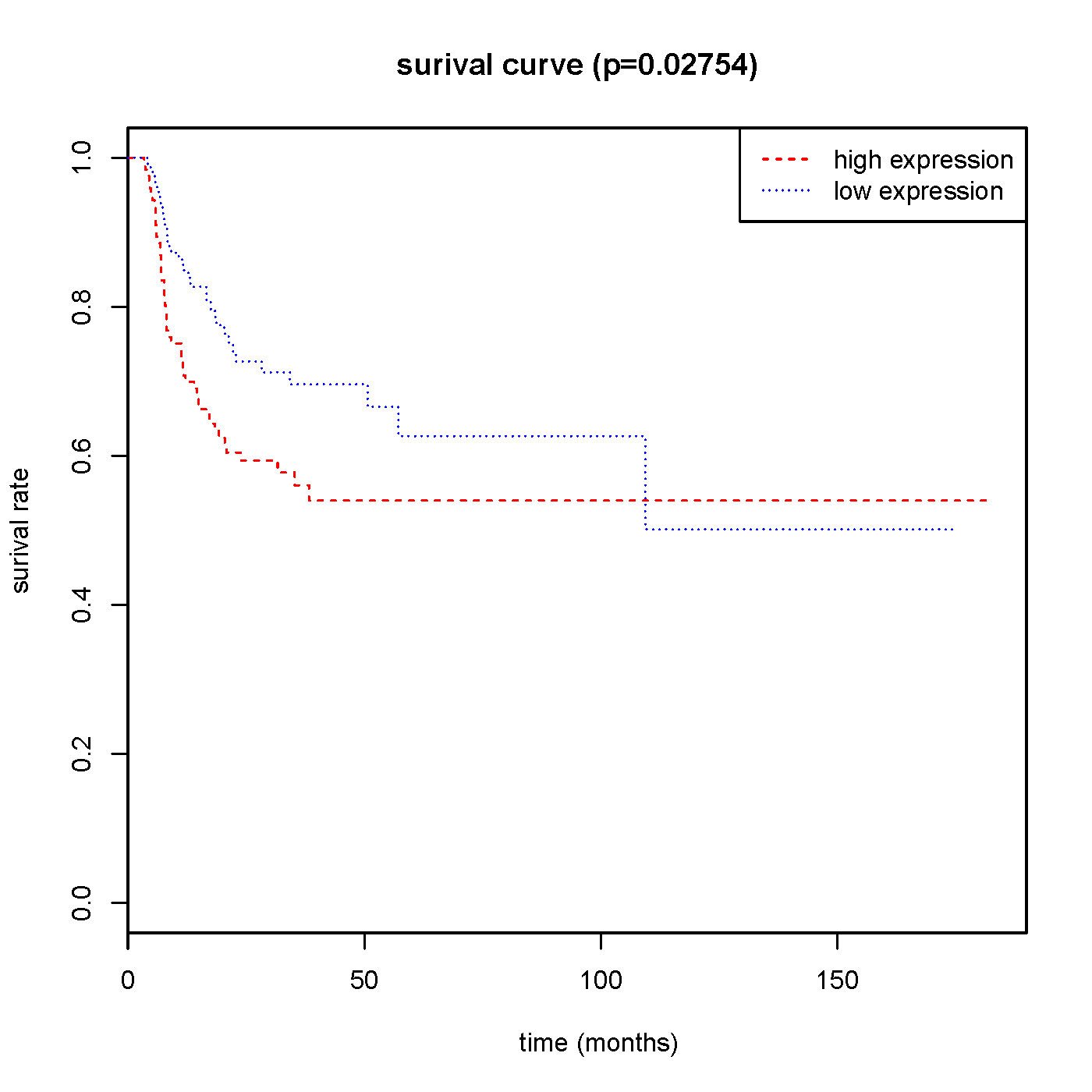

Supplement: Dataset S1 — Kaplan–Meier survival analysis with the log-rank test was used to identify relationships between the above 2493 lncRNA signatures and OSCC patient survival. Then, we determined the levels of 126 lncRNA signatures that were significantly related to DFS. [file peerj-06-5307-s005.zip › The result of Kaplan–Meier survival analyses and log-rank tests for DFS in OSCC/LINC00705.jpg]

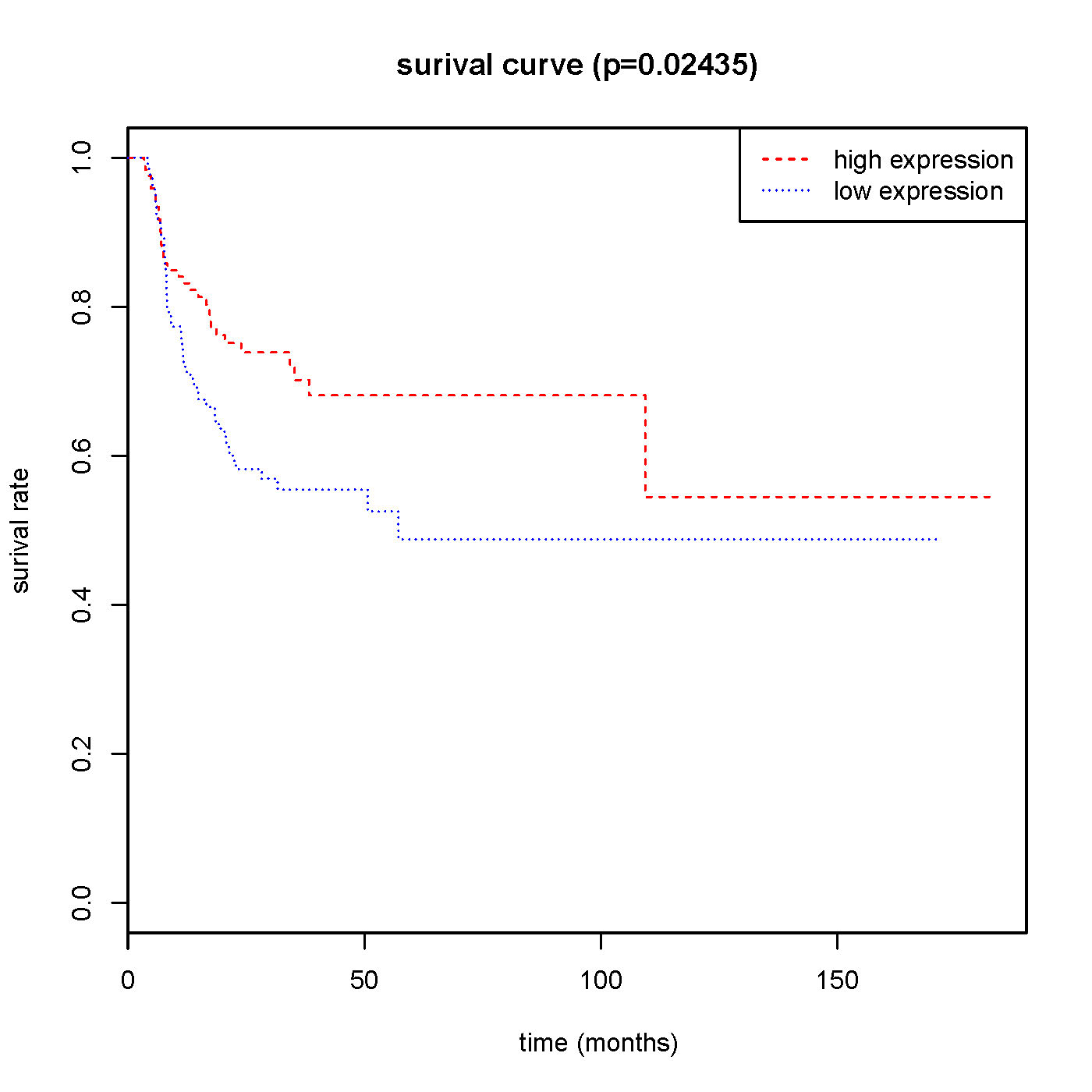

Supplement: Dataset S1 — Kaplan–Meier survival analysis with the log-rank test was used to identify relationships between the above 2493 lncRNA signatures and OSCC patient survival. Then, we determined the levels of 126 lncRNA signatures that were significantly related to DFS. [file peerj-06-5307-s005.zip › The result of Kaplan–Meier survival analyses and log-rank tests for DFS in OSCC/LINC00996.jpg]

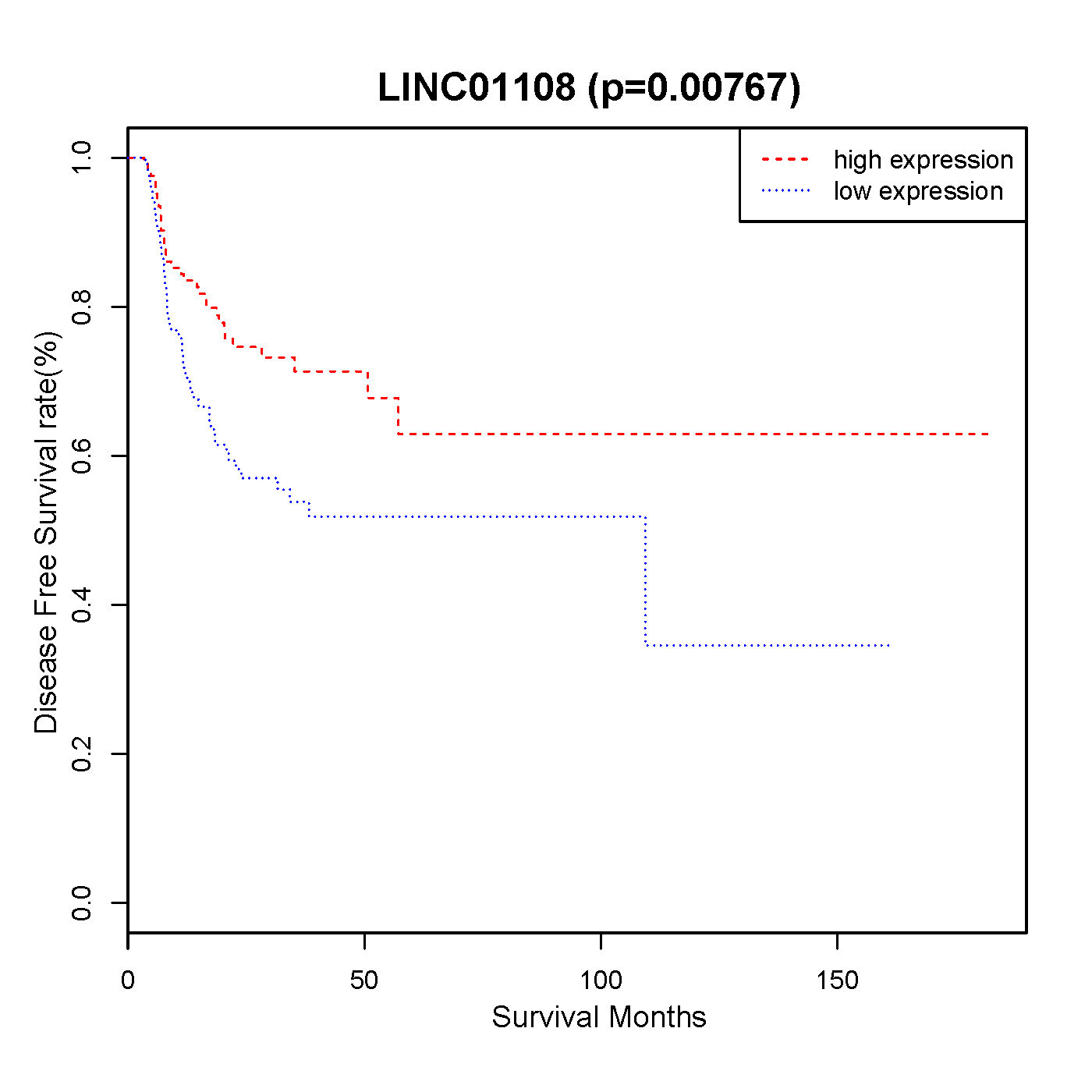

Supplement: Dataset S1 — Kaplan–Meier survival analysis with the log-rank test was used to identify relationships between the above 2493 lncRNA signatures and OSCC patient survival. Then, we determined the levels of 126 lncRNA signatures that were significantly related to DFS. [file peerj-06-5307-s005.zip › The result of Kaplan–Meier survival analyses and log-rank tests for DFS in OSCC/LINC01108.jpg]

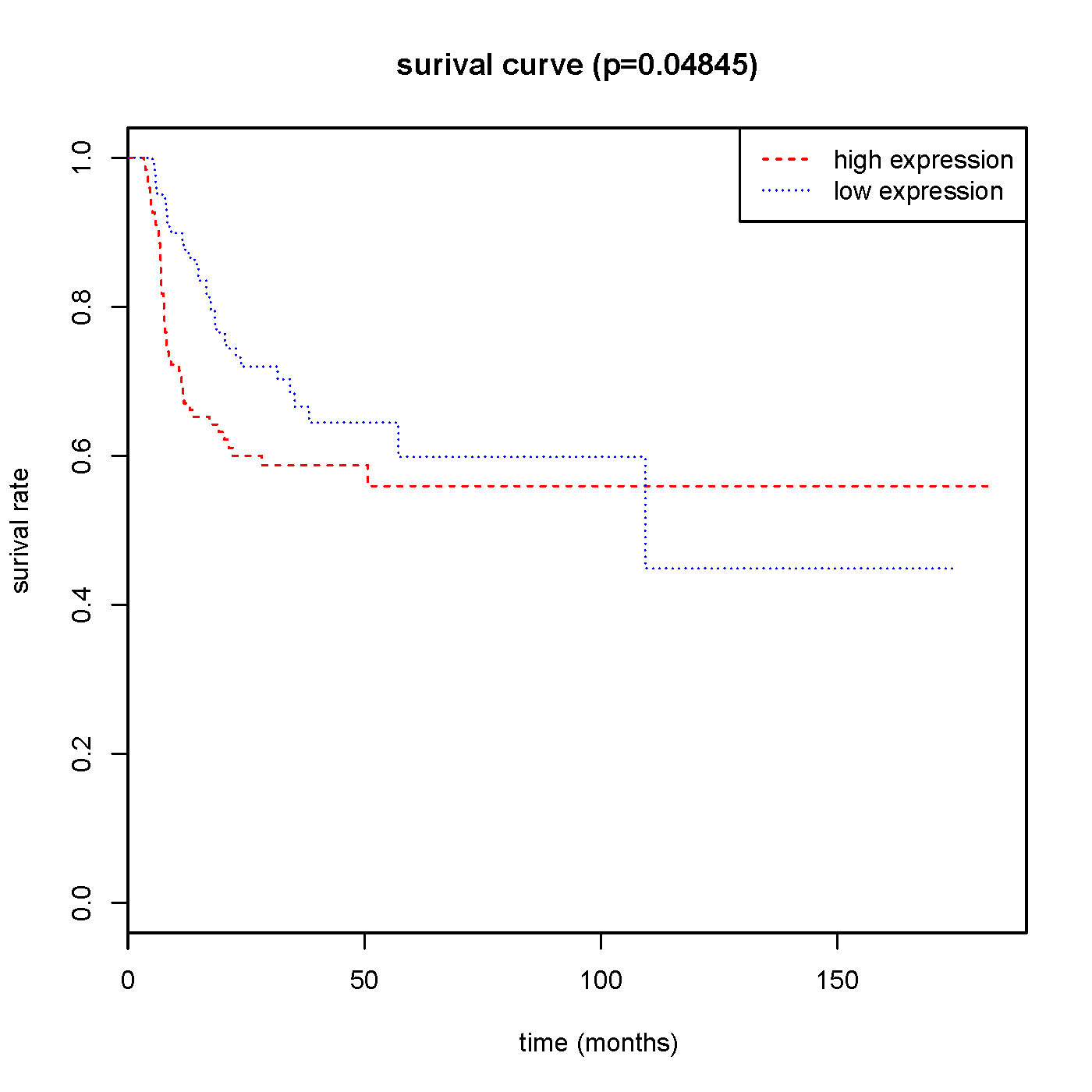

Supplement: Dataset S1 — Kaplan–Meier survival analysis with the log-rank test was used to identify relationships between the above 2493 lncRNA signatures and OSCC patient survival. Then, we determined the levels of 126 lncRNA signatures that were significantly related to DFS. [file peerj-06-5307-s005.zip › The result of Kaplan–Meier survival analyses and log-rank tests for DFS in OSCC/LINC01170.jpg]

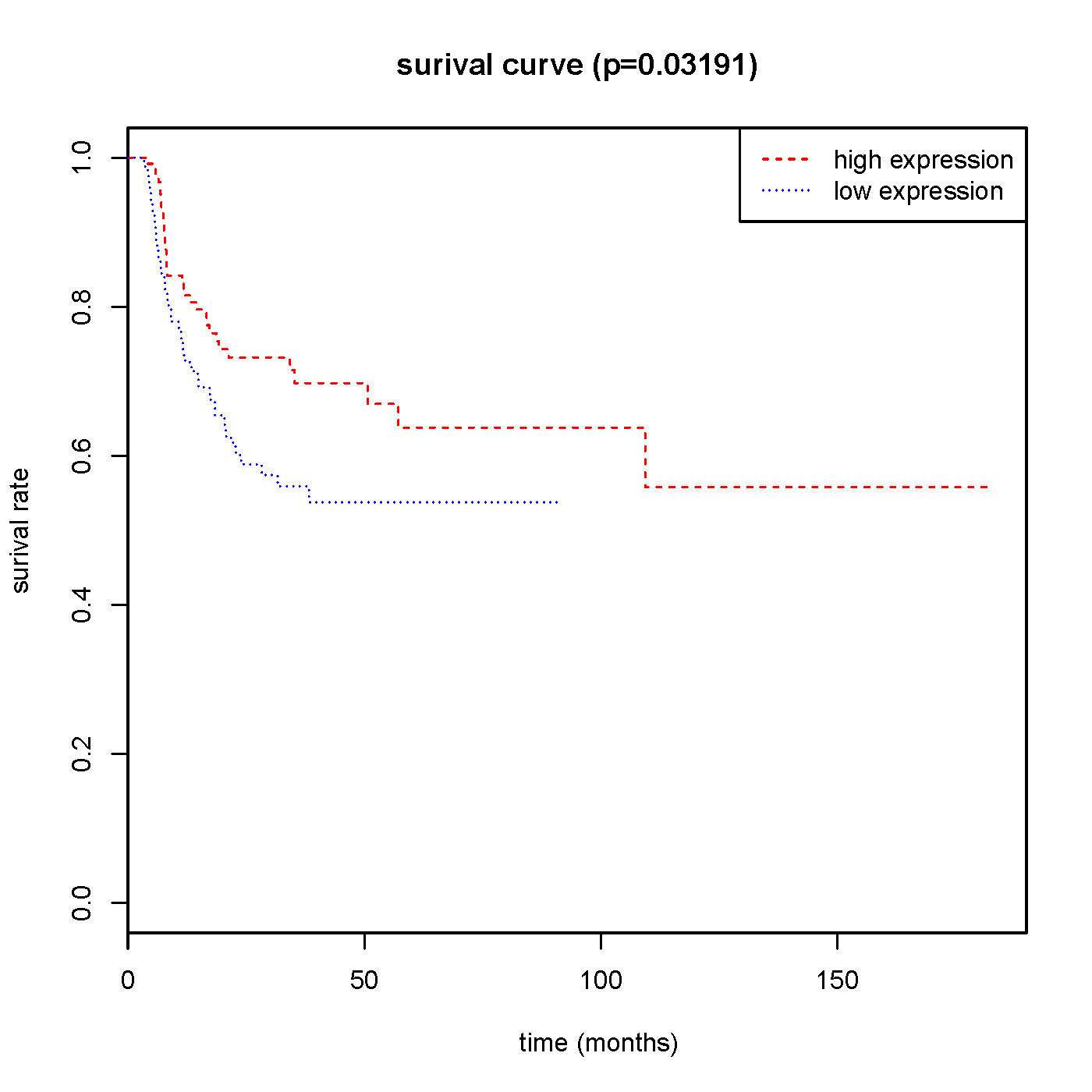

Supplement: Dataset S1 — Kaplan–Meier survival analysis with the log-rank test was used to identify relationships between the above 2493 lncRNA signatures and OSCC patient survival. Then, we determined the levels of 126 lncRNA signatures that were significantly related to DFS. [file peerj-06-5307-s005.zip › The result of Kaplan–Meier survival analyses and log-rank tests for DFS in OSCC/LINC01343.jpg]

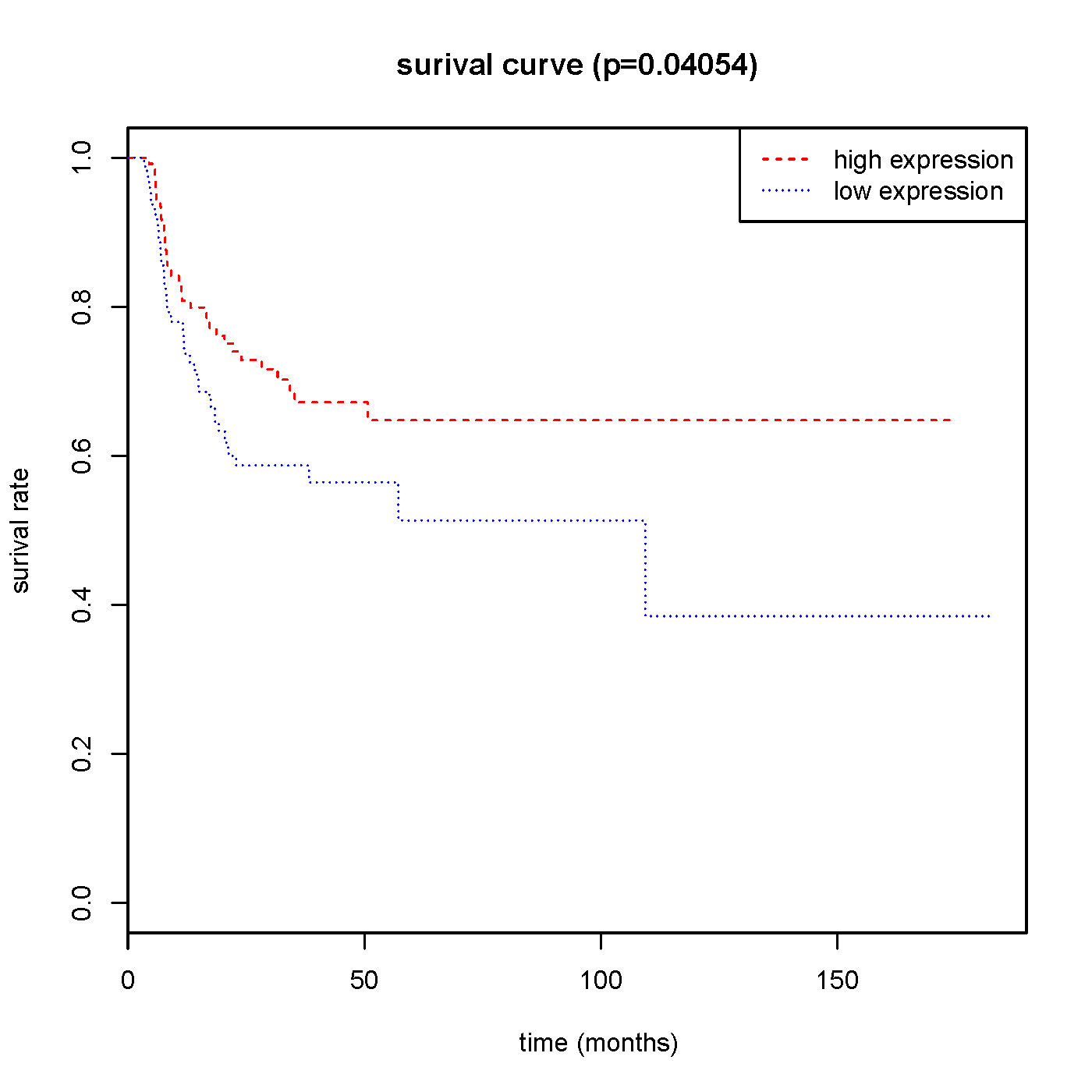

Supplement: Dataset S1 — Kaplan–Meier survival analysis with the log-rank test was used to identify relationships between the above 2493 lncRNA signatures and OSCC patient survival. Then, we determined the levels of 126 lncRNA signatures that were significantly related to DFS. [file peerj-06-5307-s005.zip › The result of Kaplan–Meier survival analyses and log-rank tests for DFS in OSCC/LINC01456.jpg]

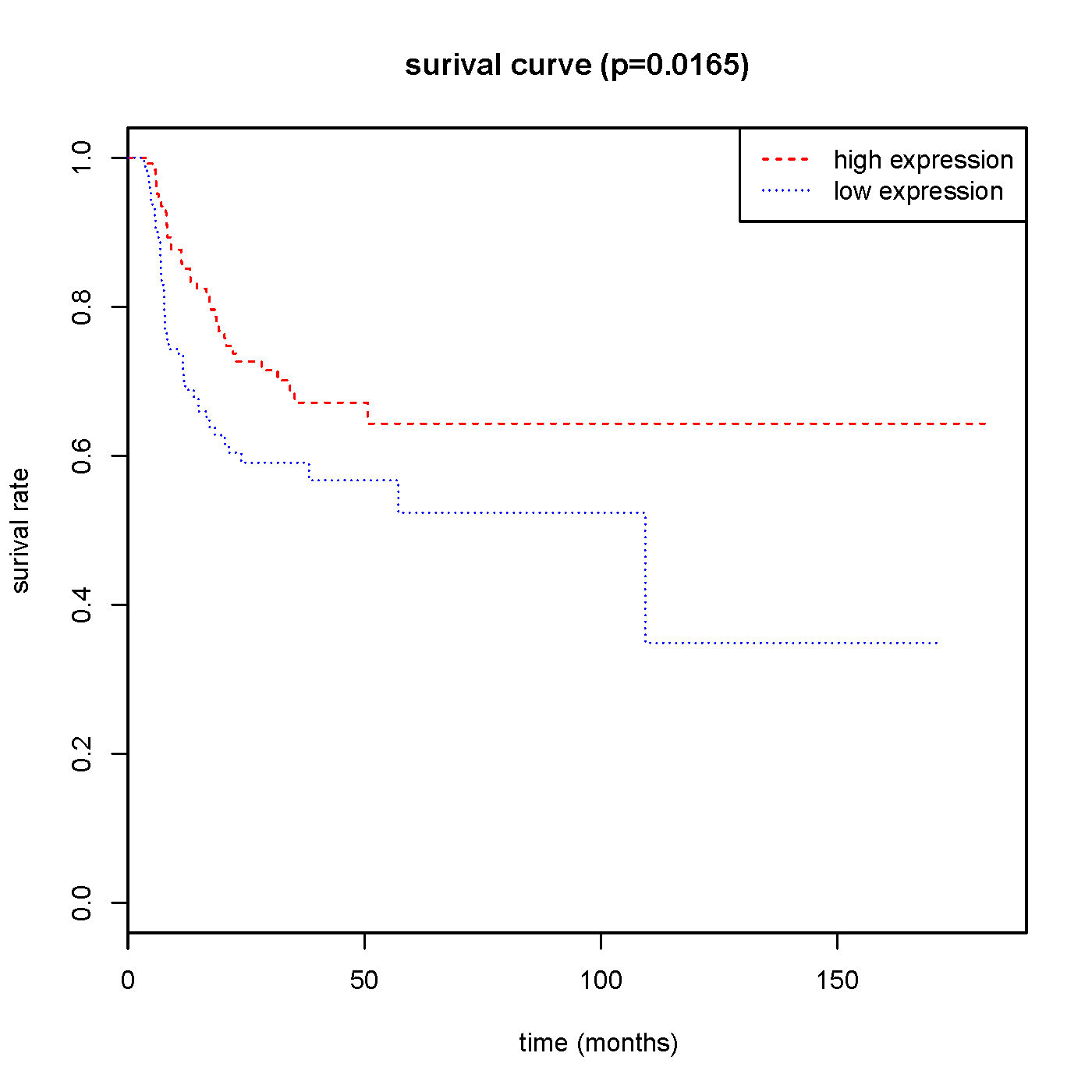

Supplement: Dataset S1 — Kaplan–Meier survival analysis with the log-rank test was used to identify relationships between the above 2493 lncRNA signatures and OSCC patient survival. Then, we determined the levels of 126 lncRNA signatures that were significantly related to DFS. [file peerj-06-5307-s005.zip › The result of Kaplan–Meier survival analyses and log-rank tests for DFS in OSCC/LINC01549.jpg]

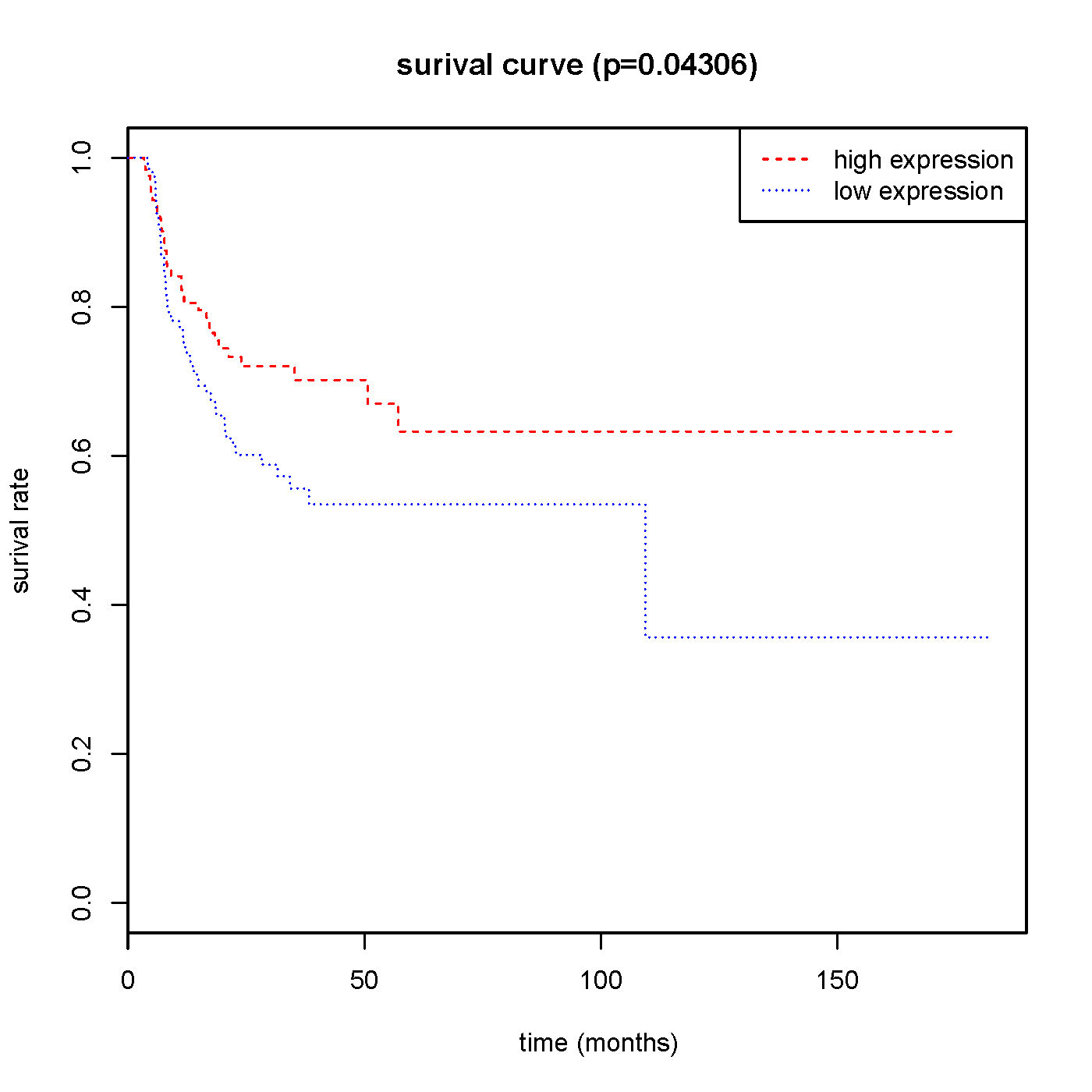

Supplement: Dataset S1 — Kaplan–Meier survival analysis with the log-rank test was used to identify relationships between the above 2493 lncRNA signatures and OSCC patient survival. Then, we determined the levels of 126 lncRNA signatures that were significantly related to DFS. [file peerj-06-5307-s005.zip › The result of Kaplan–Meier survival analyses and log-rank tests for DFS in OSCC/LINC01886.jpg]

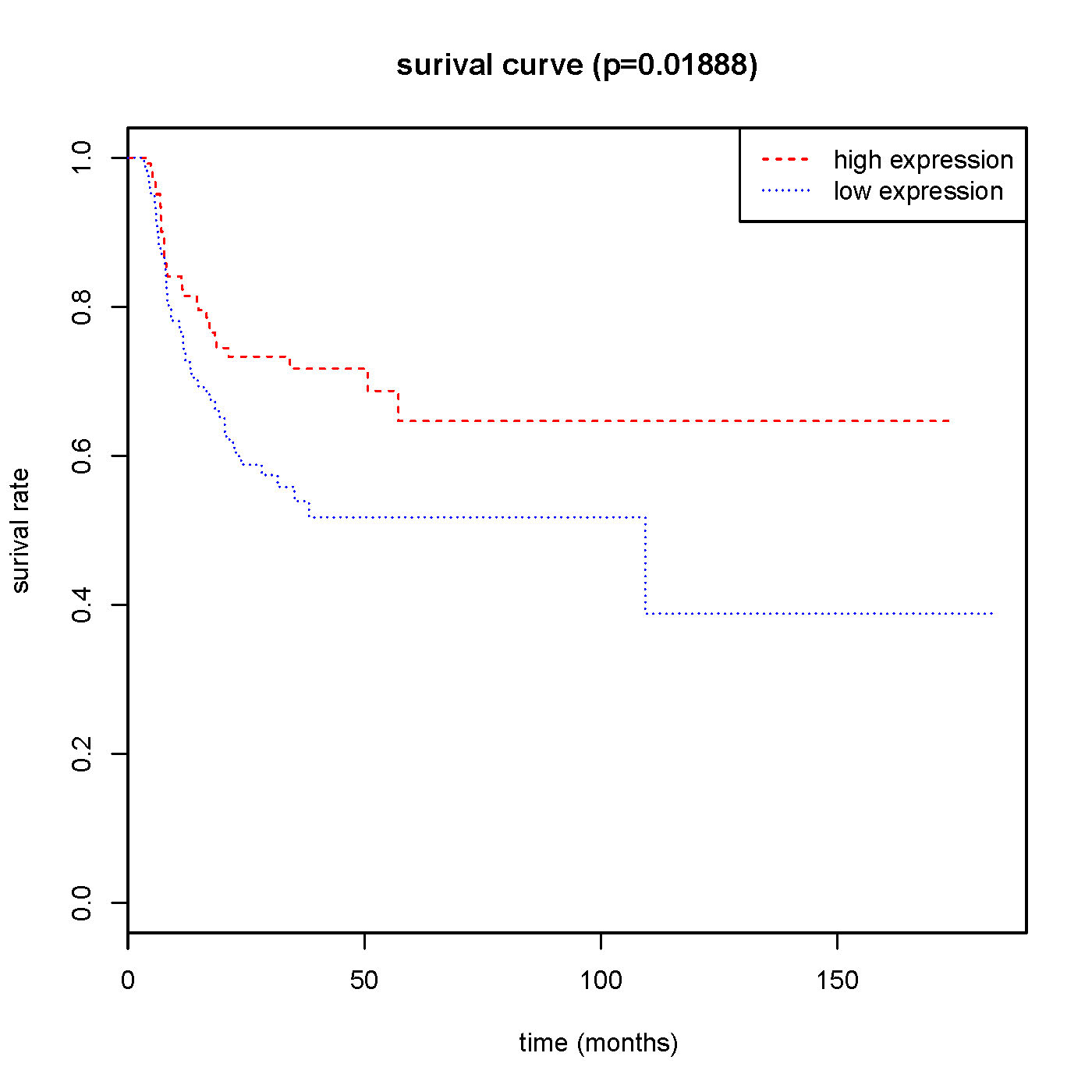

Supplement: Dataset S1 — Kaplan–Meier survival analysis with the log-rank test was used to identify relationships between the above 2493 lncRNA signatures and OSCC patient survival. Then, we determined the levels of 126 lncRNA signatures that were significantly related to DFS. [file peerj-06-5307-s005.zip › The result of Kaplan–Meier survival analyses and log-rank tests for DFS in OSCC/LINC01925.jpg]

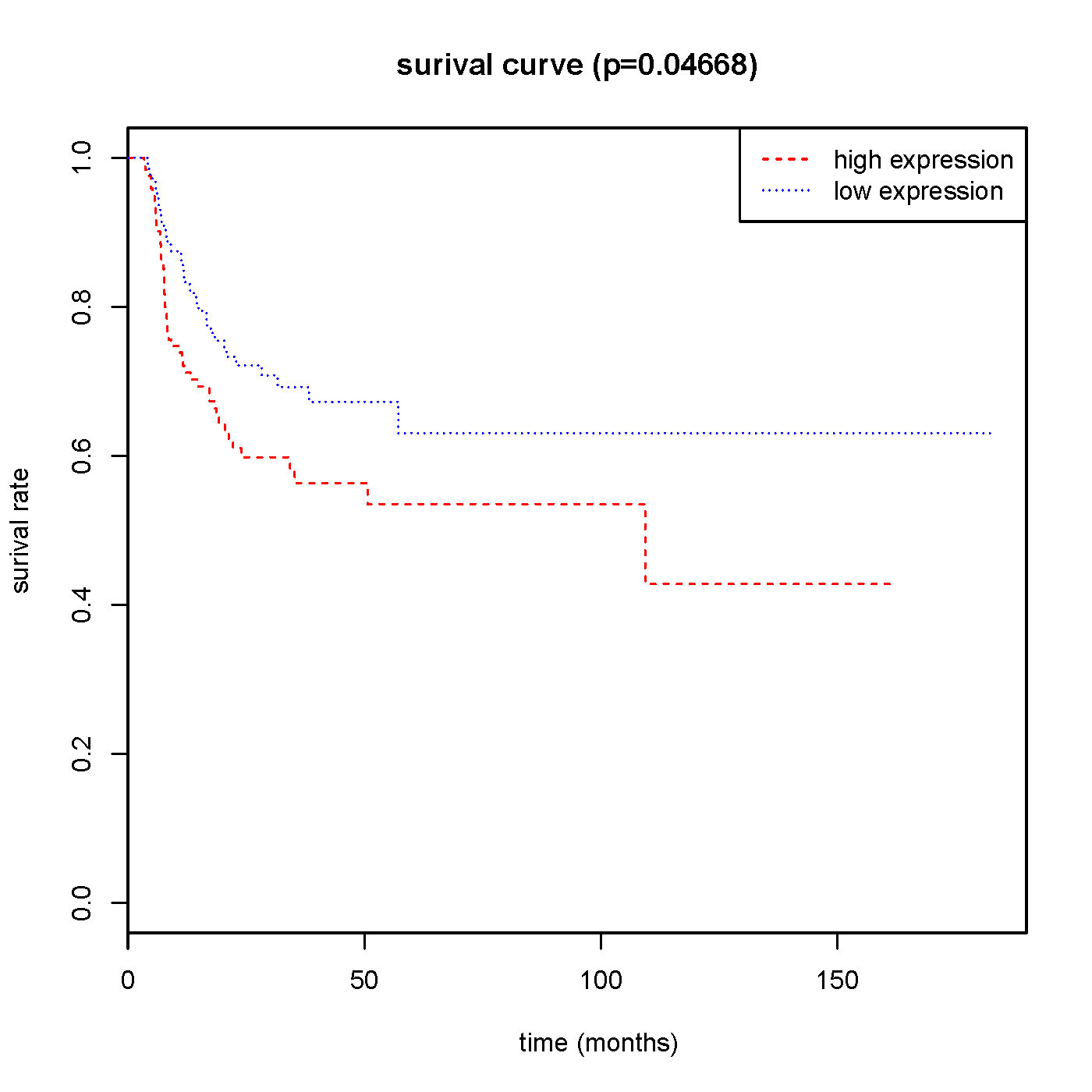

Supplement: Dataset S1 — Kaplan–Meier survival analysis with the log-rank test was used to identify relationships between the above 2493 lncRNA signatures and OSCC patient survival. Then, we determined the levels of 126 lncRNA signatures that were significantly related to DFS. [file peerj-06-5307-s005.zip › The result of Kaplan–Meier survival analyses and log-rank tests for DFS in OSCC/LINC01996.jpg]

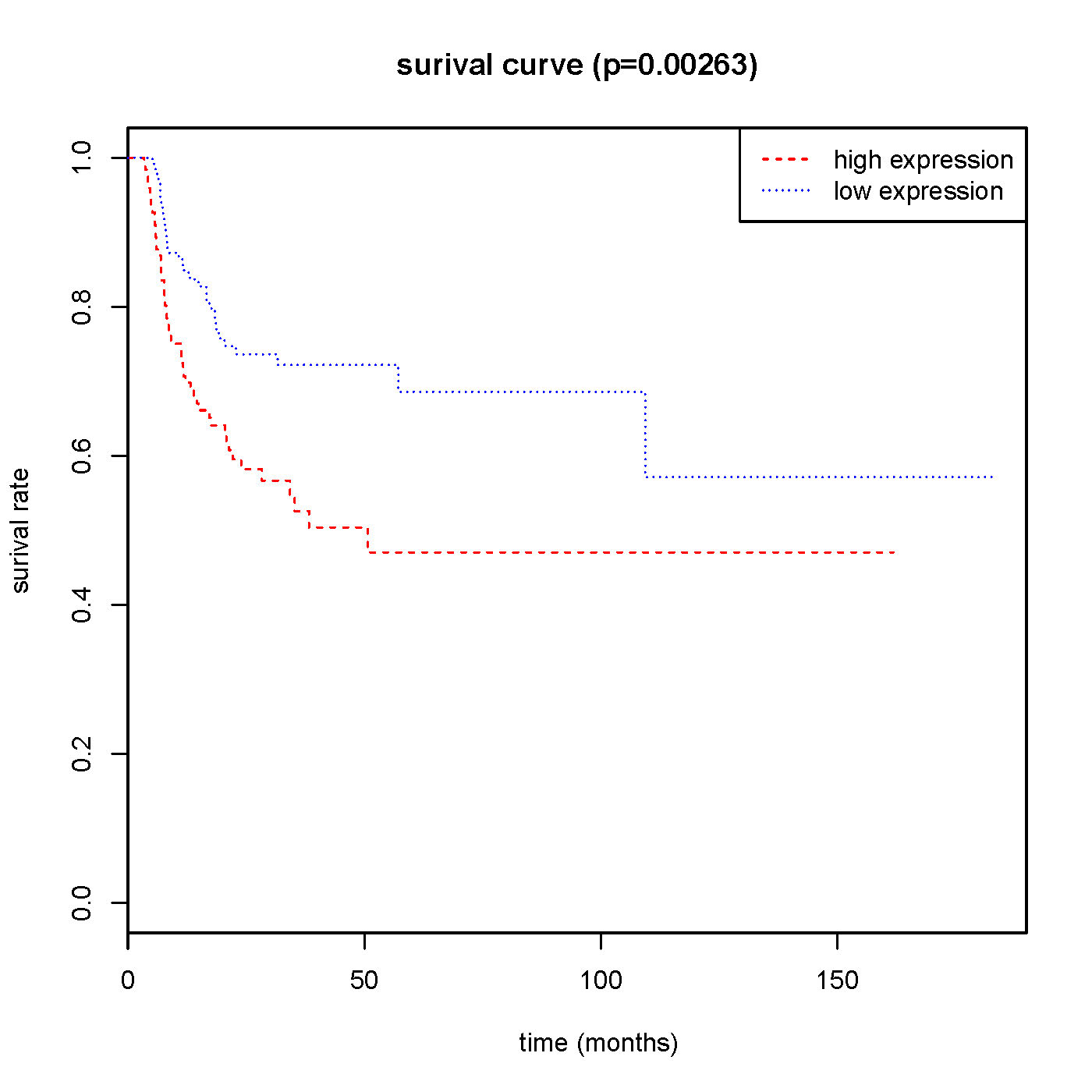

Supplement: Dataset S1 — Kaplan–Meier survival analysis with the log-rank test was used to identify relationships between the above 2493 lncRNA signatures and OSCC patient survival. Then, we determined the levels of 126 lncRNA signatures that were significantly related to DFS. [file peerj-06-5307-s005.zip › The result of Kaplan–Meier survival analyses and log-rank tests for DFS in OSCC/LINC02018.jpg]

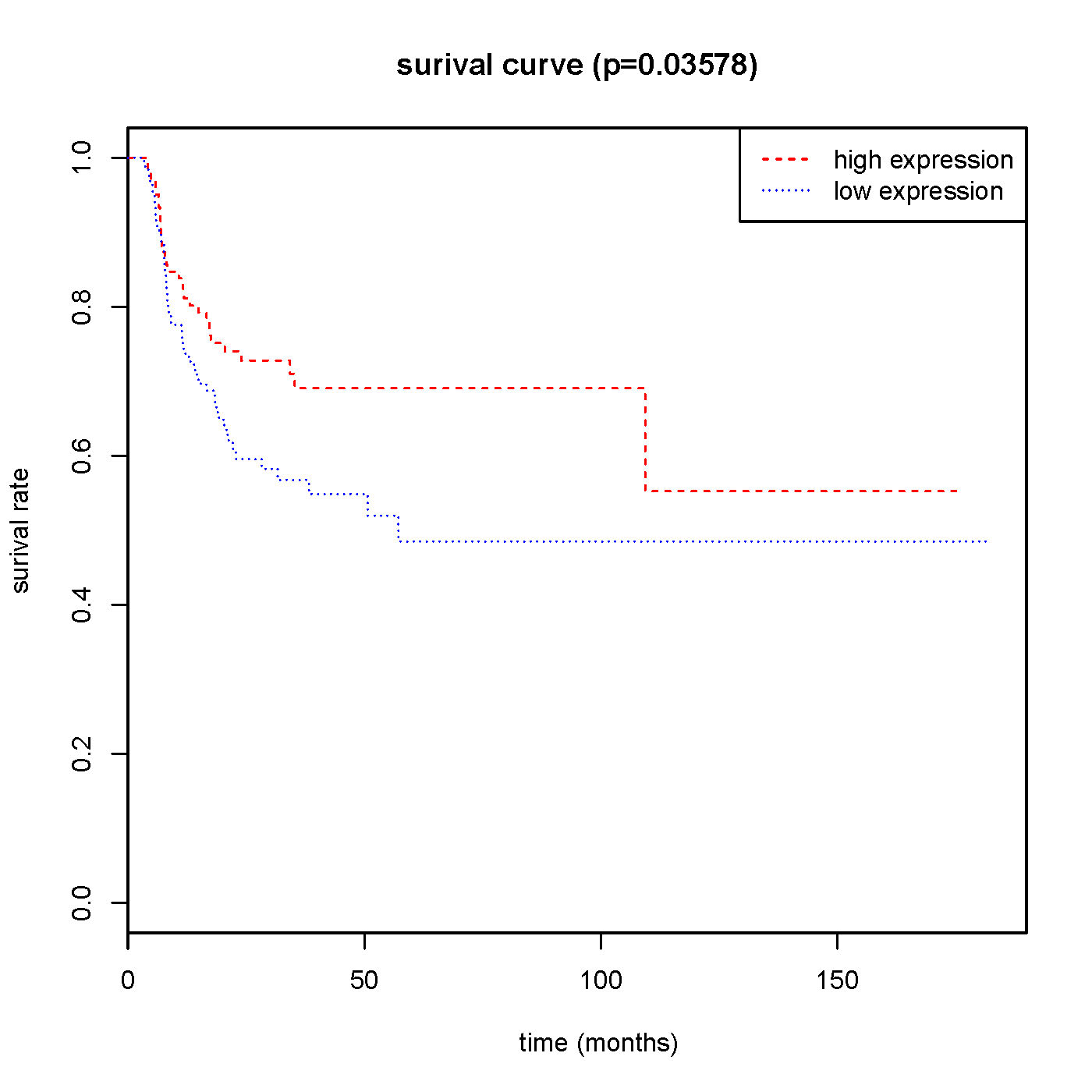

Supplement: Dataset S1 — Kaplan–Meier survival analysis with the log-rank test was used to identify relationships between the above 2493 lncRNA signatures and OSCC patient survival. Then, we determined the levels of 126 lncRNA signatures that were significantly related to DFS. [file peerj-06-5307-s005.zip › The result of Kaplan–Meier survival analyses and log-rank tests for DFS in OSCC/LINC02099.jpg]

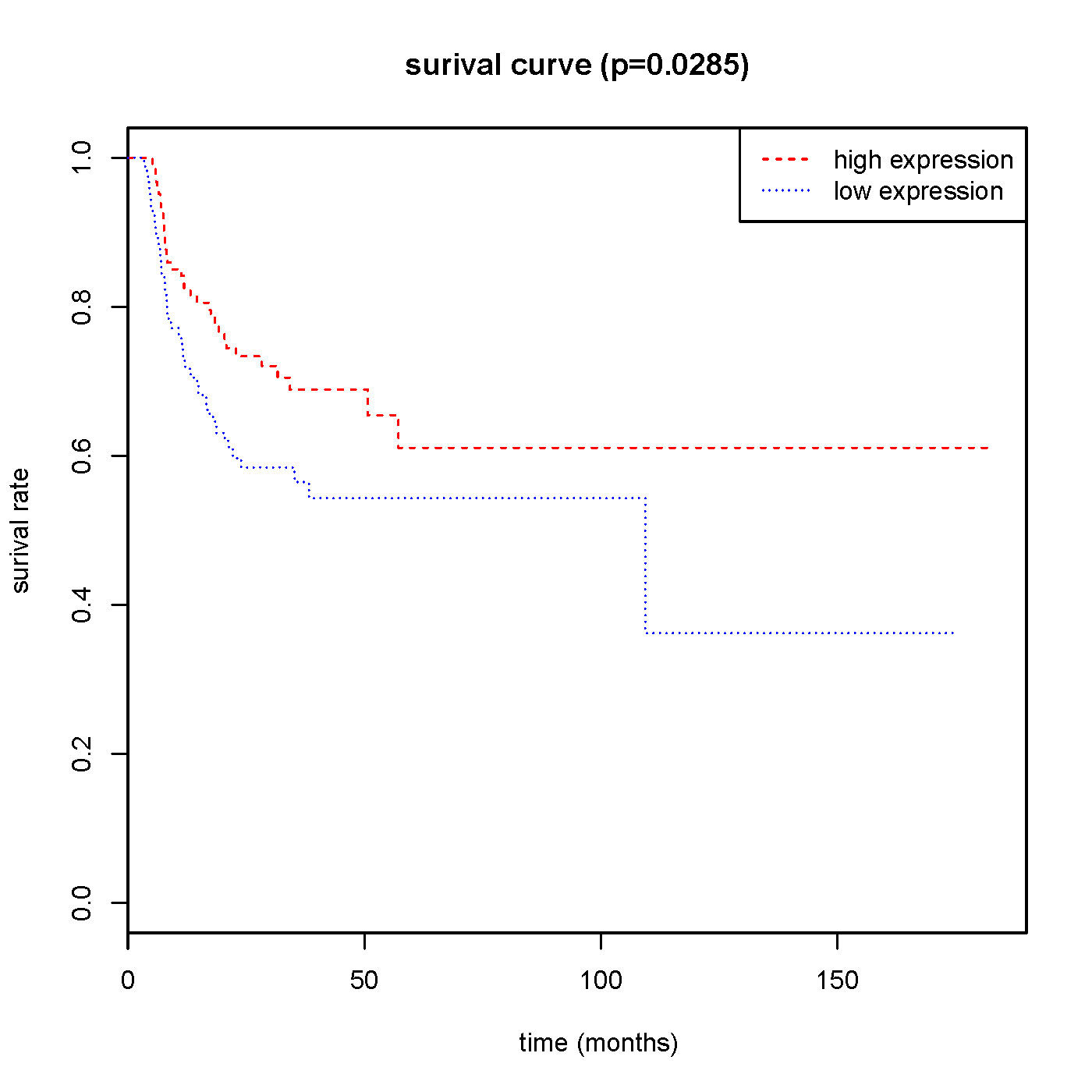

Supplement: Dataset S1 — Kaplan–Meier survival analysis with the log-rank test was used to identify relationships between the above 2493 lncRNA signatures and OSCC patient survival. Then, we determined the levels of 126 lncRNA signatures that were significantly related to DFS. [file peerj-06-5307-s005.zip › The result of Kaplan–Meier survival analyses and log-rank tests for DFS in OSCC/LINC02137.jpg]

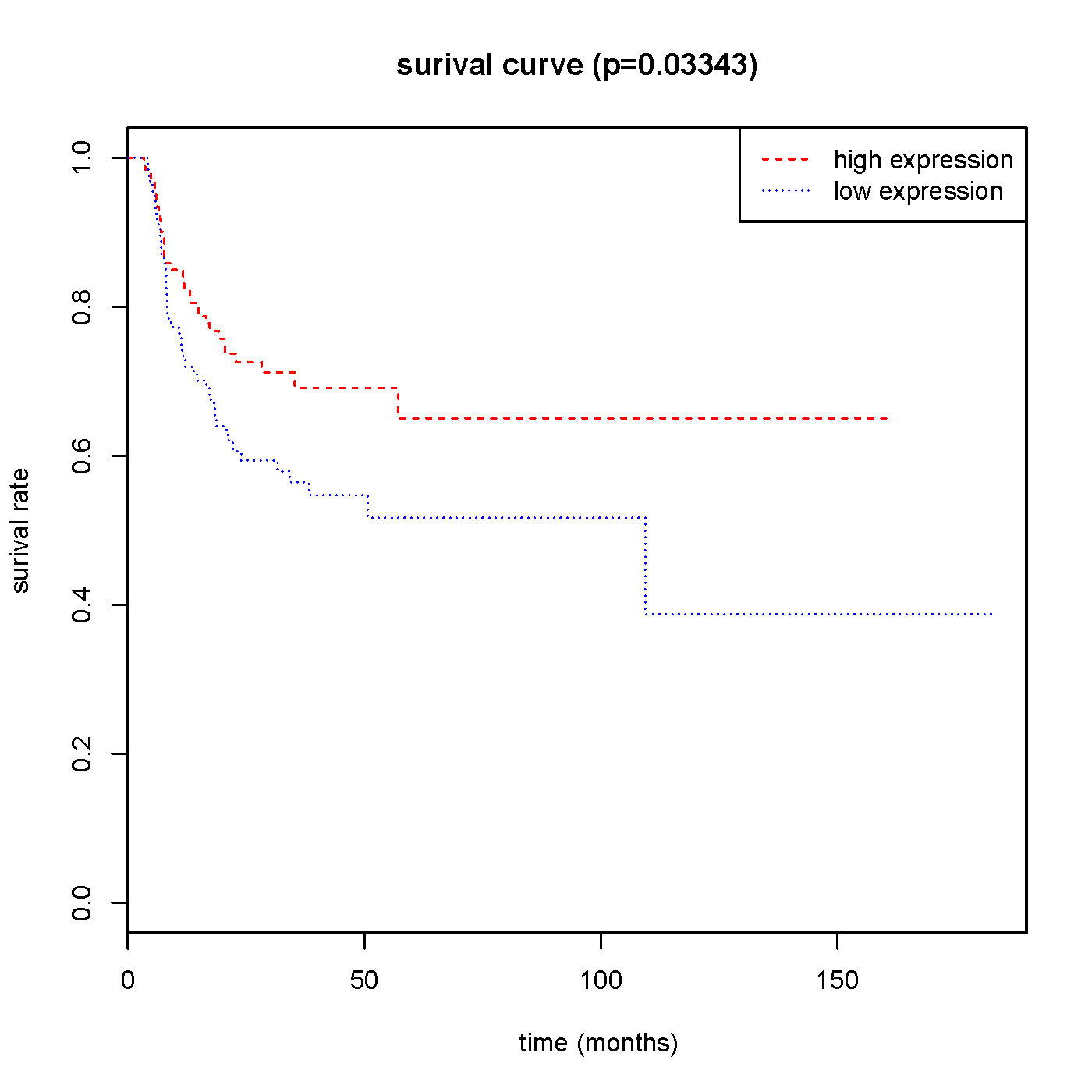

Supplement: Dataset S1 — Kaplan–Meier survival analysis with the log-rank test was used to identify relationships between the above 2493 lncRNA signatures and OSCC patient survival. Then, we determined the levels of 126 lncRNA signatures that were significantly related to DFS. [file peerj-06-5307-s005.zip › The result of Kaplan–Meier survival analyses and log-rank tests for DFS in OSCC/LINC02147.jpg]

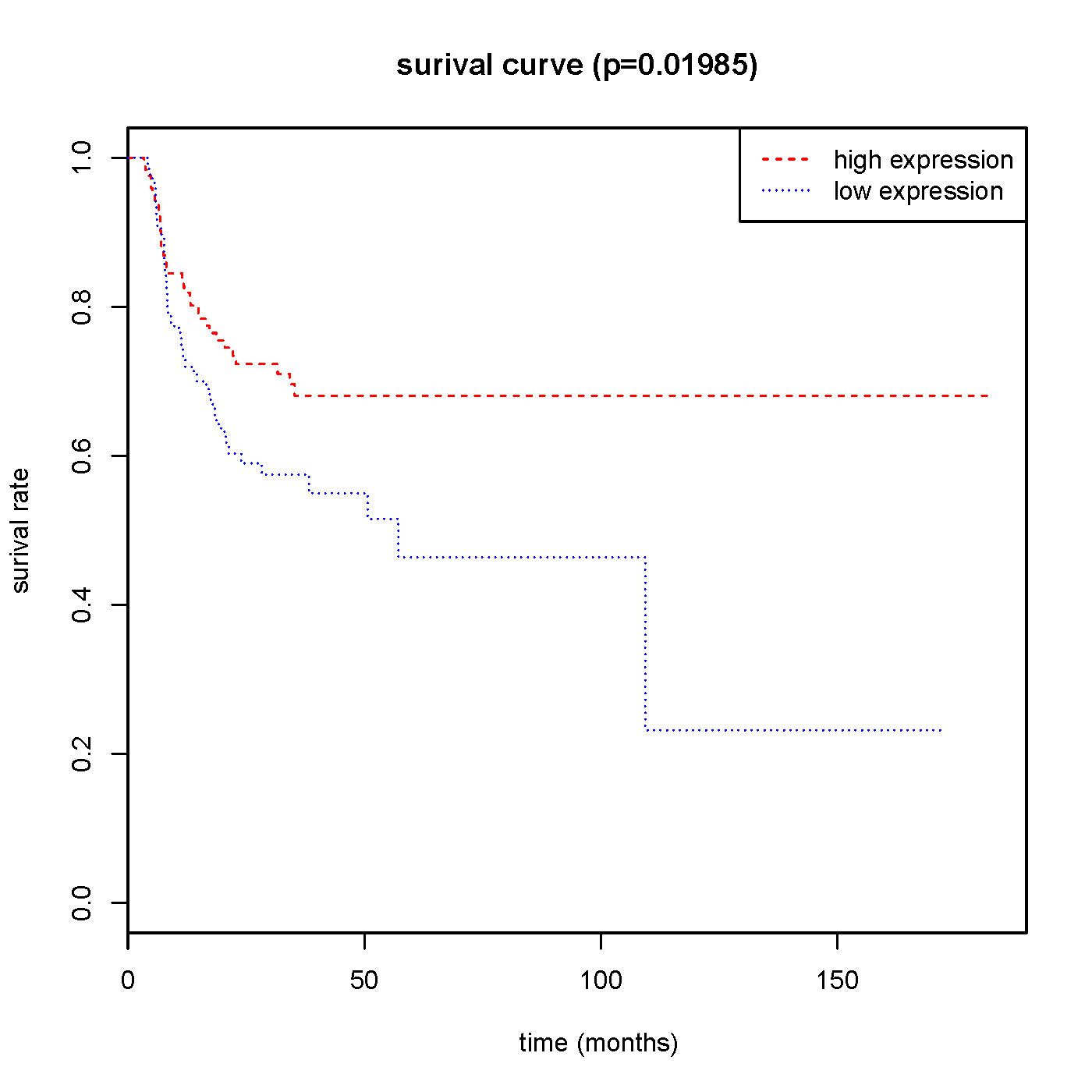

Supplement: Dataset S1 — Kaplan–Meier survival analysis with the log-rank test was used to identify relationships between the above 2493 lncRNA signatures and OSCC patient survival. Then, we determined the levels of 126 lncRNA signatures that were significantly related to DFS. [file peerj-06-5307-s005.zip › The result of Kaplan–Meier survival analyses and log-rank tests for DFS in OSCC/MAGI2-AS3.jpg]

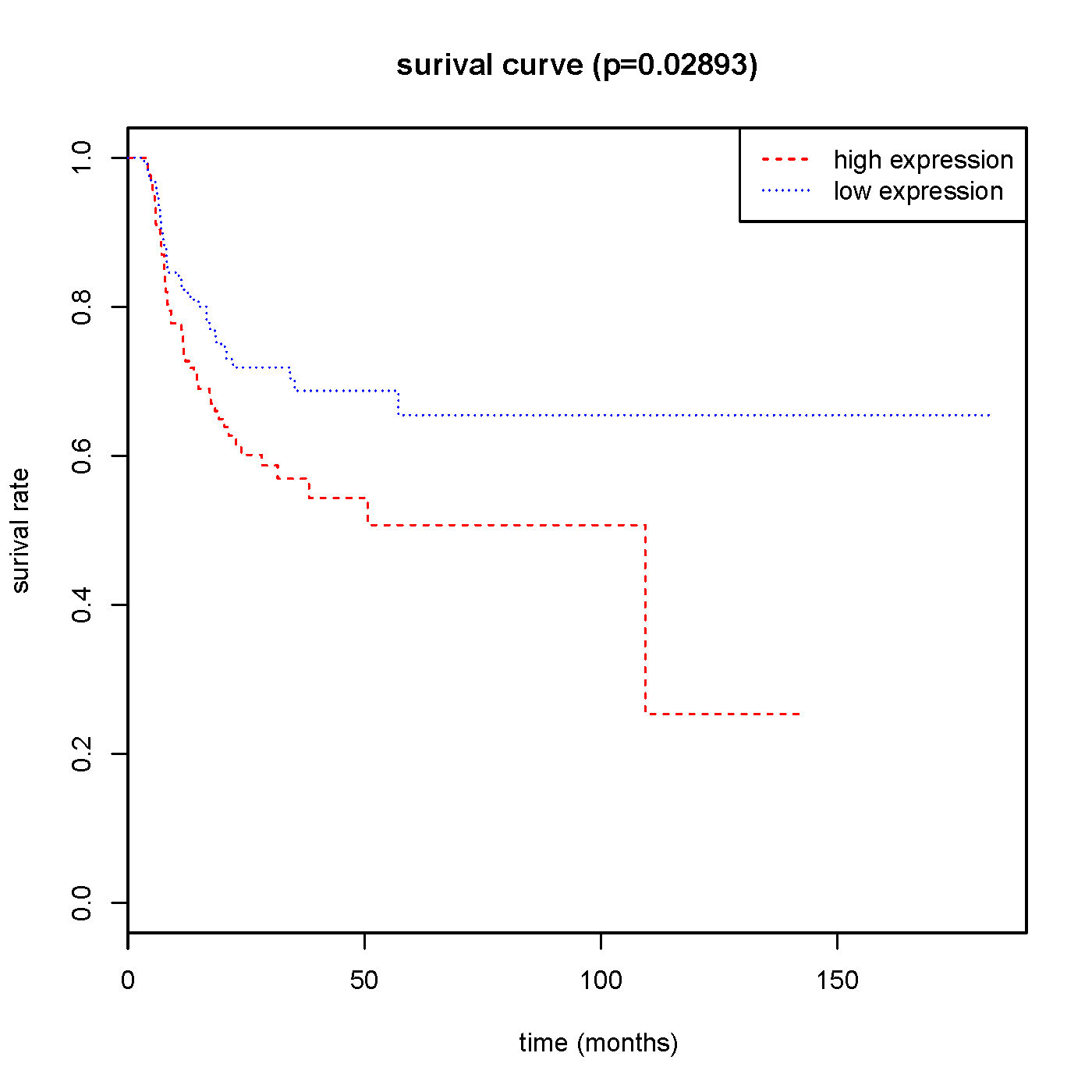

Supplement: Dataset S1 — Kaplan–Meier survival analysis with the log-rank test was used to identify relationships between the above 2493 lncRNA signatures and OSCC patient survival. Then, we determined the levels of 126 lncRNA signatures that were significantly related to DFS. [file peerj-06-5307-s005.zip › The result of Kaplan–Meier survival analyses and log-rank tests for DFS in OSCC/MIR17HG.jpg]

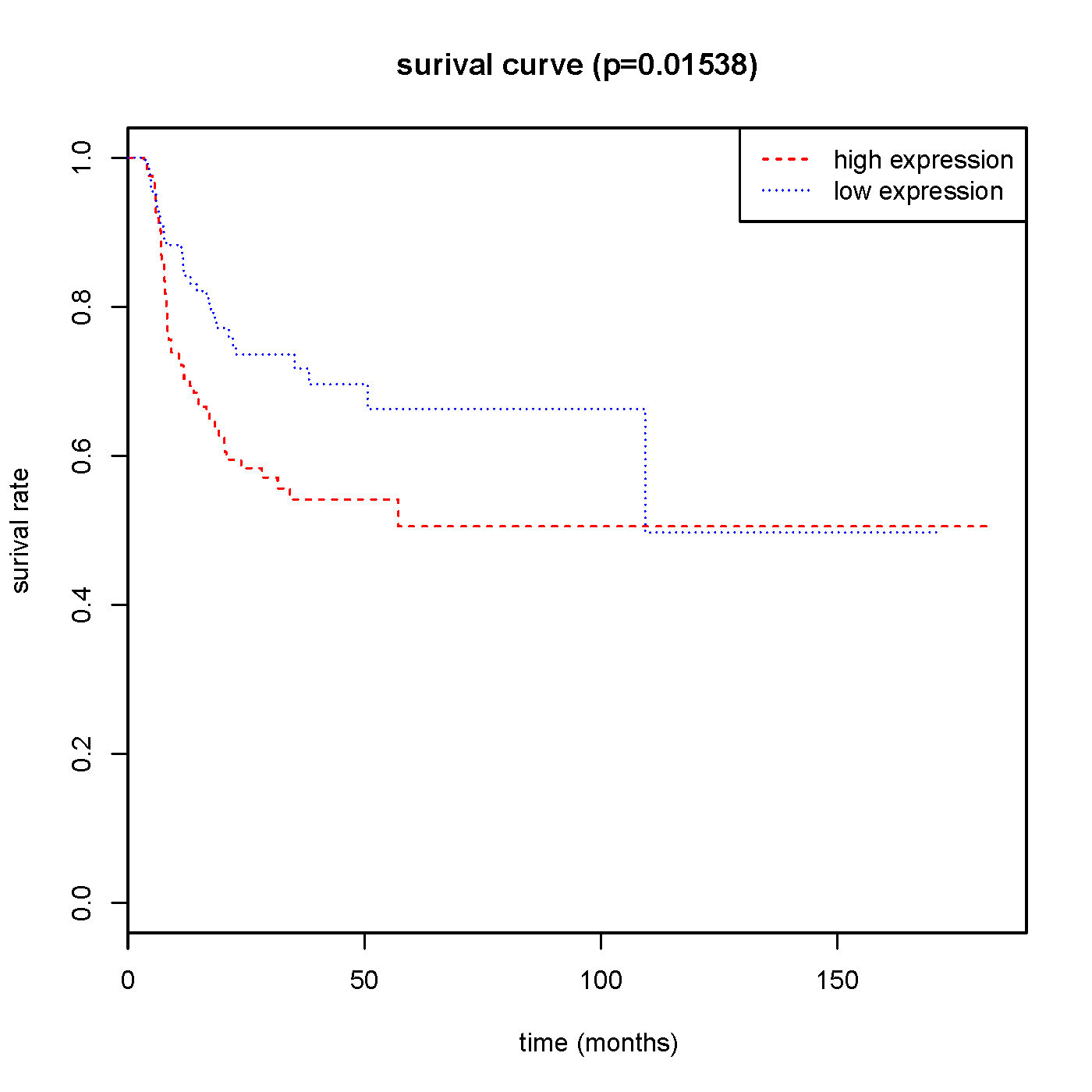

Supplement: Dataset S1 — Kaplan–Meier survival analysis with the log-rank test was used to identify relationships between the above 2493 lncRNA signatures and OSCC patient survival. Then, we determined the levels of 126 lncRNA signatures that were significantly related to DFS. [file peerj-06-5307-s005.zip › The result of Kaplan–Meier survival analyses and log-rank tests for DFS in OSCC/MYO16-AS1.jpg]

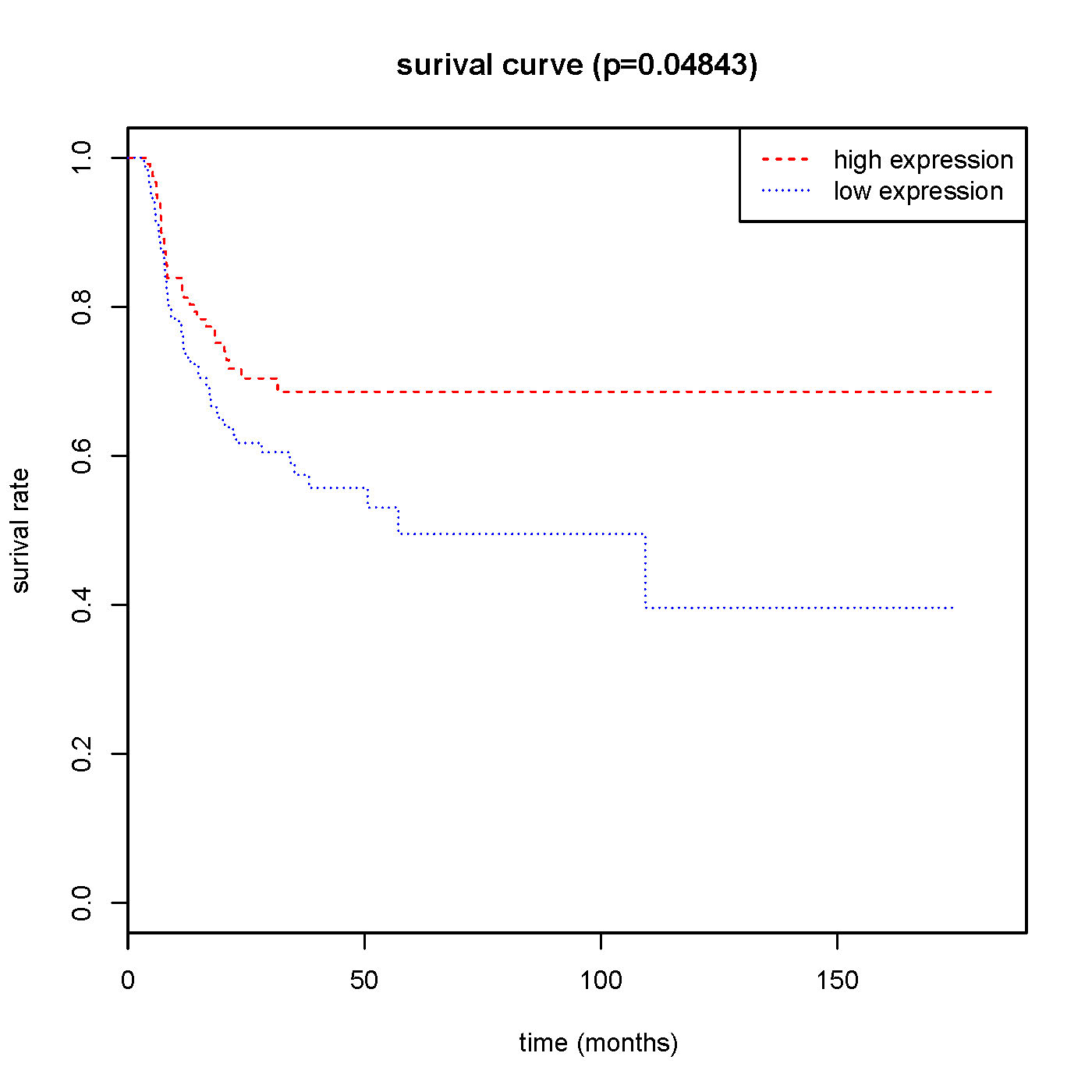

Supplement: Dataset S1 — Kaplan–Meier survival analysis with the log-rank test was used to identify relationships between the above 2493 lncRNA signatures and OSCC patient survival. Then, we determined the levels of 126 lncRNA signatures that were significantly related to DFS. [file peerj-06-5307-s005.zip › The result of Kaplan–Meier survival analyses and log-rank tests for DFS in OSCC/RP1-224A6.9.jpg]

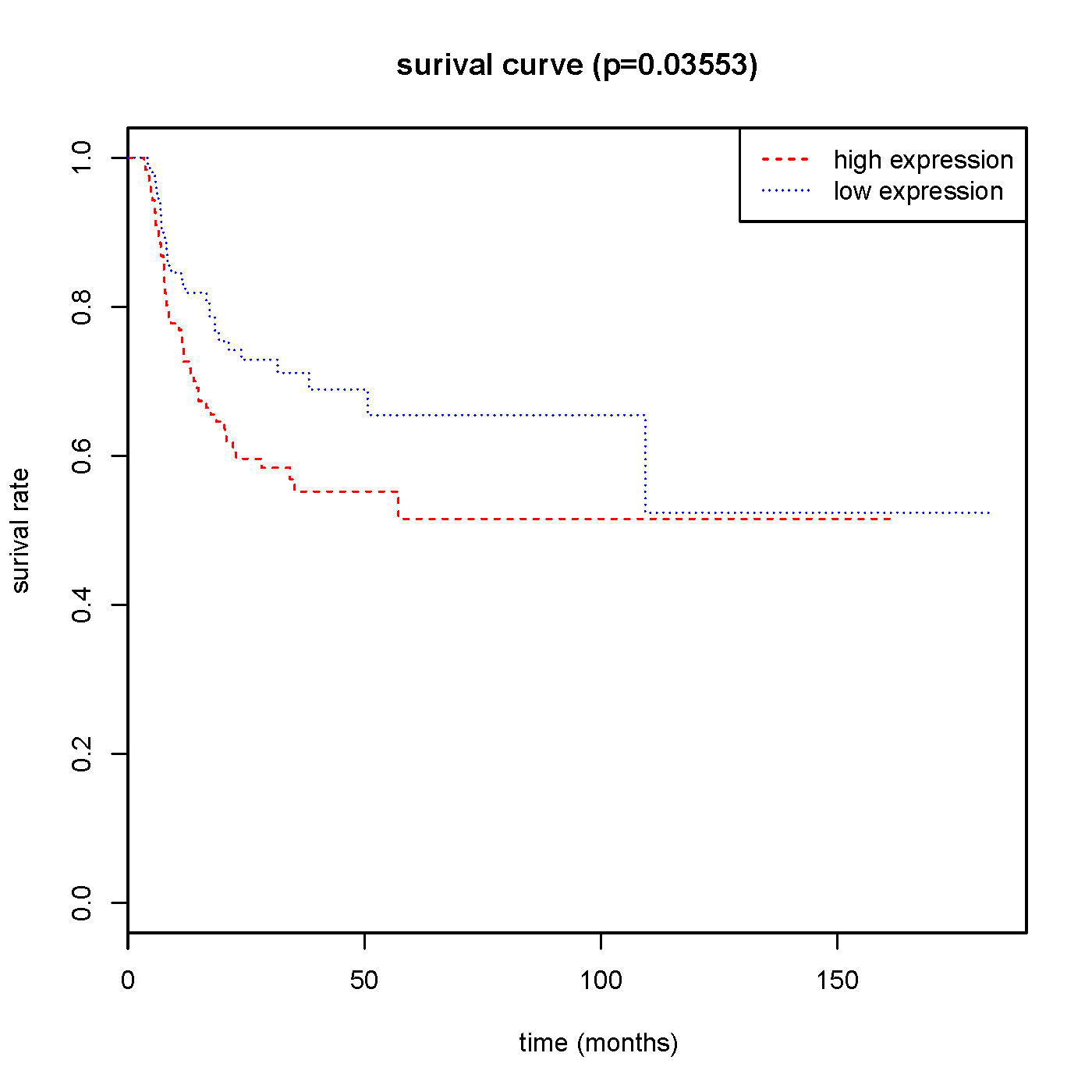

Supplement: Dataset S1 — Kaplan–Meier survival analysis with the log-rank test was used to identify relationships between the above 2493 lncRNA signatures and OSCC patient survival. Then, we determined the levels of 126 lncRNA signatures that were significantly related to DFS. [file peerj-06-5307-s005.zip › The result of Kaplan–Meier survival analyses and log-rank tests for DFS in OSCC/RP1-56K13.5.jpg]

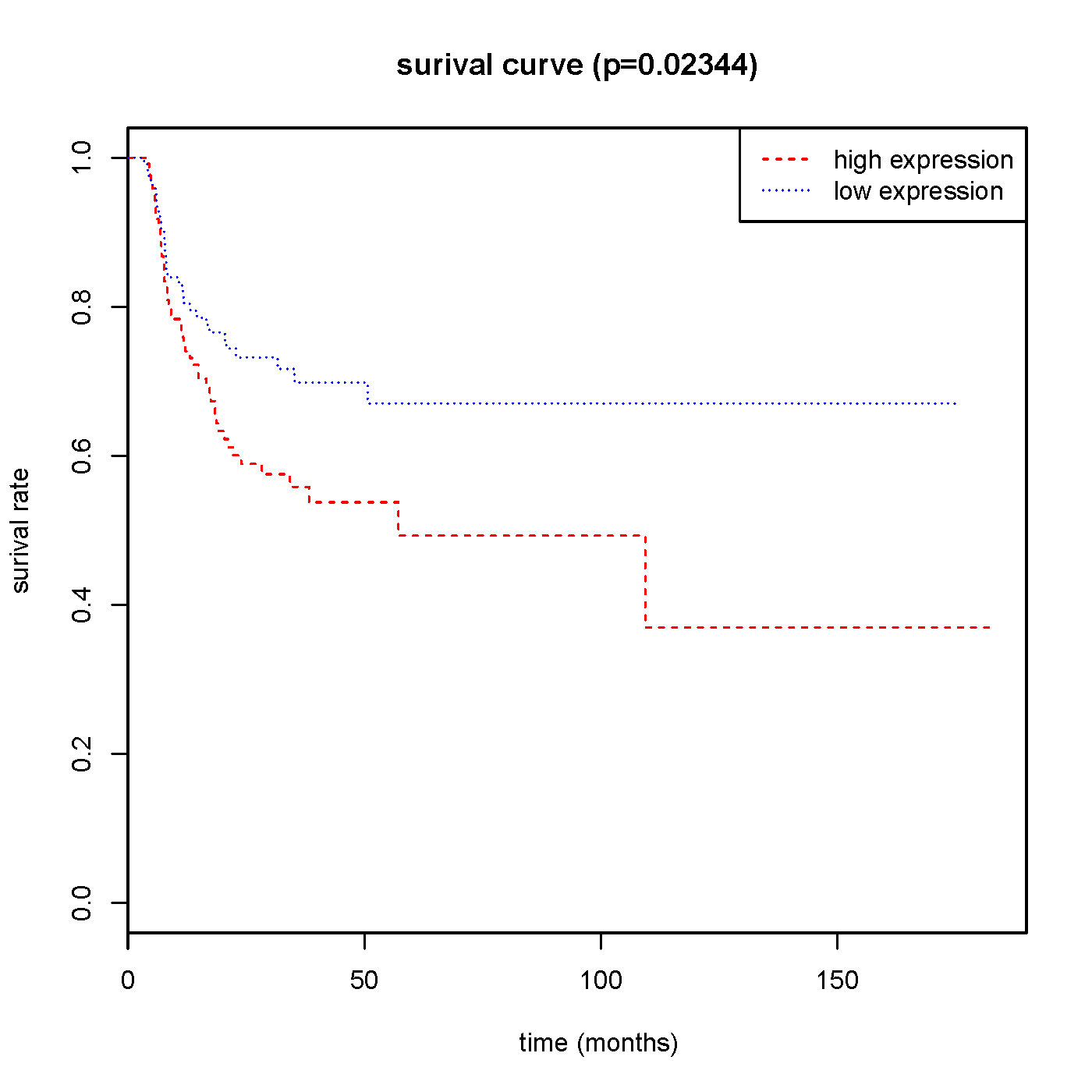

Supplement: Dataset S1 — Kaplan–Meier survival analysis with the log-rank test was used to identify relationships between the above 2493 lncRNA signatures and OSCC patient survival. Then, we determined the levels of 126 lncRNA signatures that were significantly related to DFS. [file peerj-06-5307-s005.zip › The result of Kaplan–Meier survival analyses and log-rank tests for DFS in OSCC/RP11-104O19.4.jpg]

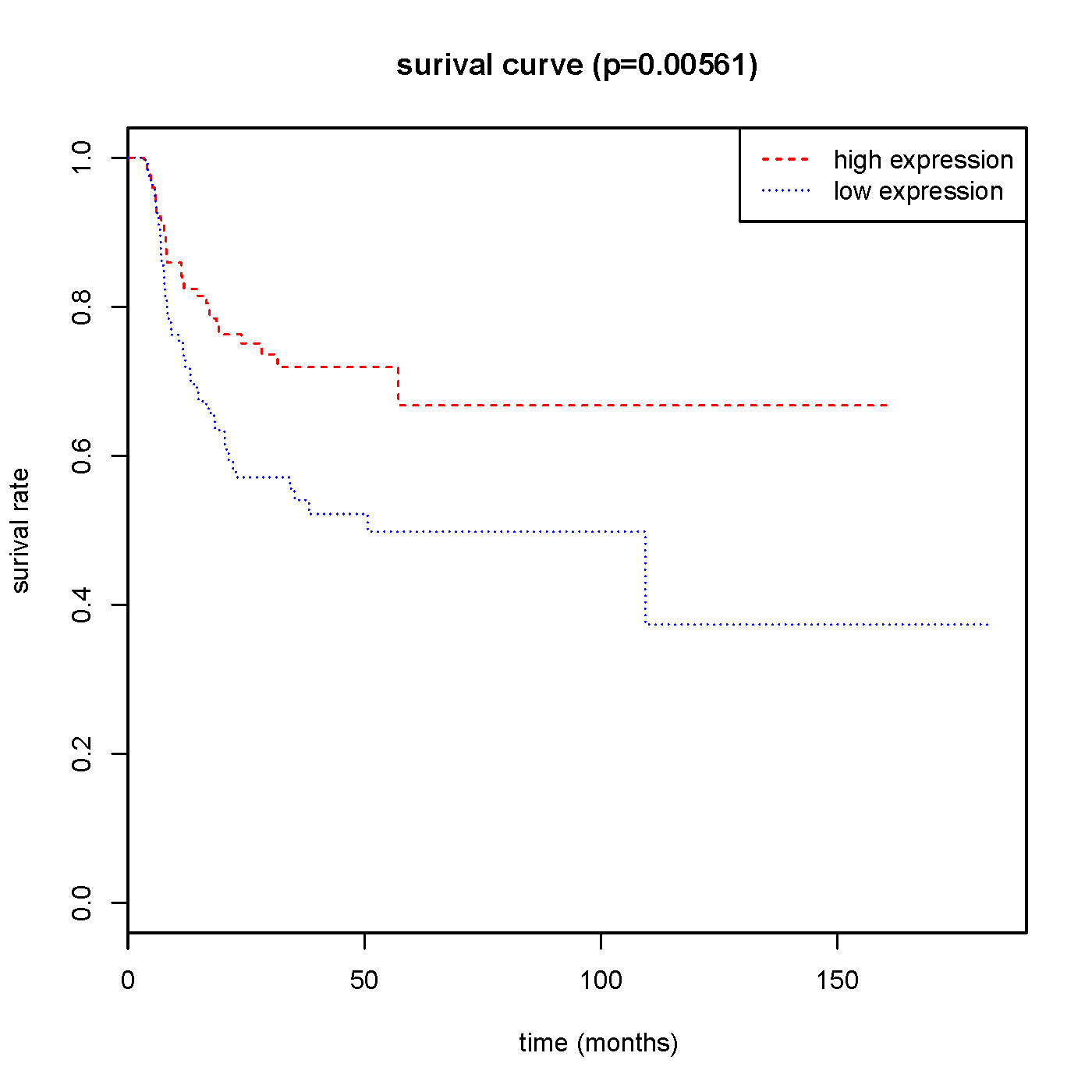

Supplement: Dataset S1 — Kaplan–Meier survival analysis with the log-rank test was used to identify relationships between the above 2493 lncRNA signatures and OSCC patient survival. Then, we determined the levels of 126 lncRNA signatures that were significantly related to DFS. [file peerj-06-5307-s005.zip › The result of Kaplan–Meier survival analyses and log-rank tests for DFS in OSCC/RP11-1055B8.2.jpg]

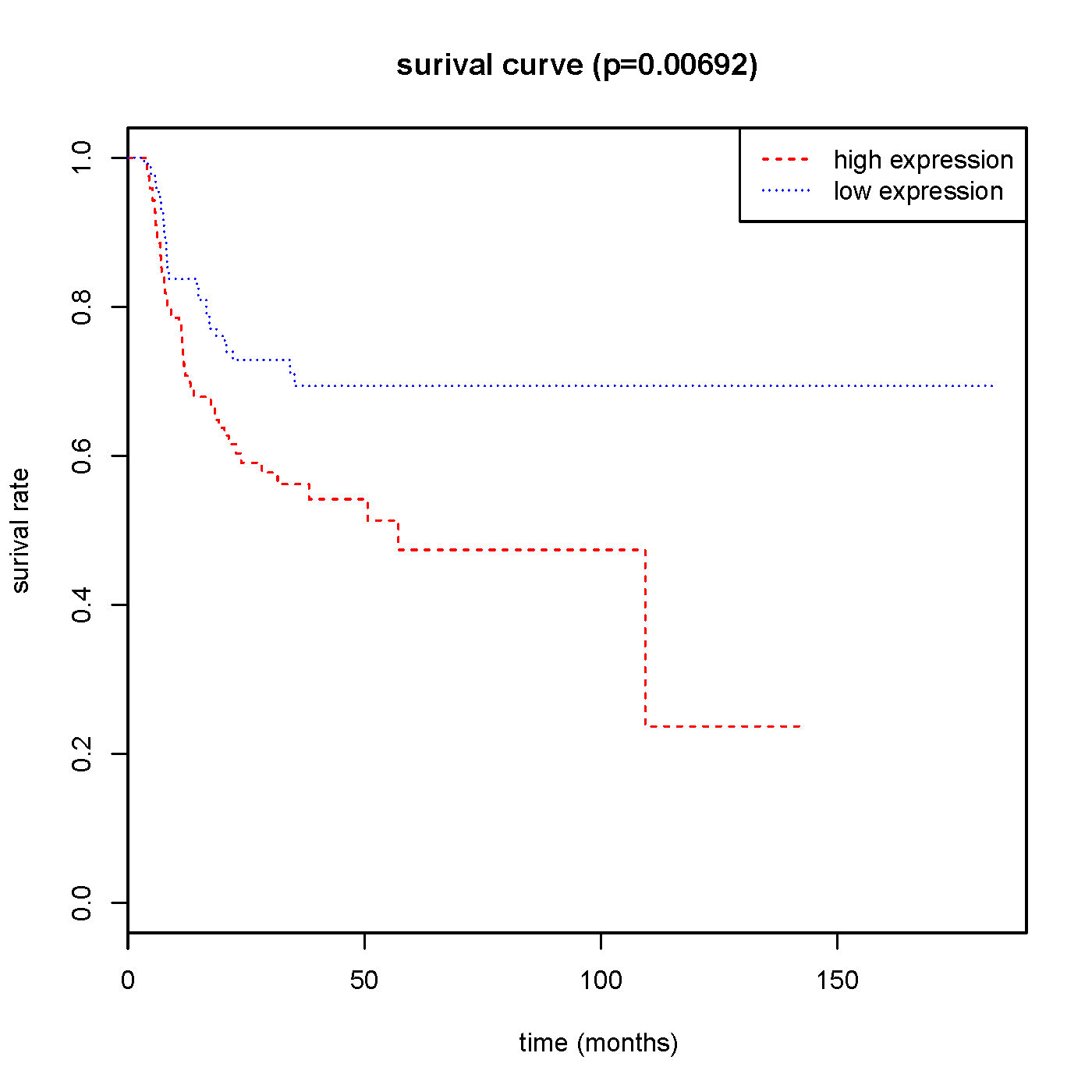

Supplement: Dataset S1 — Kaplan–Meier survival analysis with the log-rank test was used to identify relationships between the above 2493 lncRNA signatures and OSCC patient survival. Then, we determined the levels of 126 lncRNA signatures that were significantly related to DFS. [file peerj-06-5307-s005.zip › The result of Kaplan–Meier survival analyses and log-rank tests for DFS in OSCC/RP11-106M3.3.jpg]

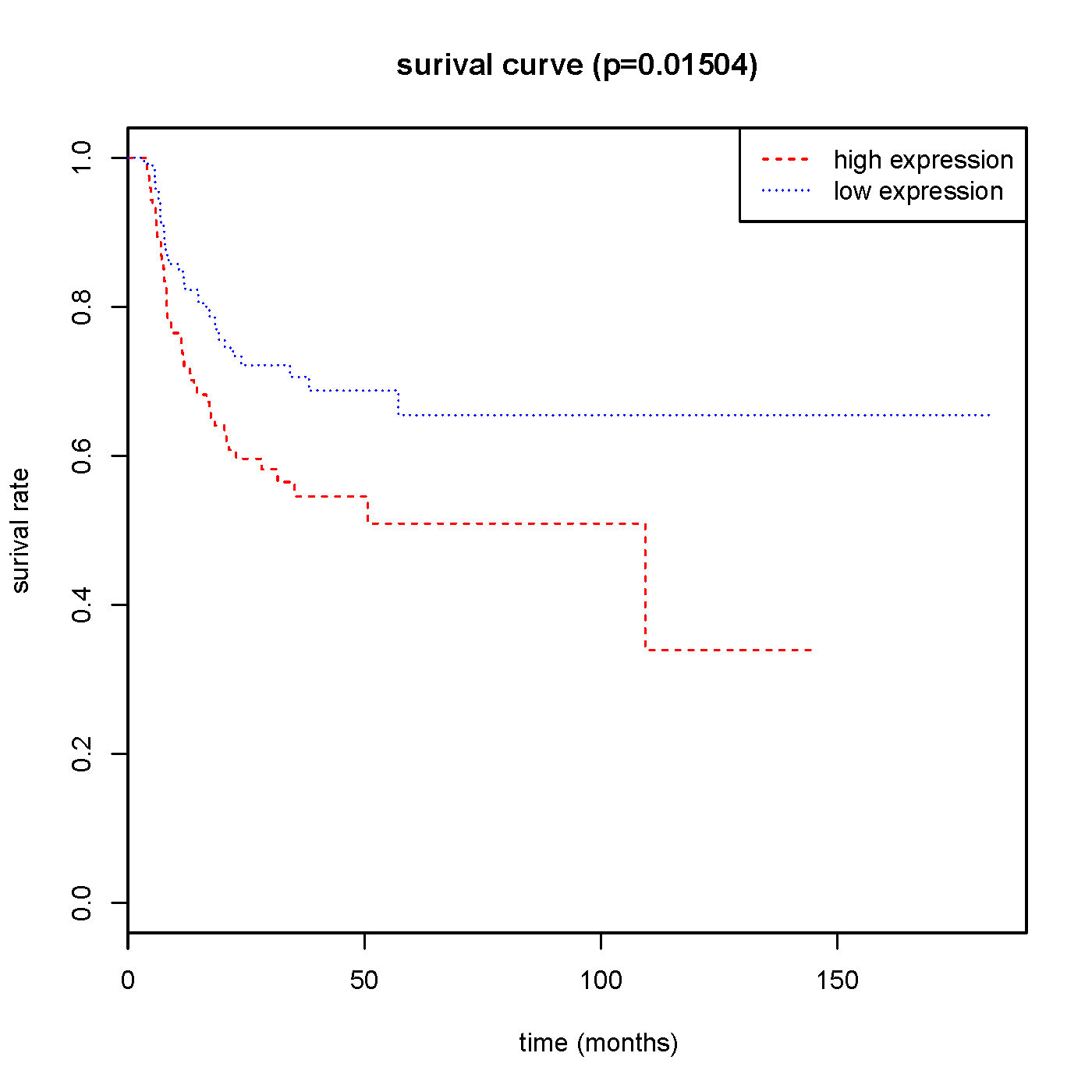

Supplement: Dataset S1 — Kaplan–Meier survival analysis with the log-rank test was used to identify relationships between the above 2493 lncRNA signatures and OSCC patient survival. Then, we determined the levels of 126 lncRNA signatures that were significantly related to DFS. [file peerj-06-5307-s005.zip › The result of Kaplan–Meier survival analyses and log-rank tests for DFS in OSCC/RP11-107M16.2.jpg]

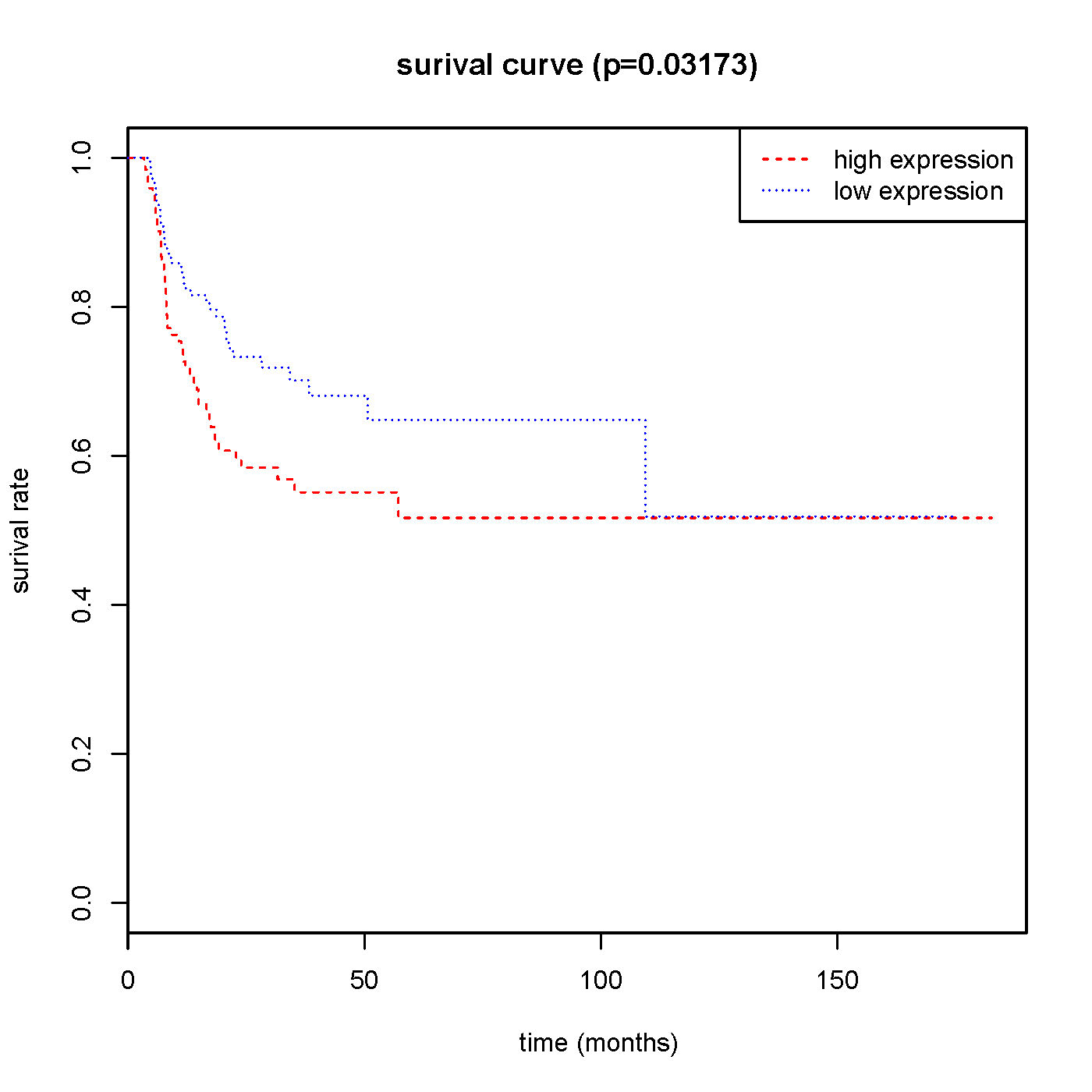

Supplement: Dataset S1 — Kaplan–Meier survival analysis with the log-rank test was used to identify relationships between the above 2493 lncRNA signatures and OSCC patient survival. Then, we determined the levels of 126 lncRNA signatures that were significantly related to DFS. [file peerj-06-5307-s005.zip › The result of Kaplan–Meier survival analyses and log-rank tests for DFS in OSCC/RP11-108K3.2.jpg]

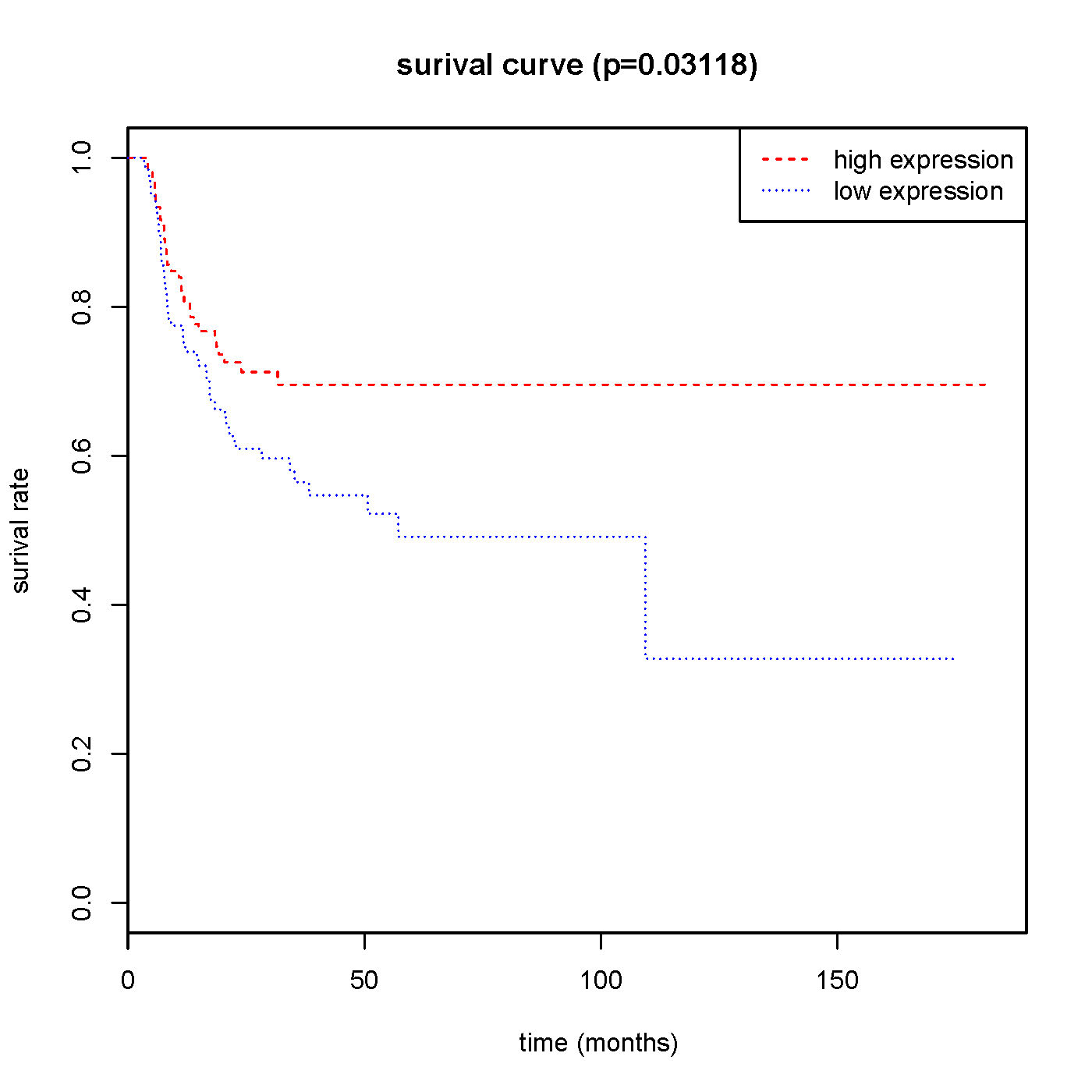

Supplement: Dataset S1 — Kaplan–Meier survival analysis with the log-rank test was used to identify relationships between the above 2493 lncRNA signatures and OSCC patient survival. Then, we determined the levels of 126 lncRNA signatures that were significantly related to DFS. [file peerj-06-5307-s005.zip › The result of Kaplan–Meier survival analyses and log-rank tests for DFS in OSCC/RP11-120K18.2.jpg]

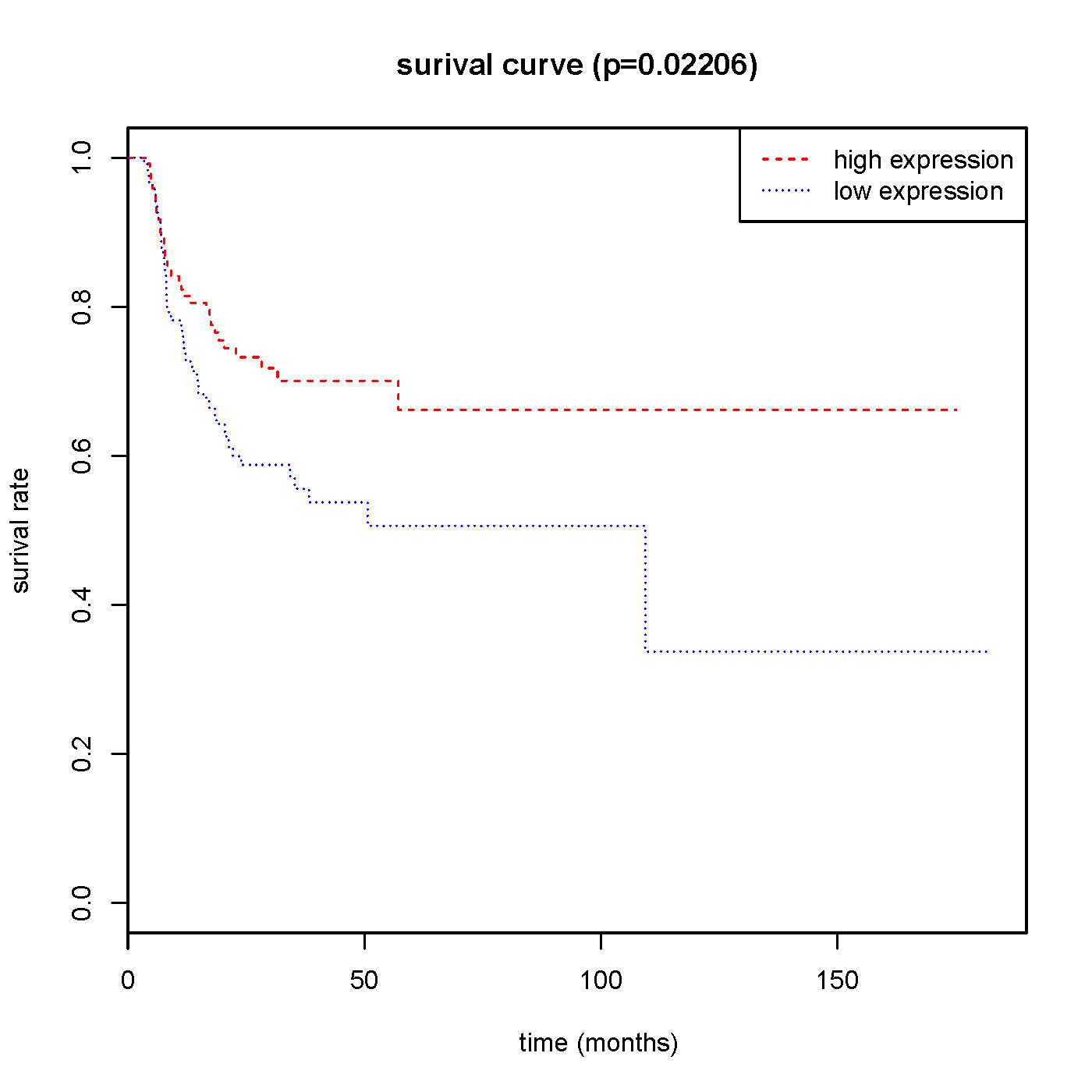

Supplement: Dataset S1 — Kaplan–Meier survival analysis with the log-rank test was used to identify relationships between the above 2493 lncRNA signatures and OSCC patient survival. Then, we determined the levels of 126 lncRNA signatures that were significantly related to DFS. [file peerj-06-5307-s005.zip › The result of Kaplan–Meier survival analyses and log-rank tests for DFS in OSCC/RP11-120K24.5.jpg]

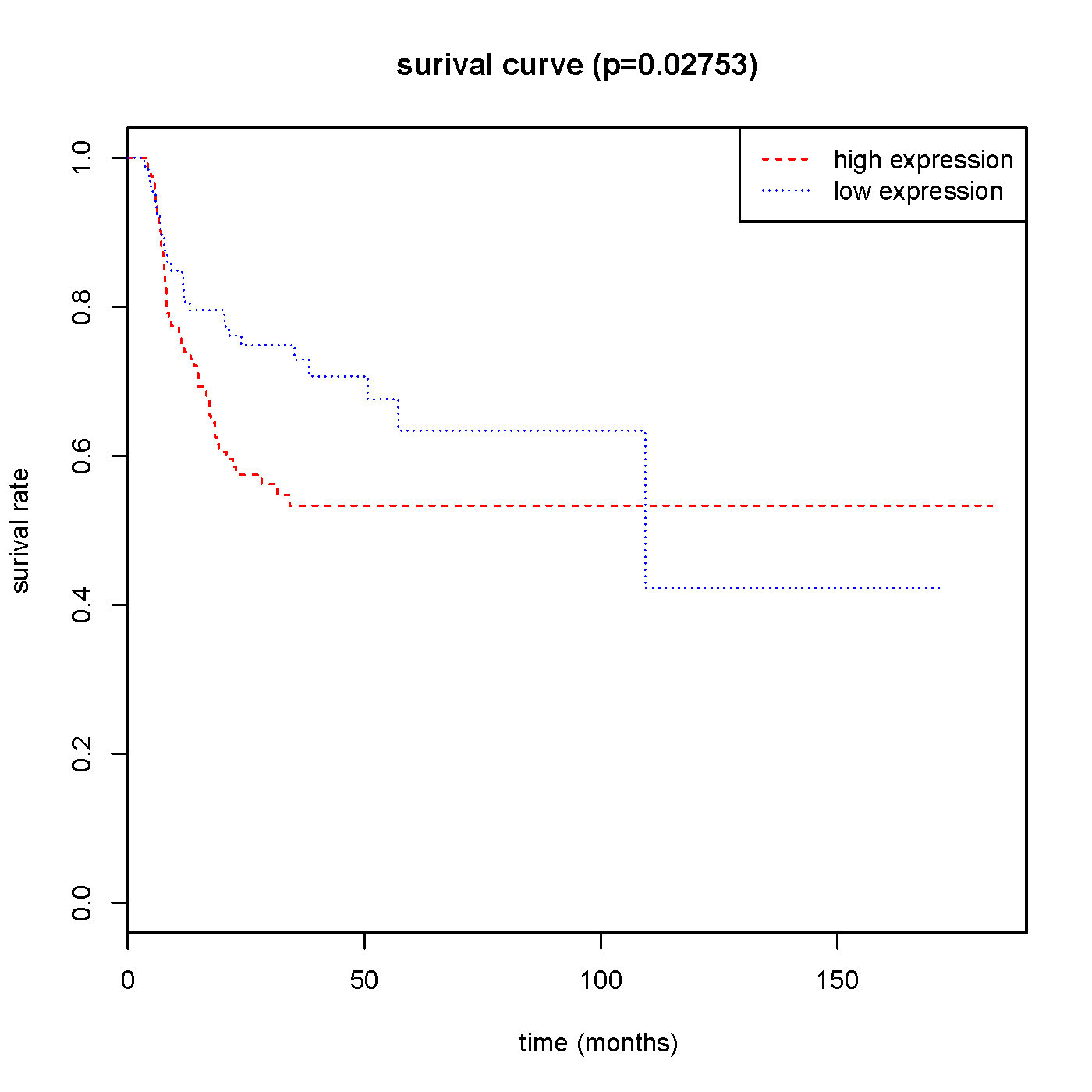

Supplement: Dataset S1 — Kaplan–Meier survival analysis with the log-rank test was used to identify relationships between the above 2493 lncRNA signatures and OSCC patient survival. Then, we determined the levels of 126 lncRNA signatures that were significantly related to DFS. [file peerj-06-5307-s005.zip › The result of Kaplan–Meier survival analyses and log-rank tests for DFS in OSCC/RP11-138E9.2.jpg]

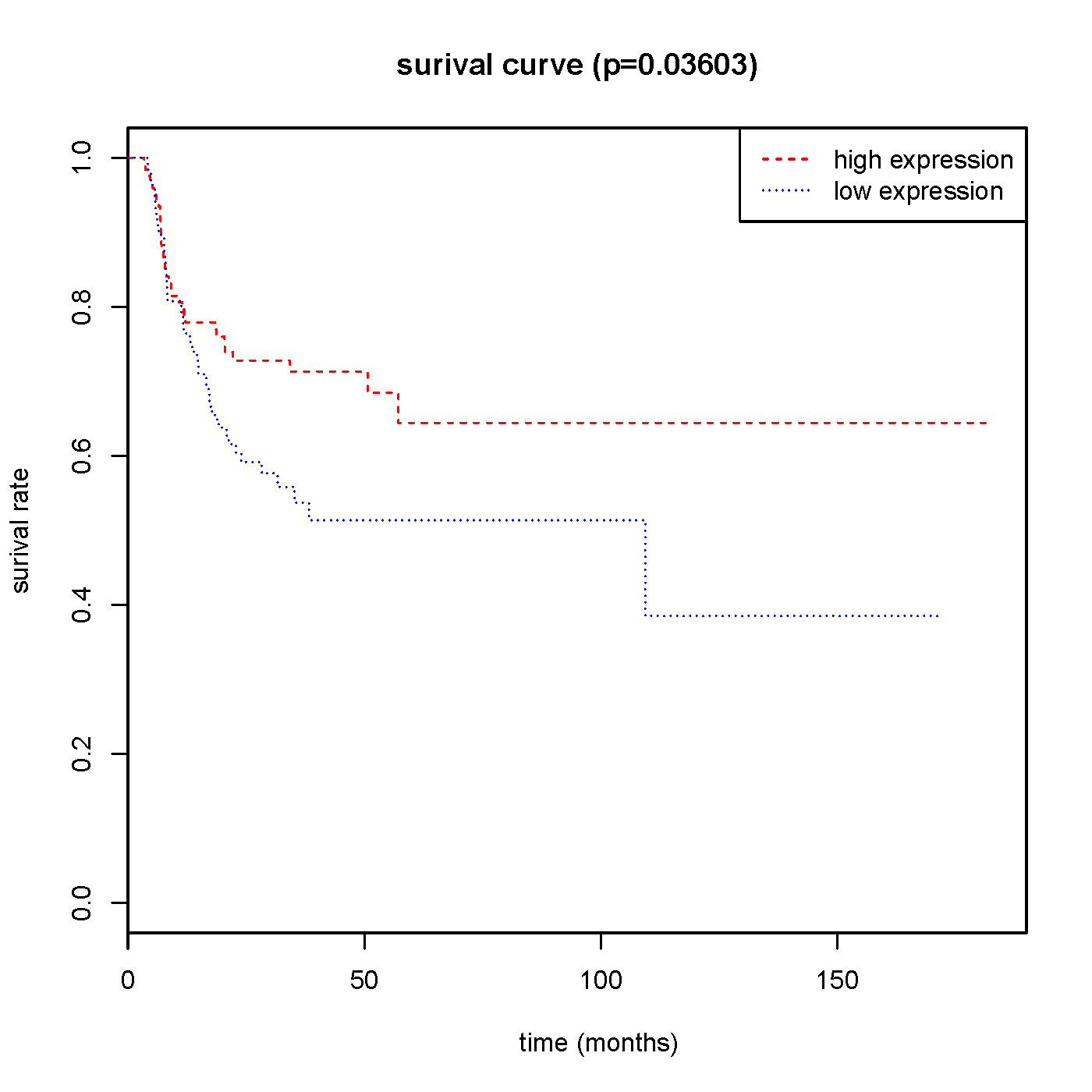

Supplement: Dataset S1 — Kaplan–Meier survival analysis with the log-rank test was used to identify relationships between the above 2493 lncRNA signatures and OSCC patient survival. Then, we determined the levels of 126 lncRNA signatures that were significantly related to DFS. [file peerj-06-5307-s005.zip › The result of Kaplan–Meier survival analyses and log-rank tests for DFS in OSCC/RP11-146E13.4.jpg]

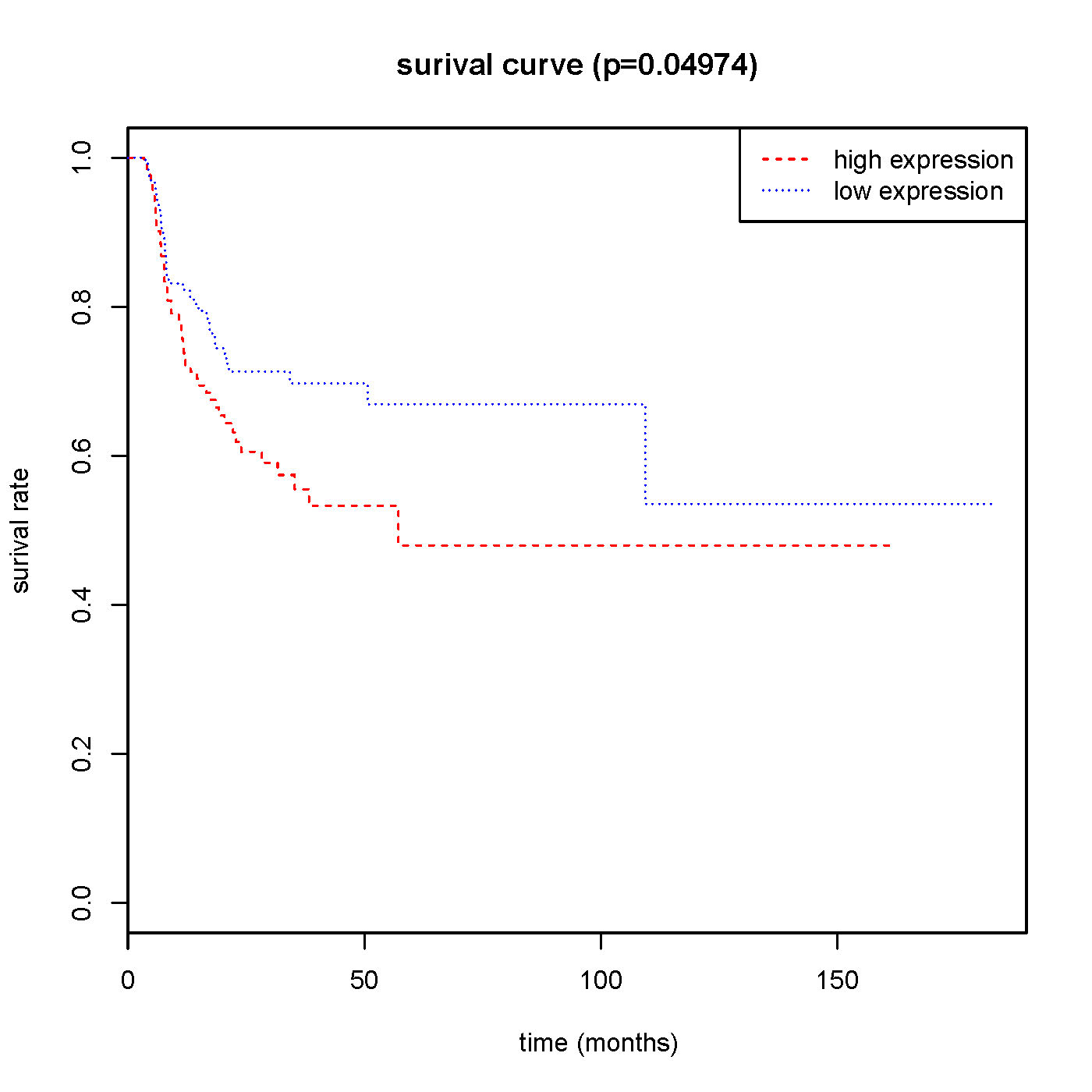

Supplement: Dataset S1 — Kaplan–Meier survival analysis with the log-rank test was used to identify relationships between the above 2493 lncRNA signatures and OSCC patient survival. Then, we determined the levels of 126 lncRNA signatures that were significantly related to DFS. [file peerj-06-5307-s005.zip › The result of Kaplan–Meier survival analyses and log-rank tests for DFS in OSCC/RP11-146F11.5.jpg]

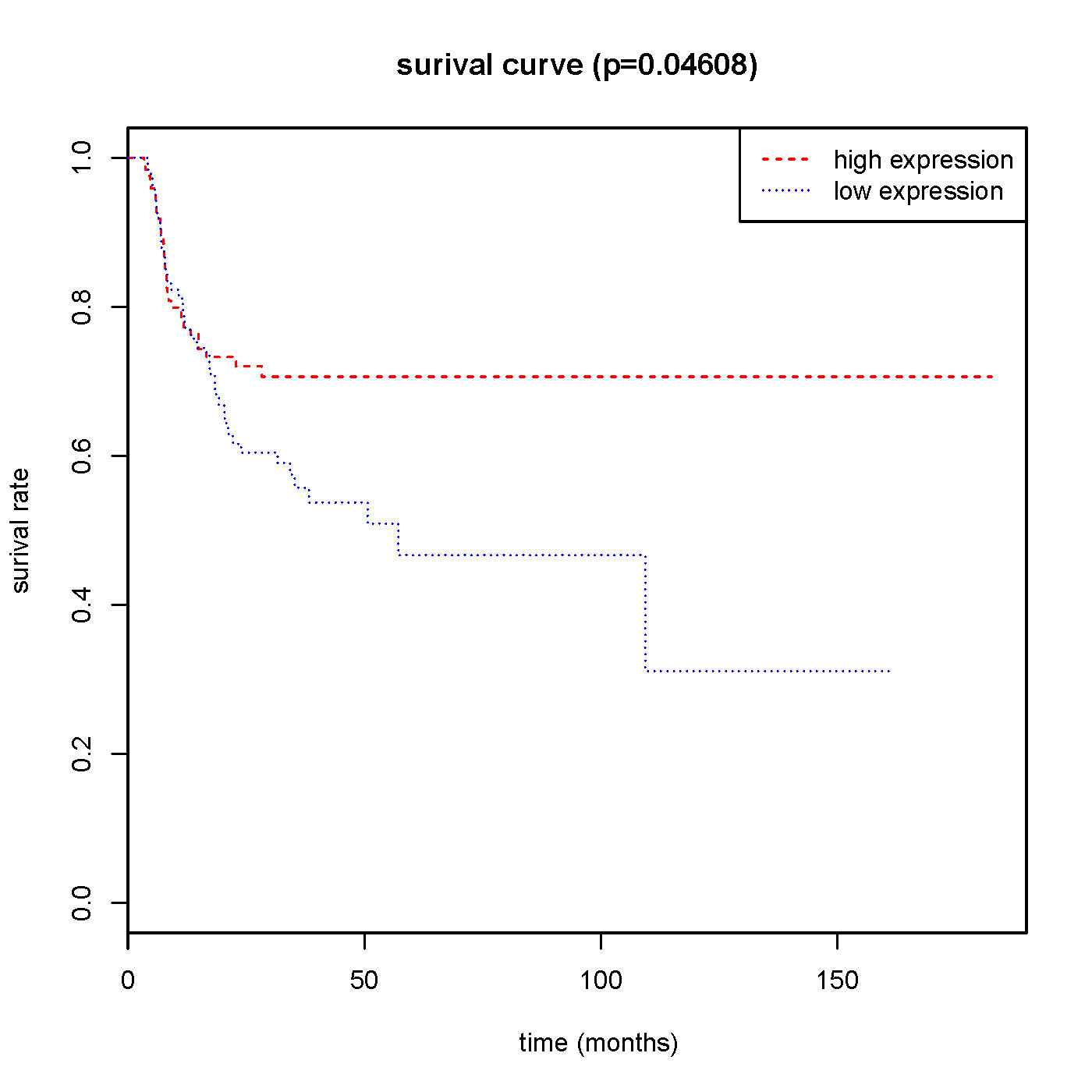

Supplement: Dataset S1 — Kaplan–Meier survival analysis with the log-rank test was used to identify relationships between the above 2493 lncRNA signatures and OSCC patient survival. Then, we determined the levels of 126 lncRNA signatures that were significantly related to DFS. [file peerj-06-5307-s005.zip › The result of Kaplan–Meier survival analyses and log-rank tests for DFS in OSCC/RP11-167H9.4.jpg]

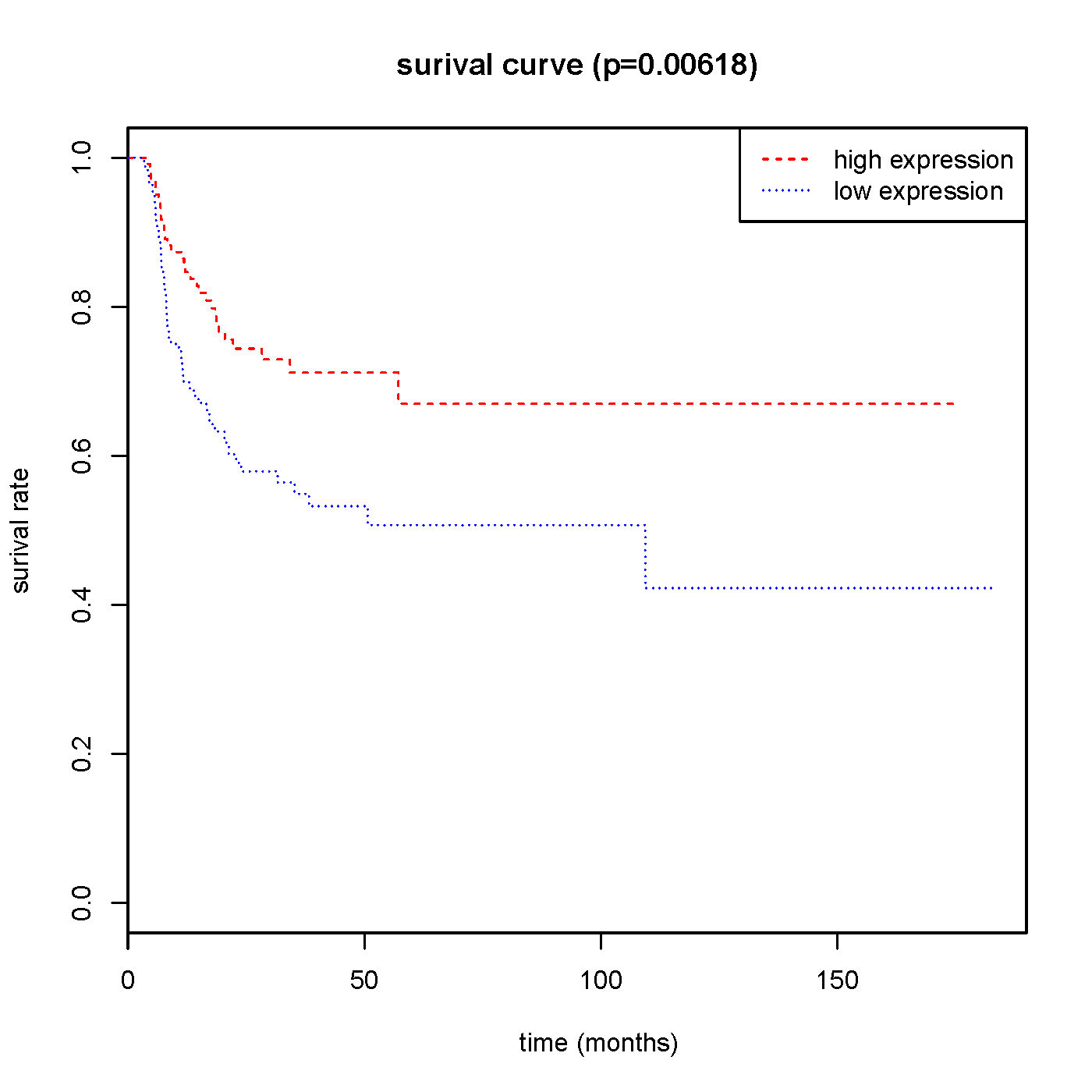

Supplement: Dataset S1 — Kaplan–Meier survival analysis with the log-rank test was used to identify relationships between the above 2493 lncRNA signatures and OSCC patient survival. Then, we determined the levels of 126 lncRNA signatures that were significantly related to DFS. [file peerj-06-5307-s005.zip › The result of Kaplan–Meier survival analyses and log-rank tests for DFS in OSCC/RP11-173C1.1.jpg]

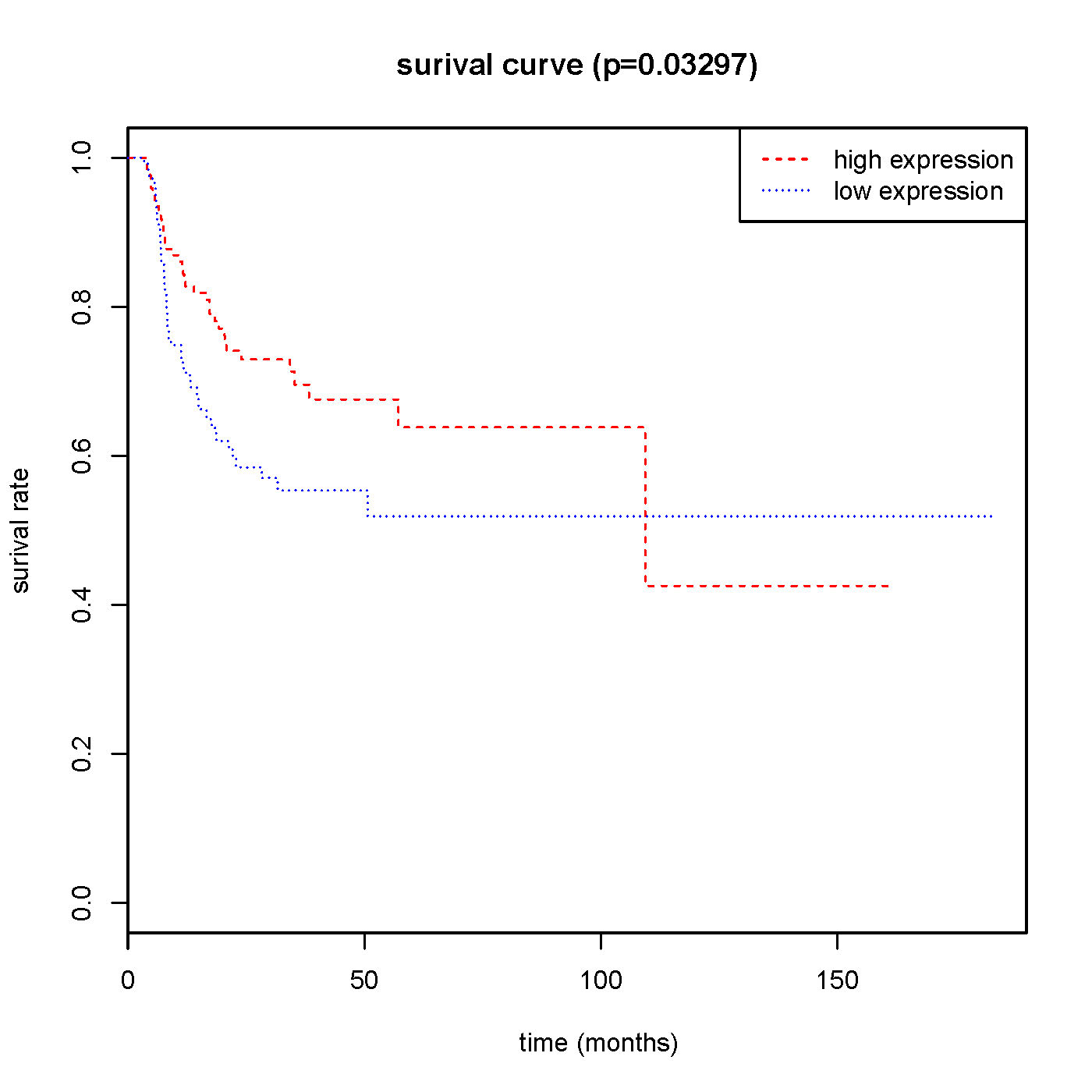

Supplement: Dataset S1 — Kaplan–Meier survival analysis with the log-rank test was used to identify relationships between the above 2493 lncRNA signatures and OSCC patient survival. Then, we determined the levels of 126 lncRNA signatures that were significantly related to DFS. [file peerj-06-5307-s005.zip › The result of Kaplan–Meier survival analyses and log-rank tests for DFS in OSCC/RP11-17A1.3.jpg]

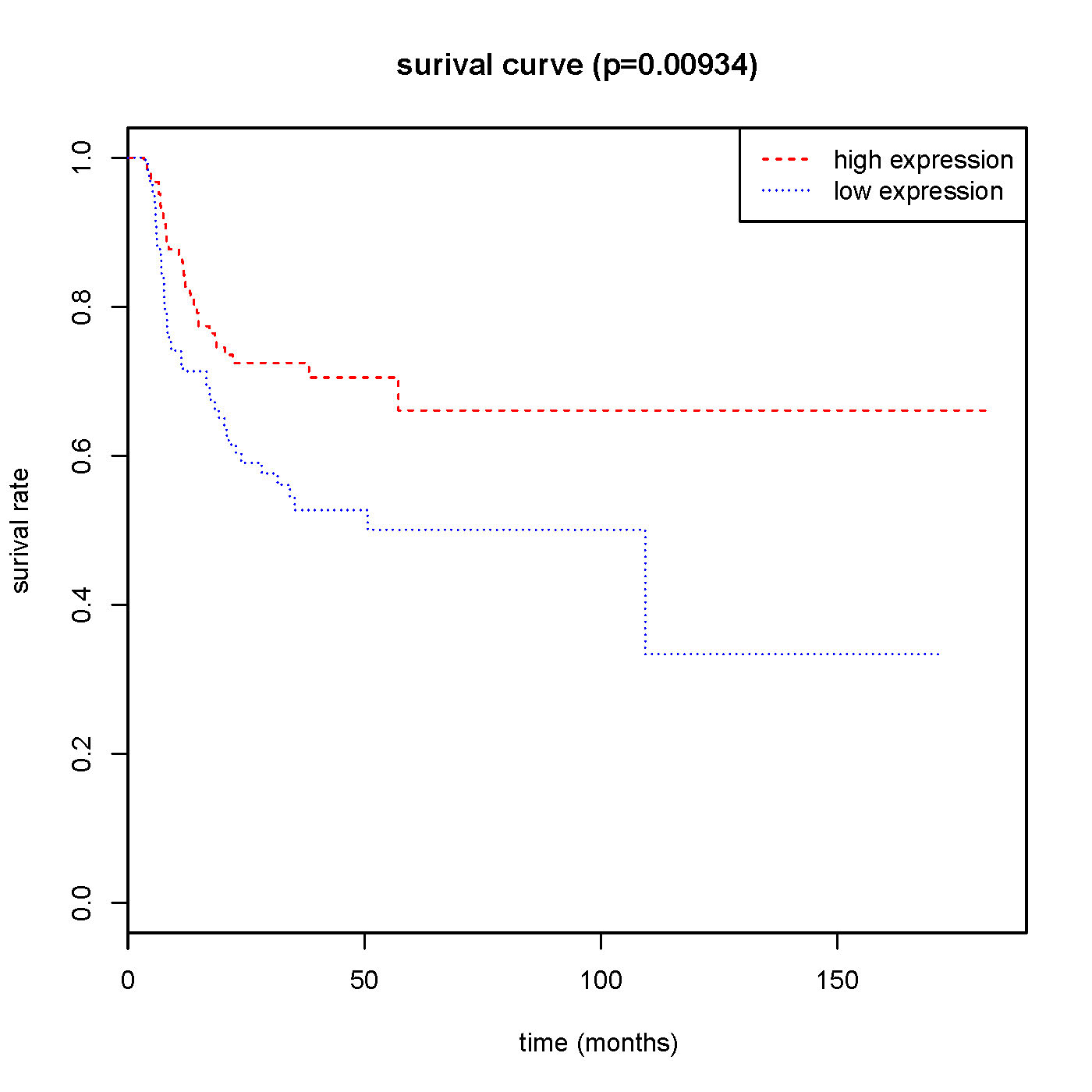

Supplement: Dataset S1 — Kaplan–Meier survival analysis with the log-rank test was used to identify relationships between the above 2493 lncRNA signatures and OSCC patient survival. Then, we determined the levels of 126 lncRNA signatures that were significantly related to DFS. [file peerj-06-5307-s005.zip › The result of Kaplan–Meier survival analyses and log-rank tests for DFS in OSCC/RP11-181E10.3.jpg]

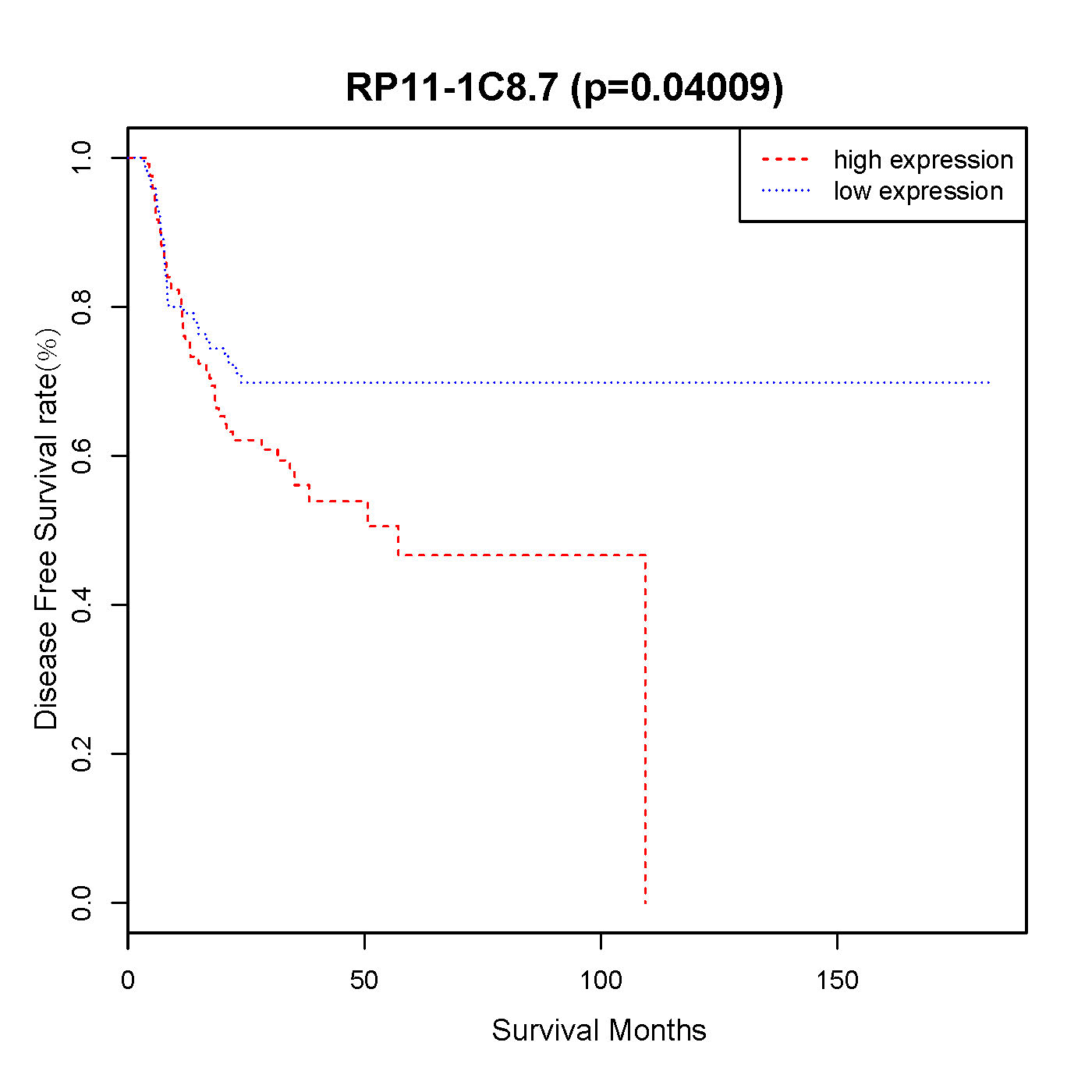

Supplement: Dataset S1 — Kaplan–Meier survival analysis with the log-rank test was used to identify relationships between the above 2493 lncRNA signatures and OSCC patient survival. Then, we determined the levels of 126 lncRNA signatures that were significantly related to DFS. [file peerj-06-5307-s005.zip › The result of Kaplan–Meier survival analyses and log-rank tests for DFS in OSCC/RP11-1C8.7.jpg]

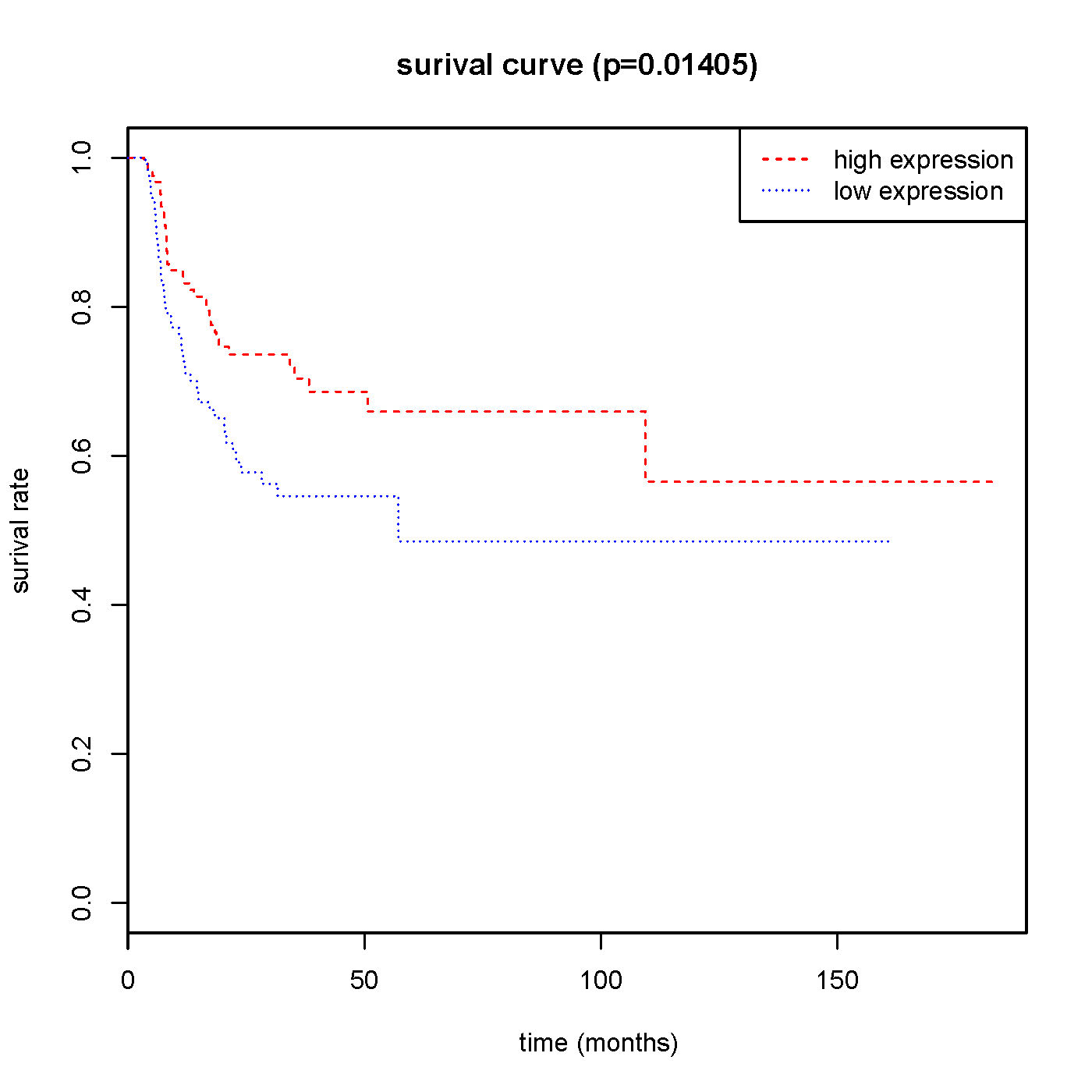

Supplement: Dataset S1 — Kaplan–Meier survival analysis with the log-rank test was used to identify relationships between the above 2493 lncRNA signatures and OSCC patient survival. Then, we determined the levels of 126 lncRNA signatures that were significantly related to DFS. [file peerj-06-5307-s005.zip › The result of Kaplan–Meier survival analyses and log-rank tests for DFS in OSCC/RP11-218E20.3.jpg]

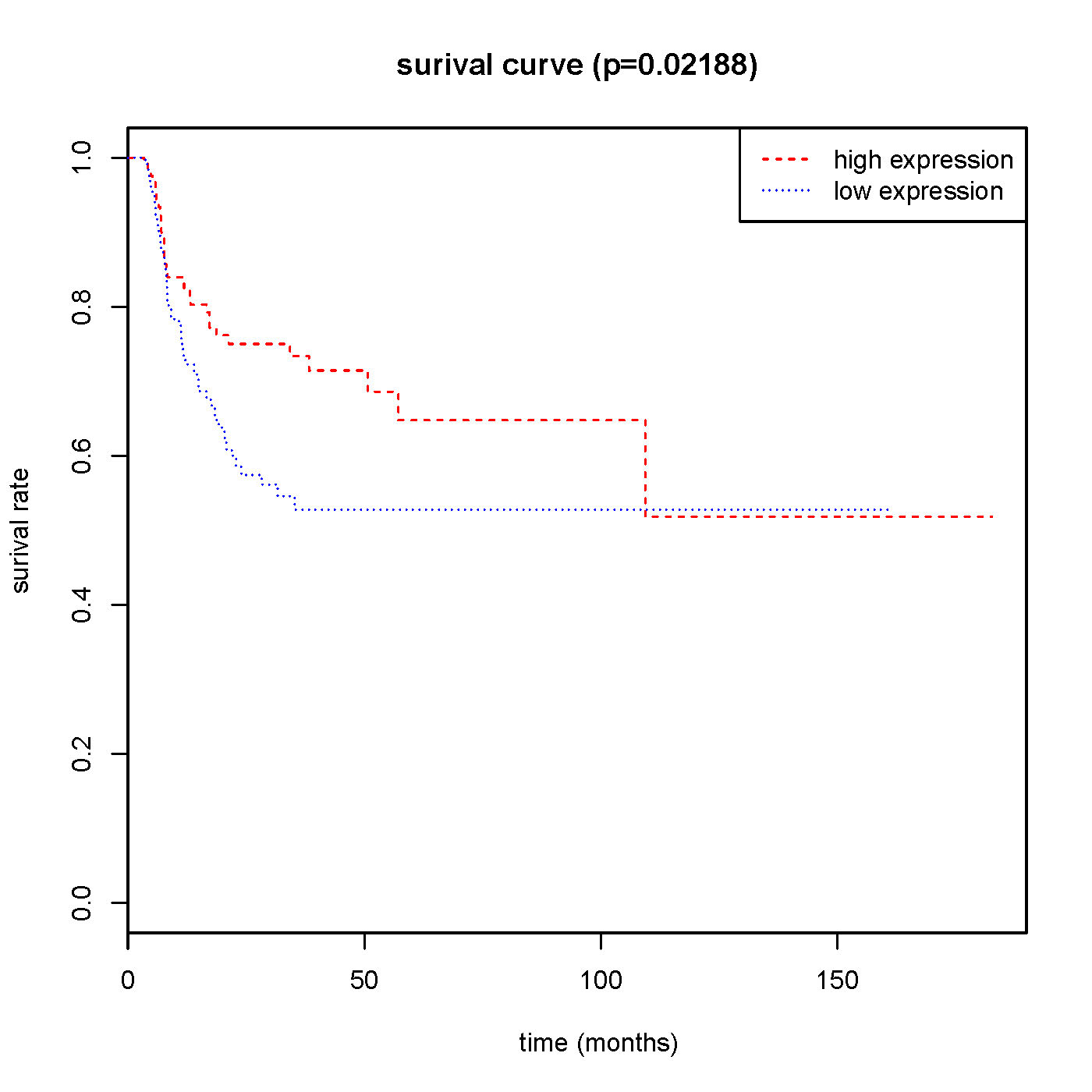

Supplement: Dataset S1 — Kaplan–Meier survival analysis with the log-rank test was used to identify relationships between the above 2493 lncRNA signatures and OSCC patient survival. Then, we determined the levels of 126 lncRNA signatures that were significantly related to DFS. [file peerj-06-5307-s005.zip › The result of Kaplan–Meier survival analyses and log-rank tests for DFS in OSCC/RP11-256L6.3.jpg]

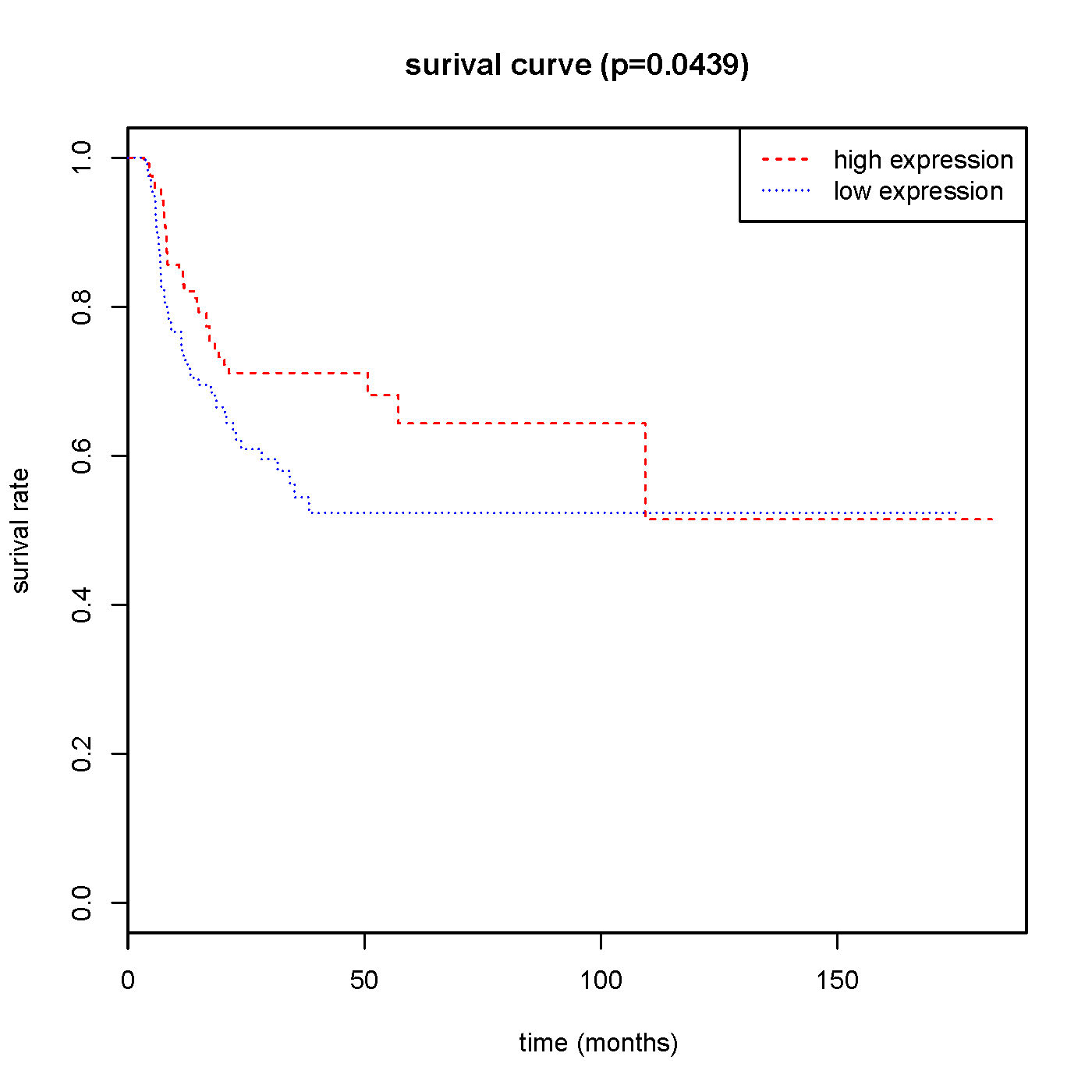

Supplement: Dataset S1 — Kaplan–Meier survival analysis with the log-rank test was used to identify relationships between the above 2493 lncRNA signatures and OSCC patient survival. Then, we determined the levels of 126 lncRNA signatures that were significantly related to DFS. [file peerj-06-5307-s005.zip › The result of Kaplan–Meier survival analyses and log-rank tests for DFS in OSCC/RP11-276H7.2.jpg]

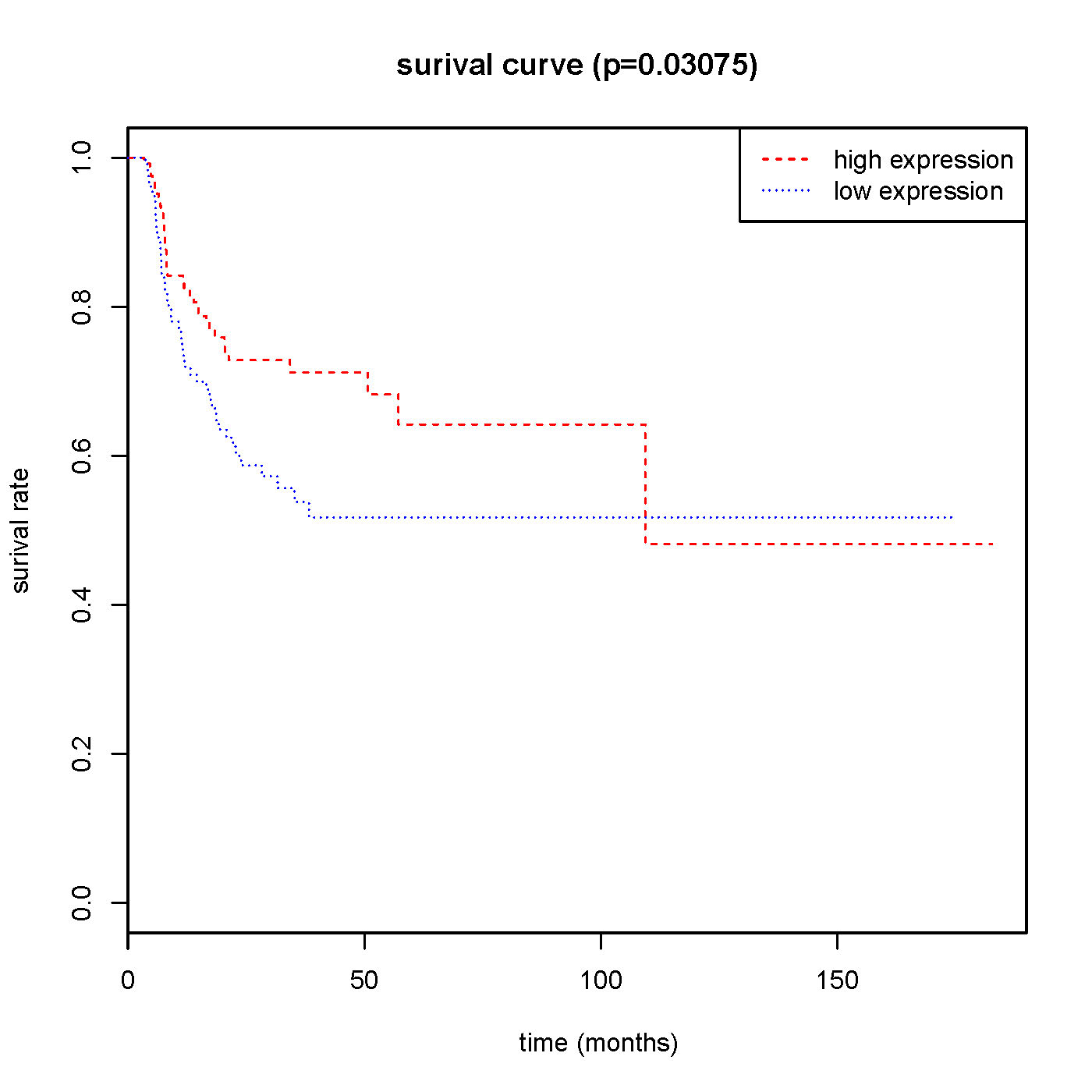

Supplement: Dataset S1 — Kaplan–Meier survival analysis with the log-rank test was used to identify relationships between the above 2493 lncRNA signatures and OSCC patient survival. Then, we determined the levels of 126 lncRNA signatures that were significantly related to DFS. [file peerj-06-5307-s005.zip › The result of Kaplan–Meier survival analyses and log-rank tests for DFS in OSCC/RP11-276H7.3.jpg]

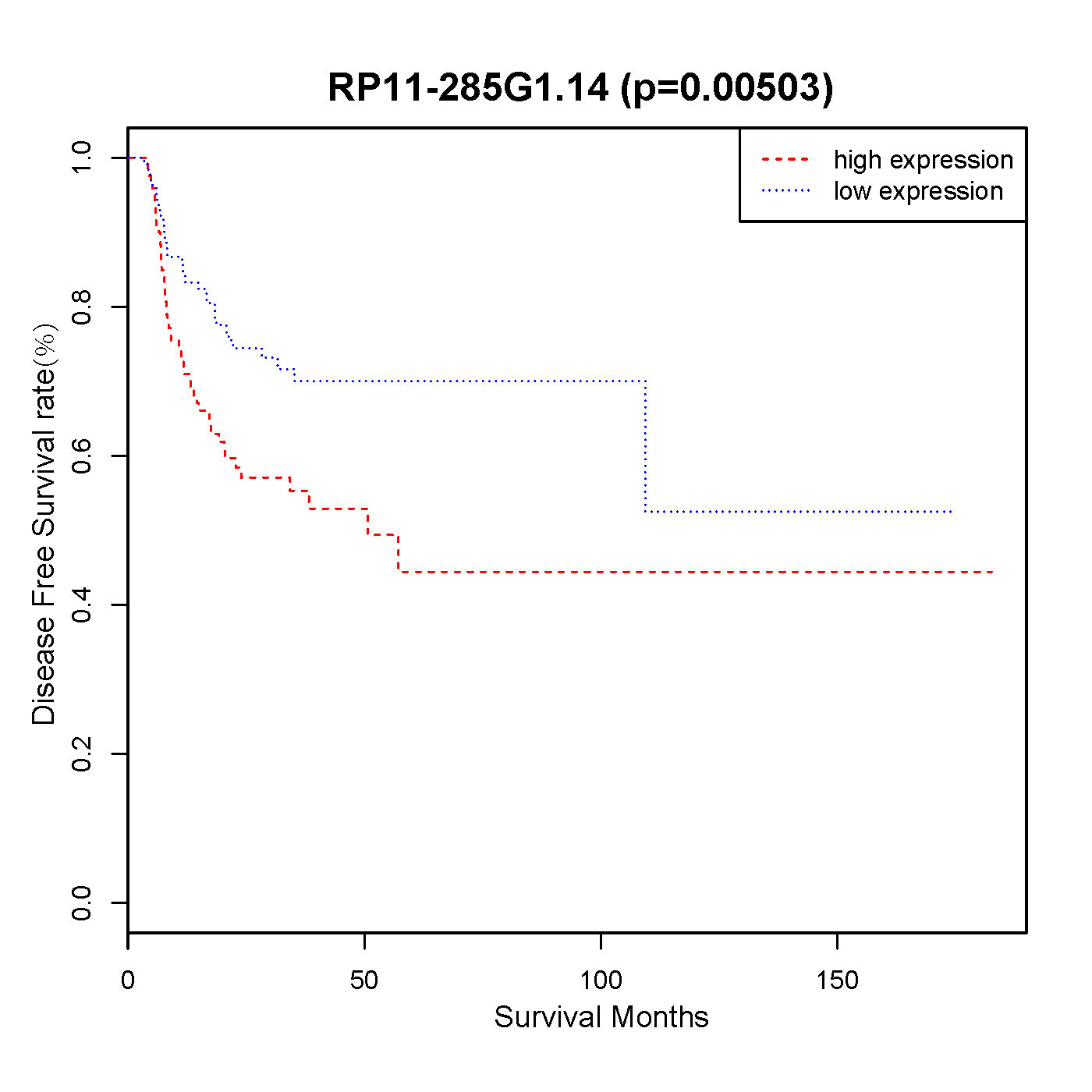

Supplement: Dataset S1 — Kaplan–Meier survival analysis with the log-rank test was used to identify relationships between the above 2493 lncRNA signatures and OSCC patient survival. Then, we determined the levels of 126 lncRNA signatures that were significantly related to DFS. [file peerj-06-5307-s005.zip › The result of Kaplan–Meier survival analyses and log-rank tests for DFS in OSCC/RP11-285G1.14.jpg]

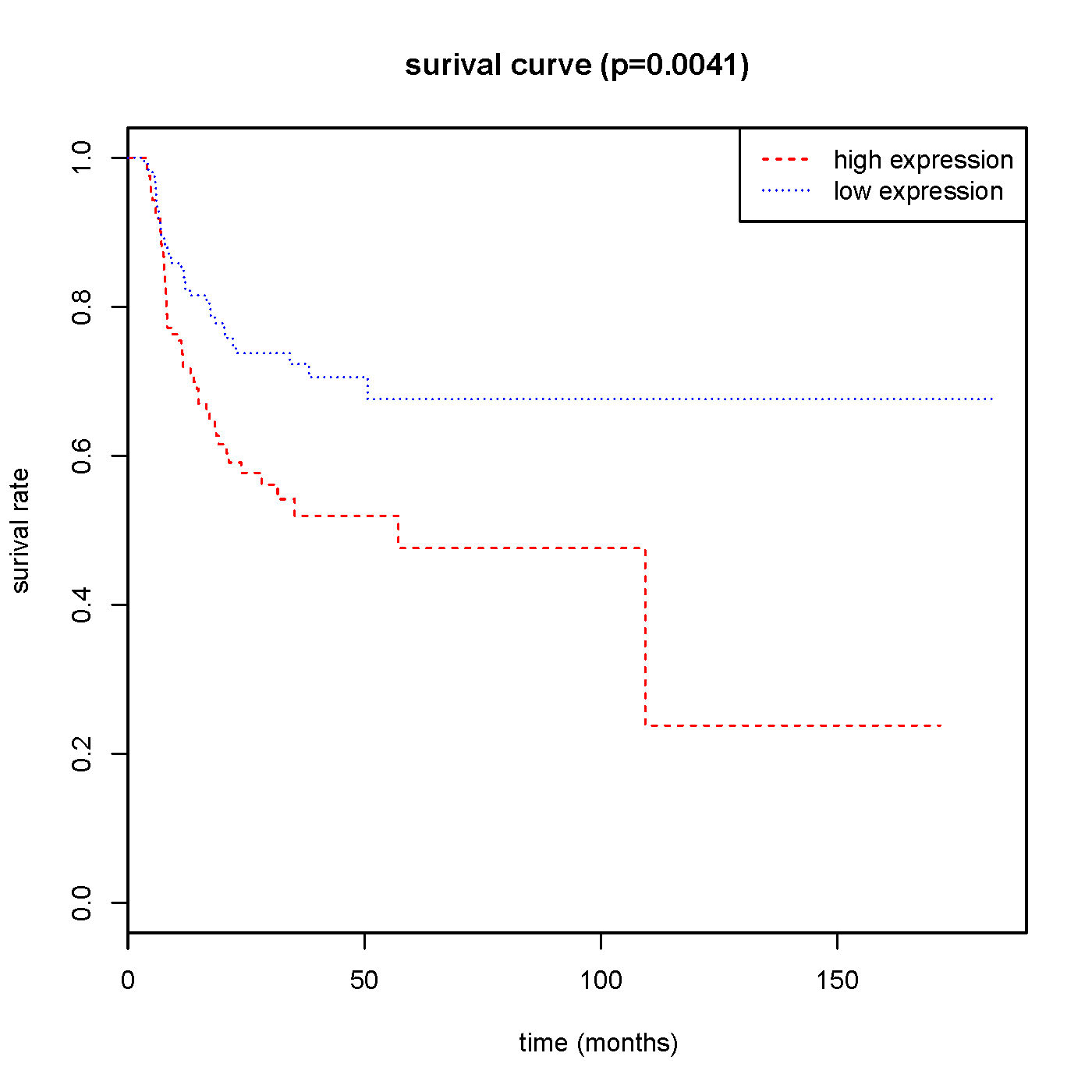

Supplement: Dataset S1 — Kaplan–Meier survival analysis with the log-rank test was used to identify relationships between the above 2493 lncRNA signatures and OSCC patient survival. Then, we determined the levels of 126 lncRNA signatures that were significantly related to DFS. [file peerj-06-5307-s005.zip › The result of Kaplan–Meier survival analyses and log-rank tests for DFS in OSCC/RP11-286E11.1.jpg]

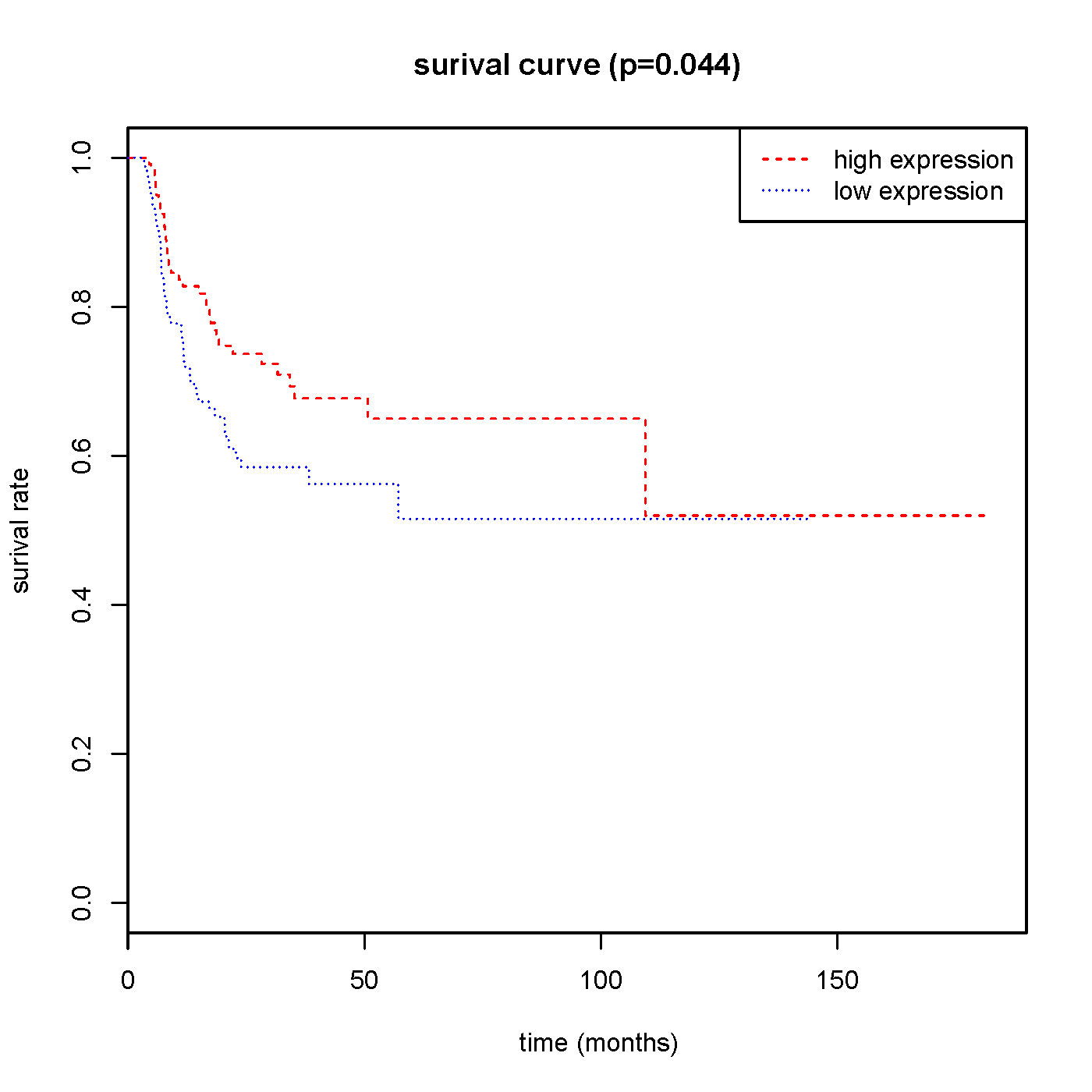

Supplement: Dataset S1 — Kaplan–Meier survival analysis with the log-rank test was used to identify relationships between the above 2493 lncRNA signatures and OSCC patient survival. Then, we determined the levels of 126 lncRNA signatures that were significantly related to DFS. [file peerj-06-5307-s005.zip › The result of Kaplan–Meier survival analyses and log-rank tests for DFS in OSCC/RP11-302L19.3.jpg]

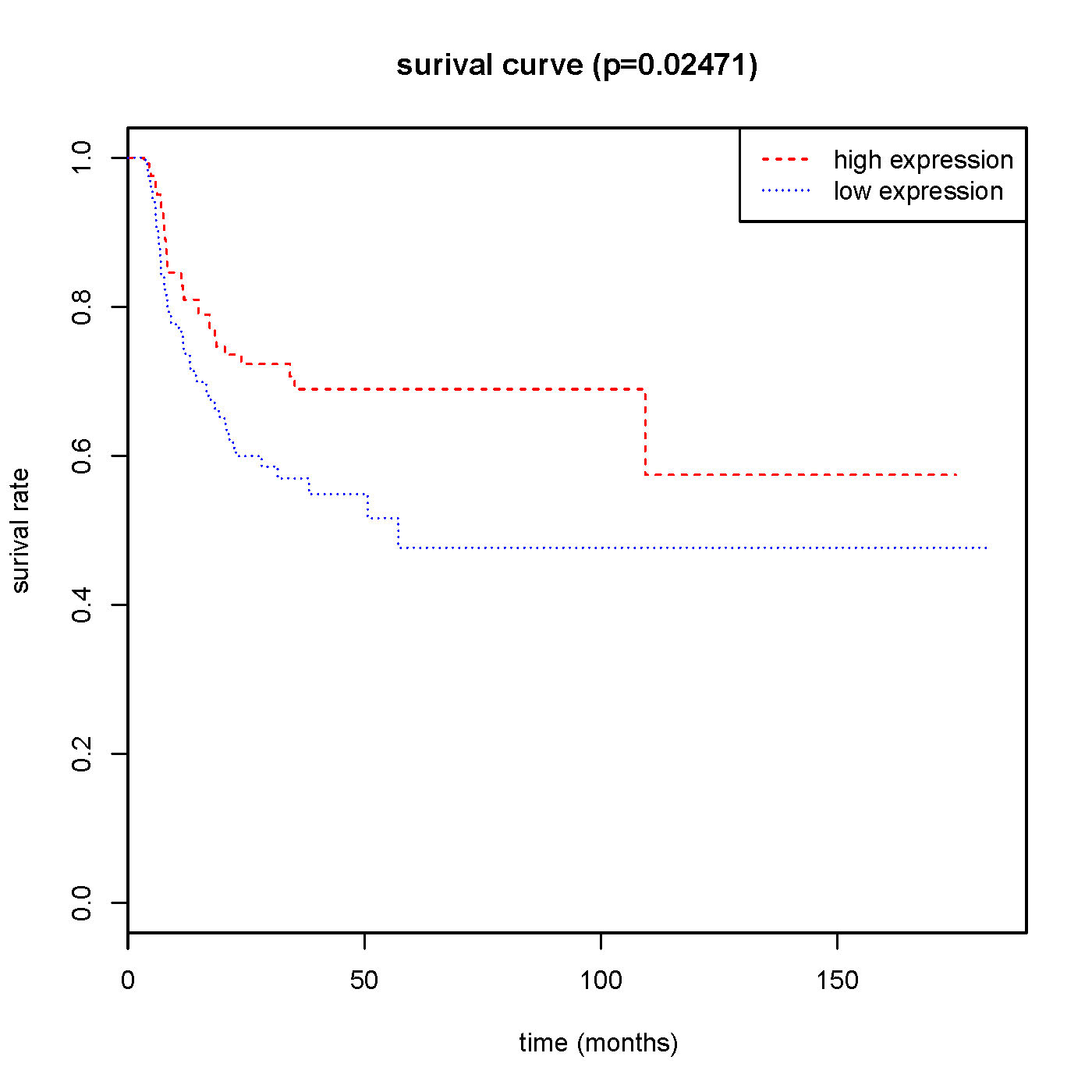

Supplement: Dataset S1 — Kaplan–Meier survival analysis with the log-rank test was used to identify relationships between the above 2493 lncRNA signatures and OSCC patient survival. Then, we determined the levels of 126 lncRNA signatures that were significantly related to DFS. [file peerj-06-5307-s005.zip › The result of Kaplan–Meier survival analyses and log-rank tests for DFS in OSCC/RP11-313E19.2.jpg]

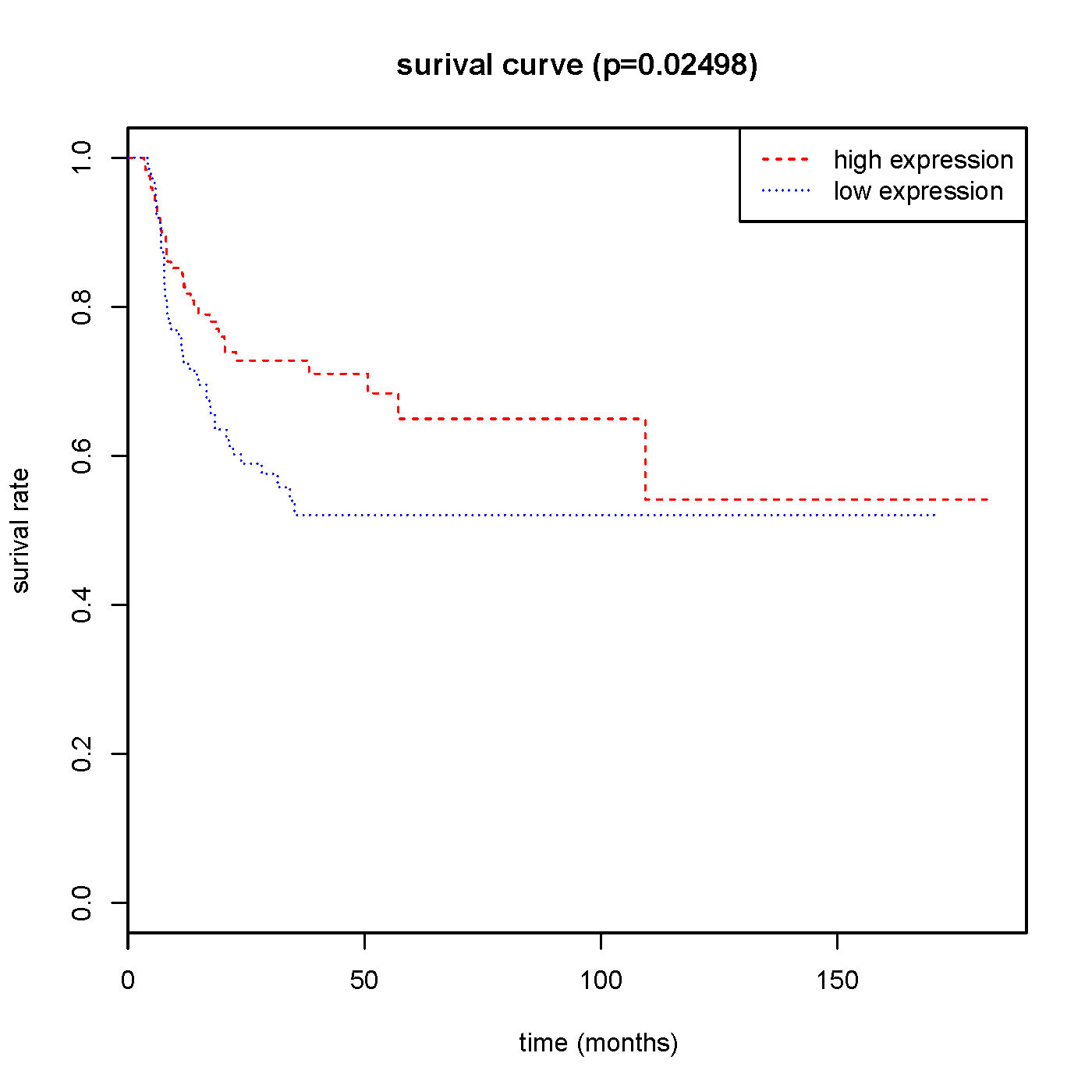

Supplement: Dataset S1 — Kaplan–Meier survival analysis with the log-rank test was used to identify relationships between the above 2493 lncRNA signatures and OSCC patient survival. Then, we determined the levels of 126 lncRNA signatures that were significantly related to DFS. [file peerj-06-5307-s005.zip › The result of Kaplan–Meier survival analyses and log-rank tests for DFS in OSCC/RP11-320G24.1.jpg]

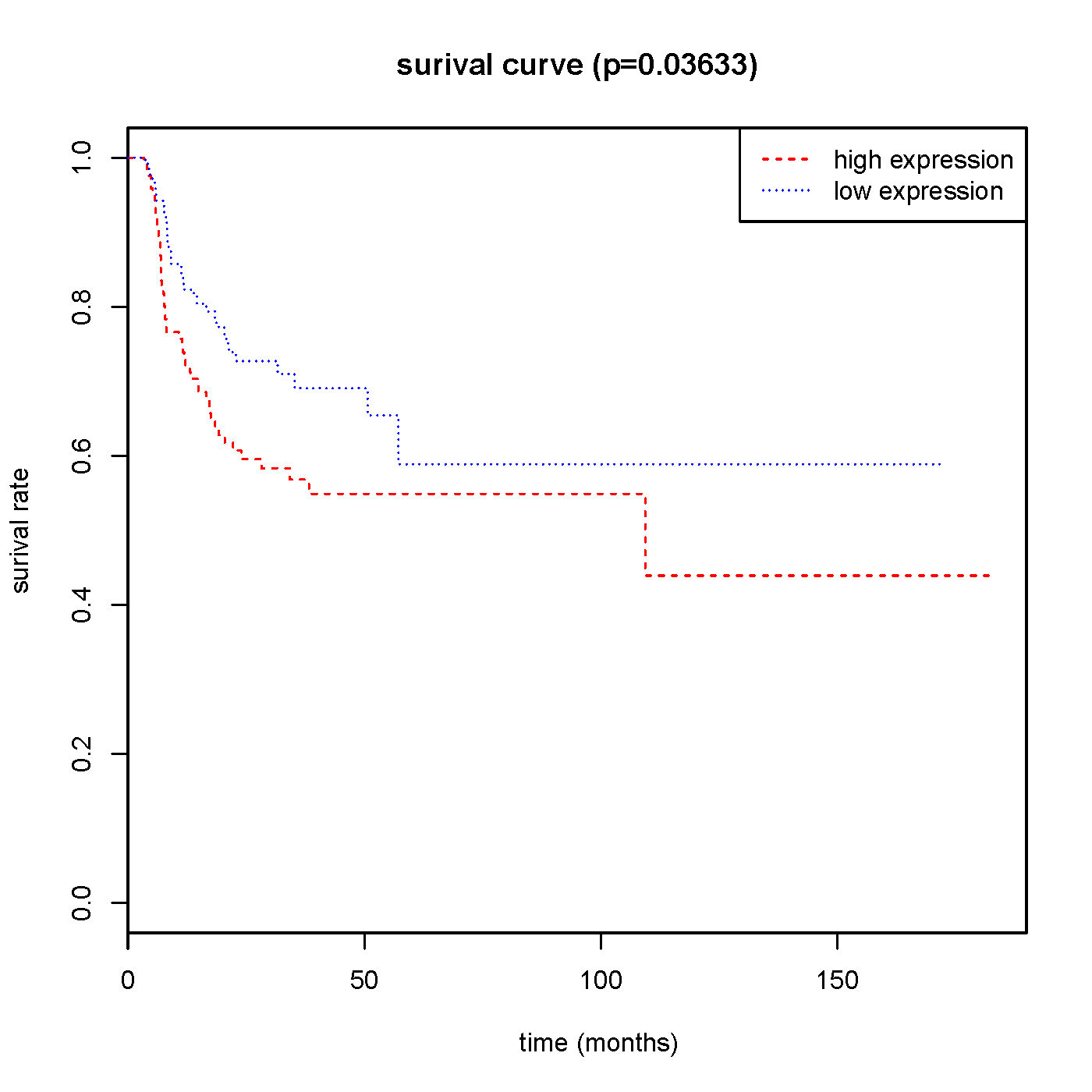

Supplement: Dataset S1 — Kaplan–Meier survival analysis with the log-rank test was used to identify relationships between the above 2493 lncRNA signatures and OSCC patient survival. Then, we determined the levels of 126 lncRNA signatures that were significantly related to DFS. [file peerj-06-5307-s005.zip › The result of Kaplan–Meier survival analyses and log-rank tests for DFS in OSCC/RP11-321P16.1.jpg]

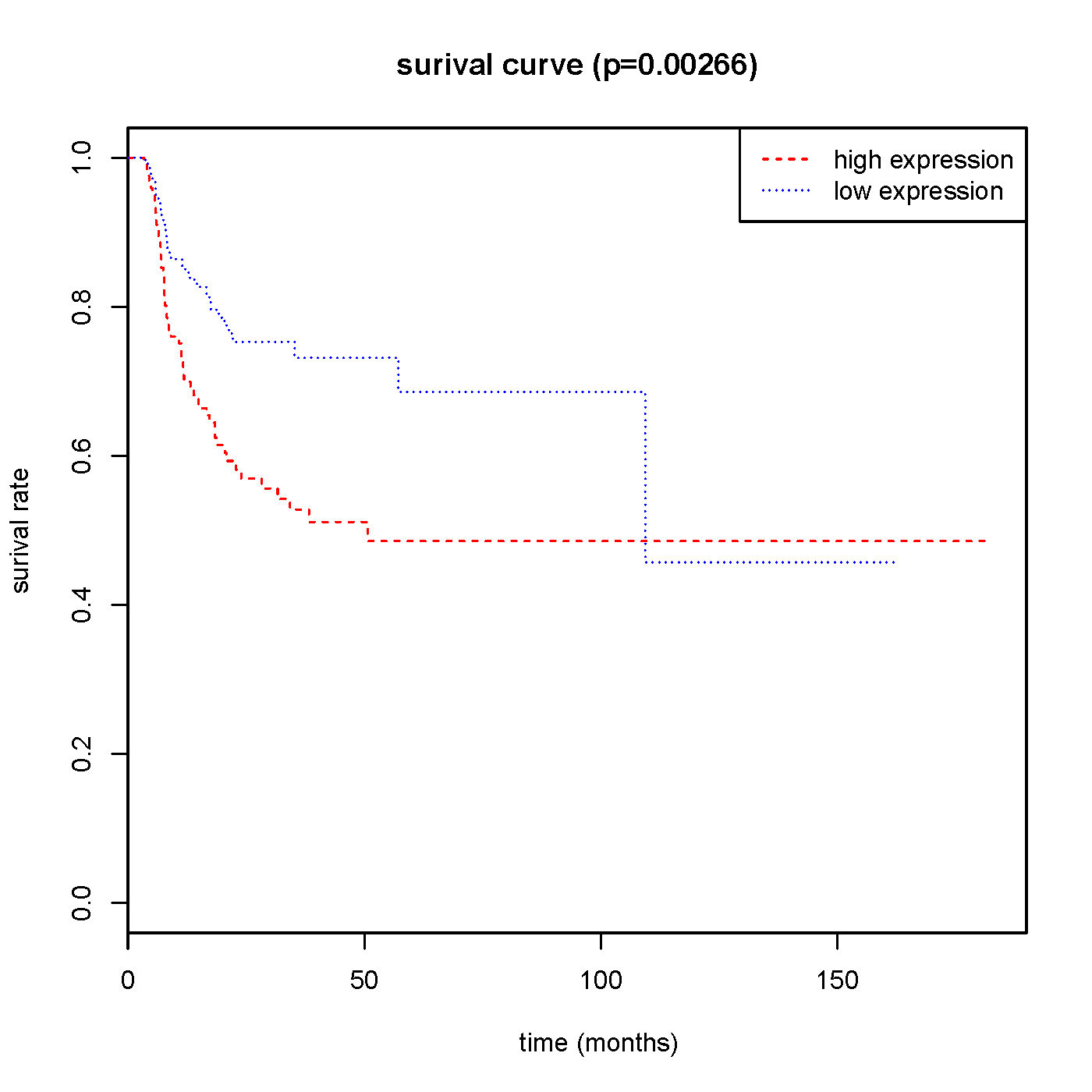

Supplement: Dataset S1 — Kaplan–Meier survival analysis with the log-rank test was used to identify relationships between the above 2493 lncRNA signatures and OSCC patient survival. Then, we determined the levels of 126 lncRNA signatures that were significantly related to DFS. [file peerj-06-5307-s005.zip › The result of Kaplan–Meier survival analyses and log-rank tests for DFS in OSCC/RP11-325N19.3.jpg]

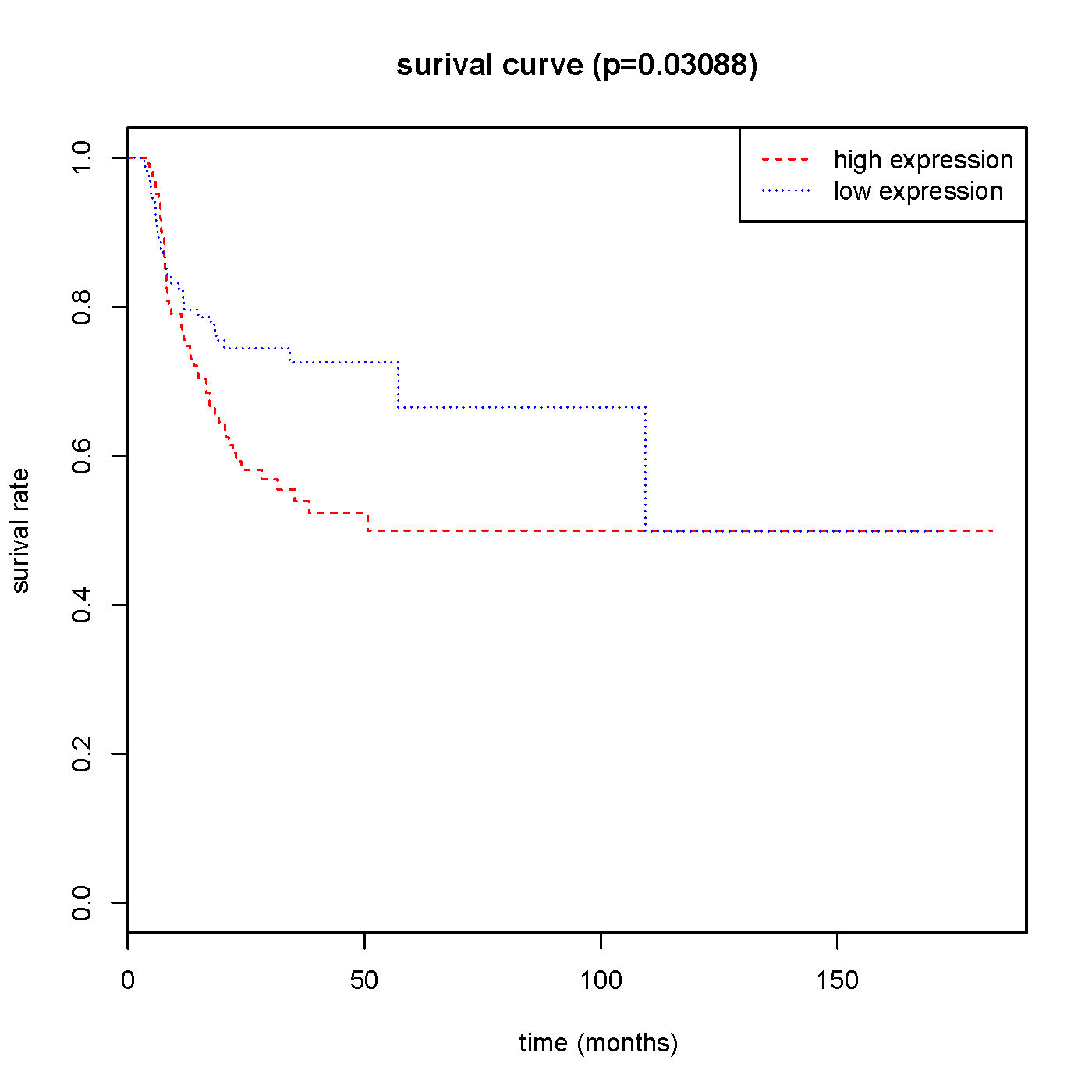

Supplement: Dataset S1 — Kaplan–Meier survival analysis with the log-rank test was used to identify relationships between the above 2493 lncRNA signatures and OSCC patient survival. Then, we determined the levels of 126 lncRNA signatures that were significantly related to DFS. [file peerj-06-5307-s005.zip › The result of Kaplan–Meier survival analyses and log-rank tests for DFS in OSCC/RP11-339B21.10.jpg]

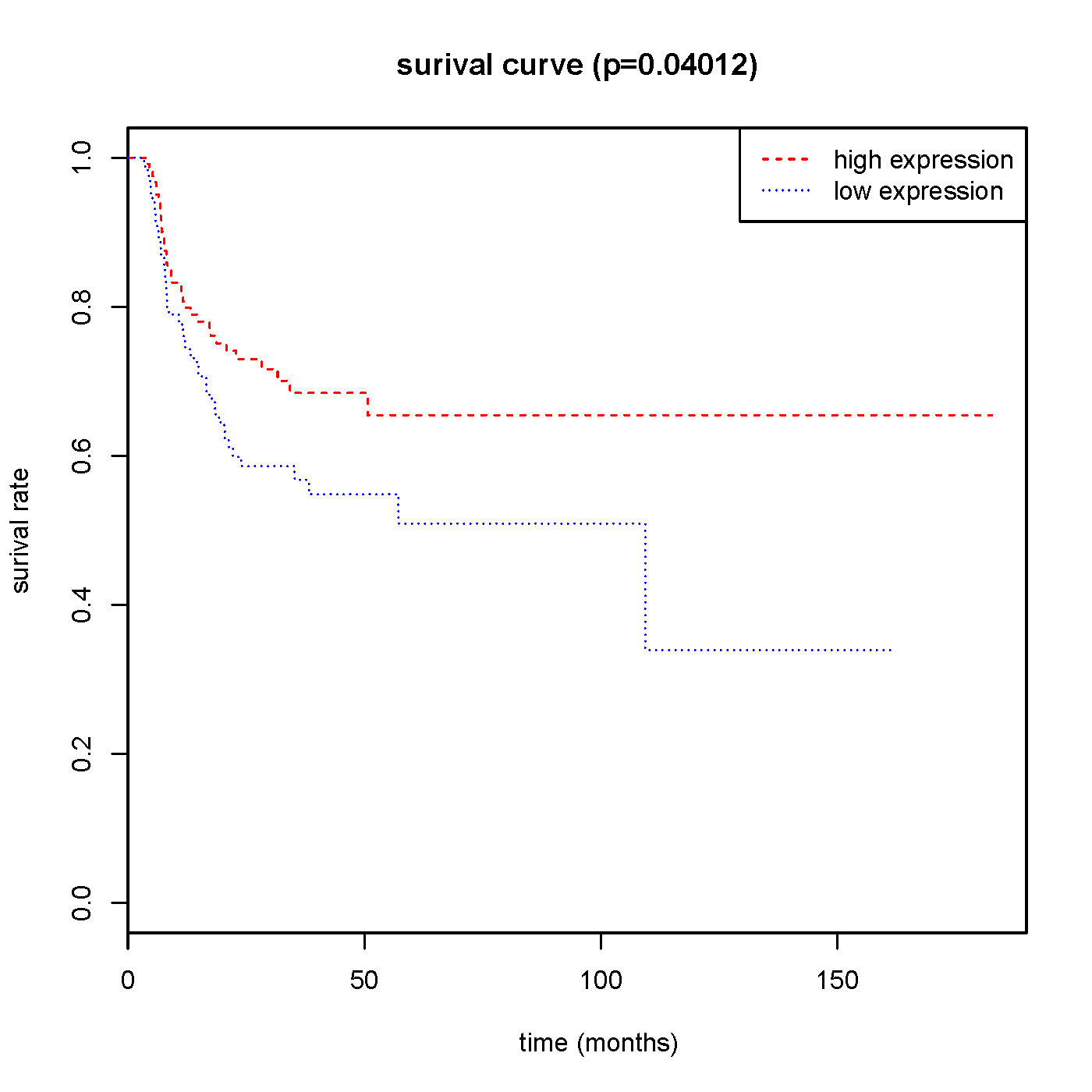

Supplement: Dataset S1 — Kaplan–Meier survival analysis with the log-rank test was used to identify relationships between the above 2493 lncRNA signatures and OSCC patient survival. Then, we determined the levels of 126 lncRNA signatures that were significantly related to DFS. [file peerj-06-5307-s005.zip › The result of Kaplan–Meier survival analyses and log-rank tests for DFS in OSCC/RP11-348J12.5.jpg]

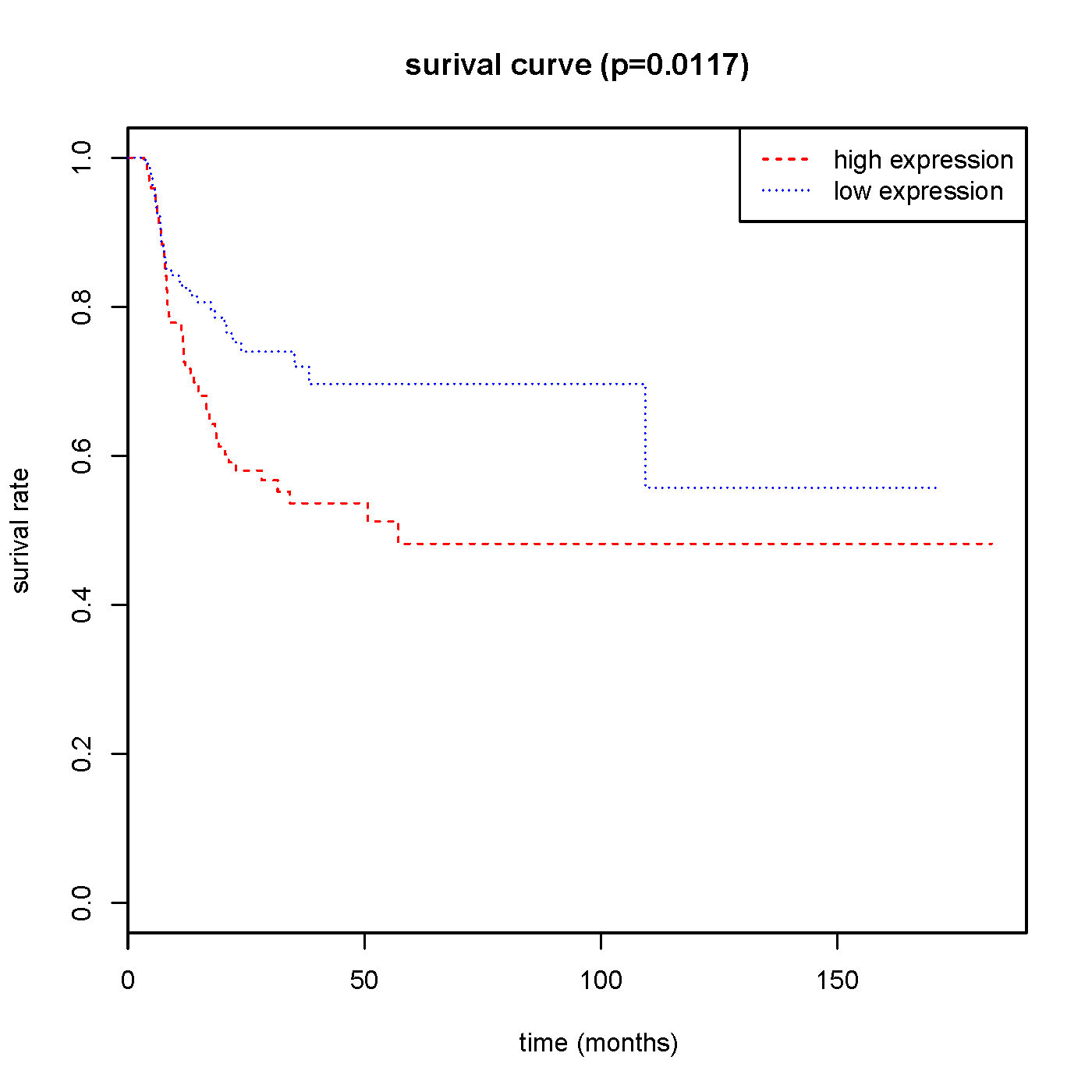

Supplement: Dataset S1 — Kaplan–Meier survival analysis with the log-rank test was used to identify relationships between the above 2493 lncRNA signatures and OSCC patient survival. Then, we determined the levels of 126 lncRNA signatures that were significantly related to DFS. [file peerj-06-5307-s005.zip › The result of Kaplan–Meier survival analyses and log-rank tests for DFS in OSCC/RP11-38M8.1.jpg]

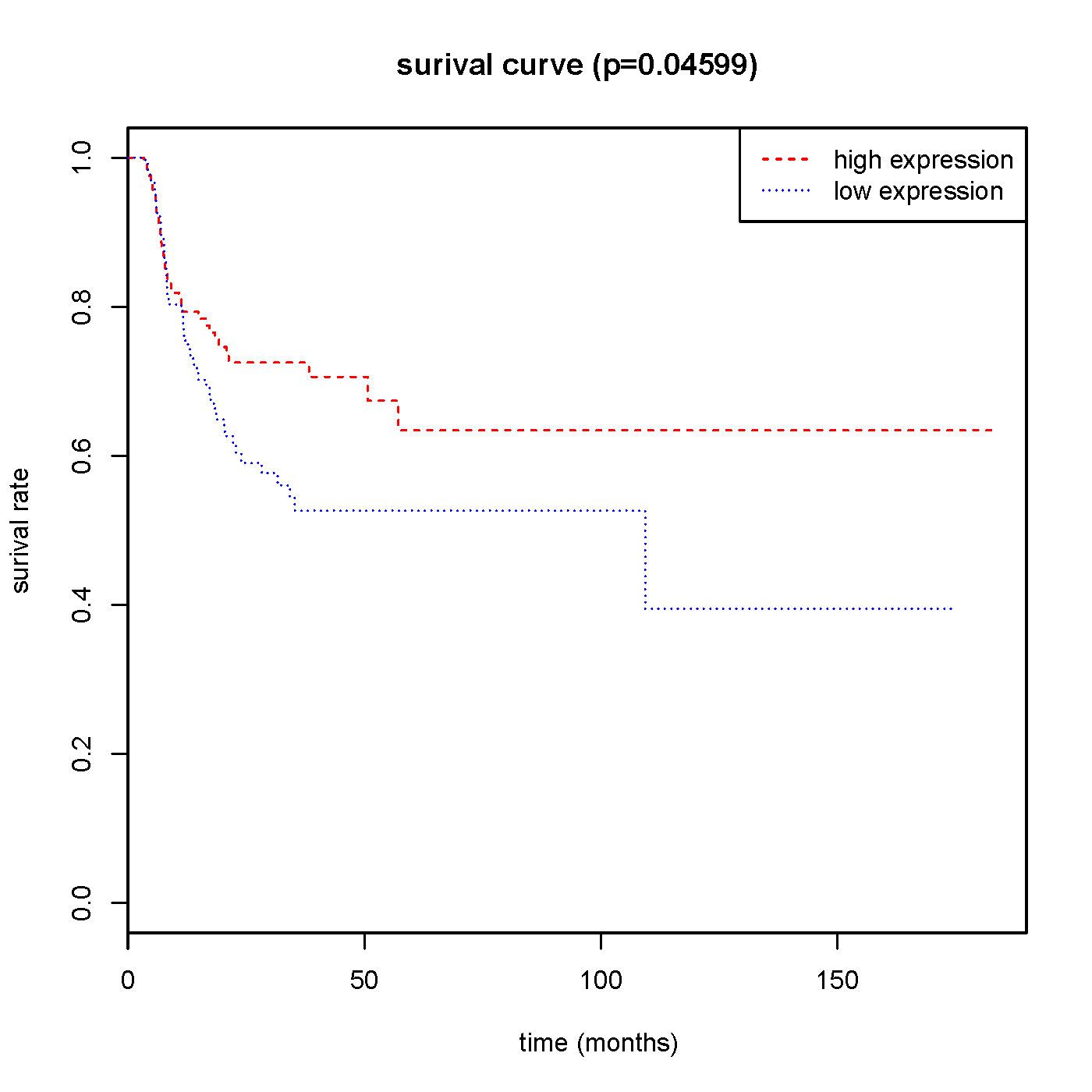

Supplement: Dataset S1 — Kaplan–Meier survival analysis with the log-rank test was used to identify relationships between the above 2493 lncRNA signatures and OSCC patient survival. Then, we determined the levels of 126 lncRNA signatures that were significantly related to DFS. [file peerj-06-5307-s005.zip › The result of Kaplan–Meier survival analyses and log-rank tests for DFS in OSCC/RP11-419C23.1.jpg]

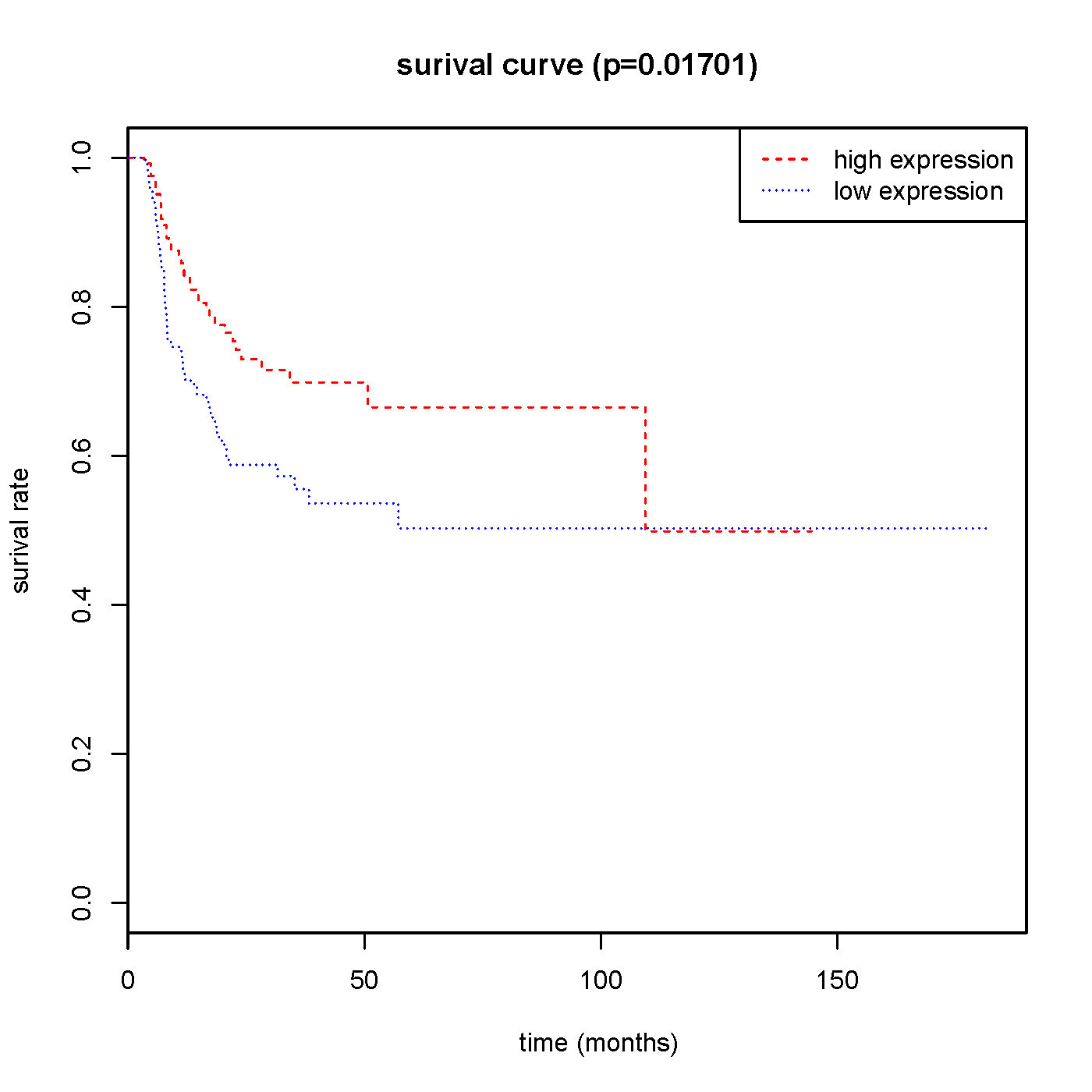

Supplement: Dataset S1 — Kaplan–Meier survival analysis with the log-rank test was used to identify relationships between the above 2493 lncRNA signatures and OSCC patient survival. Then, we determined the levels of 126 lncRNA signatures that were significantly related to DFS. [file peerj-06-5307-s005.zip › The result of Kaplan–Meier survival analyses and log-rank tests for DFS in OSCC/RP11-426C22.6.jpg]

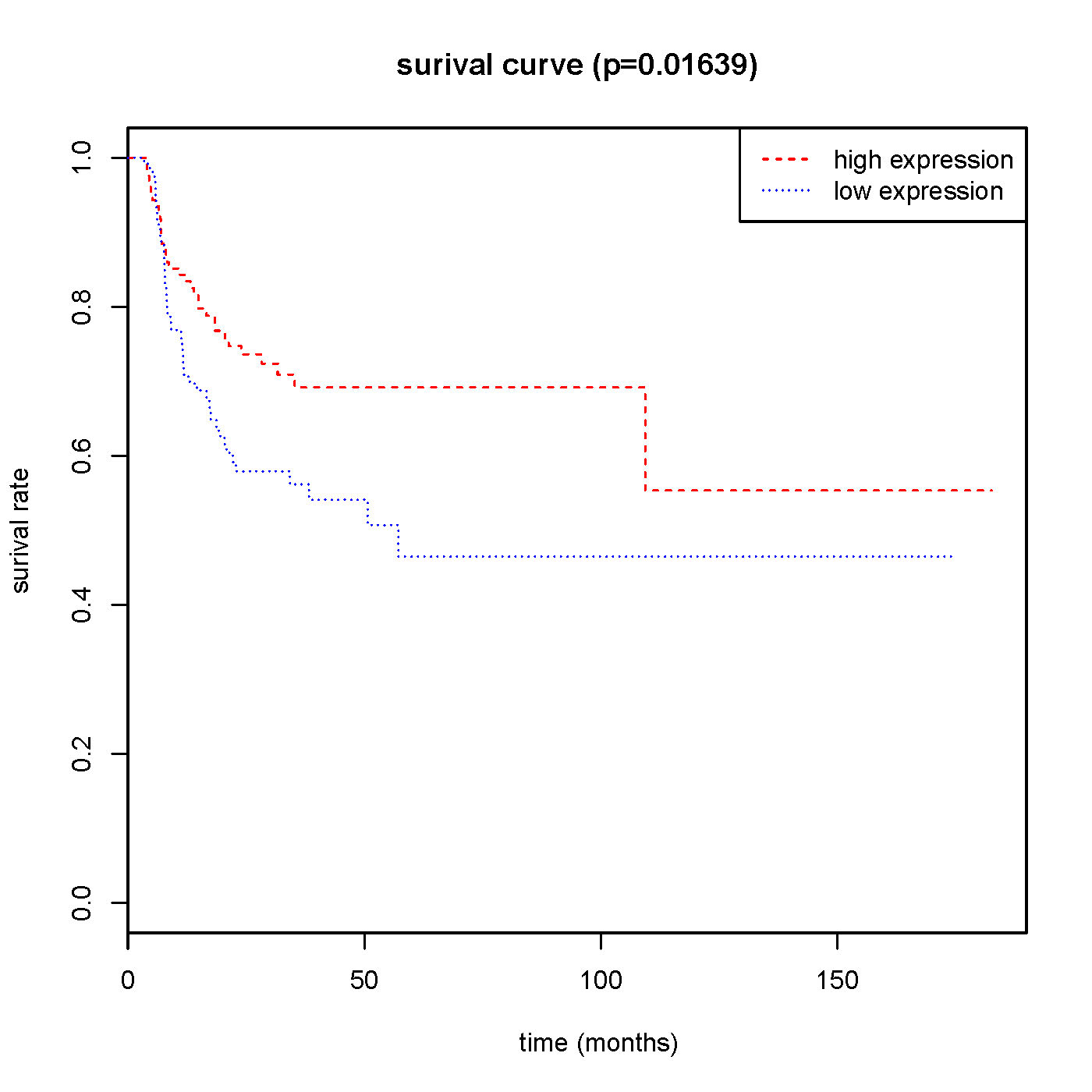

Supplement: Dataset S1 — Kaplan–Meier survival analysis with the log-rank test was used to identify relationships between the above 2493 lncRNA signatures and OSCC patient survival. Then, we determined the levels of 126 lncRNA signatures that were significantly related to DFS. [file peerj-06-5307-s005.zip › The result of Kaplan–Meier survival analyses and log-rank tests for DFS in OSCC/RP11-431J24.2.jpg]

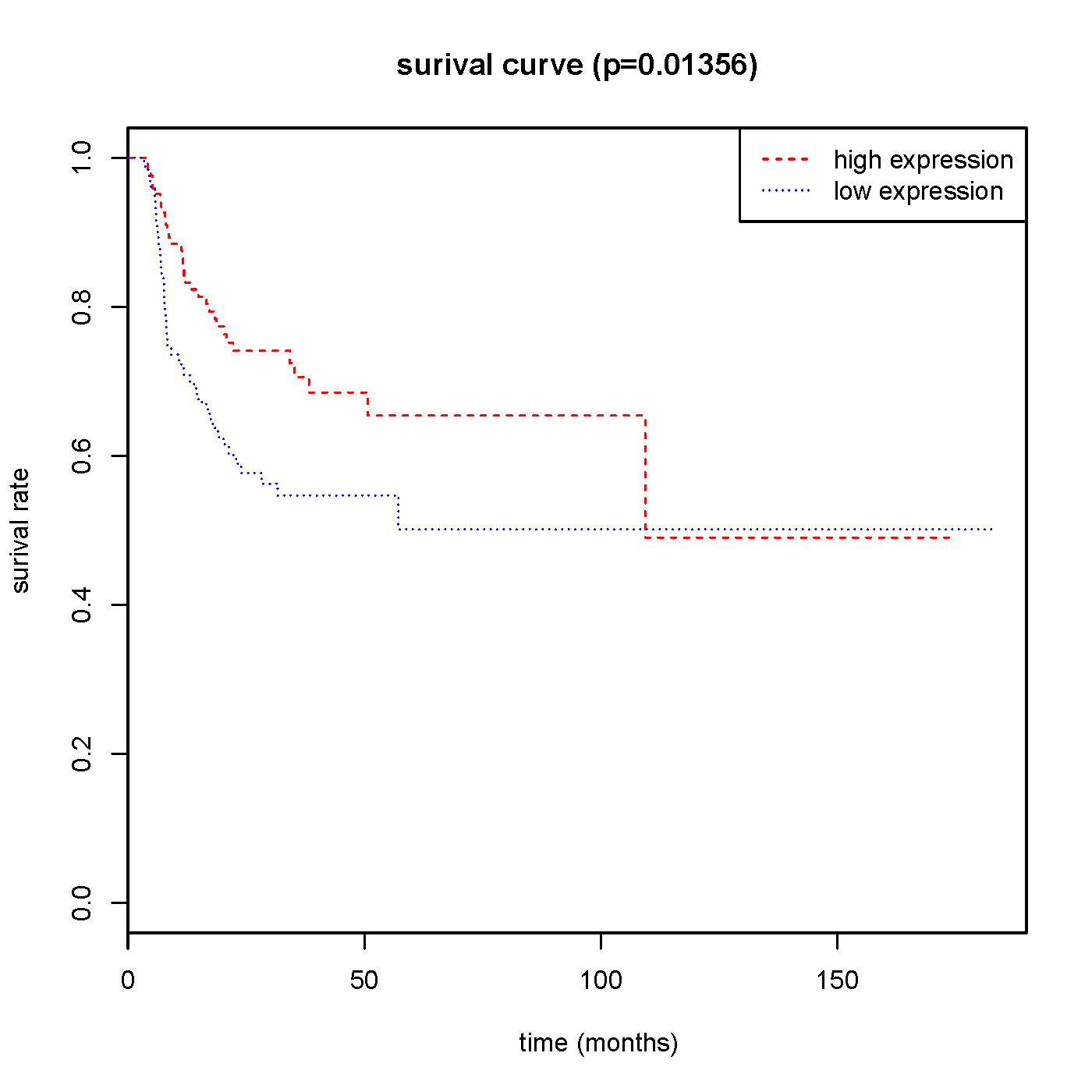

Supplement: Dataset S1 — Kaplan–Meier survival analysis with the log-rank test was used to identify relationships between the above 2493 lncRNA signatures and OSCC patient survival. Then, we determined the levels of 126 lncRNA signatures that were significantly related to DFS. [file peerj-06-5307-s005.zip › The result of Kaplan–Meier survival analyses and log-rank tests for DFS in OSCC/RP11-434D9.2.jpg]

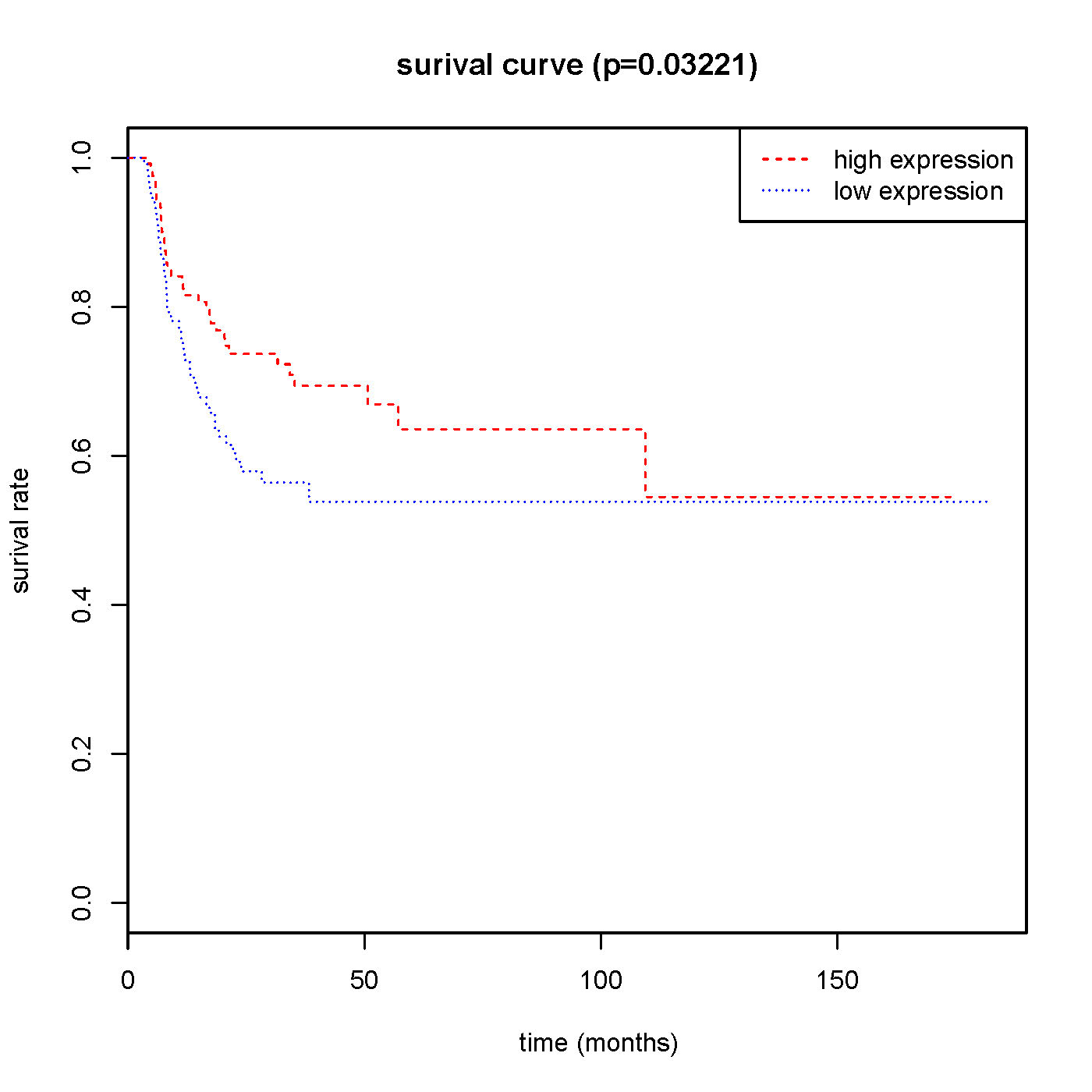

Supplement: Dataset S1 — Kaplan–Meier survival analysis with the log-rank test was used to identify relationships between the above 2493 lncRNA signatures and OSCC patient survival. Then, we determined the levels of 126 lncRNA signatures that were significantly related to DFS. [file peerj-06-5307-s005.zip › The result of Kaplan–Meier survival analyses and log-rank tests for DFS in OSCC/RP11-464F9.21.jpg]

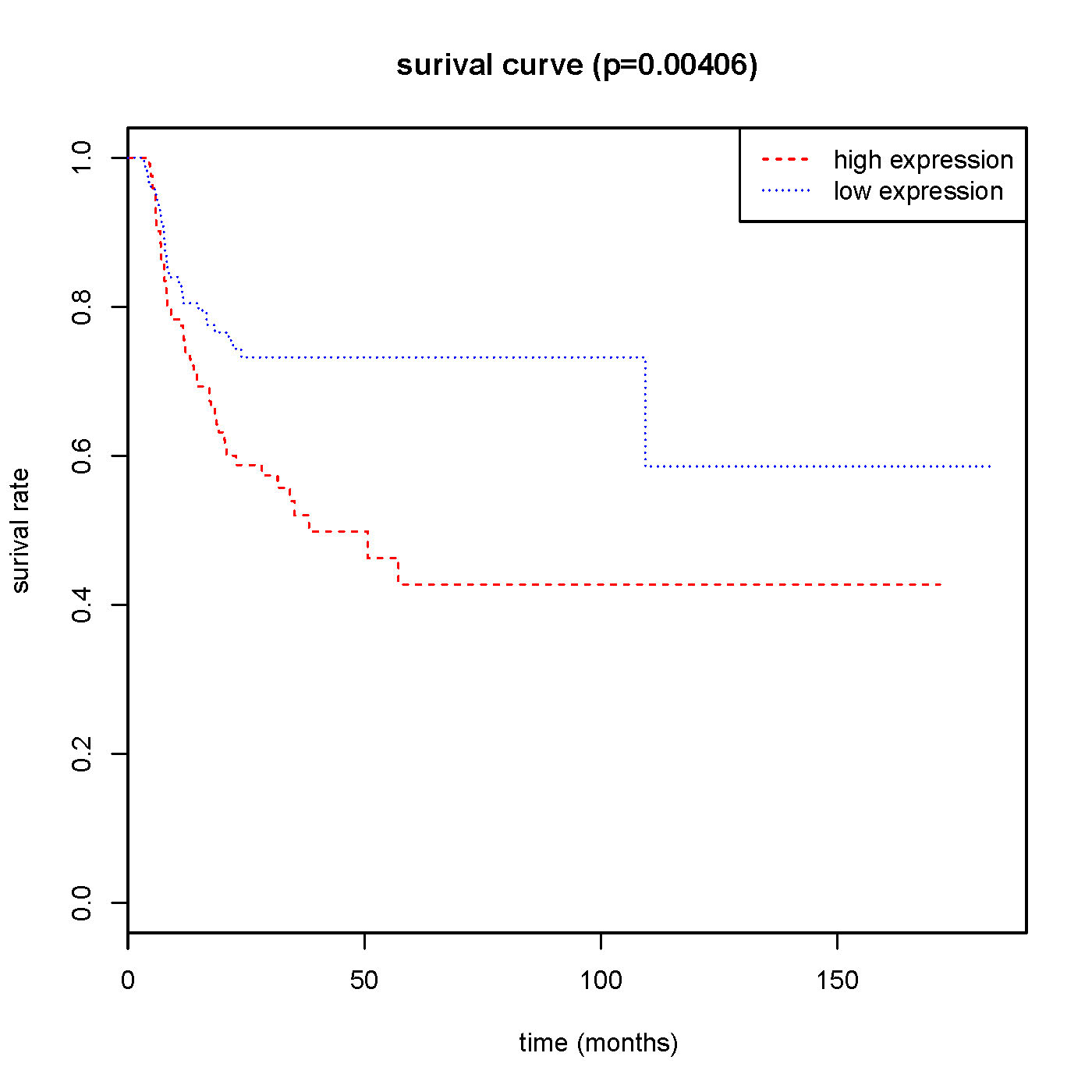

Supplement: Dataset S1 — Kaplan–Meier survival analysis with the log-rank test was used to identify relationships between the above 2493 lncRNA signatures and OSCC patient survival. Then, we determined the levels of 126 lncRNA signatures that were significantly related to DFS. [file peerj-06-5307-s005.zip › The result of Kaplan–Meier survival analyses and log-rank tests for DFS in OSCC/RP11-474N24.6.jpg]

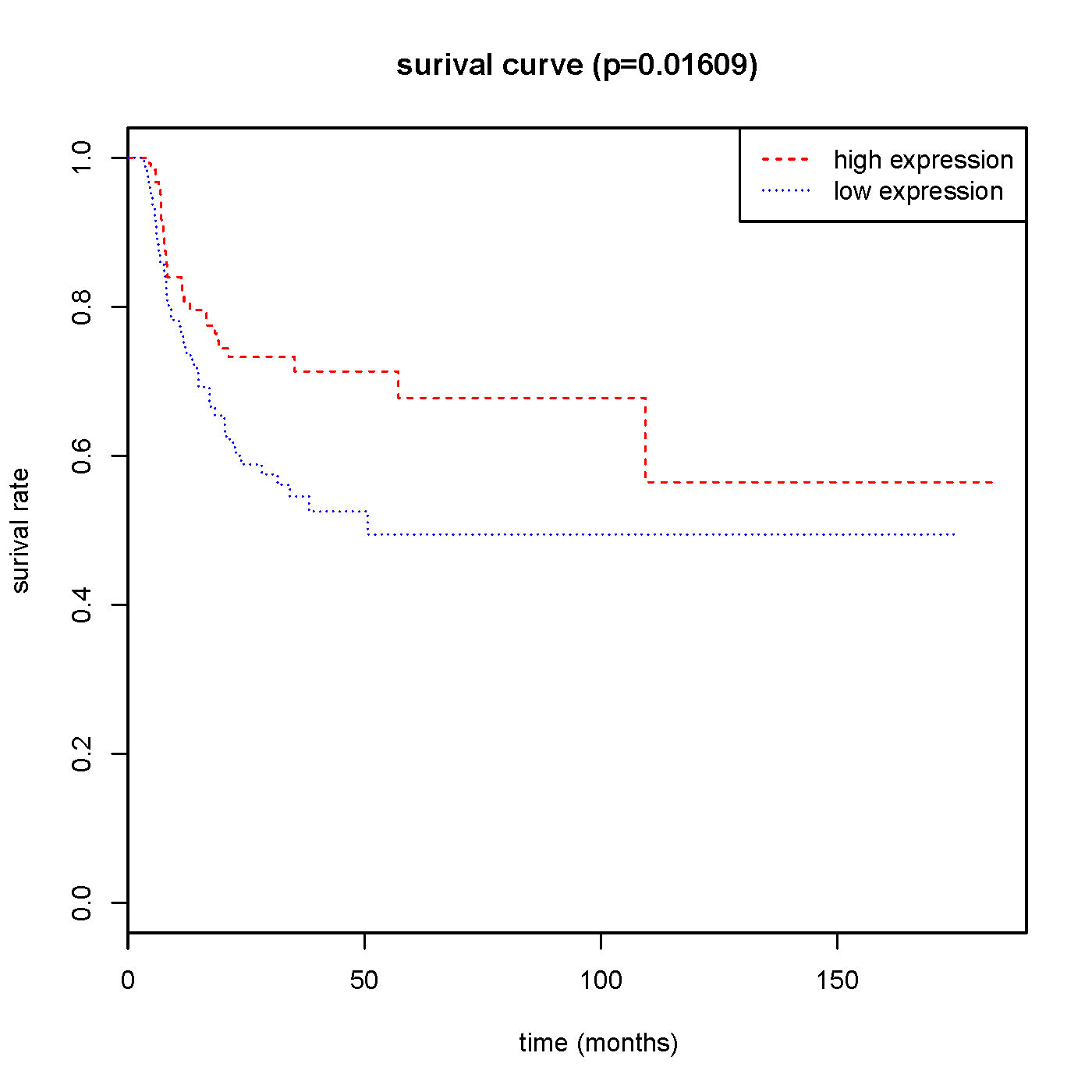

Supplement: Dataset S1 — Kaplan–Meier survival analysis with the log-rank test was used to identify relationships between the above 2493 lncRNA signatures and OSCC patient survival. Then, we determined the levels of 126 lncRNA signatures that were significantly related to DFS. [file peerj-06-5307-s005.zip › The result of Kaplan–Meier survival analyses and log-rank tests for DFS in OSCC/RP11-497D6.3.jpg]

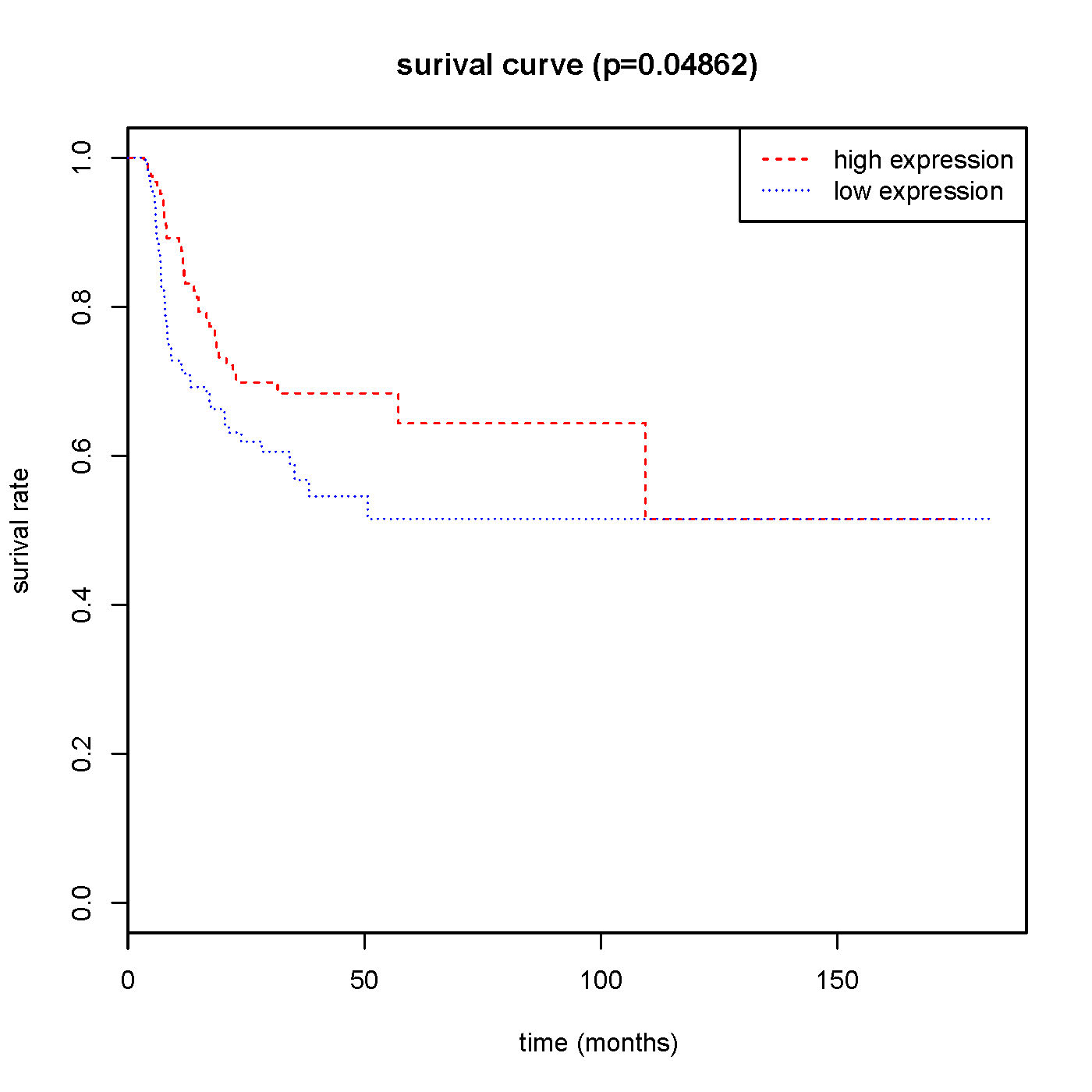

Supplement: Dataset S1 — Kaplan–Meier survival analysis with the log-rank test was used to identify relationships between the above 2493 lncRNA signatures and OSCC patient survival. Then, we determined the levels of 126 lncRNA signatures that were significantly related to DFS. [file peerj-06-5307-s005.zip › The result of Kaplan–Meier survival analyses and log-rank tests for DFS in OSCC/RP11-529G21.2.jpg]

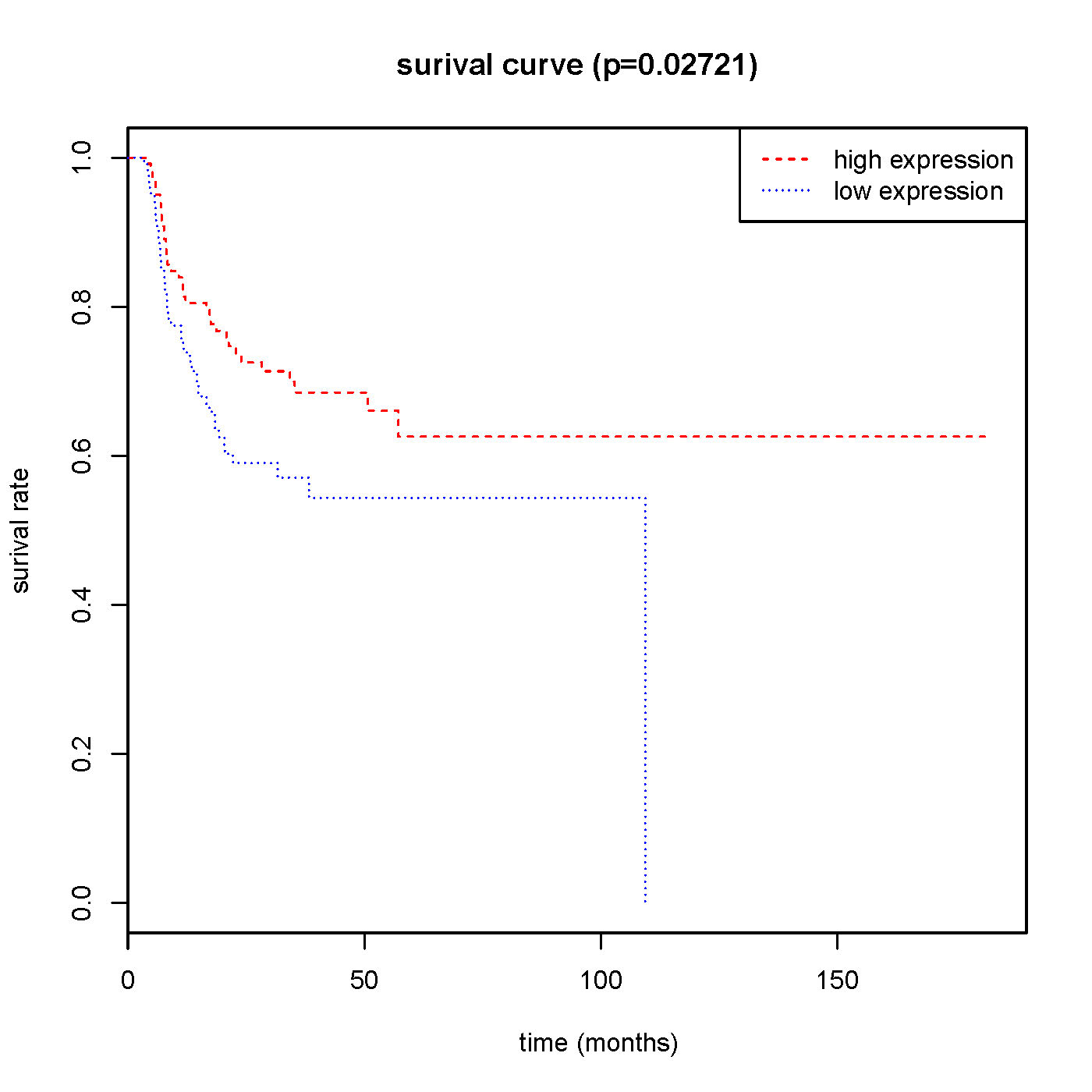

Supplement: Dataset S1 — Kaplan–Meier survival analysis with the log-rank test was used to identify relationships between the above 2493 lncRNA signatures and OSCC patient survival. Then, we determined the levels of 126 lncRNA signatures that were significantly related to DFS. [file peerj-06-5307-s005.zip › The result of Kaplan–Meier survival analyses and log-rank tests for DFS in OSCC/RP11-538D16.2.jpg]

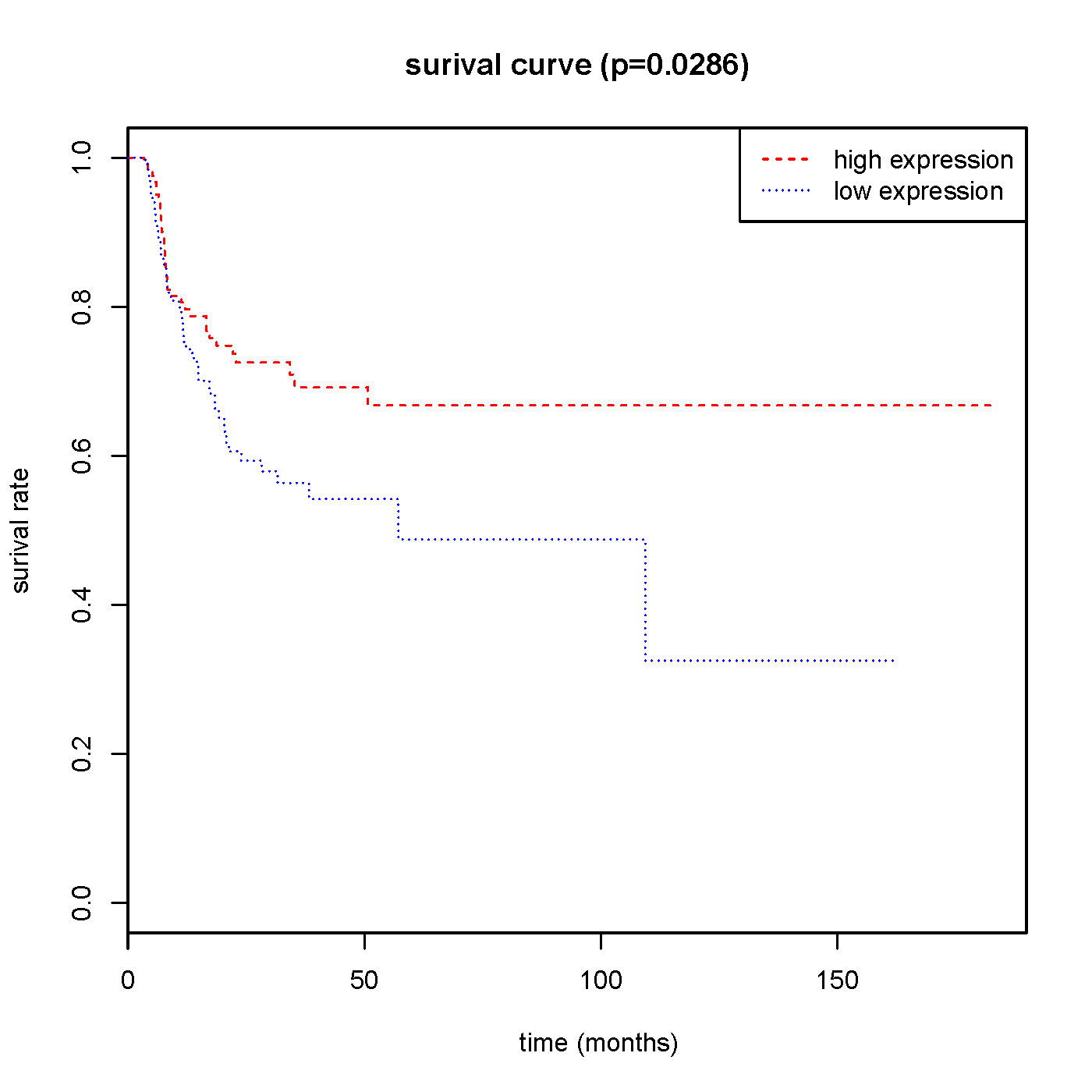

Supplement: Dataset S1 — Kaplan–Meier survival analysis with the log-rank test was used to identify relationships between the above 2493 lncRNA signatures and OSCC patient survival. Then, we determined the levels of 126 lncRNA signatures that were significantly related to DFS. [file peerj-06-5307-s005.zip › The result of Kaplan–Meier survival analyses and log-rank tests for DFS in OSCC/RP11-552M14.1.jpg]

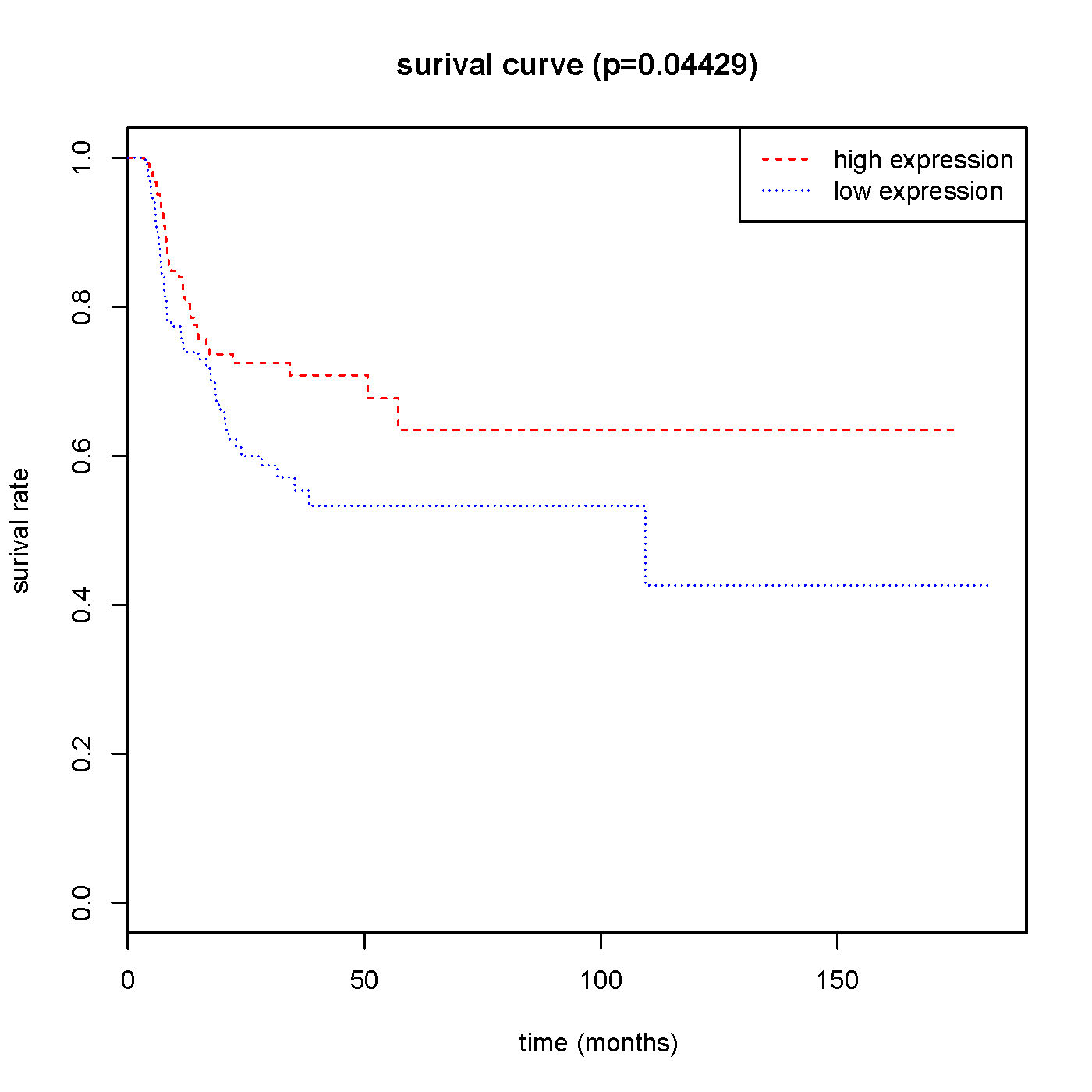

Supplement: Dataset S1 — Kaplan–Meier survival analysis with the log-rank test was used to identify relationships between the above 2493 lncRNA signatures and OSCC patient survival. Then, we determined the levels of 126 lncRNA signatures that were significantly related to DFS. [file peerj-06-5307-s005.zip › The result of Kaplan–Meier survival analyses and log-rank tests for DFS in OSCC/RP11-575F12.2.jpg]

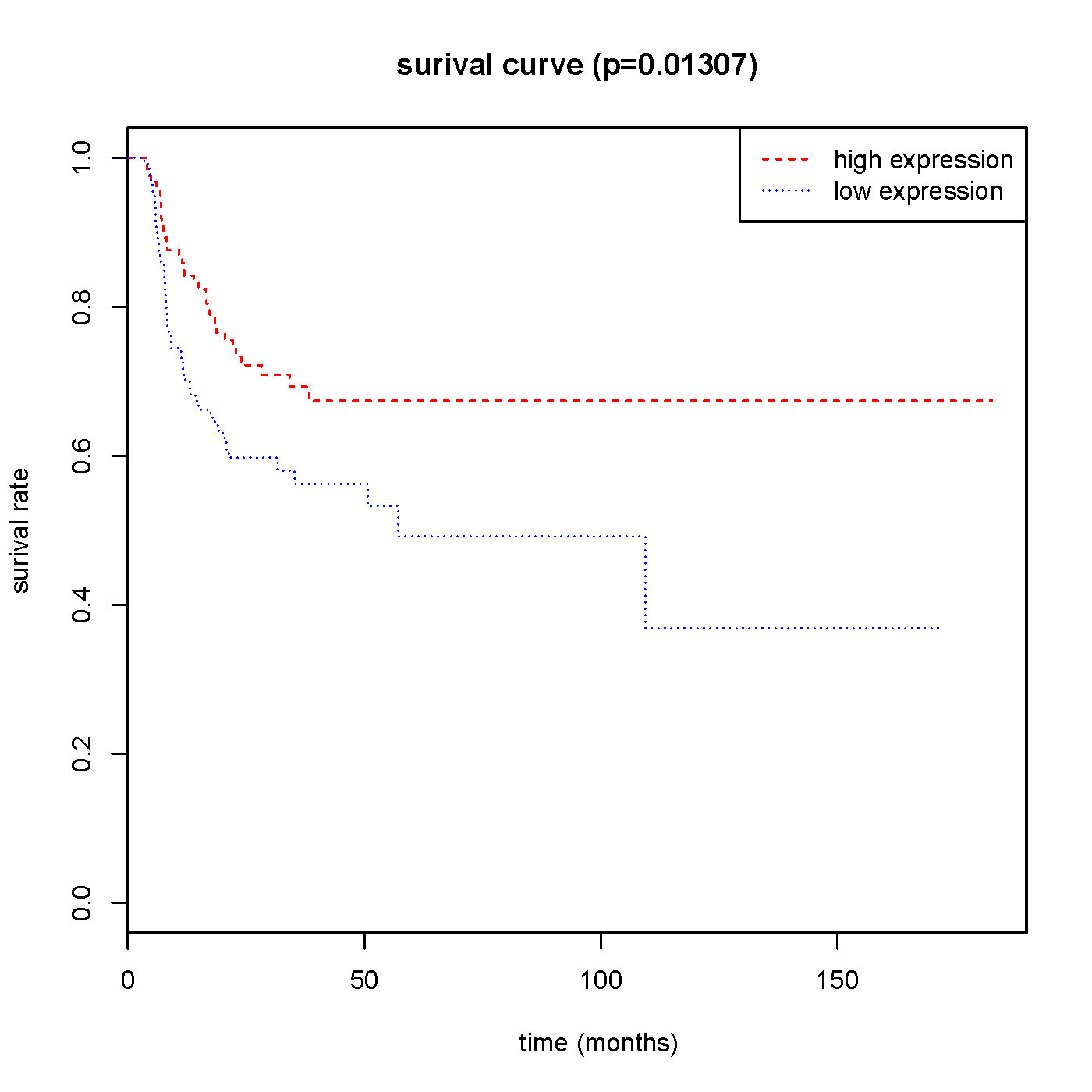

Supplement: Dataset S1 — Kaplan–Meier survival analysis with the log-rank test was used to identify relationships between the above 2493 lncRNA signatures and OSCC patient survival. Then, we determined the levels of 126 lncRNA signatures that were significantly related to DFS. [file peerj-06-5307-s005.zip › The result of Kaplan–Meier survival analyses and log-rank tests for DFS in OSCC/RP11-598F7.6.jpg]

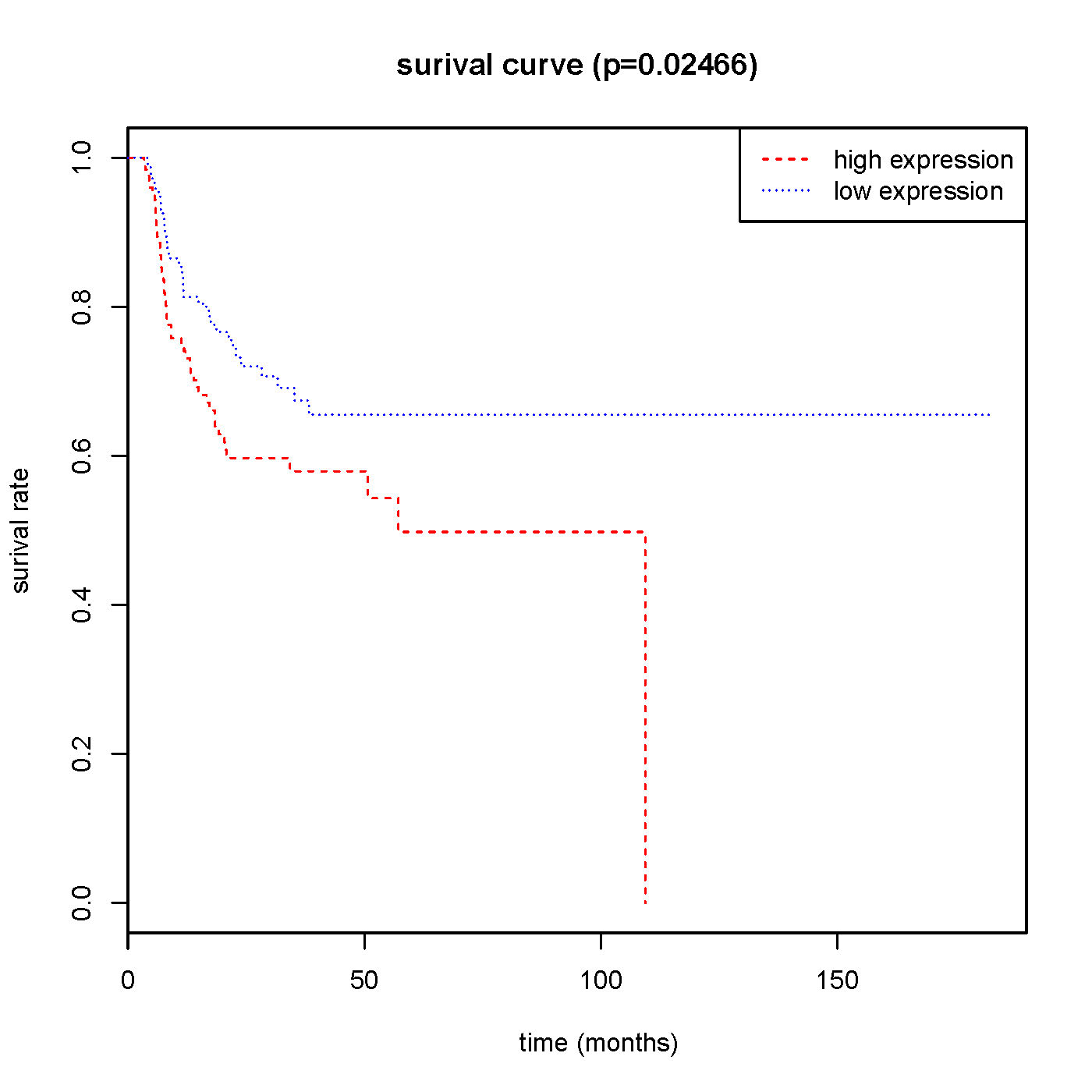

Supplement: Dataset S1 — Kaplan–Meier survival analysis with the log-rank test was used to identify relationships between the above 2493 lncRNA signatures and OSCC patient survival. Then, we determined the levels of 126 lncRNA signatures that were significantly related to DFS. [file peerj-06-5307-s005.zip › The result of Kaplan–Meier survival analyses and log-rank tests for DFS in OSCC/RP11-644C3.1.jpg]
