# Supplementary figures and images for: A novel long non-coding RNA, AC012456.4, as a valuable and independent prognostic biomarker of survival in oral squamous cell carcinoma (part 3 of 3)
Source: PeerJ. 2018 Aug 13;6:e5307. doi: 10.7717/peerj.5307 (PMC6095106; doi:10.7717/peerj.5307)

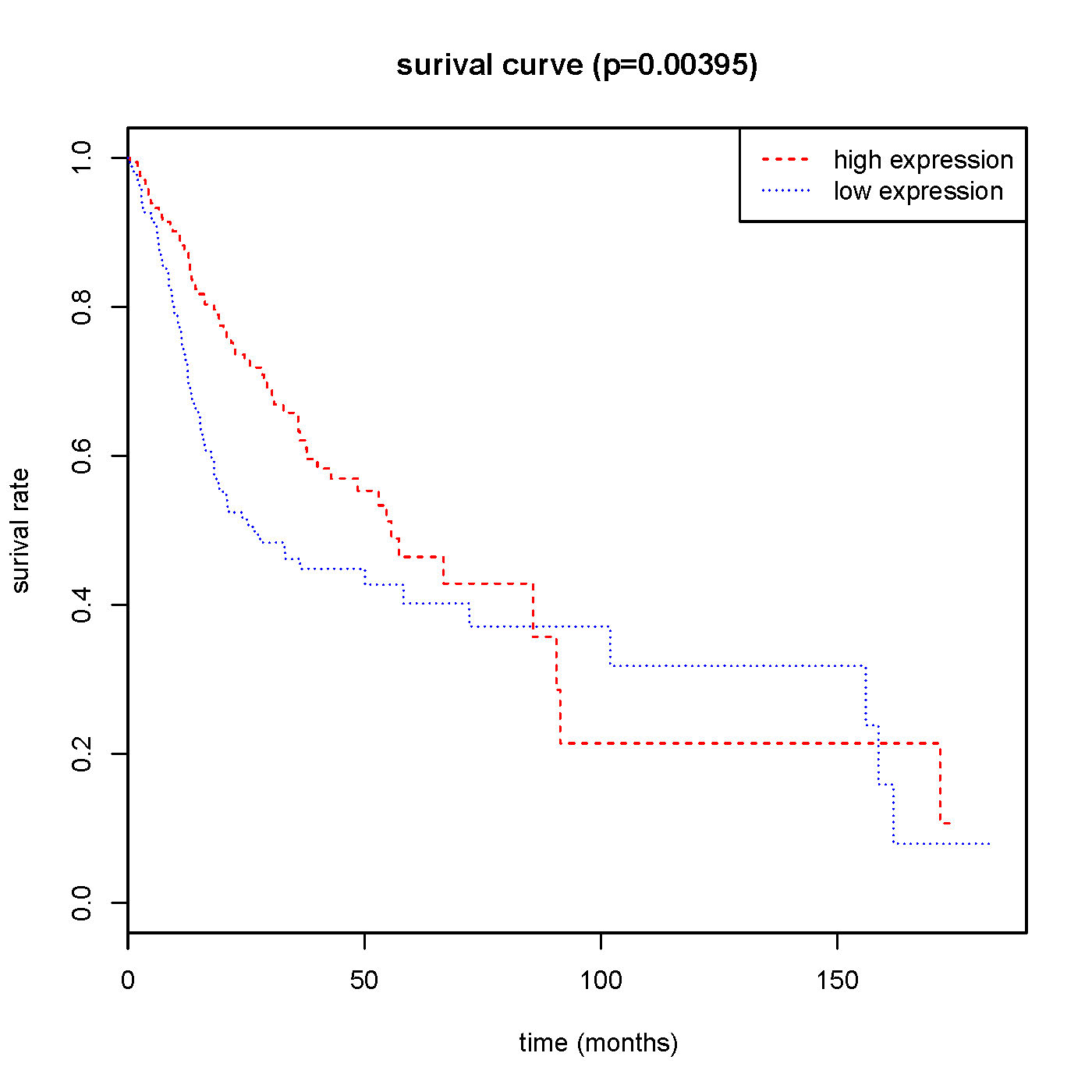

Supplement: Dataset S2 — Kaplan–Meier survival analysis with the log-rank was used to identify relationships between the above 2493 lncRNA signatures and OSCC patient survival. Then, we determined the levels of 151 lncRNA signatures that were significantly related to OS. [file peerj-06-5307-s006.zip › The result of Kaplan–Meier survival analyses and log-rank tests for OS in OSCC/LINC01896.jpg]

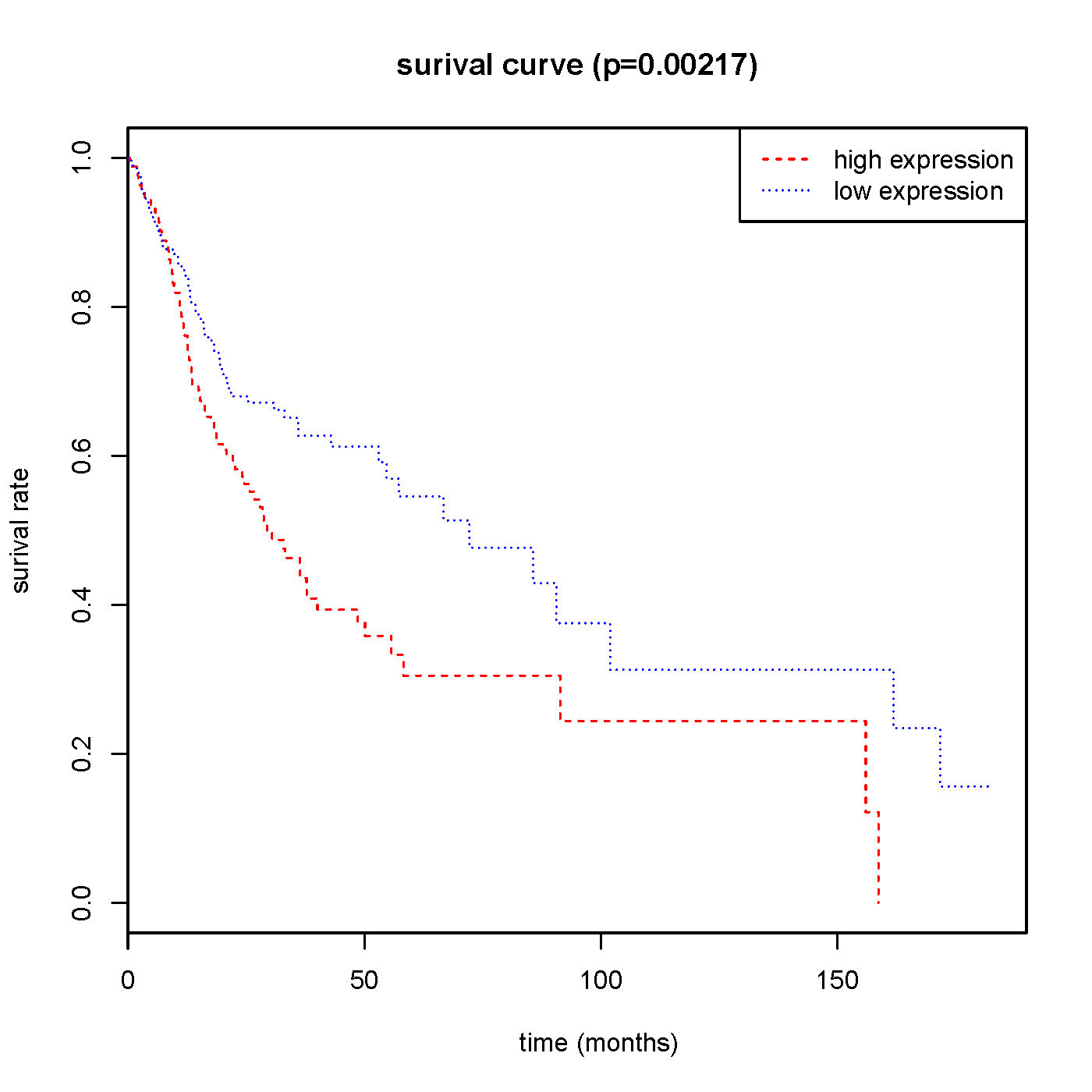

Supplement: Dataset S2 — Kaplan–Meier survival analysis with the log-rank was used to identify relationships between the above 2493 lncRNA signatures and OSCC patient survival. Then, we determined the levels of 151 lncRNA signatures that were significantly related to OS. [file peerj-06-5307-s006.zip › The result of Kaplan–Meier survival analyses and log-rank tests for OS in OSCC/LINC01983.jpg]

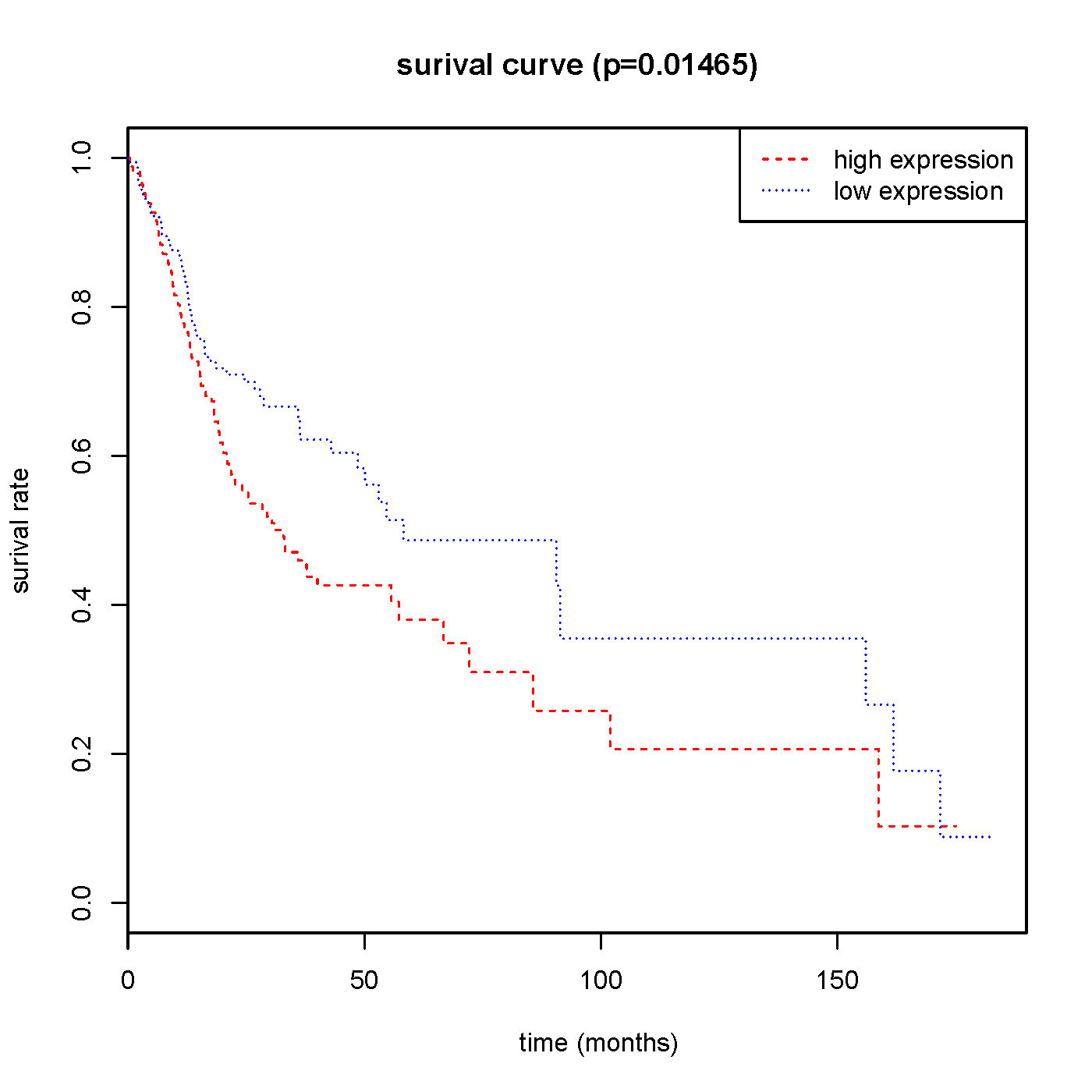

Supplement: Dataset S2 — Kaplan–Meier survival analysis with the log-rank was used to identify relationships between the above 2493 lncRNA signatures and OSCC patient survival. Then, we determined the levels of 151 lncRNA signatures that were significantly related to OS. [file peerj-06-5307-s006.zip › The result of Kaplan–Meier survival analyses and log-rank tests for OS in OSCC/LINC02100.jpg]

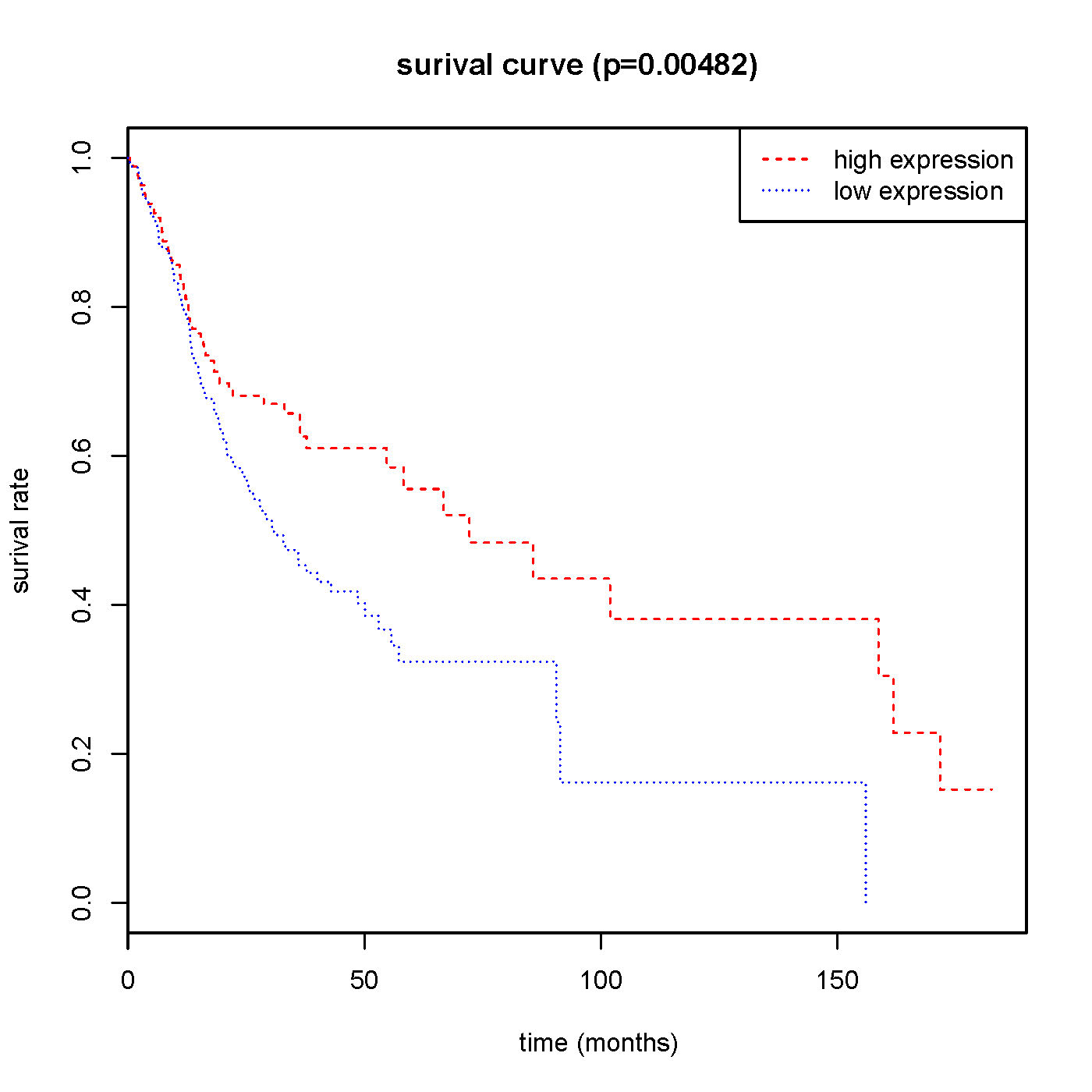

Supplement: Dataset S2 — Kaplan–Meier survival analysis with the log-rank was used to identify relationships between the above 2493 lncRNA signatures and OSCC patient survival. Then, we determined the levels of 151 lncRNA signatures that were significantly related to OS. [file peerj-06-5307-s006.zip › The result of Kaplan–Meier survival analyses and log-rank tests for OS in OSCC/LINC02166.jpg]

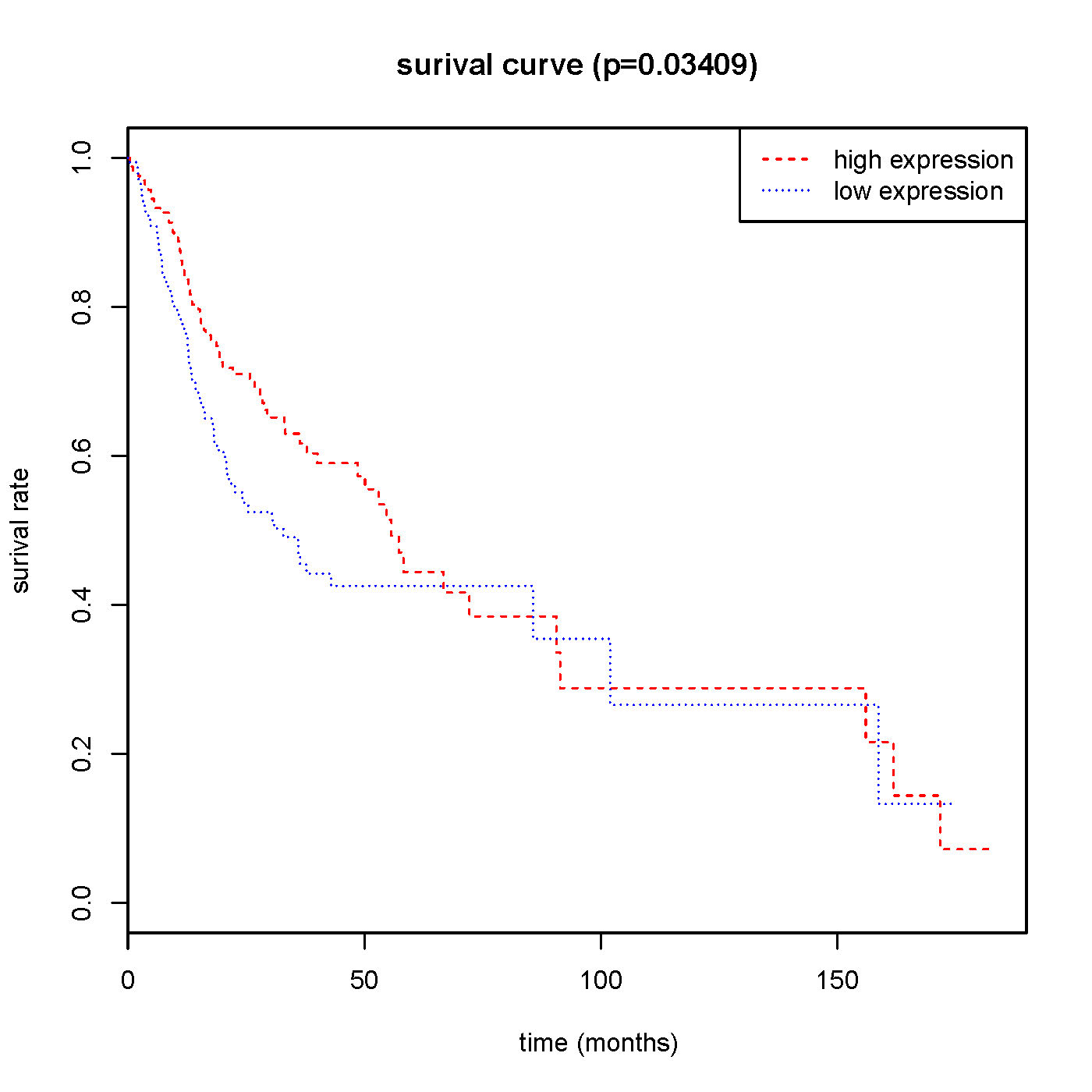

Supplement: Dataset S2 — Kaplan–Meier survival analysis with the log-rank was used to identify relationships between the above 2493 lncRNA signatures and OSCC patient survival. Then, we determined the levels of 151 lncRNA signatures that were significantly related to OS. [file peerj-06-5307-s006.zip › The result of Kaplan–Meier survival analyses and log-rank tests for OS in OSCC/MIR99AHG.jpg]

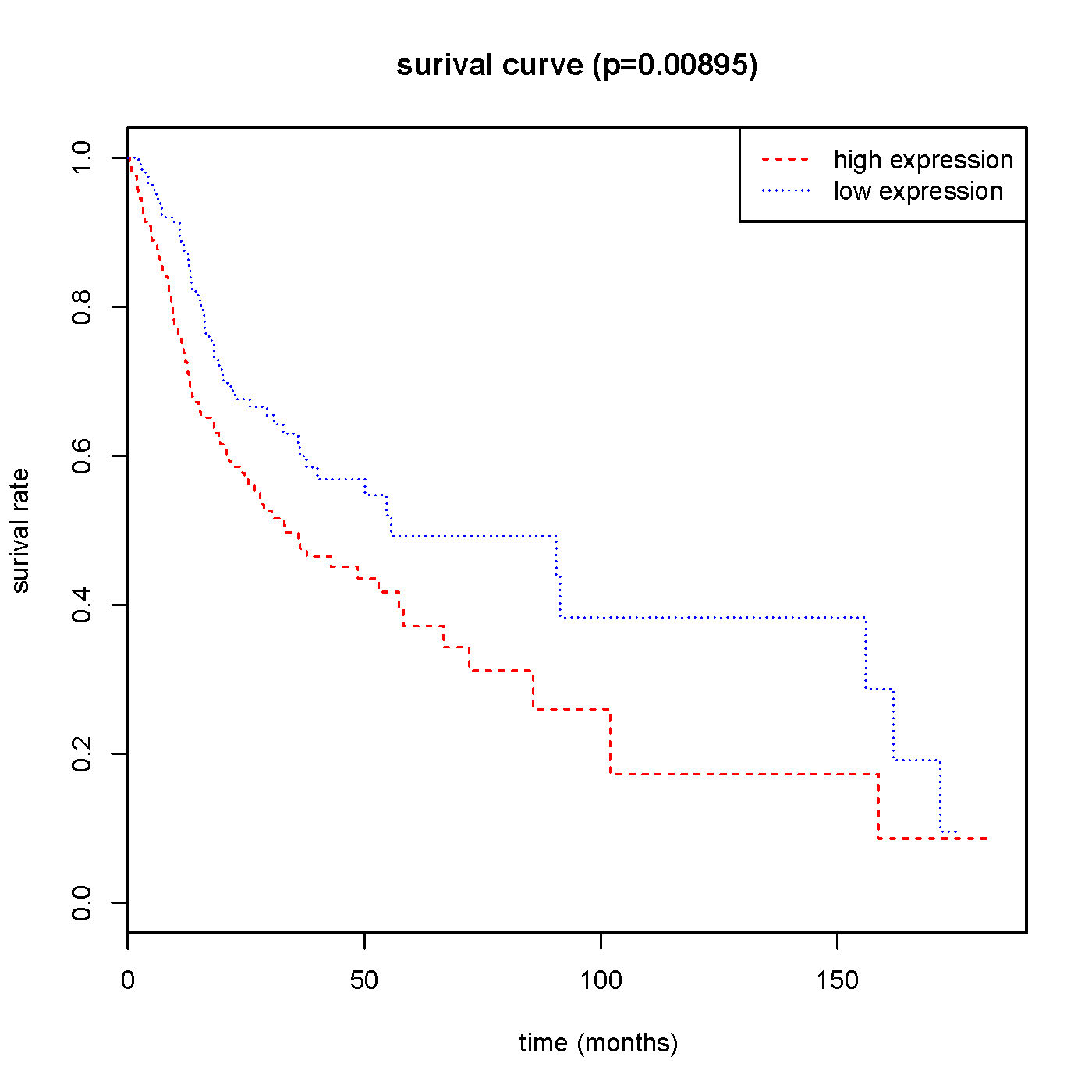

Supplement: Dataset S2 — Kaplan–Meier survival analysis with the log-rank was used to identify relationships between the above 2493 lncRNA signatures and OSCC patient survival. Then, we determined the levels of 151 lncRNA signatures that were significantly related to OS. [file peerj-06-5307-s006.zip › The result of Kaplan–Meier survival analyses and log-rank tests for OS in OSCC/POT1-AS1.jpg]

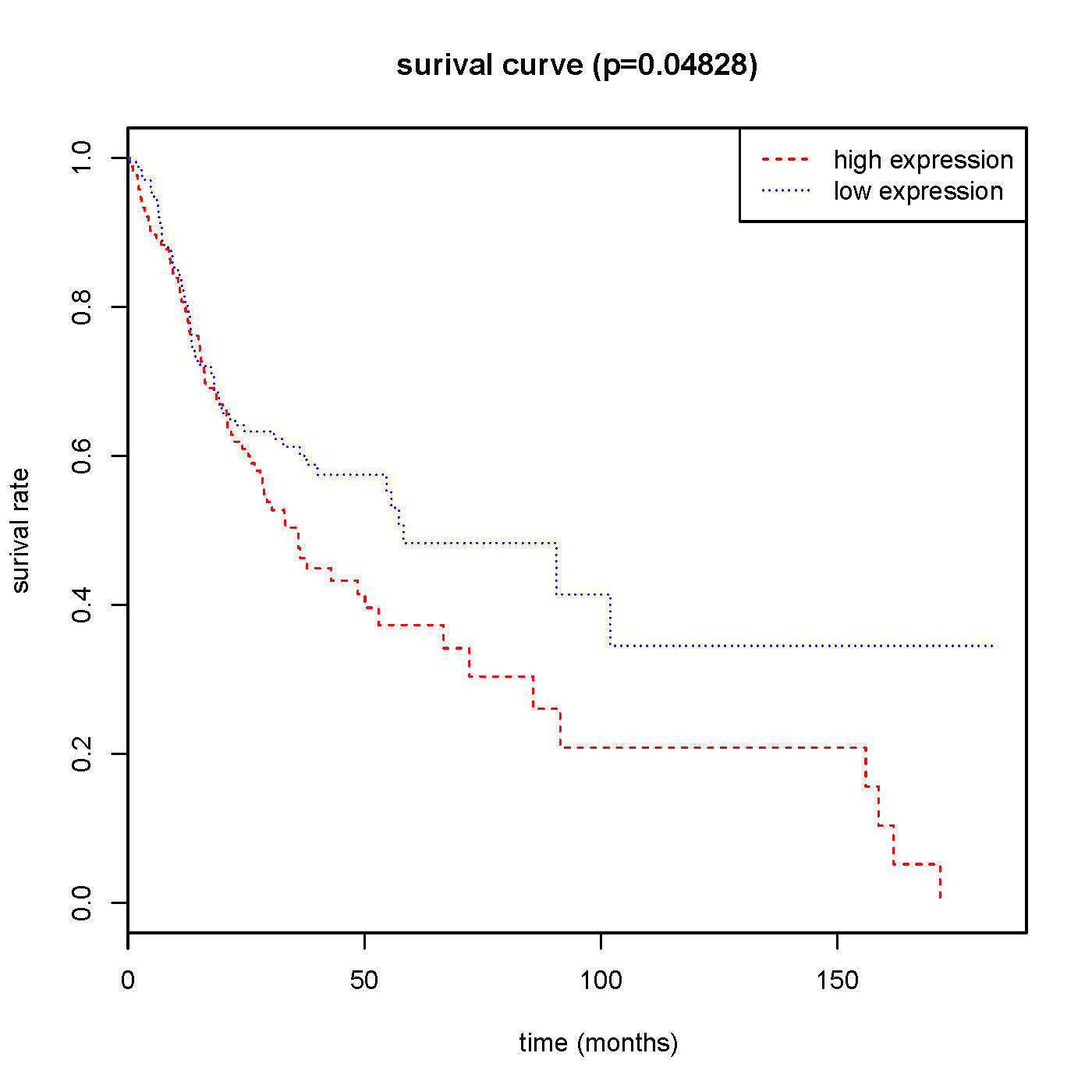

Supplement: Dataset S2 — Kaplan–Meier survival analysis with the log-rank was used to identify relationships between the above 2493 lncRNA signatures and OSCC patient survival. Then, we determined the levels of 151 lncRNA signatures that were significantly related to OS. [file peerj-06-5307-s006.zip › The result of Kaplan–Meier survival analyses and log-rank tests for OS in OSCC/PRKAG2-AS1.jpg]

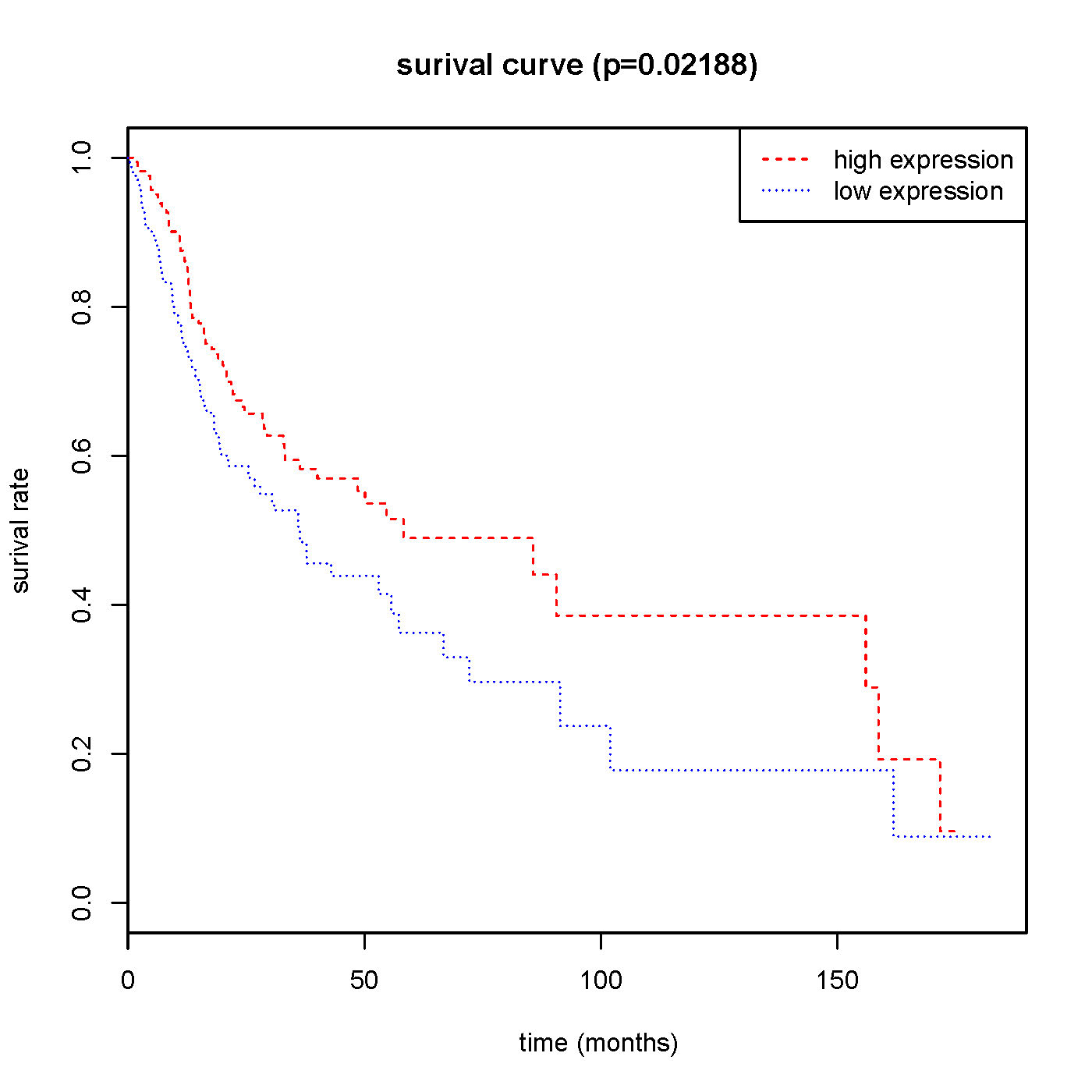

Supplement: Dataset S2 — Kaplan–Meier survival analysis with the log-rank was used to identify relationships between the above 2493 lncRNA signatures and OSCC patient survival. Then, we determined the levels of 151 lncRNA signatures that were significantly related to OS. [file peerj-06-5307-s006.zip › The result of Kaplan–Meier survival analyses and log-rank tests for OS in OSCC/RP11-1036E20.9.jpg]

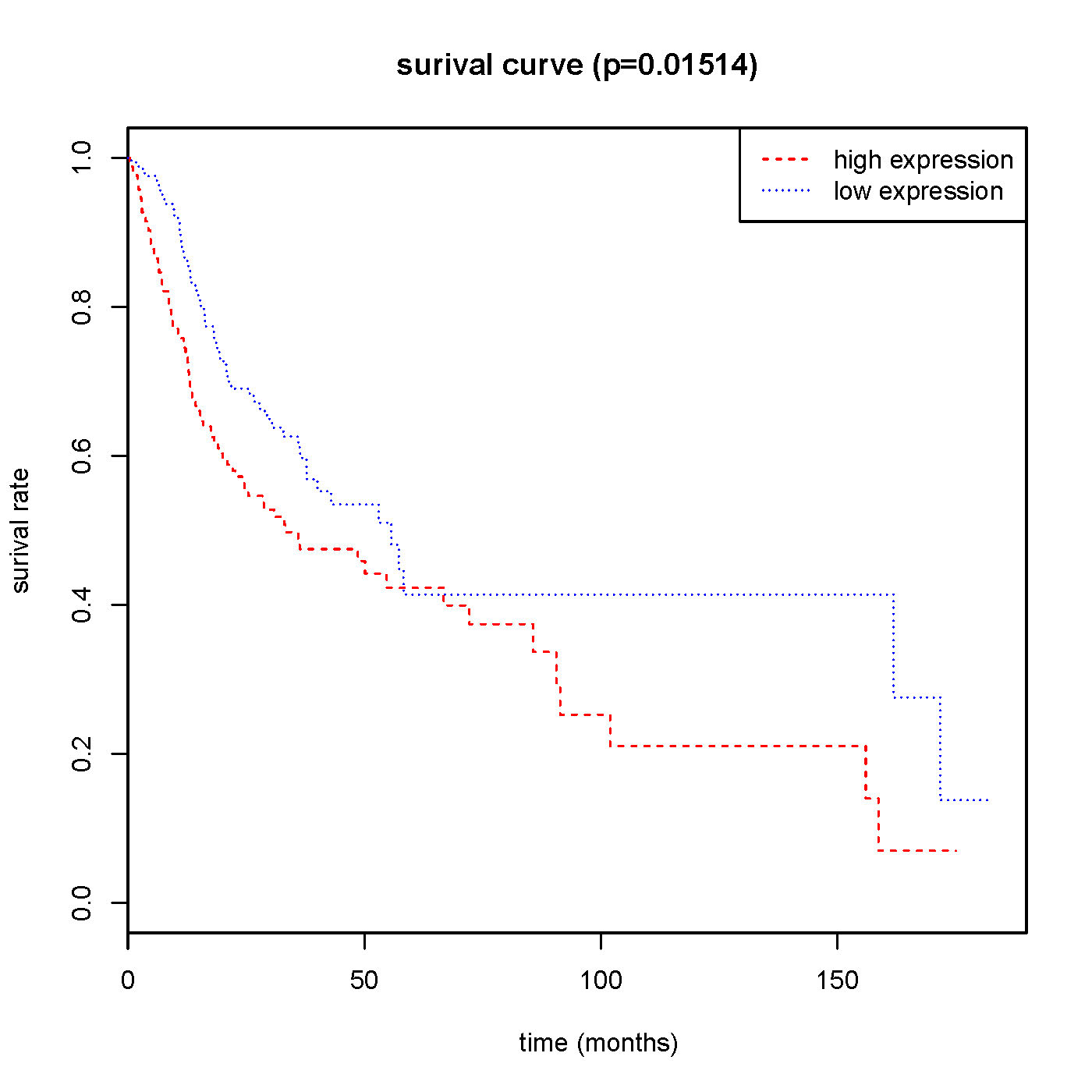

Supplement: Dataset S2 — Kaplan–Meier survival analysis with the log-rank was used to identify relationships between the above 2493 lncRNA signatures and OSCC patient survival. Then, we determined the levels of 151 lncRNA signatures that were significantly related to OS. [file peerj-06-5307-s006.zip › The result of Kaplan–Meier survival analyses and log-rank tests for OS in OSCC/RP11-103C16.2.jpg]

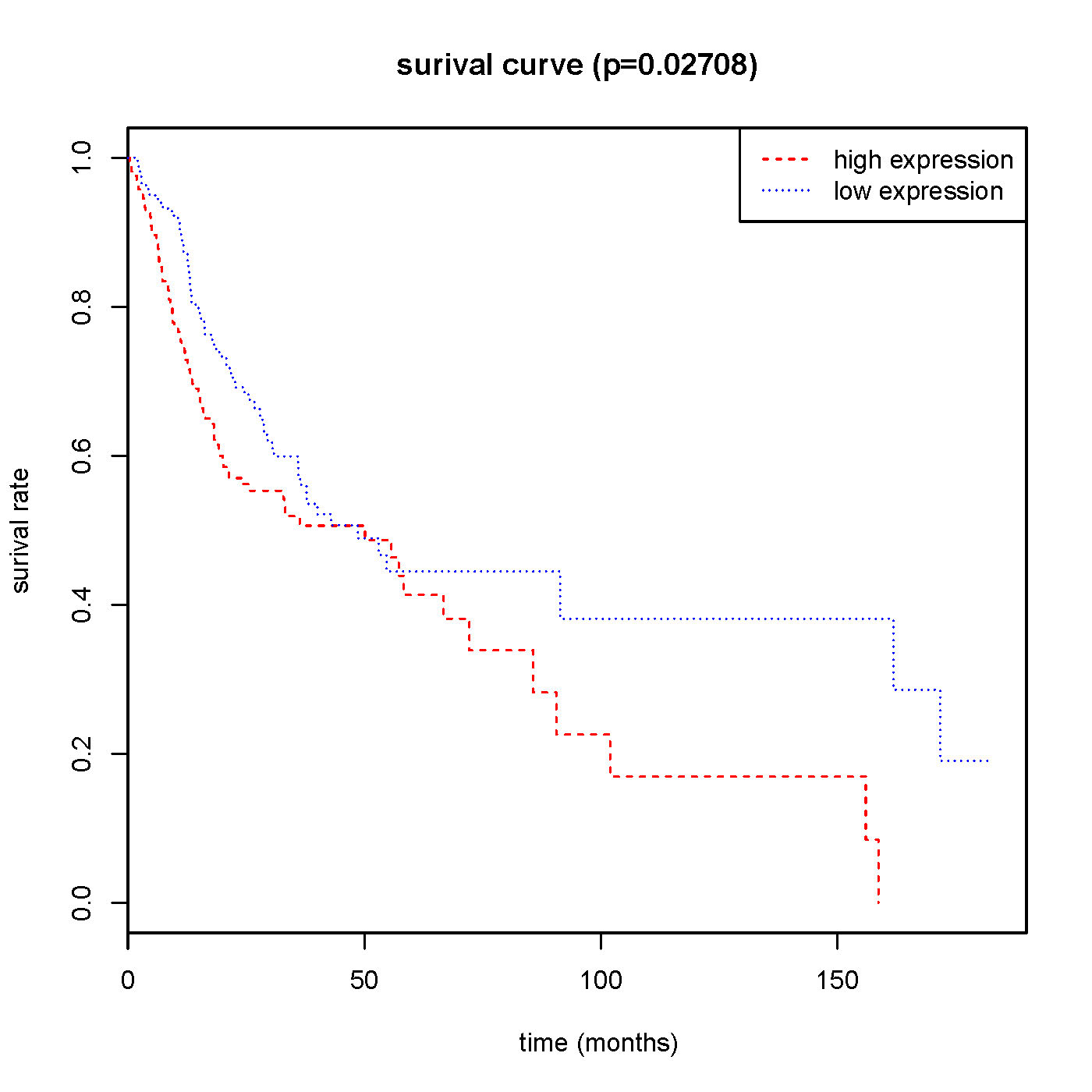

Supplement: Dataset S2 — Kaplan–Meier survival analysis with the log-rank was used to identify relationships between the above 2493 lncRNA signatures and OSCC patient survival. Then, we determined the levels of 151 lncRNA signatures that were significantly related to OS. [file peerj-06-5307-s006.zip › The result of Kaplan–Meier survival analyses and log-rank tests for OS in OSCC/RP11-108K3.1.jpg]

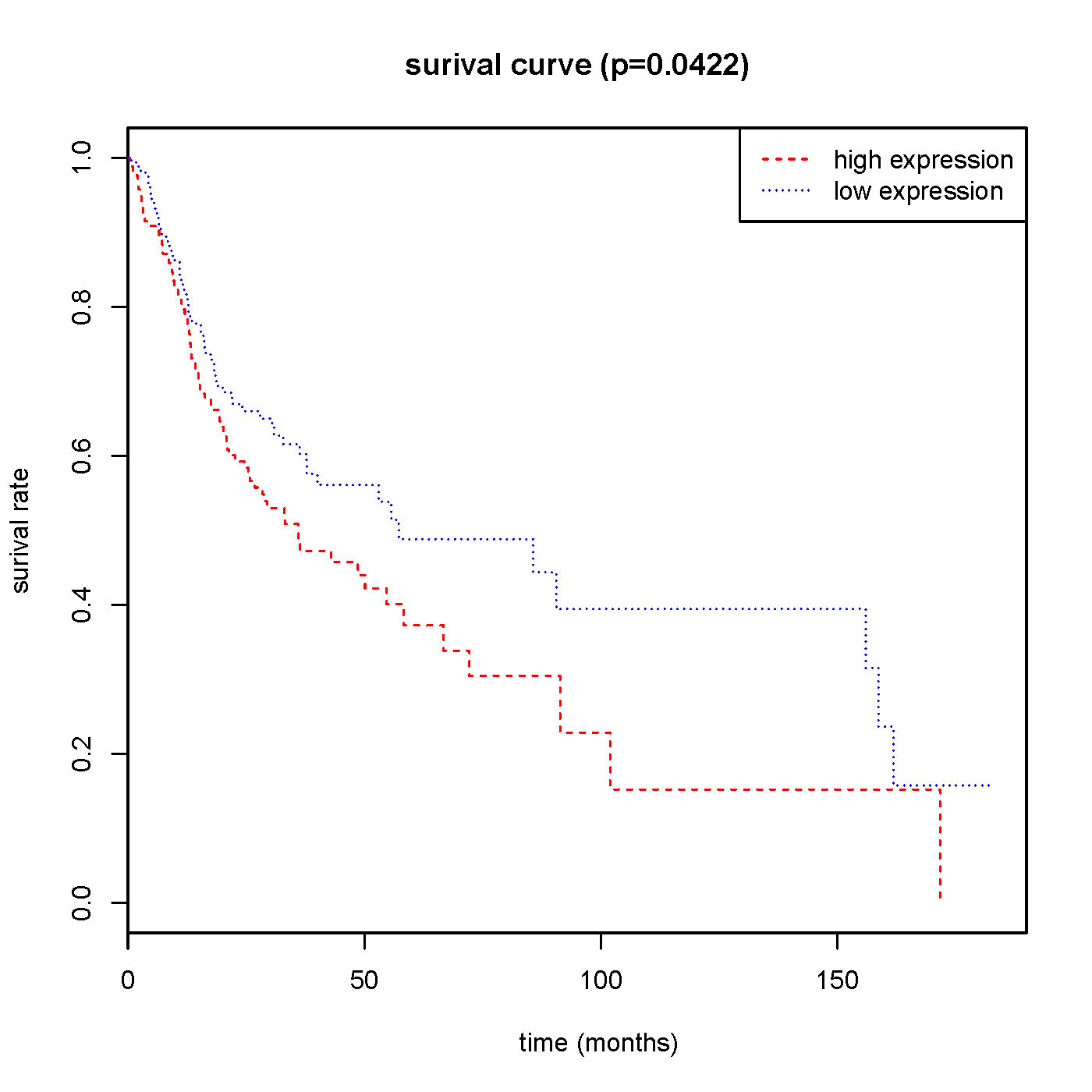

Supplement: Dataset S2 — Kaplan–Meier survival analysis with the log-rank was used to identify relationships between the above 2493 lncRNA signatures and OSCC patient survival. Then, we determined the levels of 151 lncRNA signatures that were significantly related to OS. [file peerj-06-5307-s006.zip › The result of Kaplan–Meier survival analyses and log-rank tests for OS in OSCC/RP11-1103G16.1.jpg]

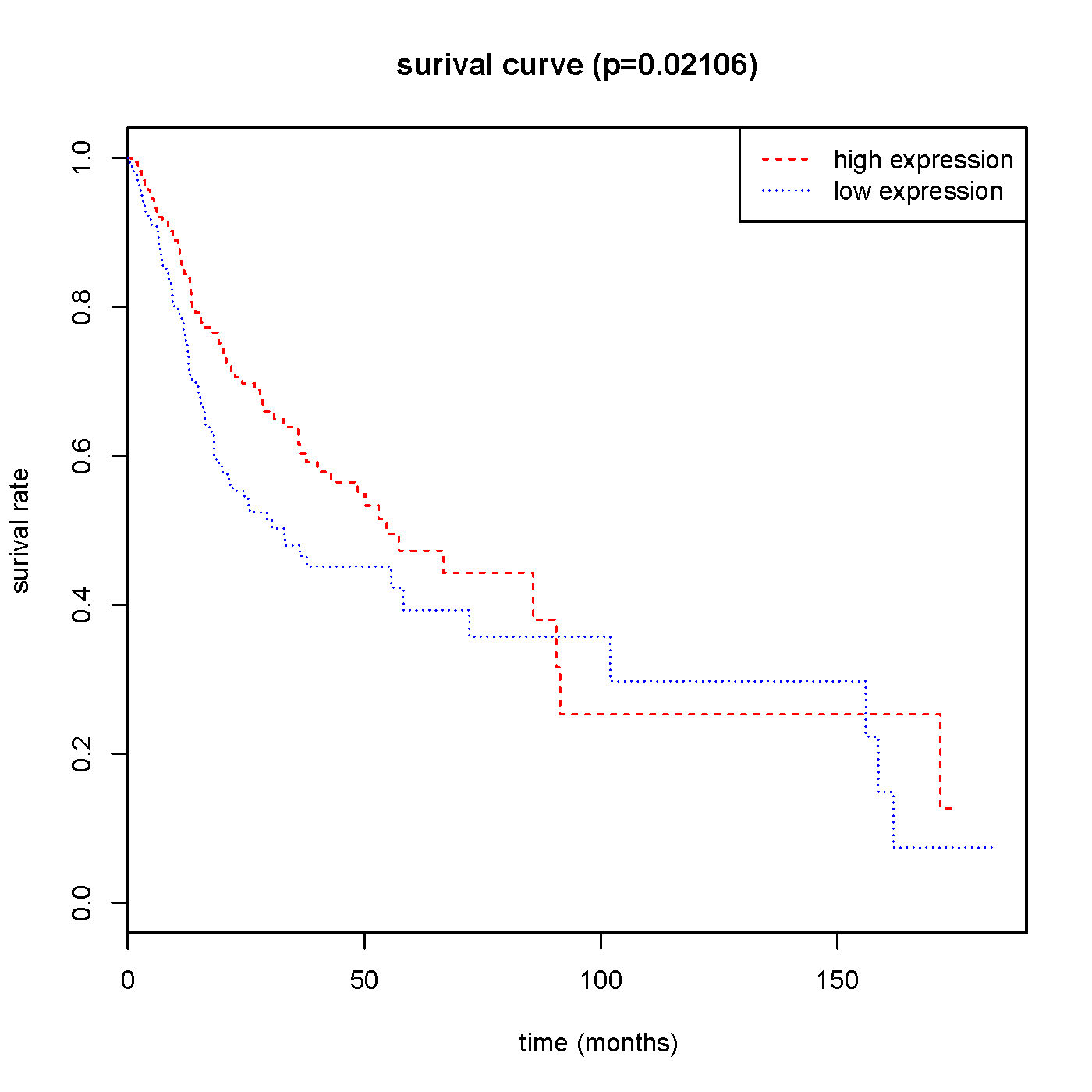

Supplement: Dataset S2 — Kaplan–Meier survival analysis with the log-rank was used to identify relationships between the above 2493 lncRNA signatures and OSCC patient survival. Then, we determined the levels of 151 lncRNA signatures that were significantly related to OS. [file peerj-06-5307-s006.zip › The result of Kaplan–Meier survival analyses and log-rank tests for OS in OSCC/RP11-1263C18.1.jpg]

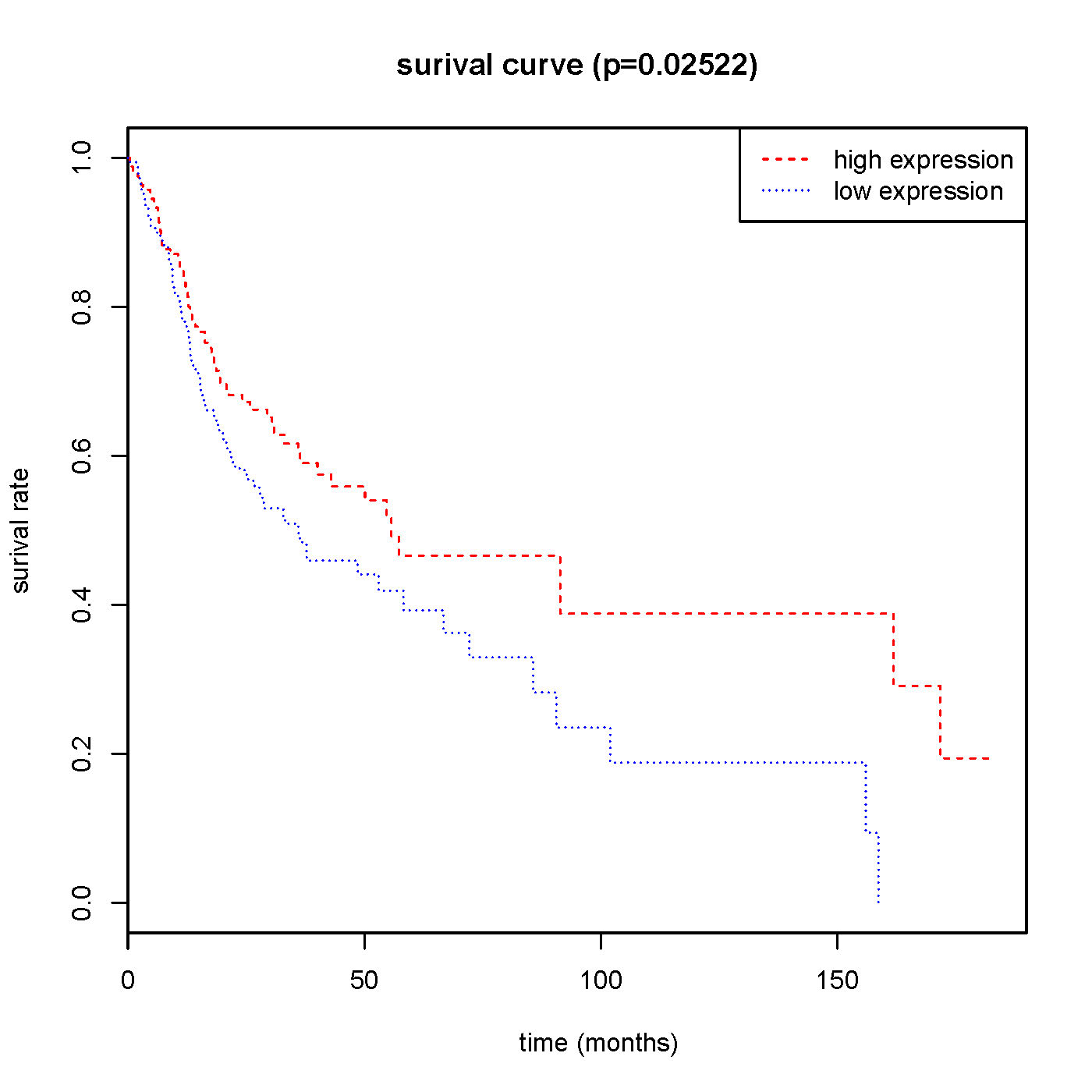

Supplement: Dataset S2 — Kaplan–Meier survival analysis with the log-rank was used to identify relationships between the above 2493 lncRNA signatures and OSCC patient survival. Then, we determined the levels of 151 lncRNA signatures that were significantly related to OS. [file peerj-06-5307-s006.zip › The result of Kaplan–Meier survival analyses and log-rank tests for OS in OSCC/RP11-164P12.3.jpg]

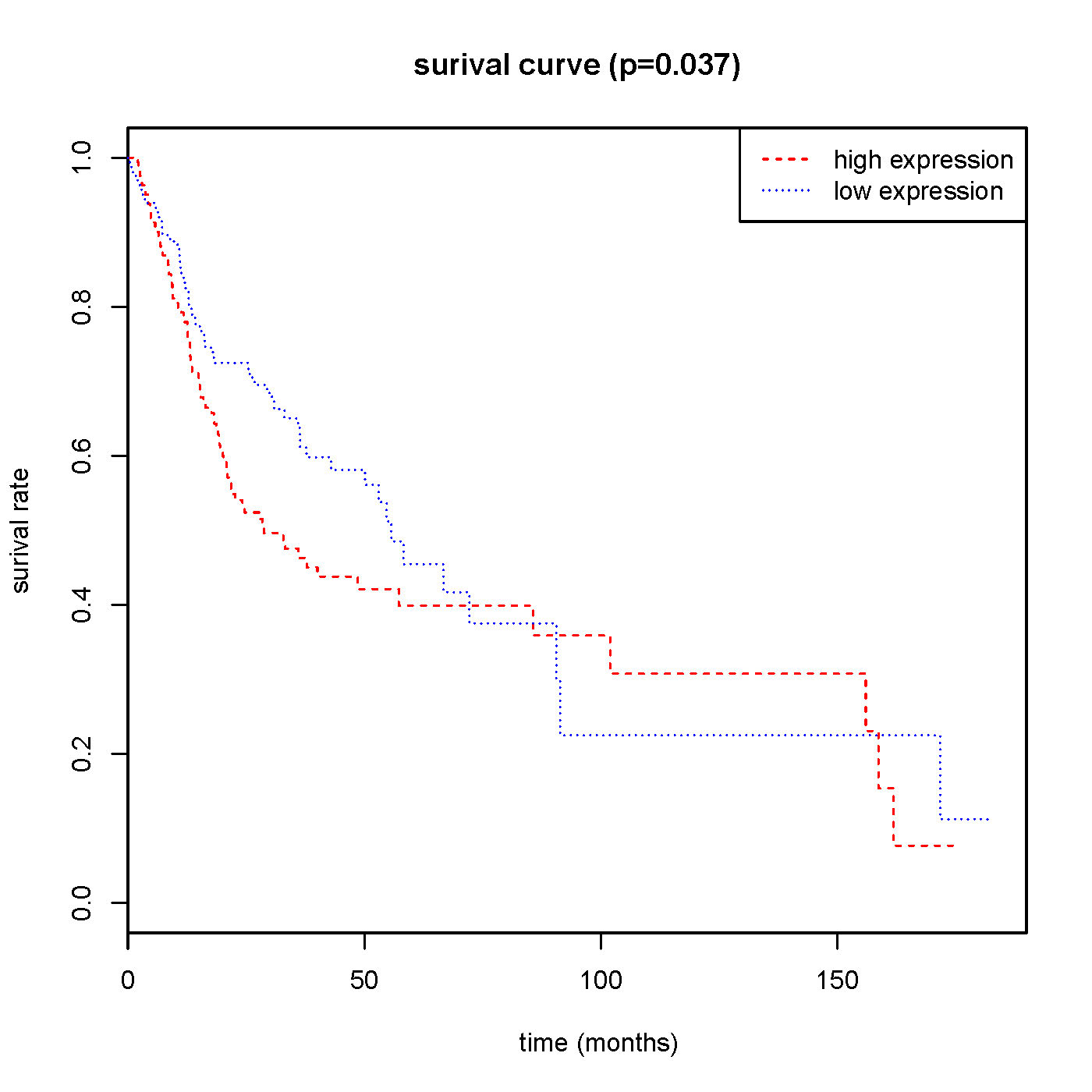

Supplement: Dataset S2 — Kaplan–Meier survival analysis with the log-rank was used to identify relationships between the above 2493 lncRNA signatures and OSCC patient survival. Then, we determined the levels of 151 lncRNA signatures that were significantly related to OS. [file peerj-06-5307-s006.zip › The result of Kaplan–Meier survival analyses and log-rank tests for OS in OSCC/RP11-16K12.1.jpg]

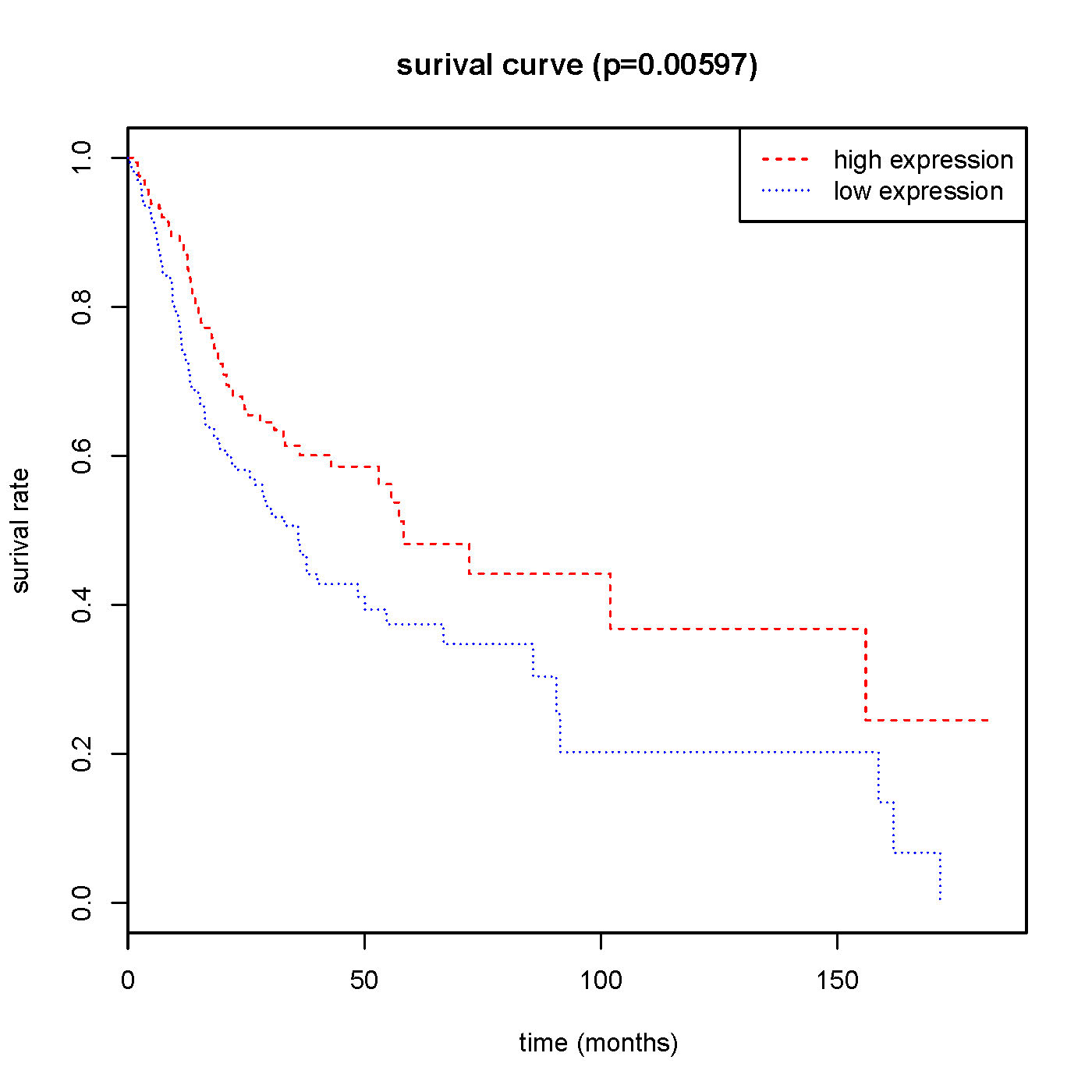

Supplement: Dataset S2 — Kaplan–Meier survival analysis with the log-rank was used to identify relationships between the above 2493 lncRNA signatures and OSCC patient survival. Then, we determined the levels of 151 lncRNA signatures that were significantly related to OS. [file peerj-06-5307-s006.zip › The result of Kaplan–Meier survival analyses and log-rank tests for OS in OSCC/RP11-181E10.3.jpg]

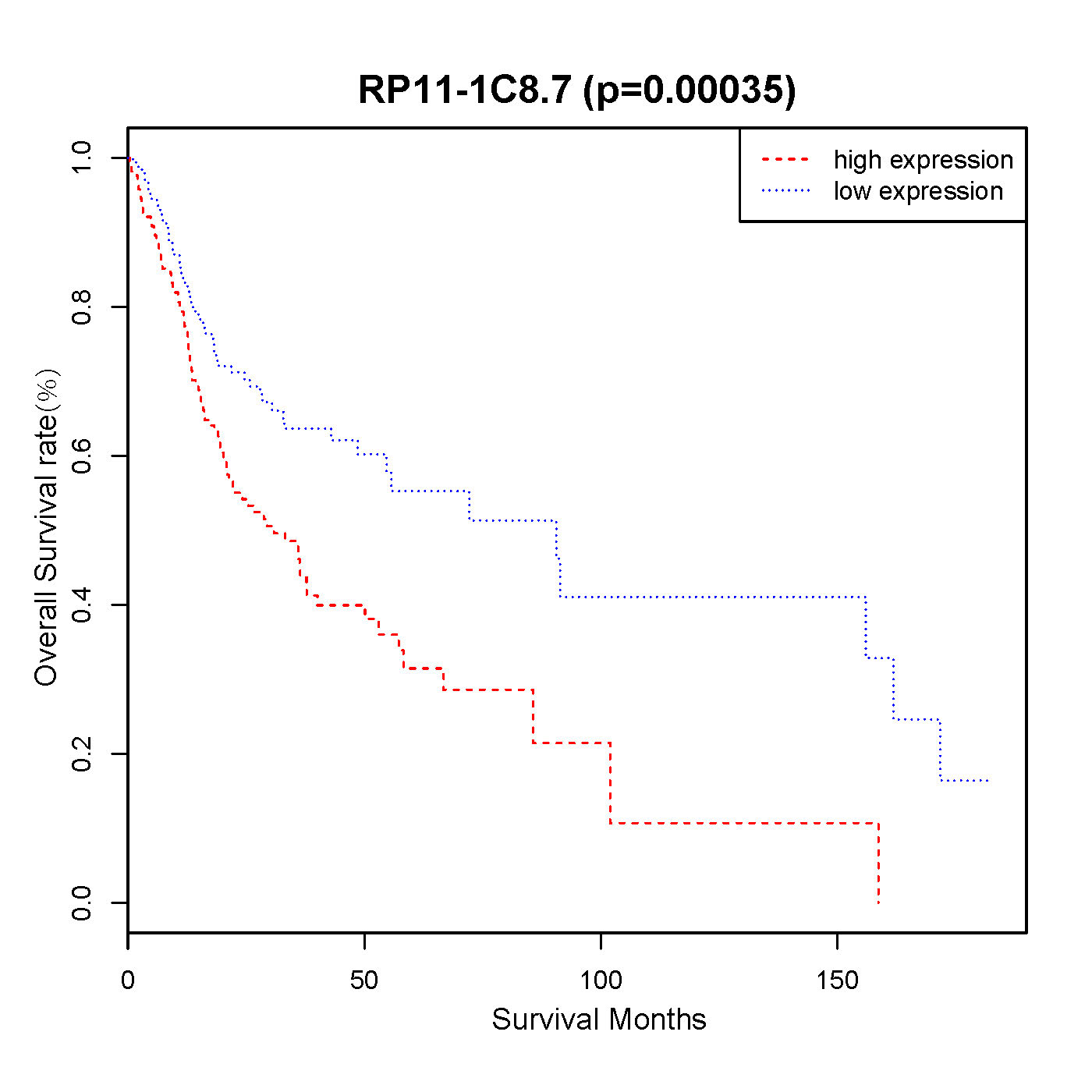

Supplement: Dataset S2 — Kaplan–Meier survival analysis with the log-rank was used to identify relationships between the above 2493 lncRNA signatures and OSCC patient survival. Then, we determined the levels of 151 lncRNA signatures that were significantly related to OS. [file peerj-06-5307-s006.zip › The result of Kaplan–Meier survival analyses and log-rank tests for OS in OSCC/RP11-1C8.7.jpg]

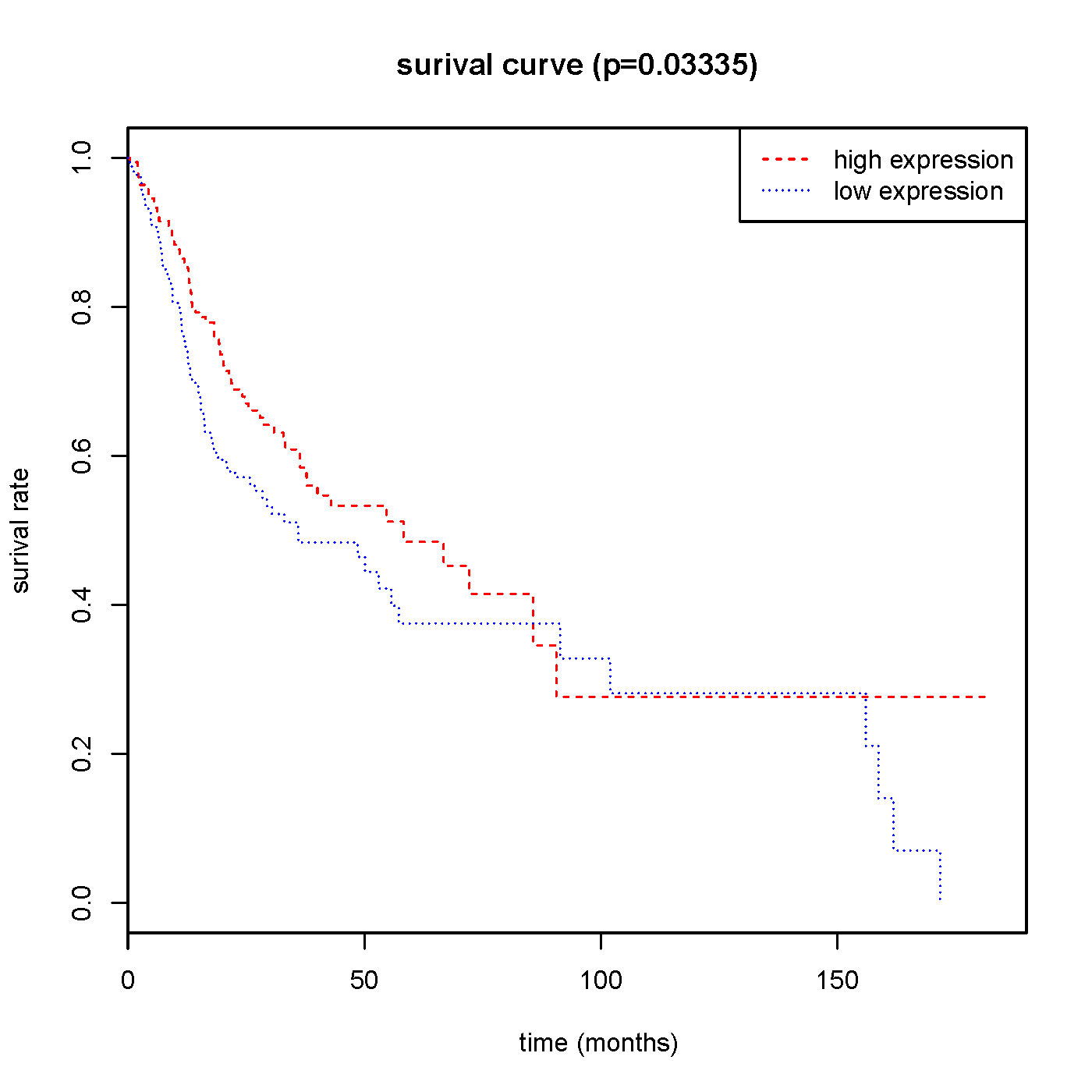

Supplement: Dataset S2 — Kaplan–Meier survival analysis with the log-rank was used to identify relationships between the above 2493 lncRNA signatures and OSCC patient survival. Then, we determined the levels of 151 lncRNA signatures that were significantly related to OS. [file peerj-06-5307-s006.zip › The result of Kaplan–Meier survival analyses and log-rank tests for OS in OSCC/RP11-1D12.2.jpg]

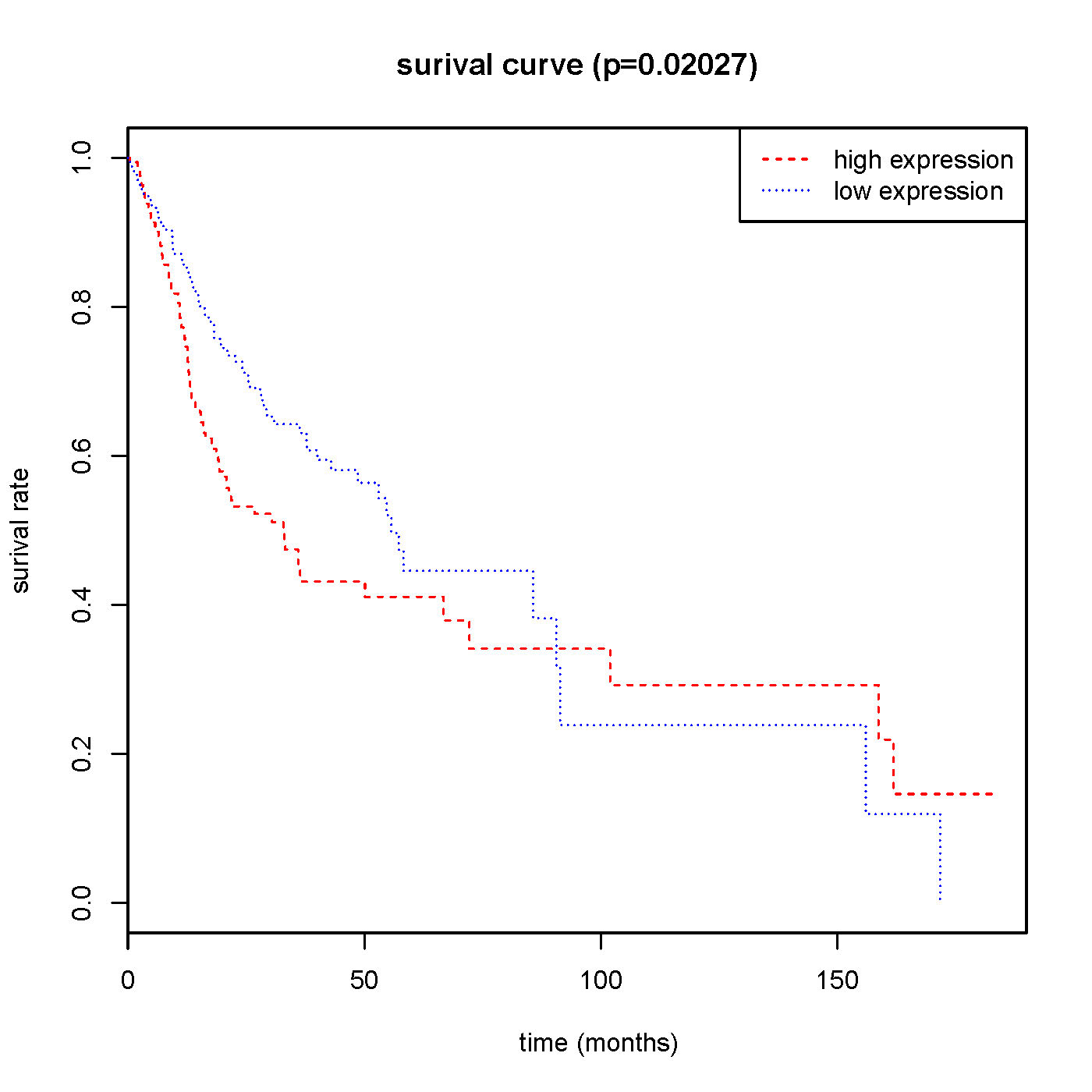

Supplement: Dataset S2 — Kaplan–Meier survival analysis with the log-rank was used to identify relationships between the above 2493 lncRNA signatures and OSCC patient survival. Then, we determined the levels of 151 lncRNA signatures that were significantly related to OS. [file peerj-06-5307-s006.zip › The result of Kaplan–Meier survival analyses and log-rank tests for OS in OSCC/RP11-209D14.4.jpg]

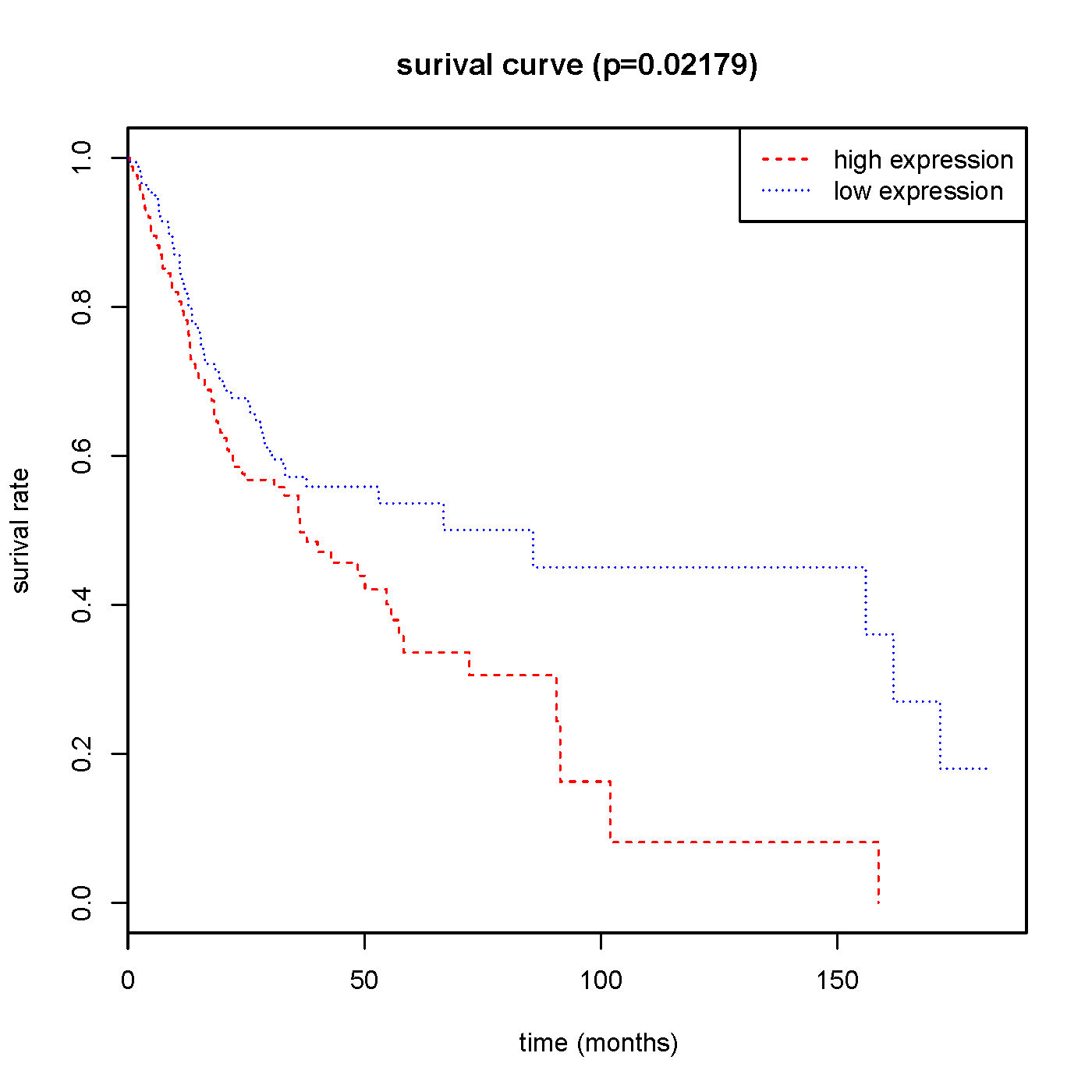

Supplement: Dataset S2 — Kaplan–Meier survival analysis with the log-rank was used to identify relationships between the above 2493 lncRNA signatures and OSCC patient survival. Then, we determined the levels of 151 lncRNA signatures that were significantly related to OS. [file peerj-06-5307-s006.zip › The result of Kaplan–Meier survival analyses and log-rank tests for OS in OSCC/RP11-215P8.4.jpg]

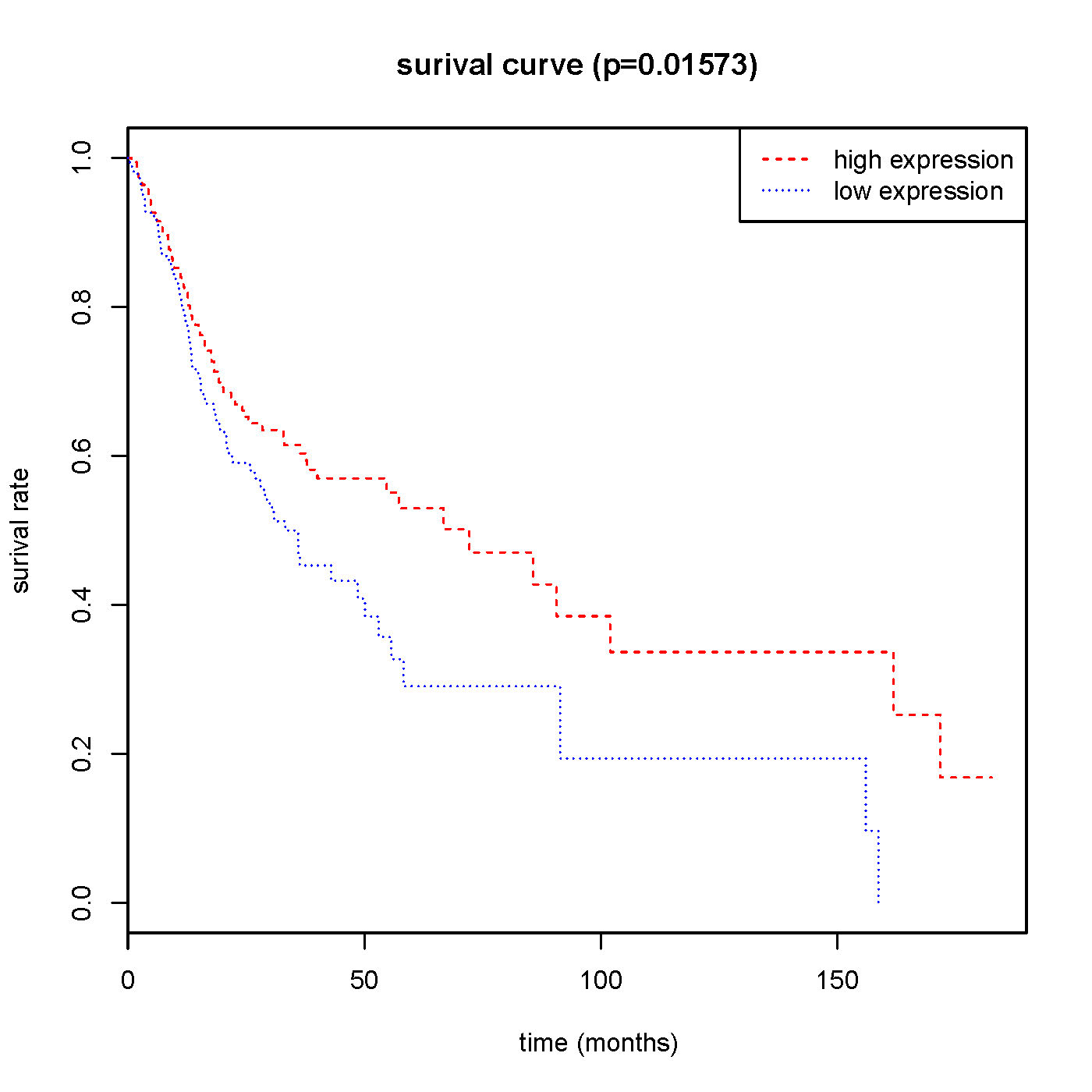

Supplement: Dataset S2 — Kaplan–Meier survival analysis with the log-rank was used to identify relationships between the above 2493 lncRNA signatures and OSCC patient survival. Then, we determined the levels of 151 lncRNA signatures that were significantly related to OS. [file peerj-06-5307-s006.zip › The result of Kaplan–Meier survival analyses and log-rank tests for OS in OSCC/RP11-22D3.2.jpg]

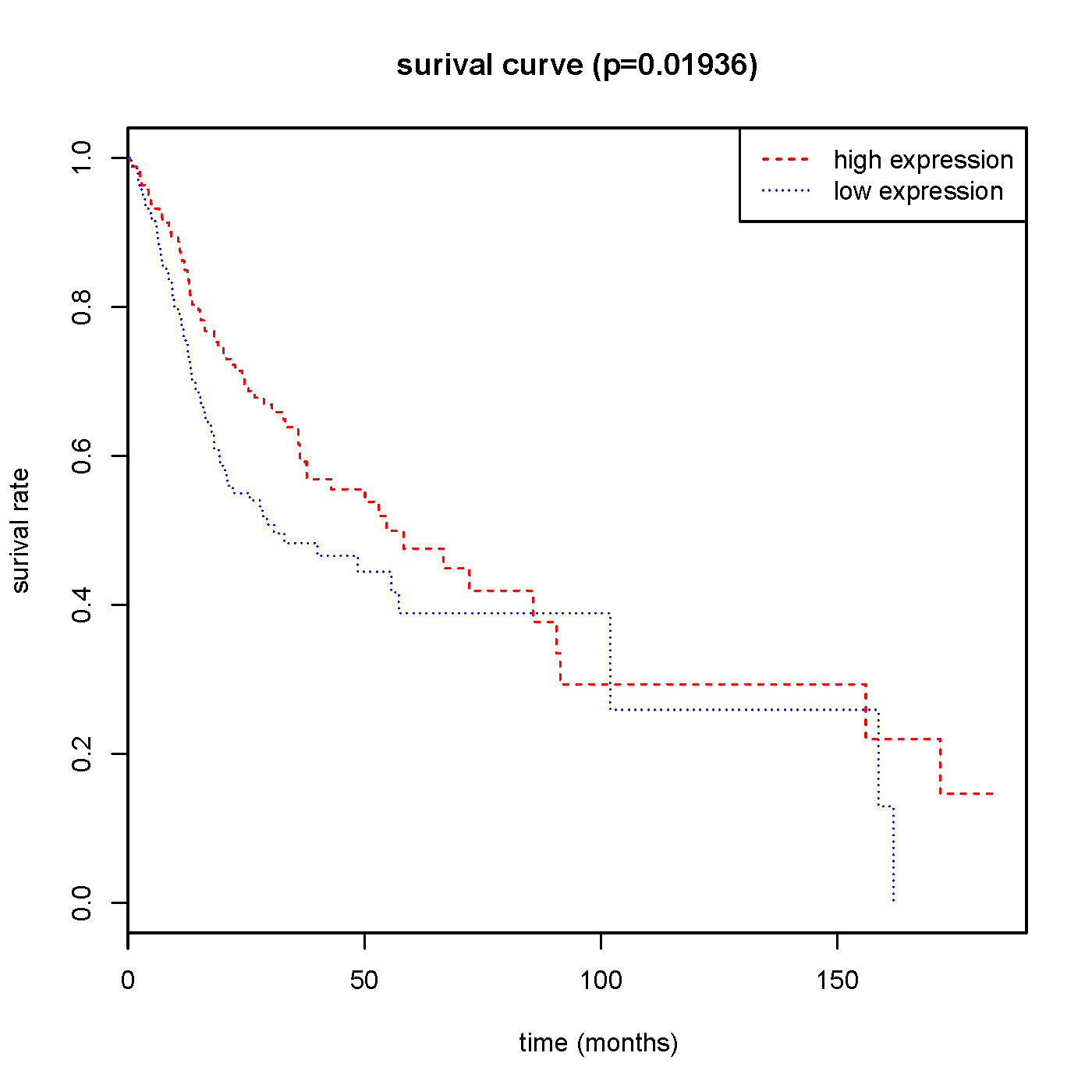

Supplement: Dataset S2 — Kaplan–Meier survival analysis with the log-rank was used to identify relationships between the above 2493 lncRNA signatures and OSCC patient survival. Then, we determined the levels of 151 lncRNA signatures that were significantly related to OS. [file peerj-06-5307-s006.zip › The result of Kaplan–Meier survival analyses and log-rank tests for OS in OSCC/RP11-254F7.1.jpg]

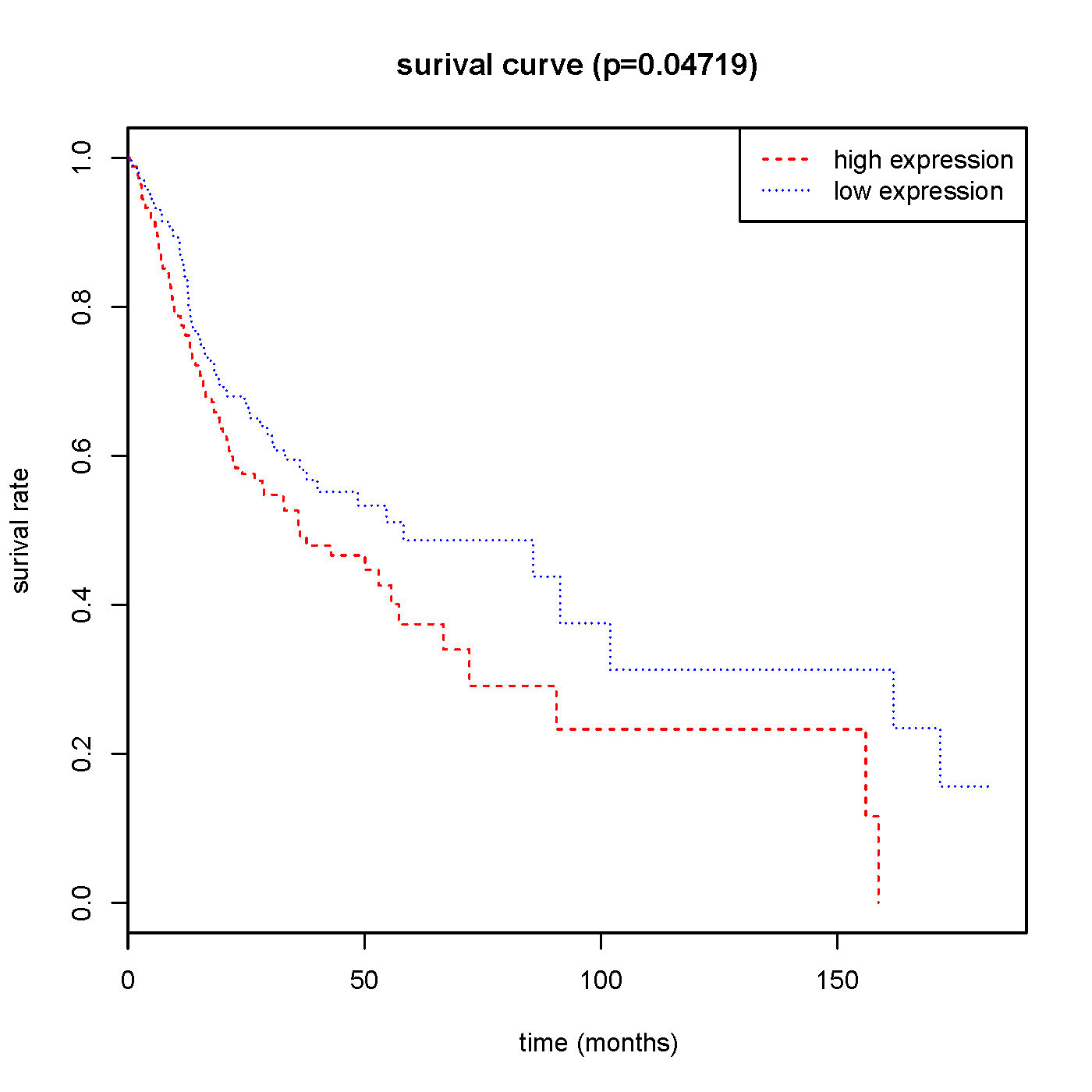

Supplement: Dataset S2 — Kaplan–Meier survival analysis with the log-rank was used to identify relationships between the above 2493 lncRNA signatures and OSCC patient survival. Then, we determined the levels of 151 lncRNA signatures that were significantly related to OS. [file peerj-06-5307-s006.zip › The result of Kaplan–Meier survival analyses and log-rank tests for OS in OSCC/RP11-25I15.3.jpg]

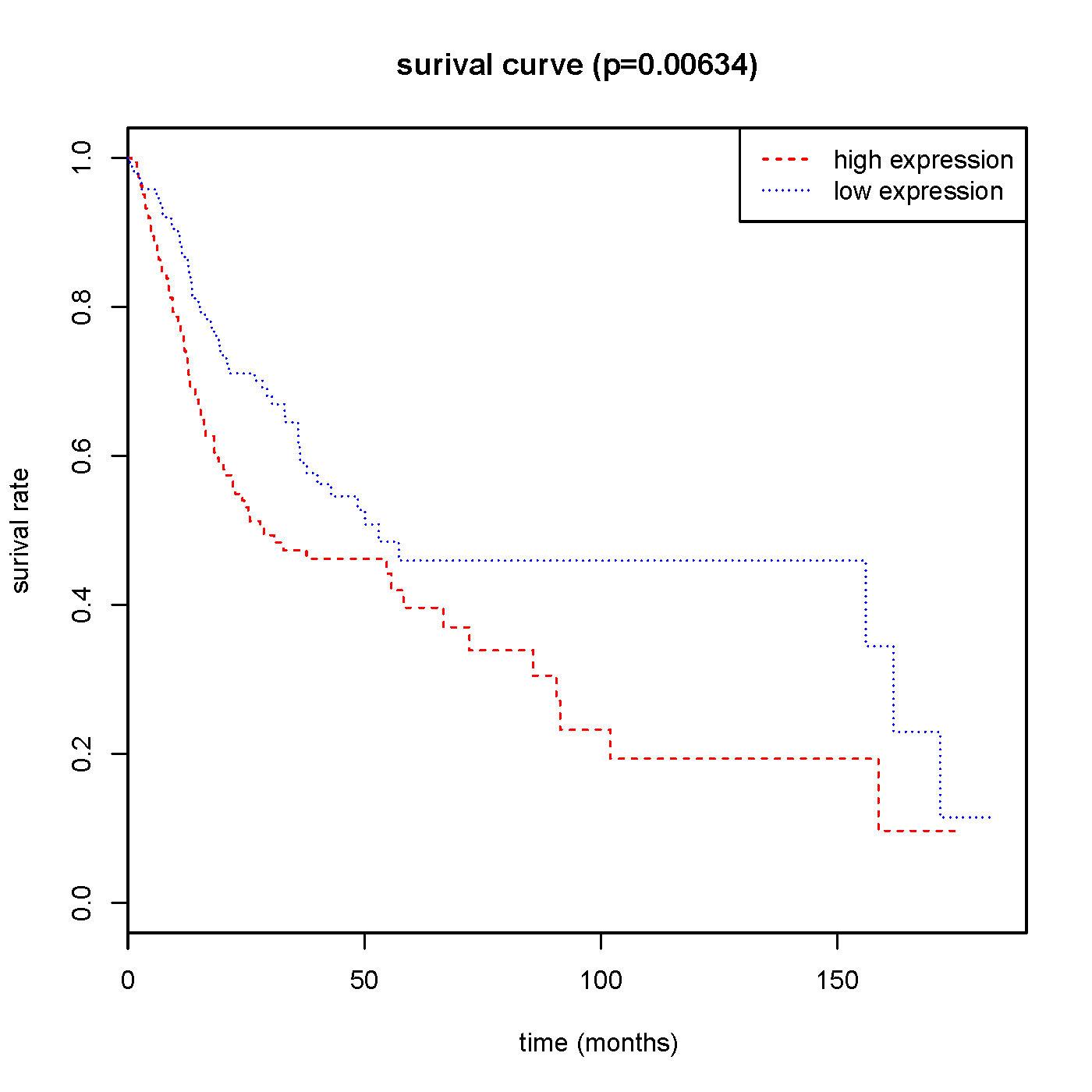

Supplement: Dataset S2 — Kaplan–Meier survival analysis with the log-rank was used to identify relationships between the above 2493 lncRNA signatures and OSCC patient survival. Then, we determined the levels of 151 lncRNA signatures that were significantly related to OS. [file peerj-06-5307-s006.zip › The result of Kaplan–Meier survival analyses and log-rank tests for OS in OSCC/RP11-275N1.1.jpg]

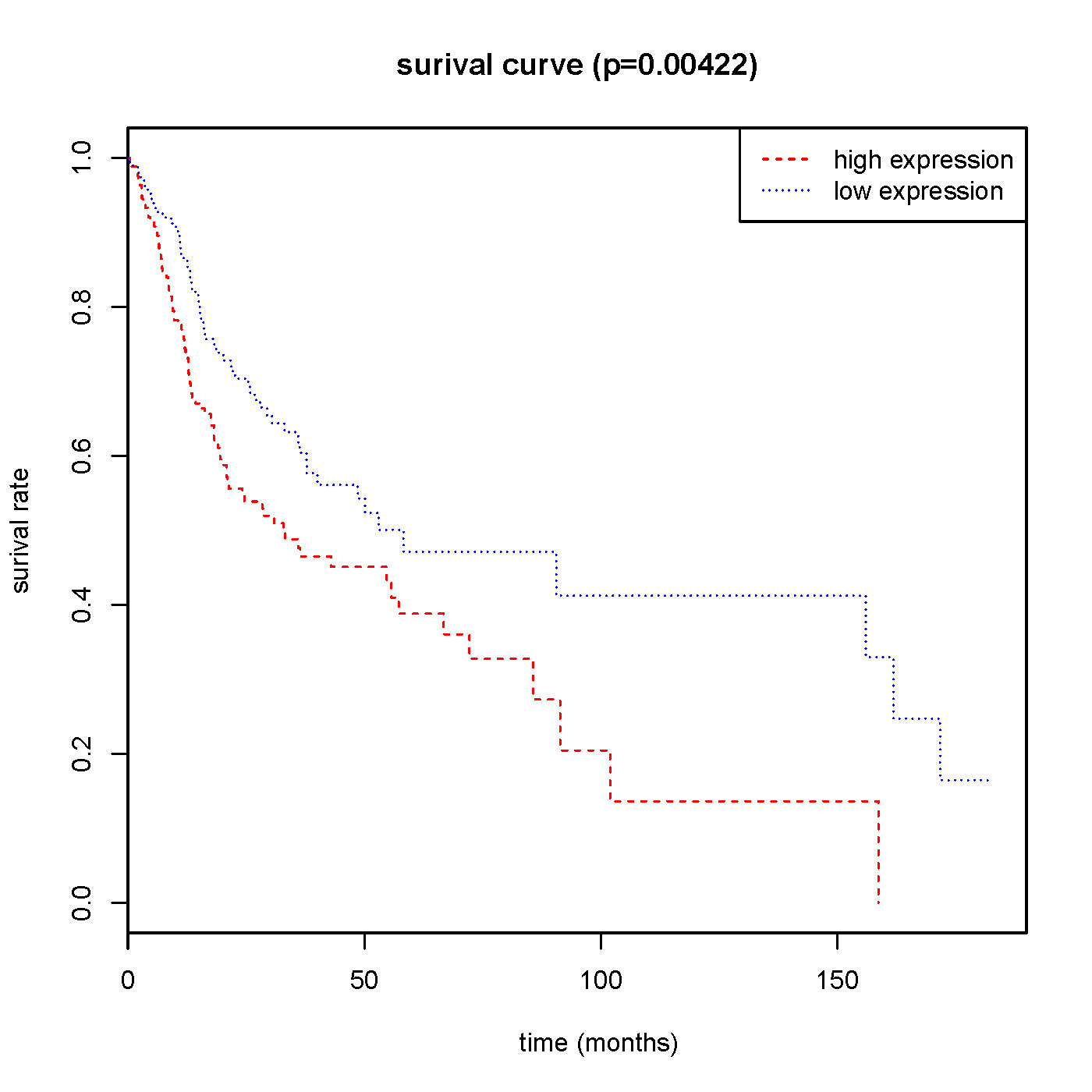

Supplement: Dataset S2 — Kaplan–Meier survival analysis with the log-rank was used to identify relationships between the above 2493 lncRNA signatures and OSCC patient survival. Then, we determined the levels of 151 lncRNA signatures that were significantly related to OS. [file peerj-06-5307-s006.zip › The result of Kaplan–Meier survival analyses and log-rank tests for OS in OSCC/RP11-279N8.1.jpg]

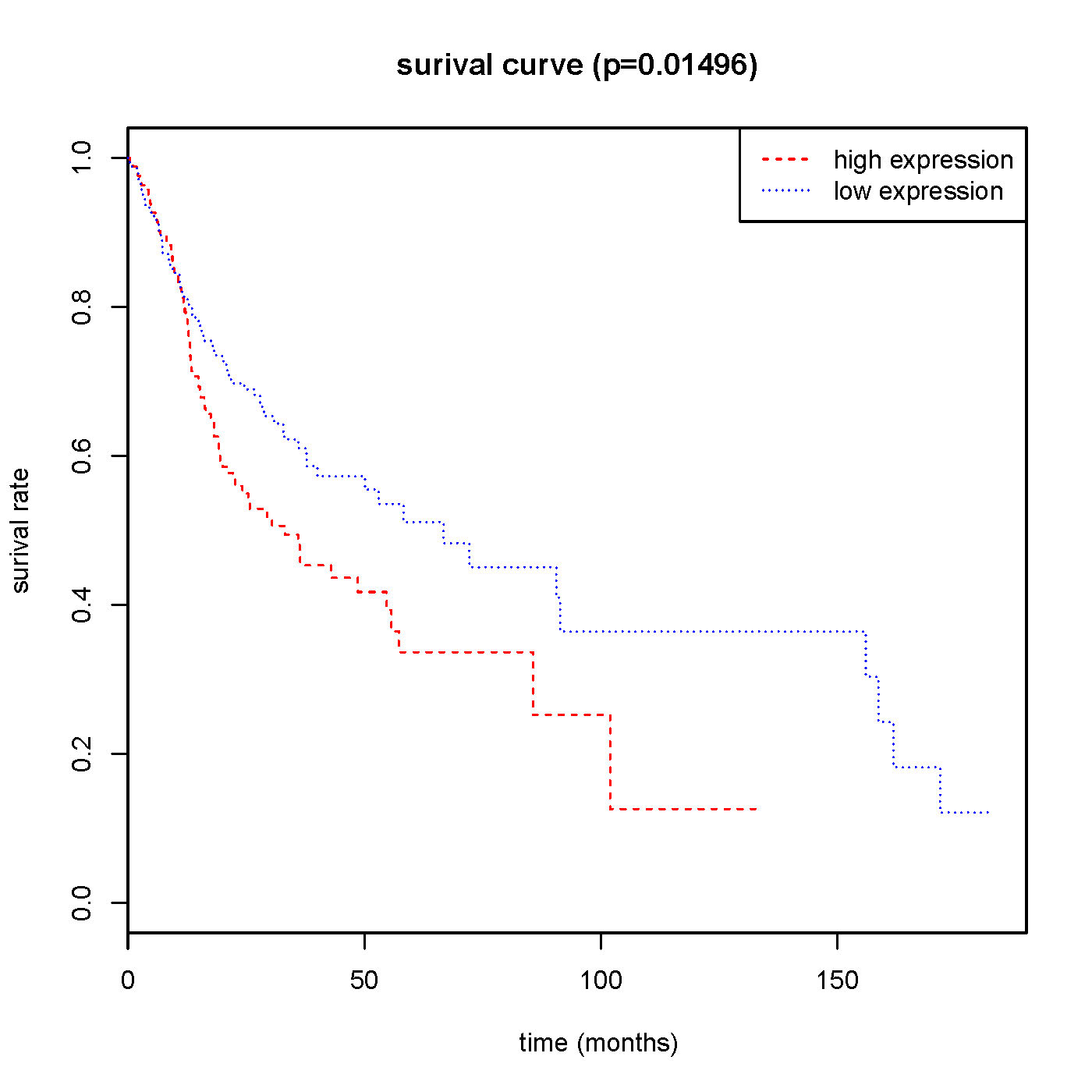

Supplement: Dataset S2 — Kaplan–Meier survival analysis with the log-rank was used to identify relationships between the above 2493 lncRNA signatures and OSCC patient survival. Then, we determined the levels of 151 lncRNA signatures that were significantly related to OS. [file peerj-06-5307-s006.zip › The result of Kaplan–Meier survival analyses and log-rank tests for OS in OSCC/RP11-281P23.2.jpg]

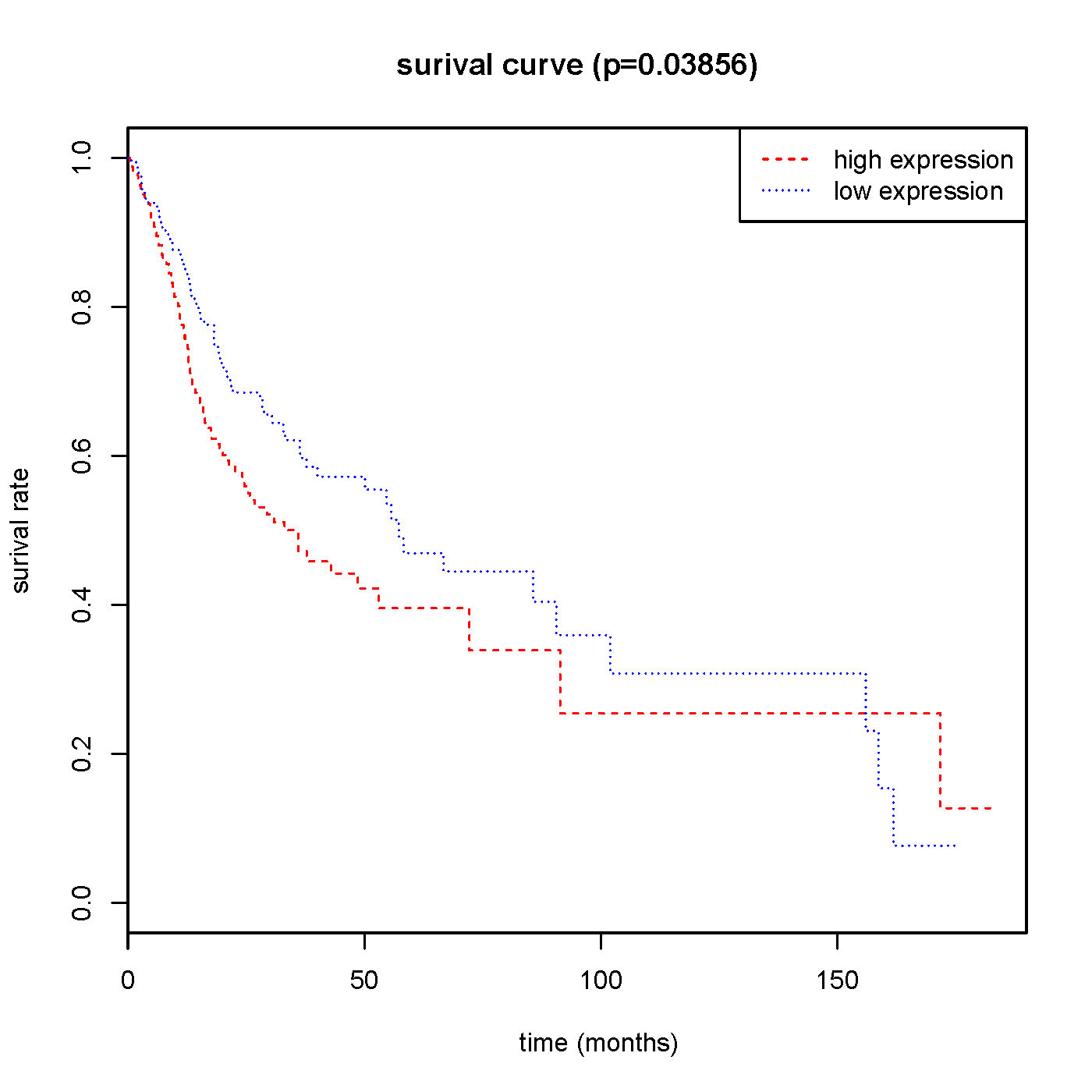

Supplement: Dataset S2 — Kaplan–Meier survival analysis with the log-rank was used to identify relationships between the above 2493 lncRNA signatures and OSCC patient survival. Then, we determined the levels of 151 lncRNA signatures that were significantly related to OS. [file peerj-06-5307-s006.zip › The result of Kaplan–Meier survival analyses and log-rank tests for OS in OSCC/RP11-285F16.1.jpg]

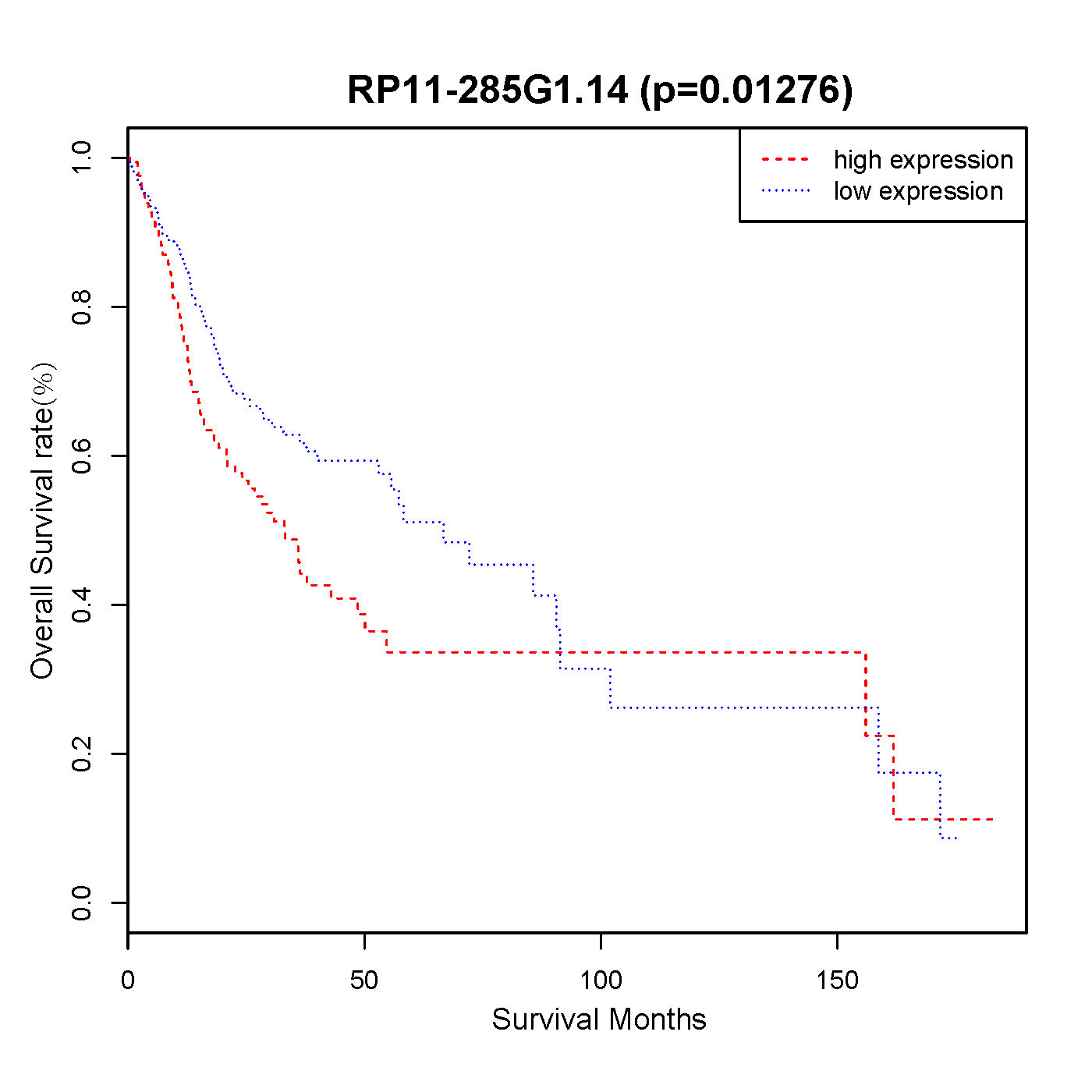

Supplement: Dataset S2 — Kaplan–Meier survival analysis with the log-rank was used to identify relationships between the above 2493 lncRNA signatures and OSCC patient survival. Then, we determined the levels of 151 lncRNA signatures that were significantly related to OS. [file peerj-06-5307-s006.zip › The result of Kaplan–Meier survival analyses and log-rank tests for OS in OSCC/RP11-285G1.14.jpg]

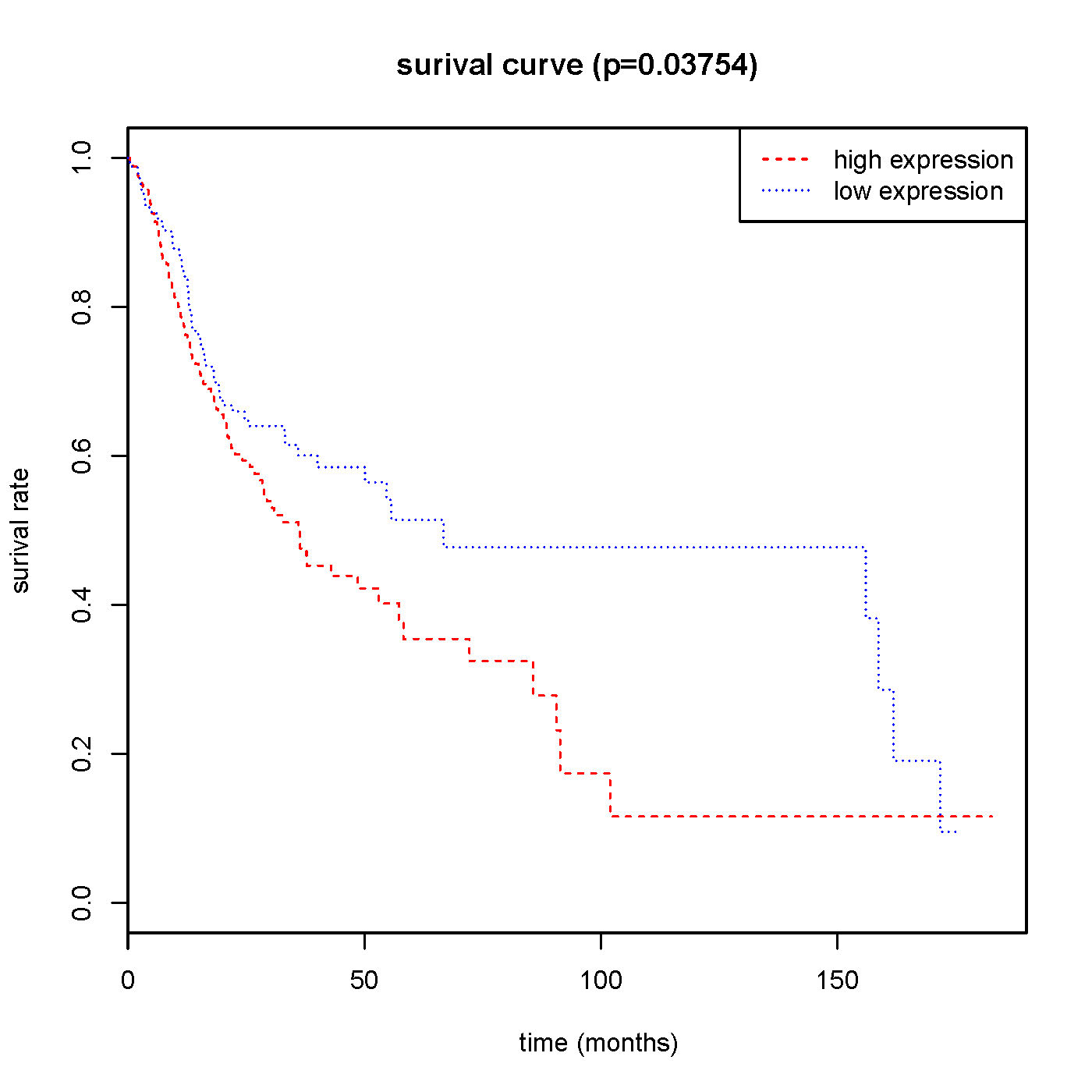

Supplement: Dataset S2 — Kaplan–Meier survival analysis with the log-rank was used to identify relationships between the above 2493 lncRNA signatures and OSCC patient survival. Then, we determined the levels of 151 lncRNA signatures that were significantly related to OS. [file peerj-06-5307-s006.zip › The result of Kaplan–Meier survival analyses and log-rank tests for OS in OSCC/RP11-286B14.1.jpg]

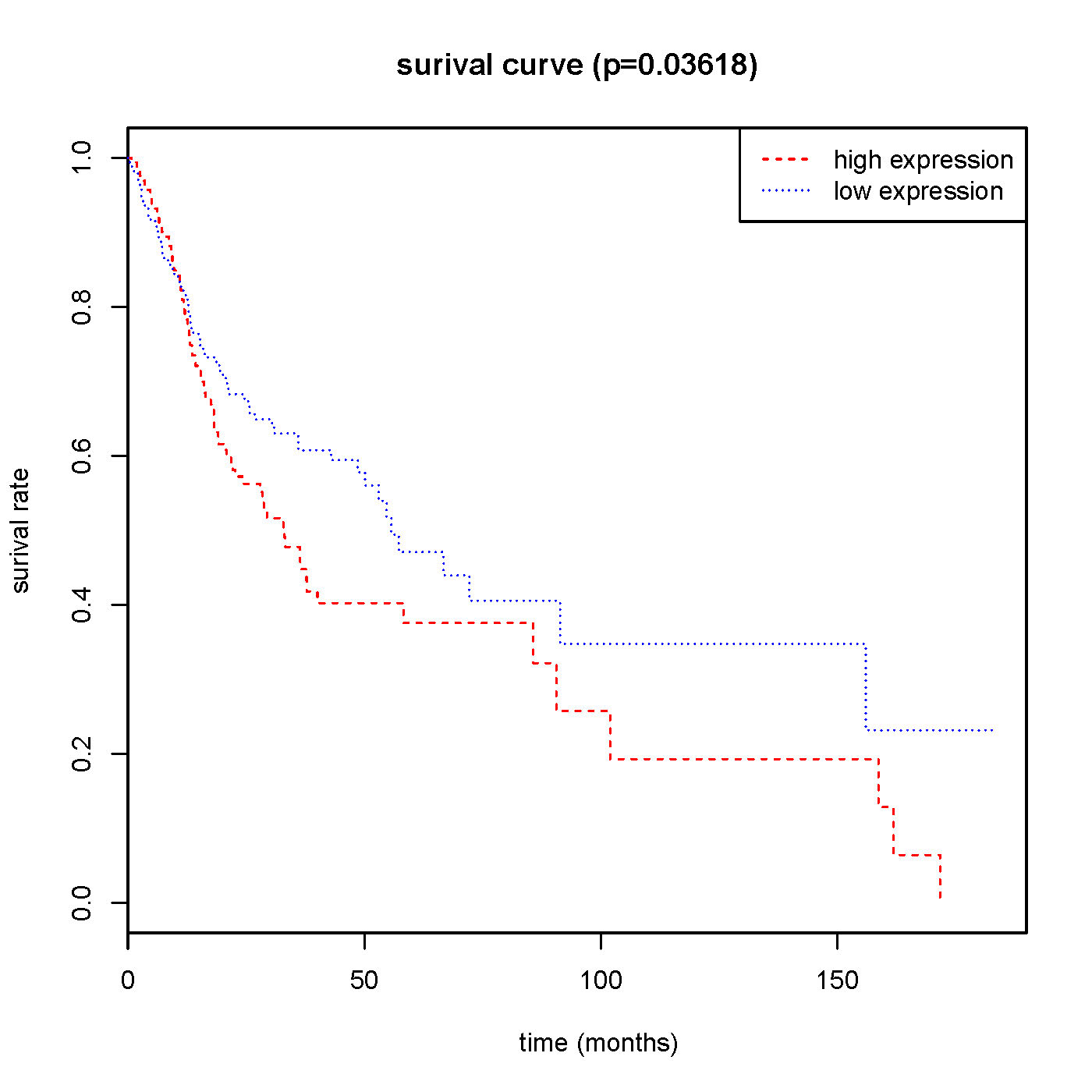

Supplement: Dataset S2 — Kaplan–Meier survival analysis with the log-rank was used to identify relationships between the above 2493 lncRNA signatures and OSCC patient survival. Then, we determined the levels of 151 lncRNA signatures that were significantly related to OS. [file peerj-06-5307-s006.zip › The result of Kaplan–Meier survival analyses and log-rank tests for OS in OSCC/RP11-286E11.1.jpg]

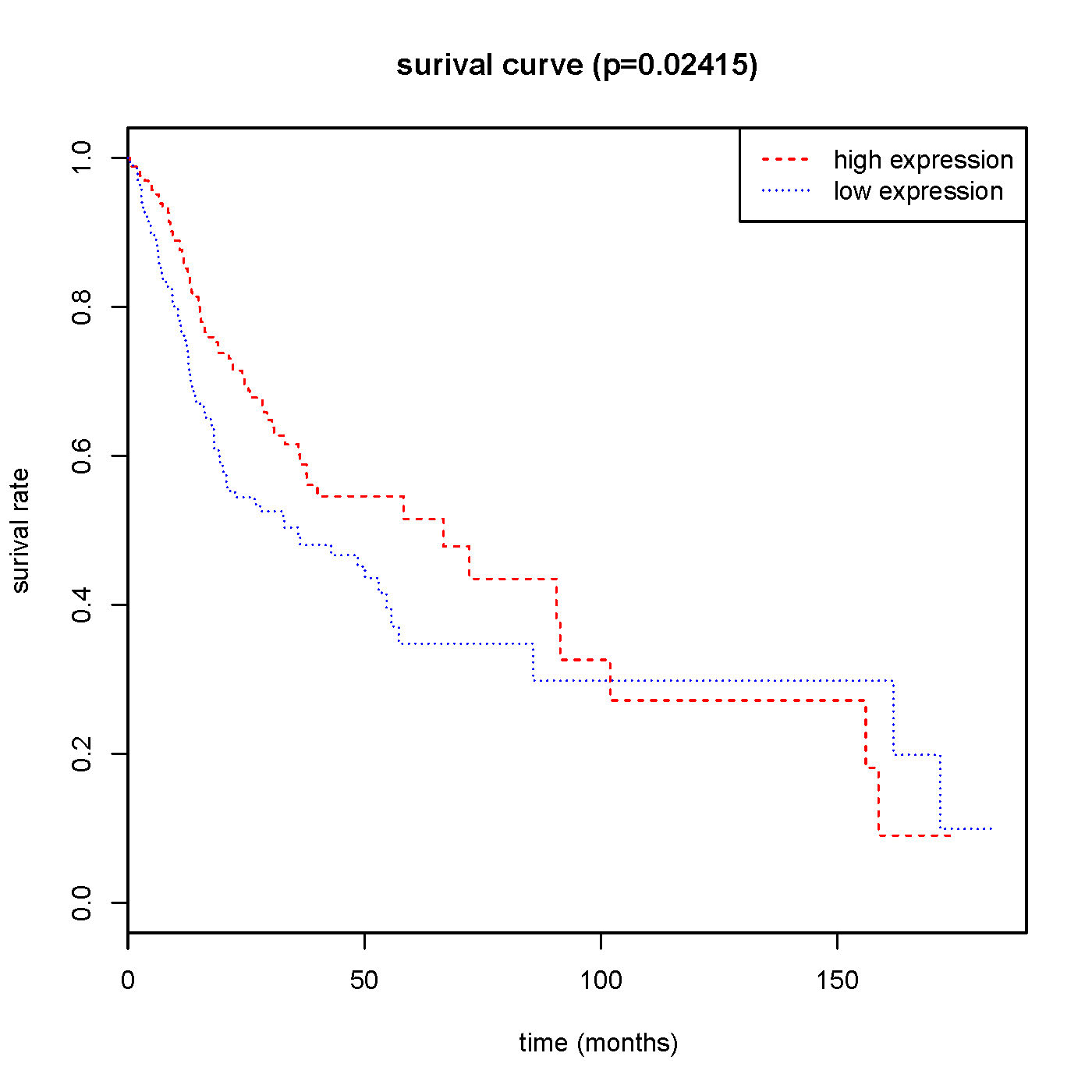

Supplement: Dataset S2 — Kaplan–Meier survival analysis with the log-rank was used to identify relationships between the above 2493 lncRNA signatures and OSCC patient survival. Then, we determined the levels of 151 lncRNA signatures that were significantly related to OS. [file peerj-06-5307-s006.zip › The result of Kaplan–Meier survival analyses and log-rank tests for OS in OSCC/RP11-30K9.5.jpg]

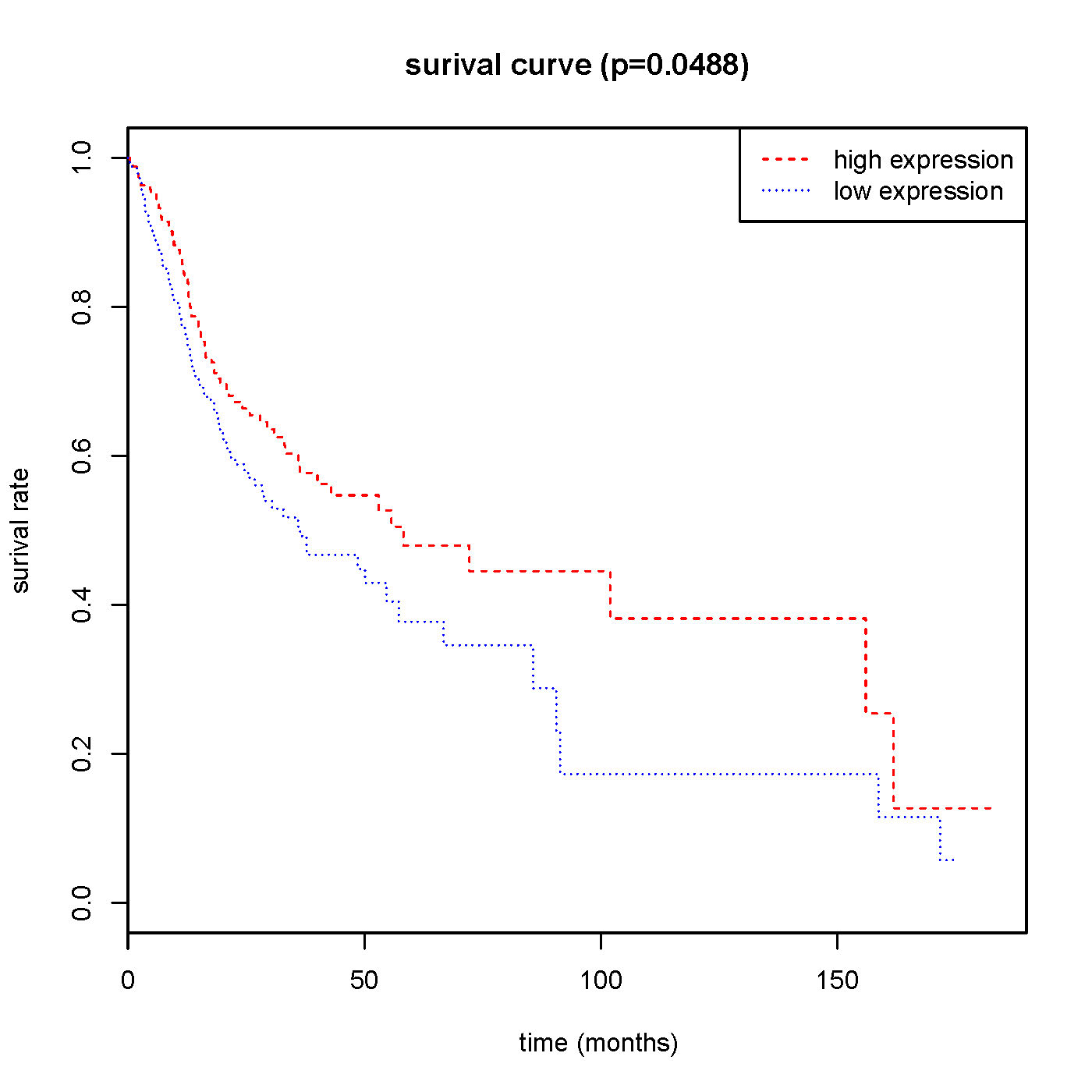

Supplement: Dataset S2 — Kaplan–Meier survival analysis with the log-rank was used to identify relationships between the above 2493 lncRNA signatures and OSCC patient survival. Then, we determined the levels of 151 lncRNA signatures that were significantly related to OS. [file peerj-06-5307-s006.zip › The result of Kaplan–Meier survival analyses and log-rank tests for OS in OSCC/RP11-318C2.1.jpg]

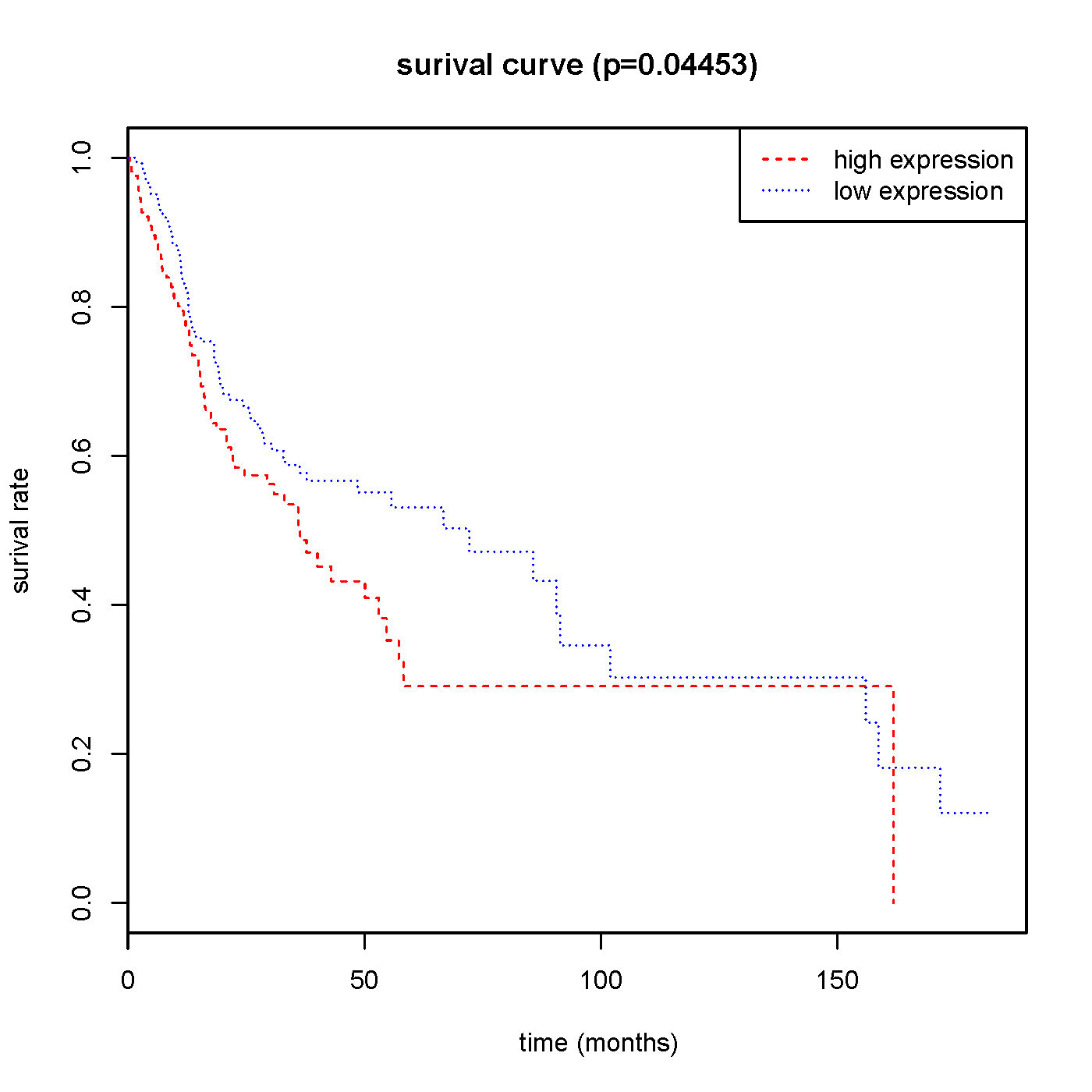

Supplement: Dataset S2 — Kaplan–Meier survival analysis with the log-rank was used to identify relationships between the above 2493 lncRNA signatures and OSCC patient survival. Then, we determined the levels of 151 lncRNA signatures that were significantly related to OS. [file peerj-06-5307-s006.zip › The result of Kaplan–Meier survival analyses and log-rank tests for OS in OSCC/RP11-325L12.7.jpg]

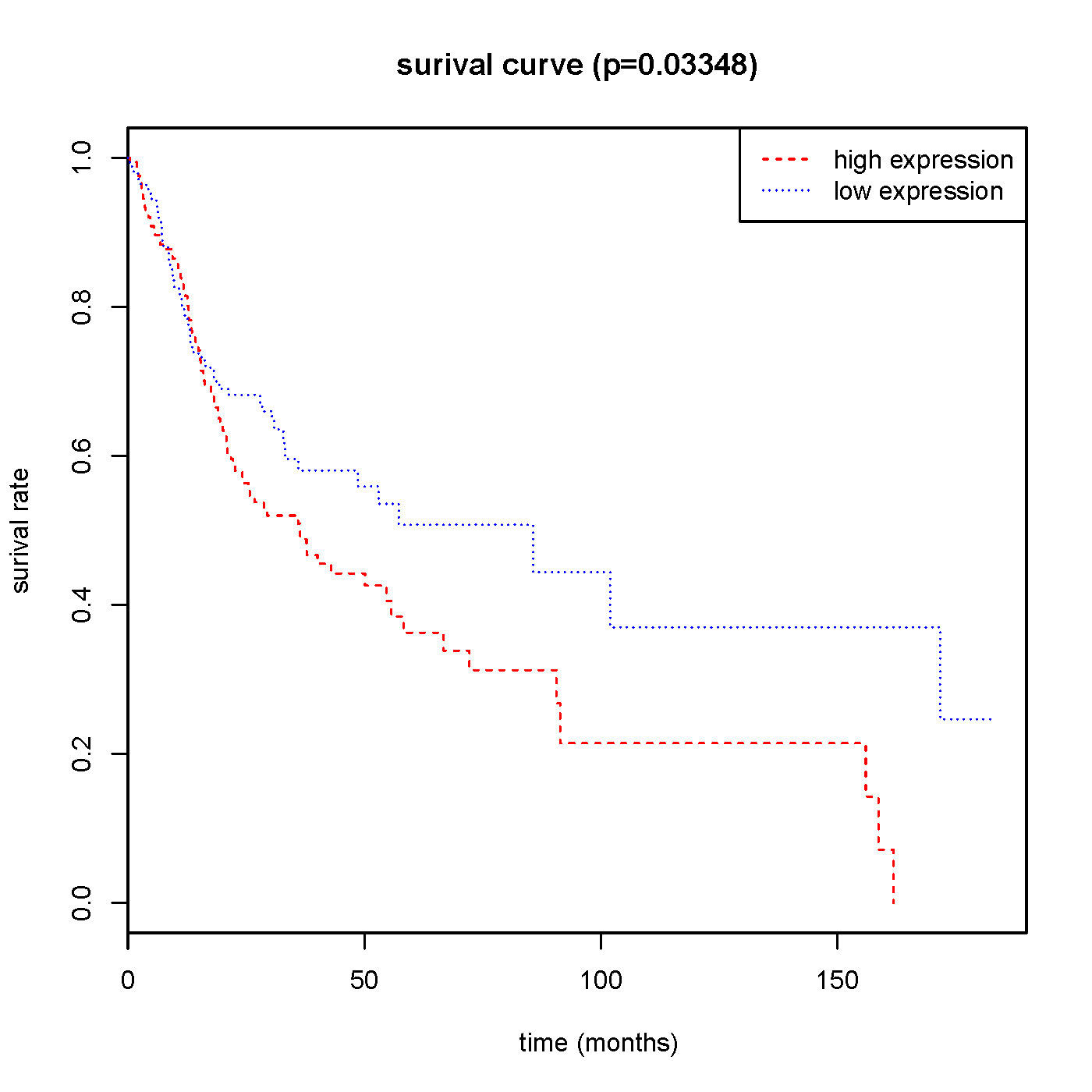

Supplement: Dataset S2 — Kaplan–Meier survival analysis with the log-rank was used to identify relationships between the above 2493 lncRNA signatures and OSCC patient survival. Then, we determined the levels of 151 lncRNA signatures that were significantly related to OS. [file peerj-06-5307-s006.zip › The result of Kaplan–Meier survival analyses and log-rank tests for OS in OSCC/RP11-335K5.2.jpg]

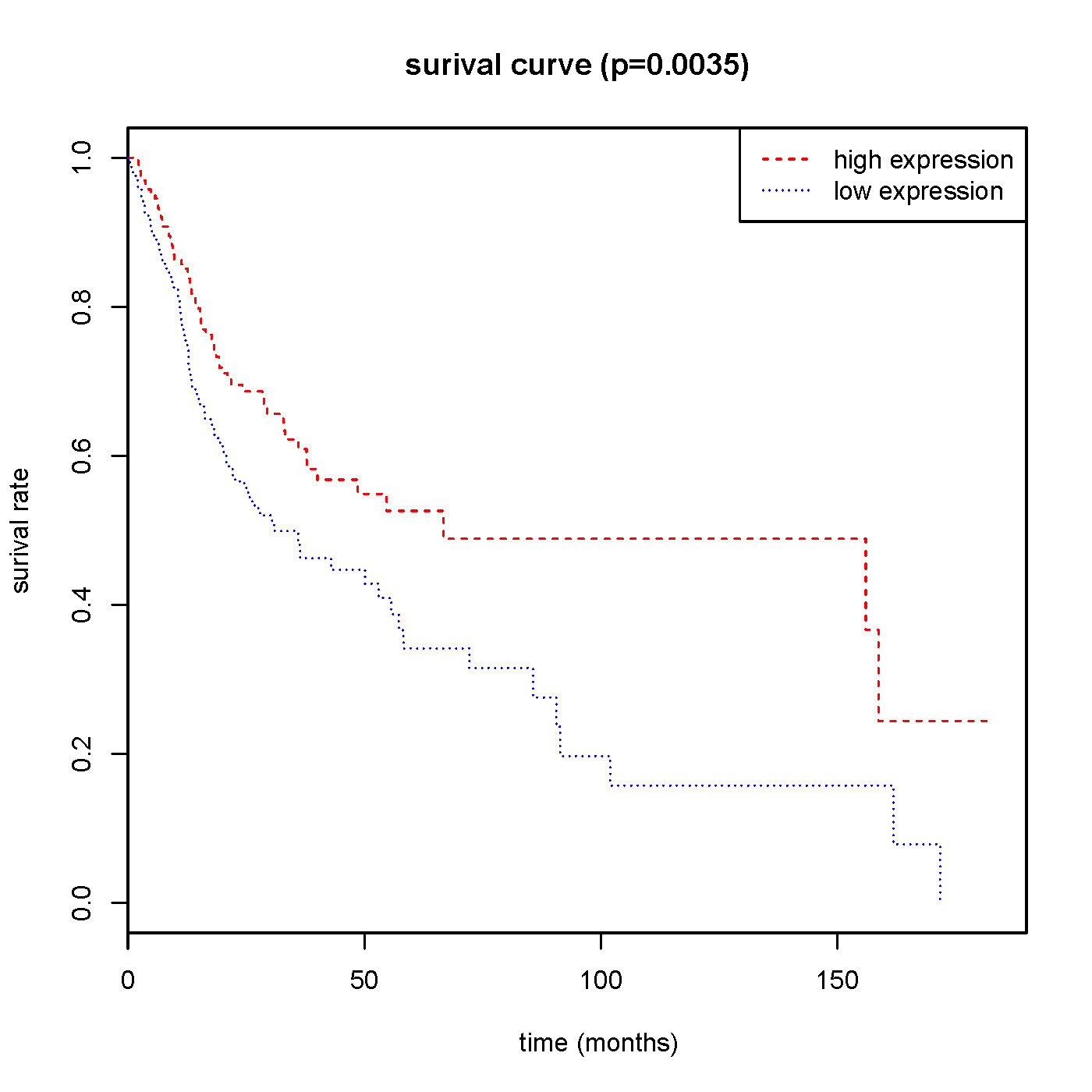

Supplement: Dataset S2 — Kaplan–Meier survival analysis with the log-rank was used to identify relationships between the above 2493 lncRNA signatures and OSCC patient survival. Then, we determined the levels of 151 lncRNA signatures that were significantly related to OS. [file peerj-06-5307-s006.zip › The result of Kaplan–Meier survival analyses and log-rank tests for OS in OSCC/RP11-356I2.1.jpg]

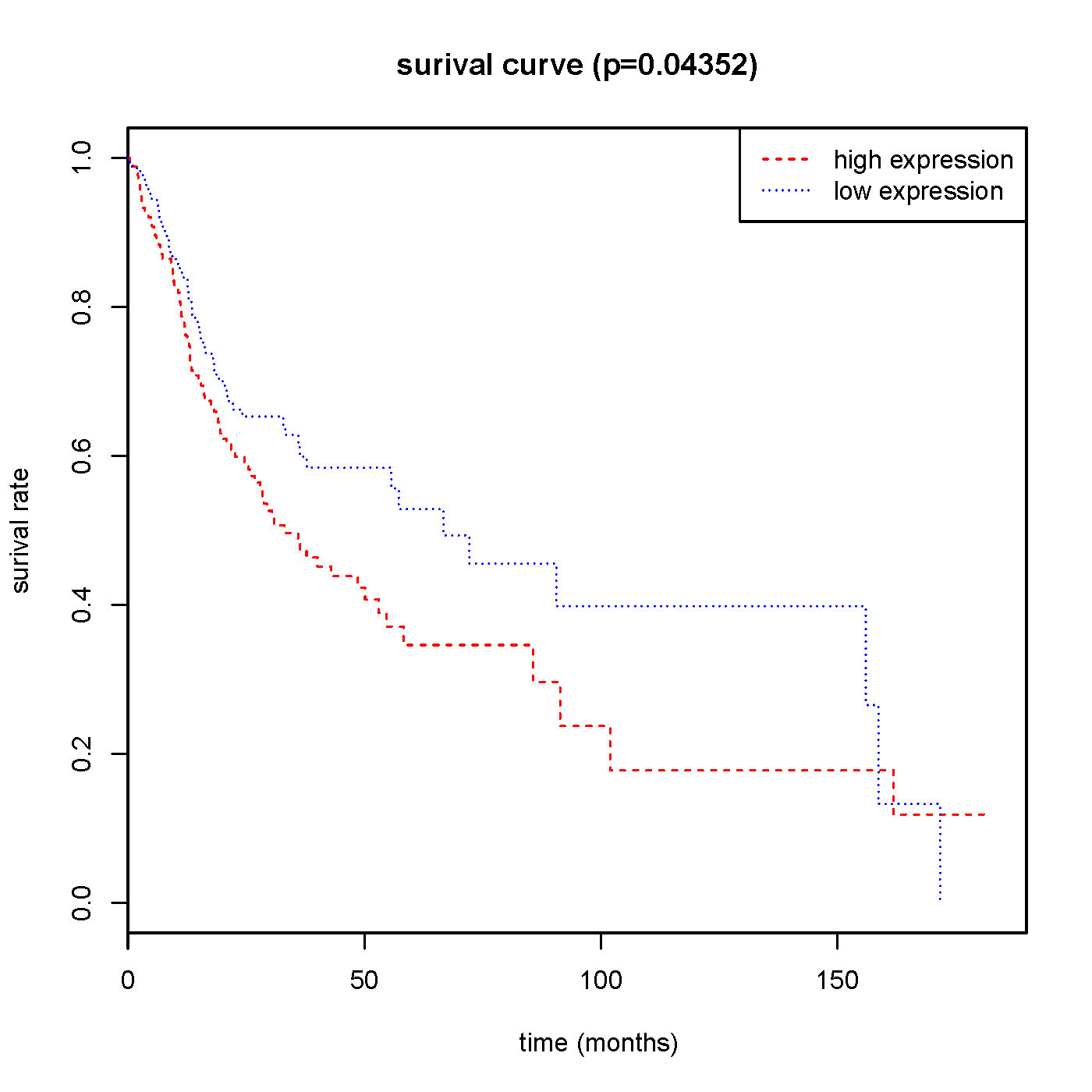

Supplement: Dataset S2 — Kaplan–Meier survival analysis with the log-rank was used to identify relationships between the above 2493 lncRNA signatures and OSCC patient survival. Then, we determined the levels of 151 lncRNA signatures that were significantly related to OS. [file peerj-06-5307-s006.zip › The result of Kaplan–Meier survival analyses and log-rank tests for OS in OSCC/RP11-367J11.3.jpg]

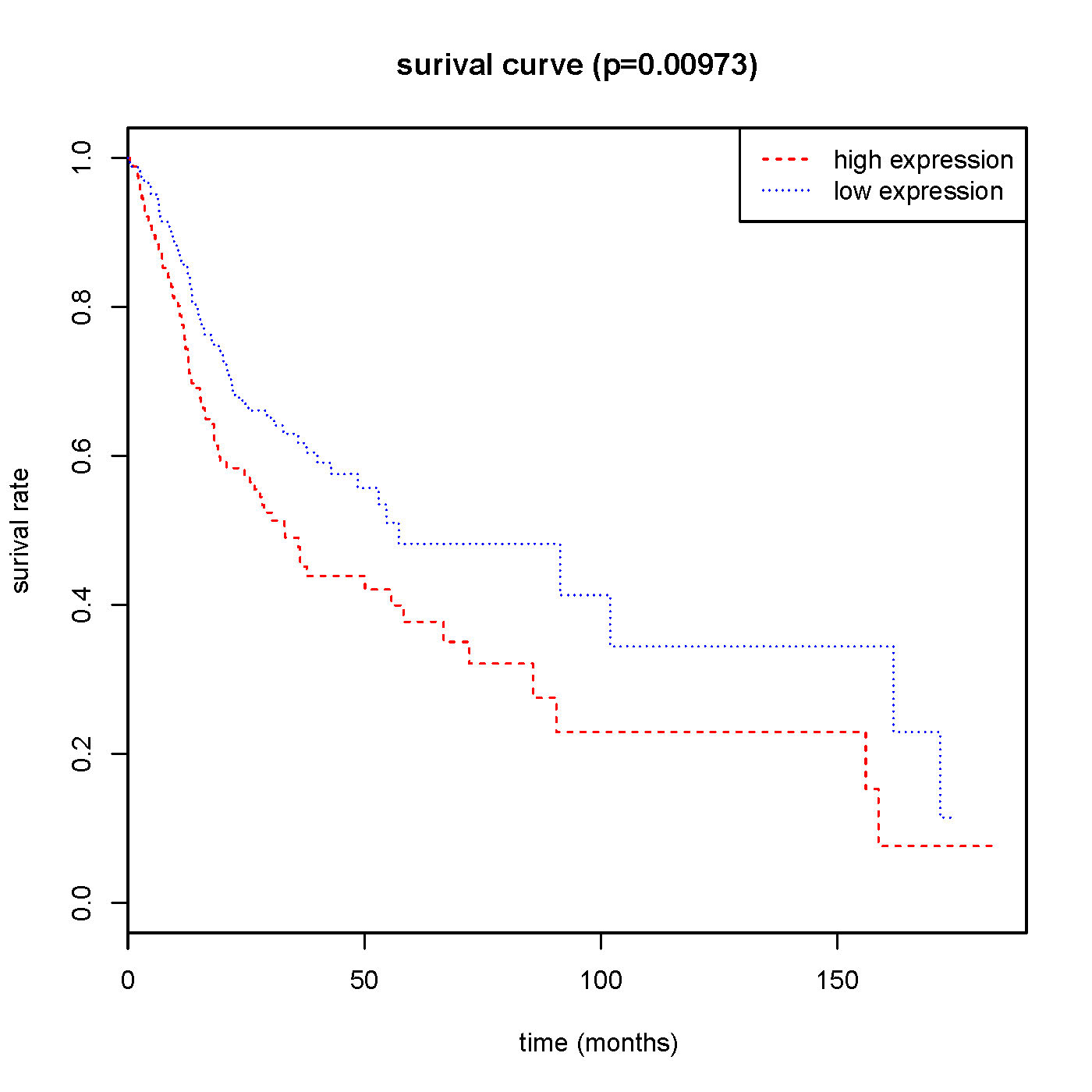

Supplement: Dataset S2 — Kaplan–Meier survival analysis with the log-rank was used to identify relationships between the above 2493 lncRNA signatures and OSCC patient survival. Then, we determined the levels of 151 lncRNA signatures that were significantly related to OS. [file peerj-06-5307-s006.zip › The result of Kaplan–Meier survival analyses and log-rank tests for OS in OSCC/RP11-386G11.5.jpg]

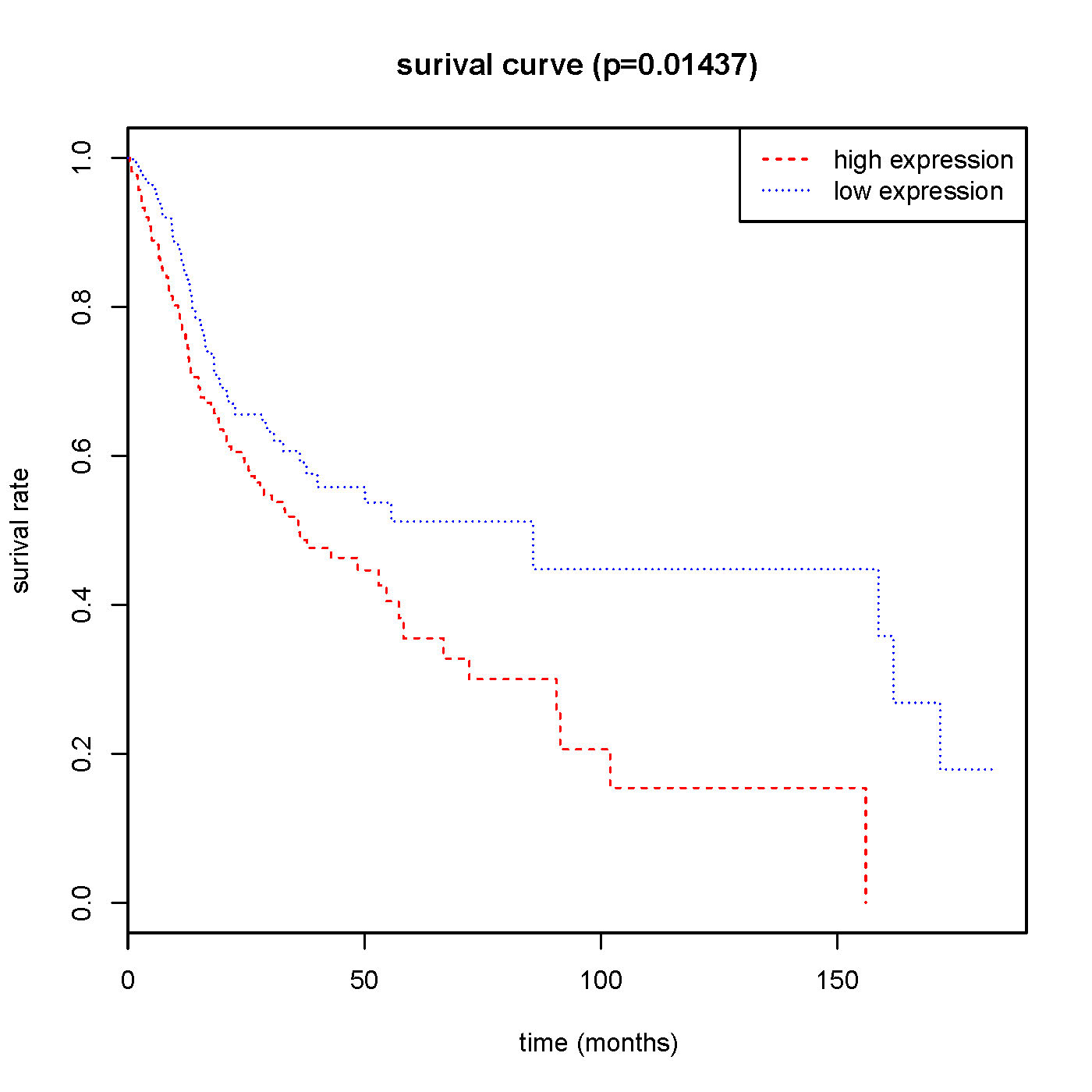

Supplement: Dataset S2 — Kaplan–Meier survival analysis with the log-rank was used to identify relationships between the above 2493 lncRNA signatures and OSCC patient survival. Then, we determined the levels of 151 lncRNA signatures that were significantly related to OS. [file peerj-06-5307-s006.zip › The result of Kaplan–Meier survival analyses and log-rank tests for OS in OSCC/RP11-390N6.1.jpg]

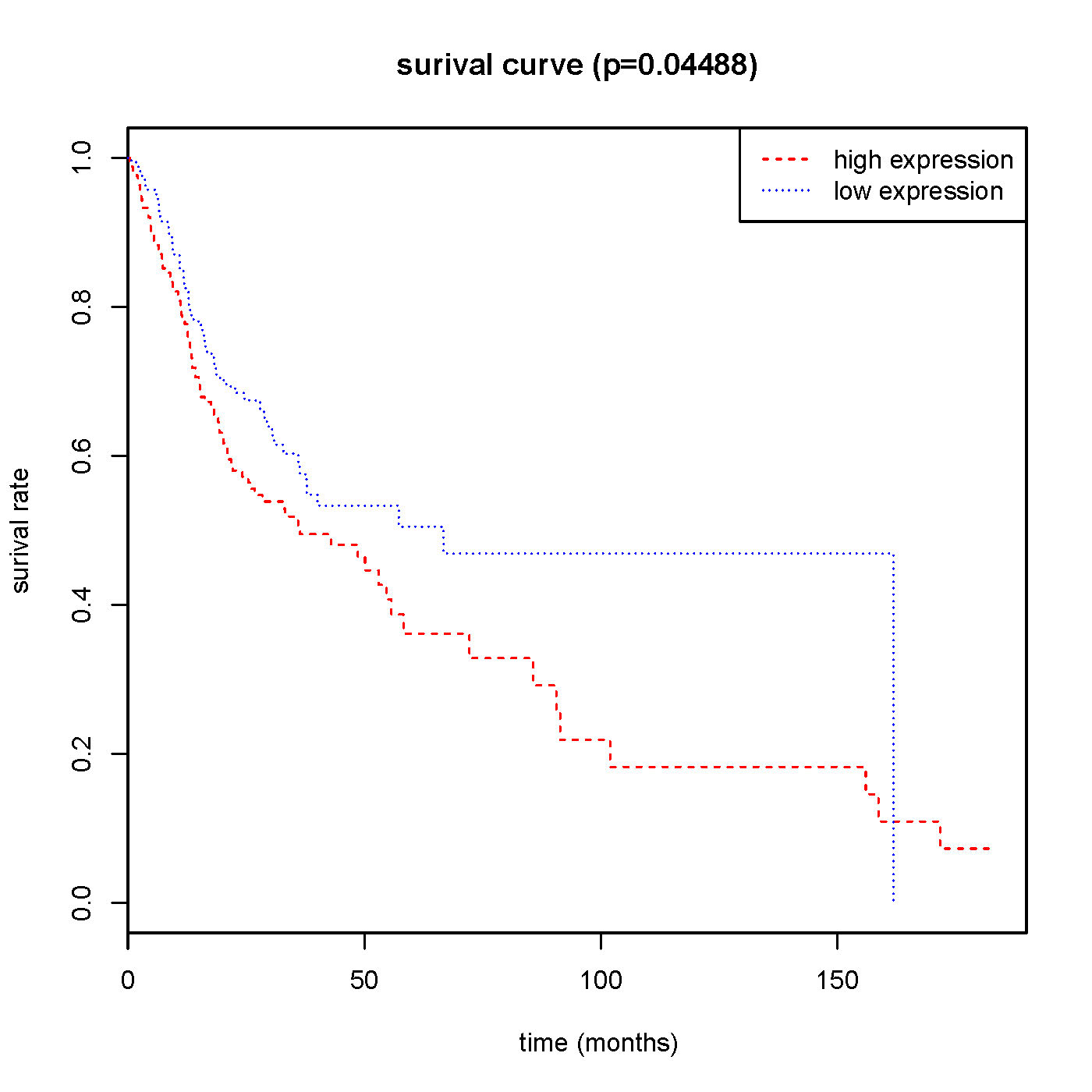

Supplement: Dataset S2 — Kaplan–Meier survival analysis with the log-rank was used to identify relationships between the above 2493 lncRNA signatures and OSCC patient survival. Then, we determined the levels of 151 lncRNA signatures that were significantly related to OS. [file peerj-06-5307-s006.zip › The result of Kaplan–Meier survival analyses and log-rank tests for OS in OSCC/RP11-397A16.1.jpg]

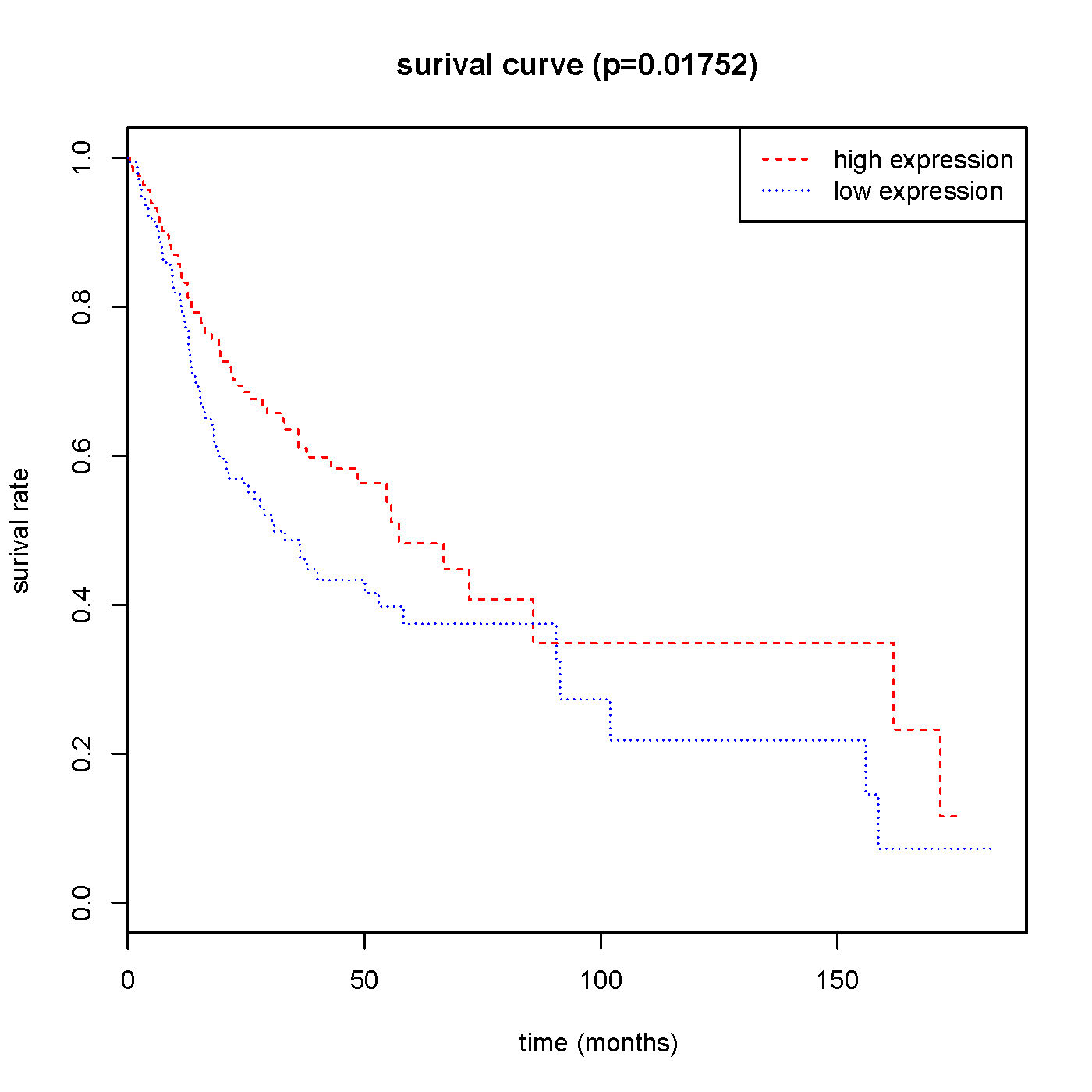

Supplement: Dataset S2 — Kaplan–Meier survival analysis with the log-rank was used to identify relationships between the above 2493 lncRNA signatures and OSCC patient survival. Then, we determined the levels of 151 lncRNA signatures that were significantly related to OS. [file peerj-06-5307-s006.zip › The result of Kaplan–Meier survival analyses and log-rank tests for OS in OSCC/RP11-404F10.2.jpg]

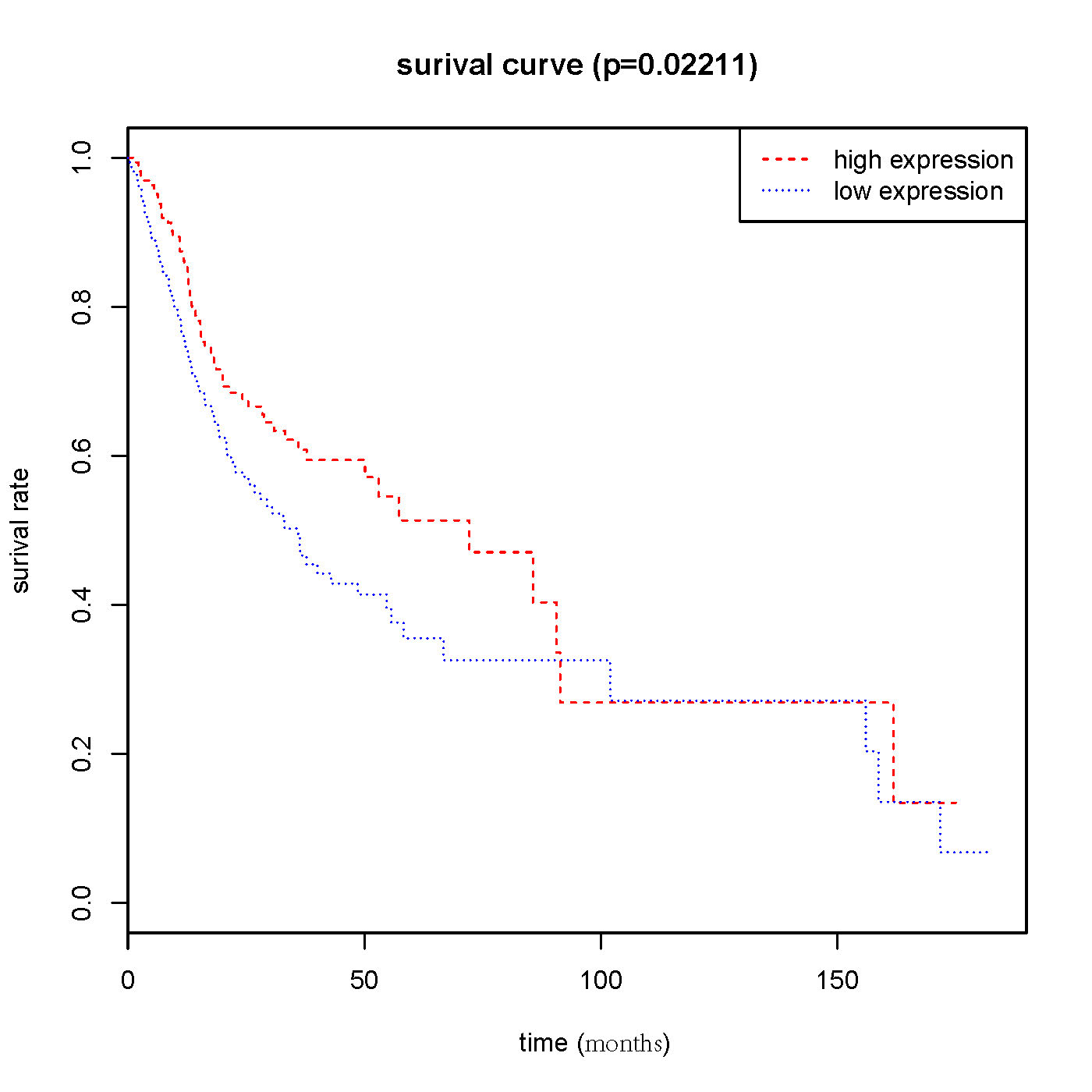

Supplement: Dataset S2 — Kaplan–Meier survival analysis with the log-rank was used to identify relationships between the above 2493 lncRNA signatures and OSCC patient survival. Then, we determined the levels of 151 lncRNA signatures that were significantly related to OS. [file peerj-06-5307-s006.zip › The result of Kaplan–Meier survival analyses and log-rank tests for OS in OSCC/RP11-43A14.1.jpg]

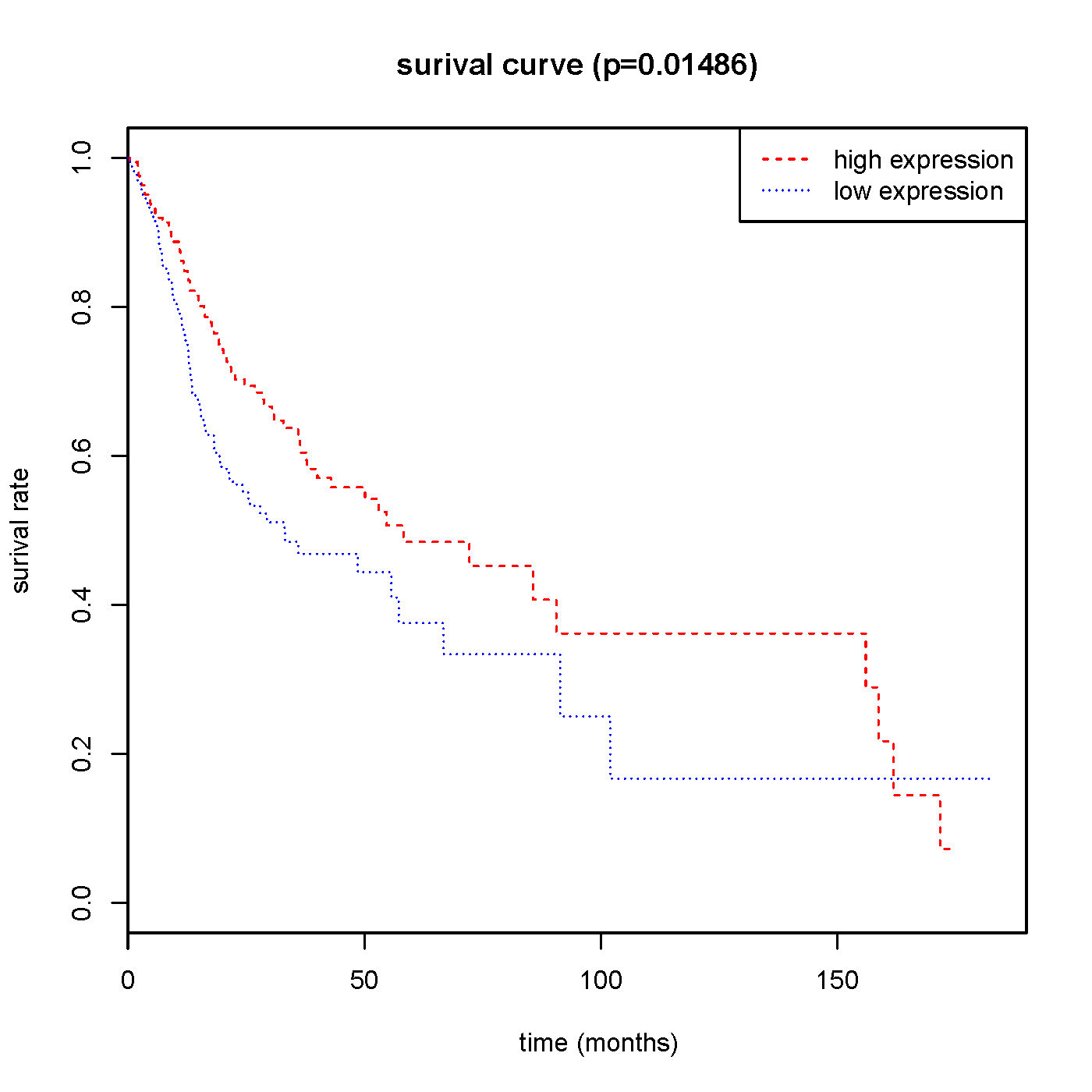

Supplement: Dataset S2 — Kaplan–Meier survival analysis with the log-rank was used to identify relationships between the above 2493 lncRNA signatures and OSCC patient survival. Then, we determined the levels of 151 lncRNA signatures that were significantly related to OS. [file peerj-06-5307-s006.zip › The result of Kaplan–Meier survival analyses and log-rank tests for OS in OSCC/RP11-464F9.21.jpg]

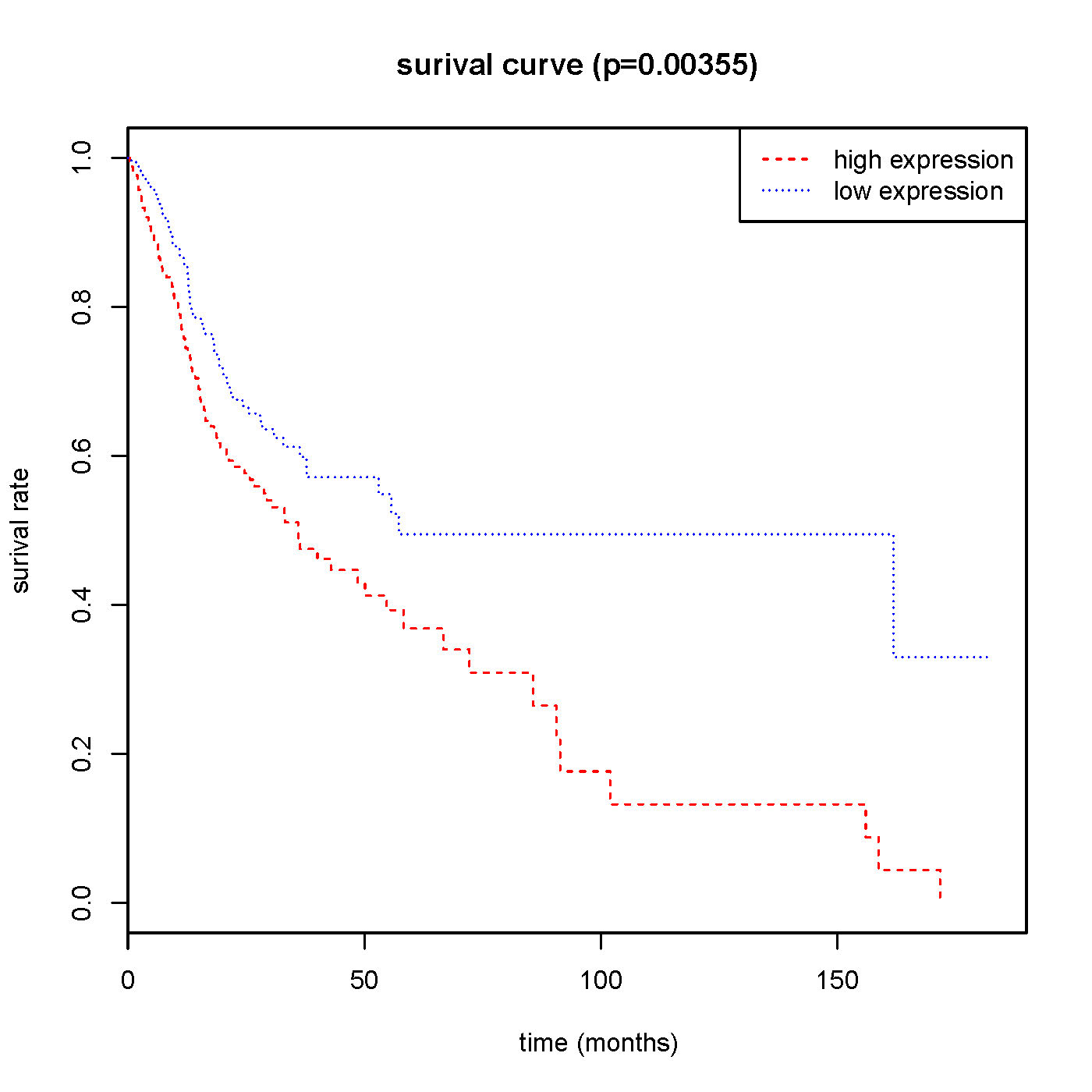

Supplement: Dataset S2 — Kaplan–Meier survival analysis with the log-rank was used to identify relationships between the above 2493 lncRNA signatures and OSCC patient survival. Then, we determined the levels of 151 lncRNA signatures that were significantly related to OS. [file peerj-06-5307-s006.zip › The result of Kaplan–Meier survival analyses and log-rank tests for OS in OSCC/RP11-499F3.2.jpg]

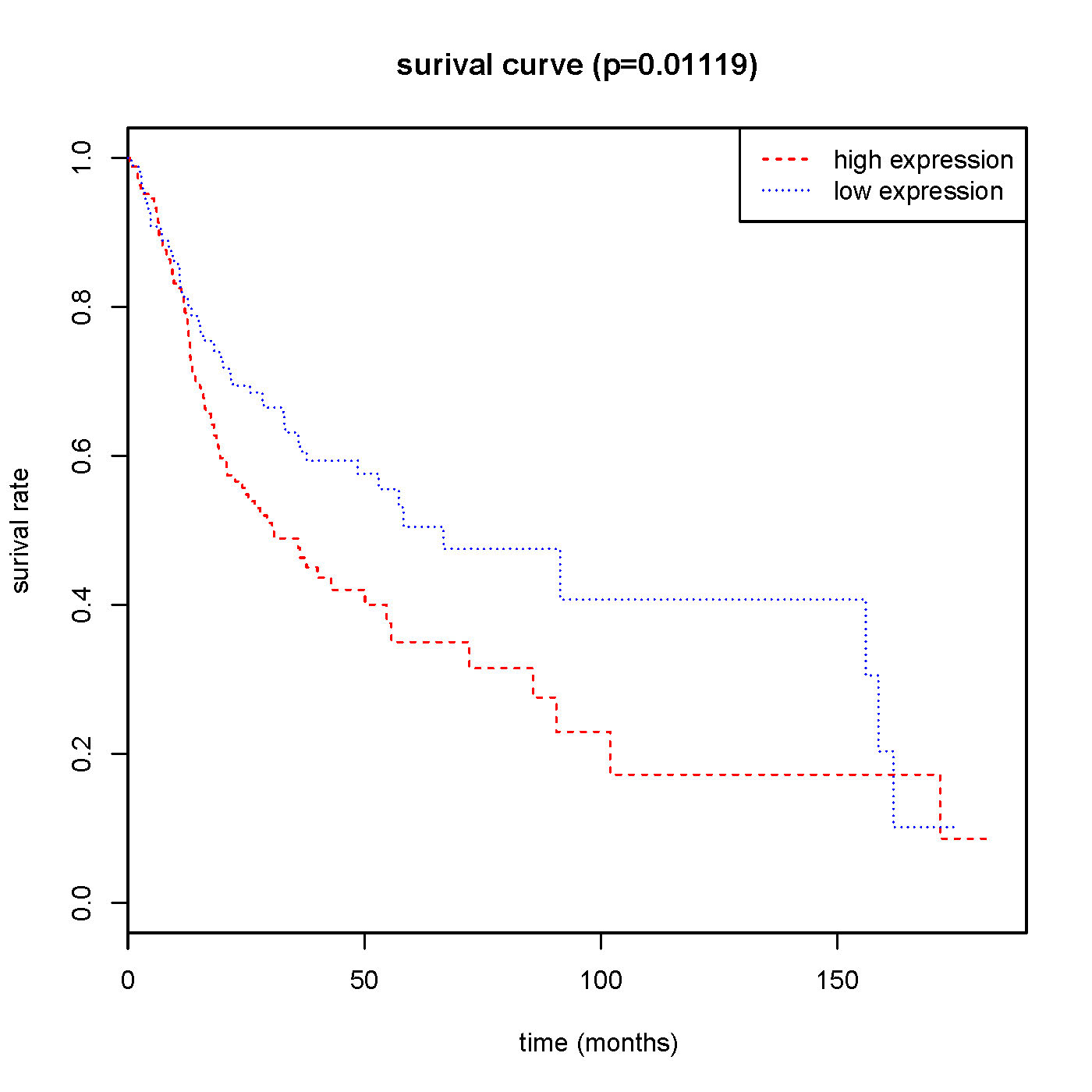

Supplement: Dataset S2 — Kaplan–Meier survival analysis with the log-rank was used to identify relationships between the above 2493 lncRNA signatures and OSCC patient survival. Then, we determined the levels of 151 lncRNA signatures that were significantly related to OS. [file peerj-06-5307-s006.zip › The result of Kaplan–Meier survival analyses and log-rank tests for OS in OSCC/RP11-532M24.1.jpg]

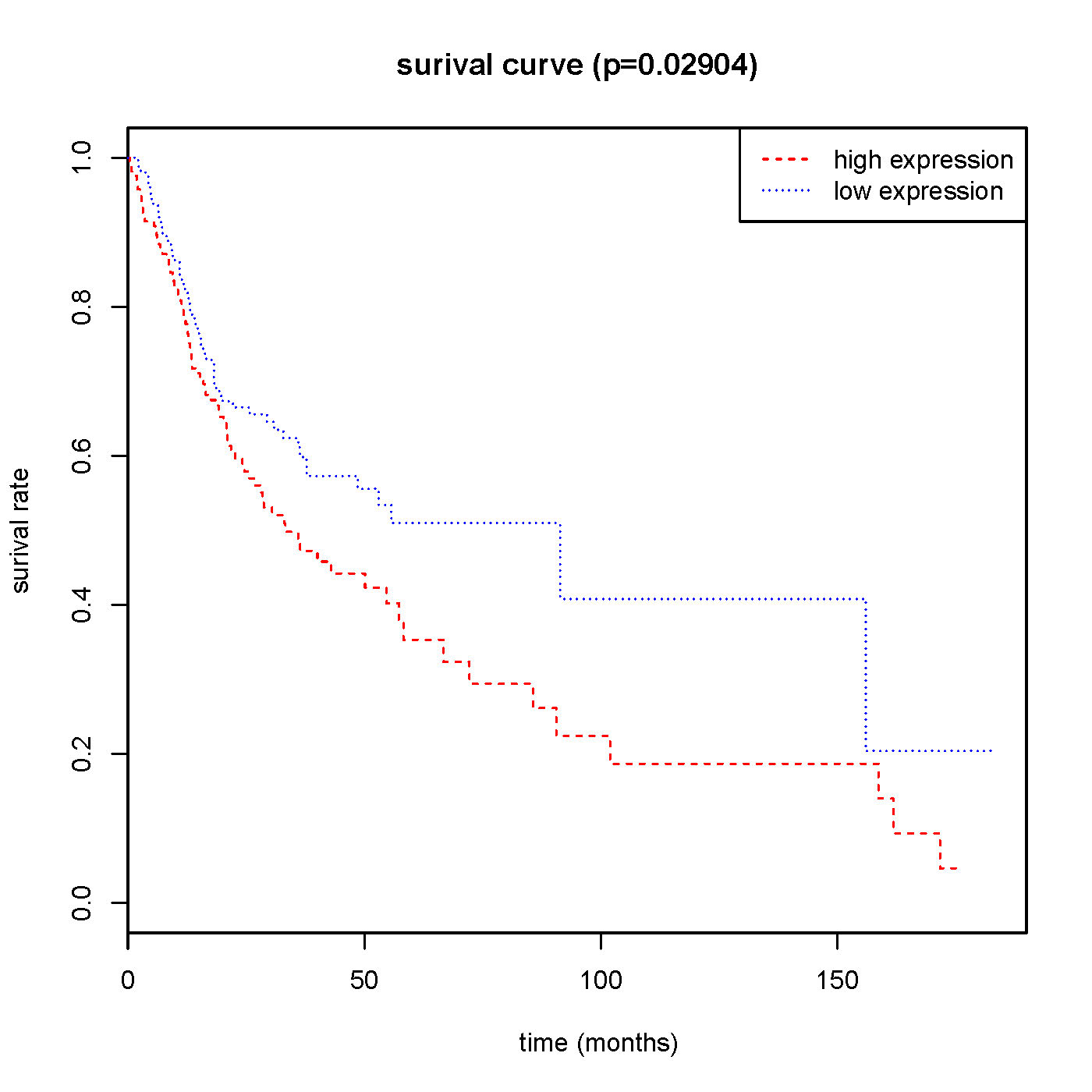

Supplement: Dataset S2 — Kaplan–Meier survival analysis with the log-rank was used to identify relationships between the above 2493 lncRNA signatures and OSCC patient survival. Then, we determined the levels of 151 lncRNA signatures that were significantly related to OS. [file peerj-06-5307-s006.zip › The result of Kaplan–Meier survival analyses and log-rank tests for OS in OSCC/RP11-542G1.1.jpg]

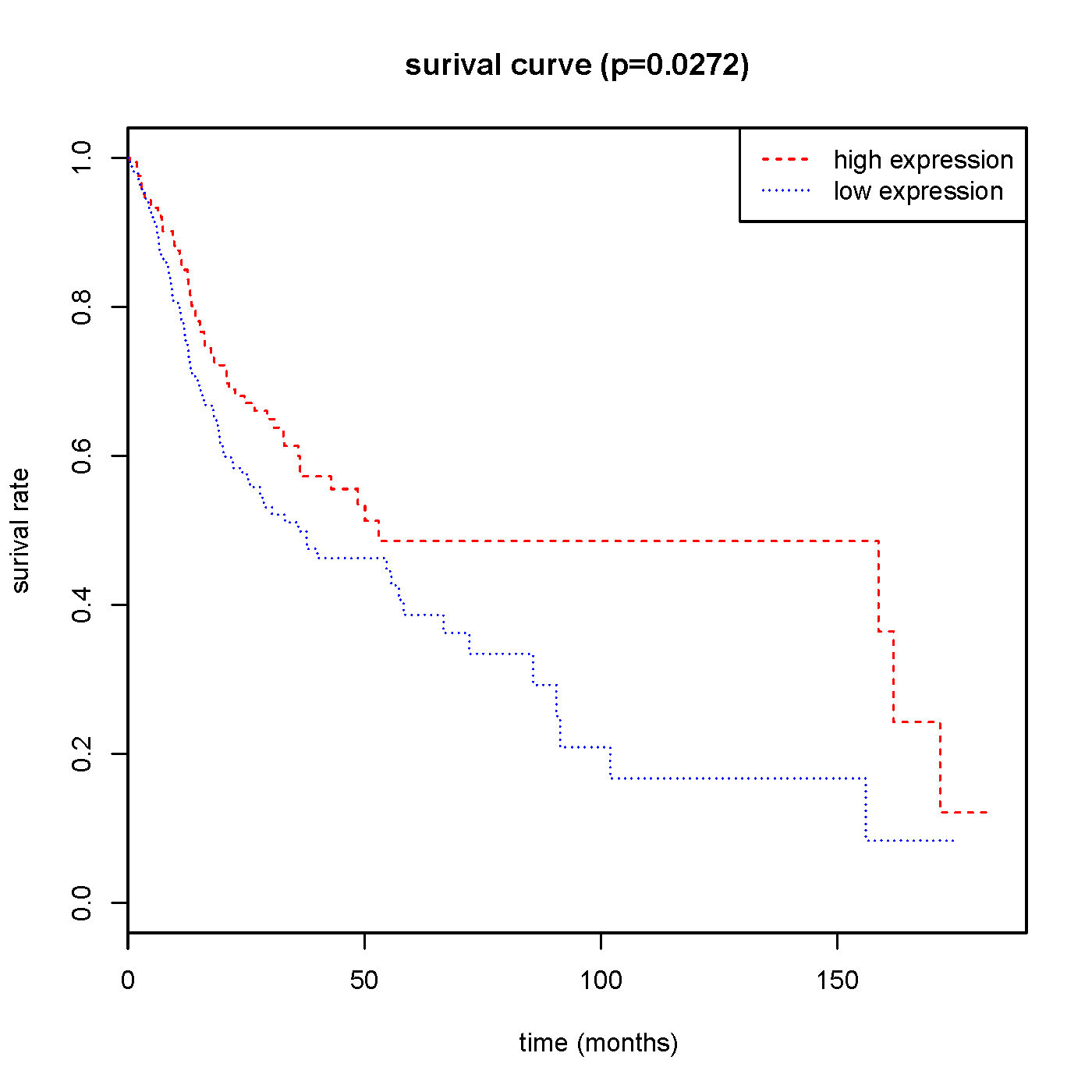

Supplement: Dataset S2 — Kaplan–Meier survival analysis with the log-rank was used to identify relationships between the above 2493 lncRNA signatures and OSCC patient survival. Then, we determined the levels of 151 lncRNA signatures that were significantly related to OS. [file peerj-06-5307-s006.zip › The result of Kaplan–Meier survival analyses and log-rank tests for OS in OSCC/RP11-55K13.1.jpg]

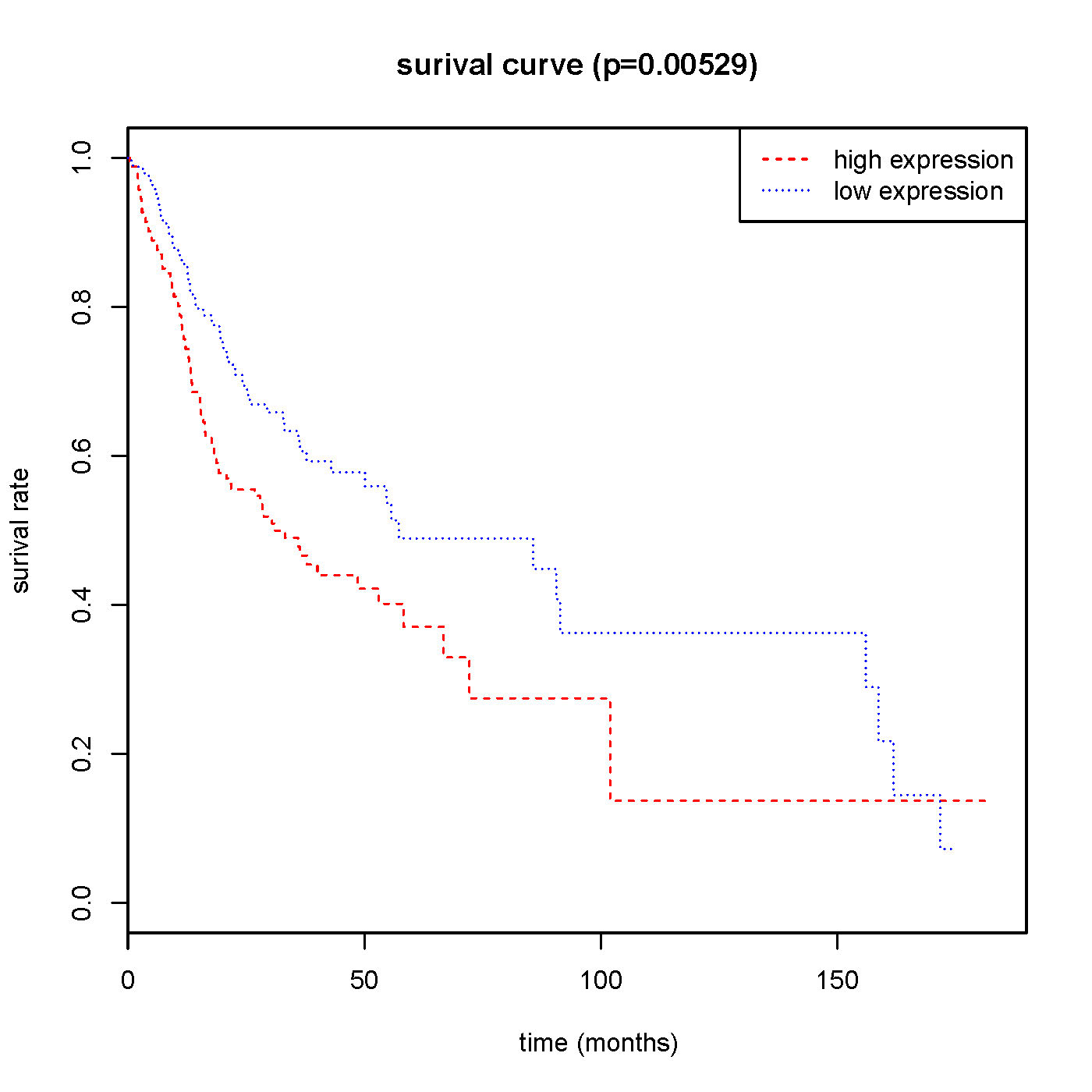

Supplement: Dataset S2 — Kaplan–Meier survival analysis with the log-rank was used to identify relationships between the above 2493 lncRNA signatures and OSCC patient survival. Then, we determined the levels of 151 lncRNA signatures that were significantly related to OS. [file peerj-06-5307-s006.zip › The result of Kaplan–Meier survival analyses and log-rank tests for OS in OSCC/RP11-59D5__B.2.jpg]

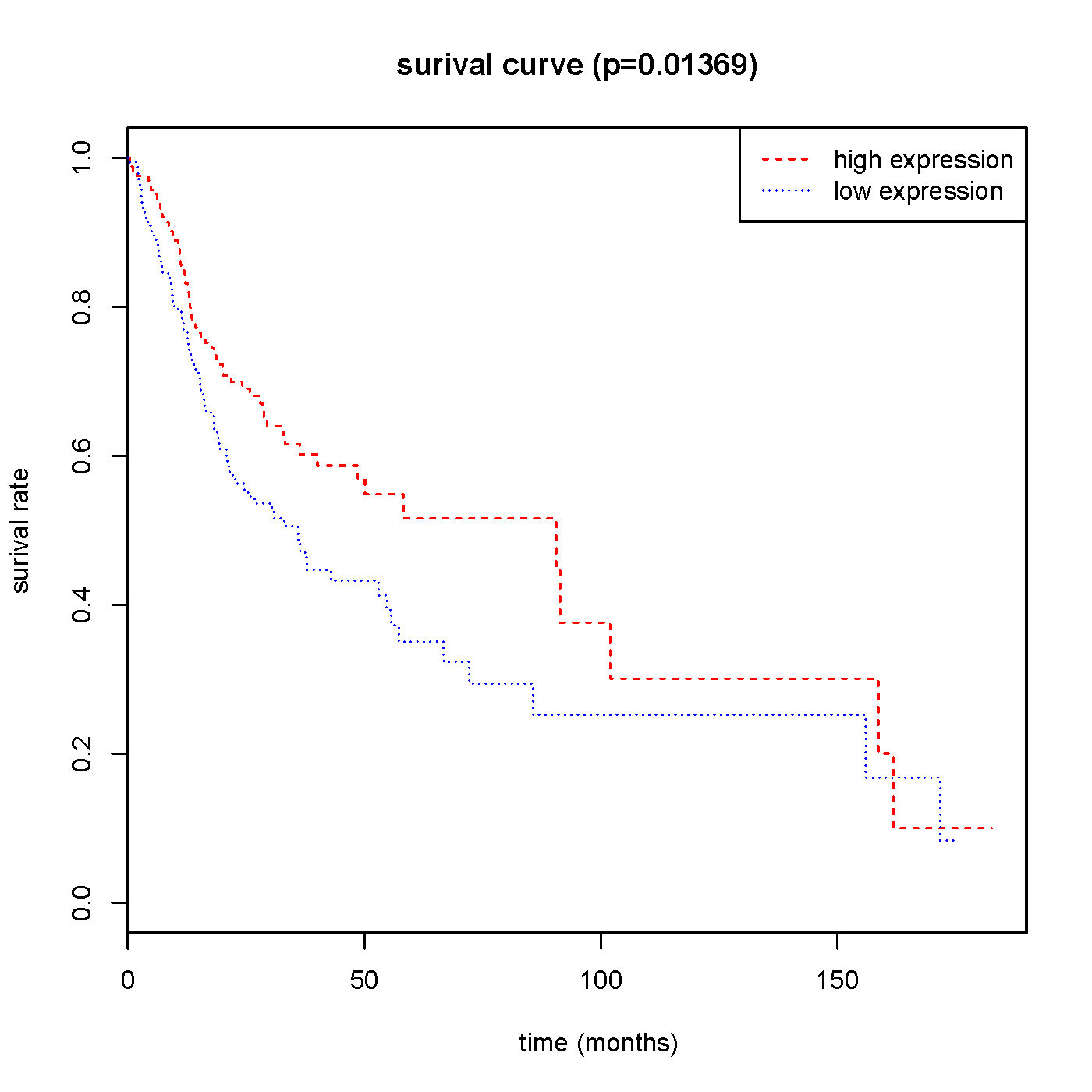

Supplement: Dataset S2 — Kaplan–Meier survival analysis with the log-rank was used to identify relationships between the above 2493 lncRNA signatures and OSCC patient survival. Then, we determined the levels of 151 lncRNA signatures that were significantly related to OS. [file peerj-06-5307-s006.zip › The result of Kaplan–Meier survival analyses and log-rank tests for OS in OSCC/RP11-64B16.4.jpg]

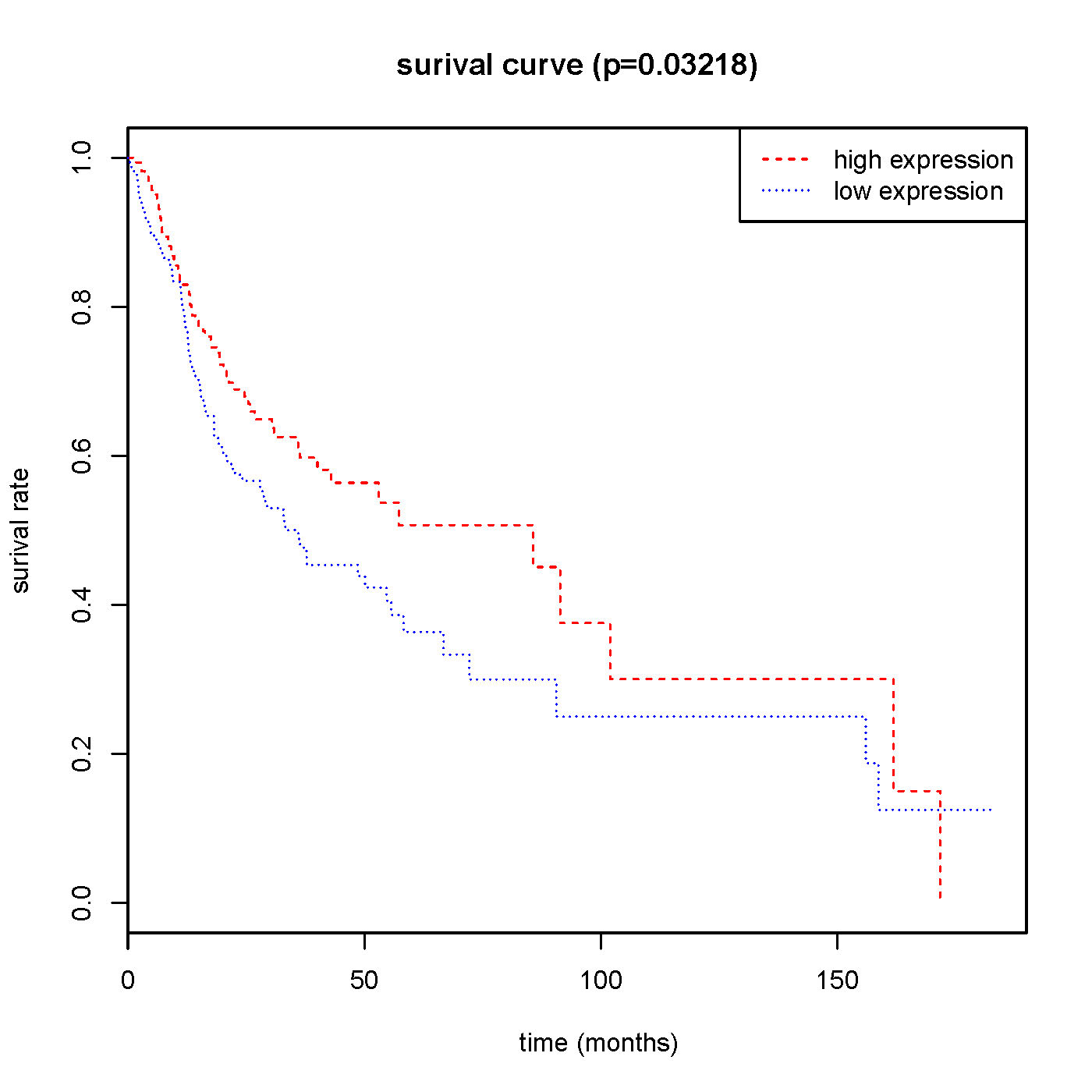

Supplement: Dataset S2 — Kaplan–Meier survival analysis with the log-rank was used to identify relationships between the above 2493 lncRNA signatures and OSCC patient survival. Then, we determined the levels of 151 lncRNA signatures that were significantly related to OS. [file peerj-06-5307-s006.zip › The result of Kaplan–Meier survival analyses and log-rank tests for OS in OSCC/RP11-650P15.1.jpg]

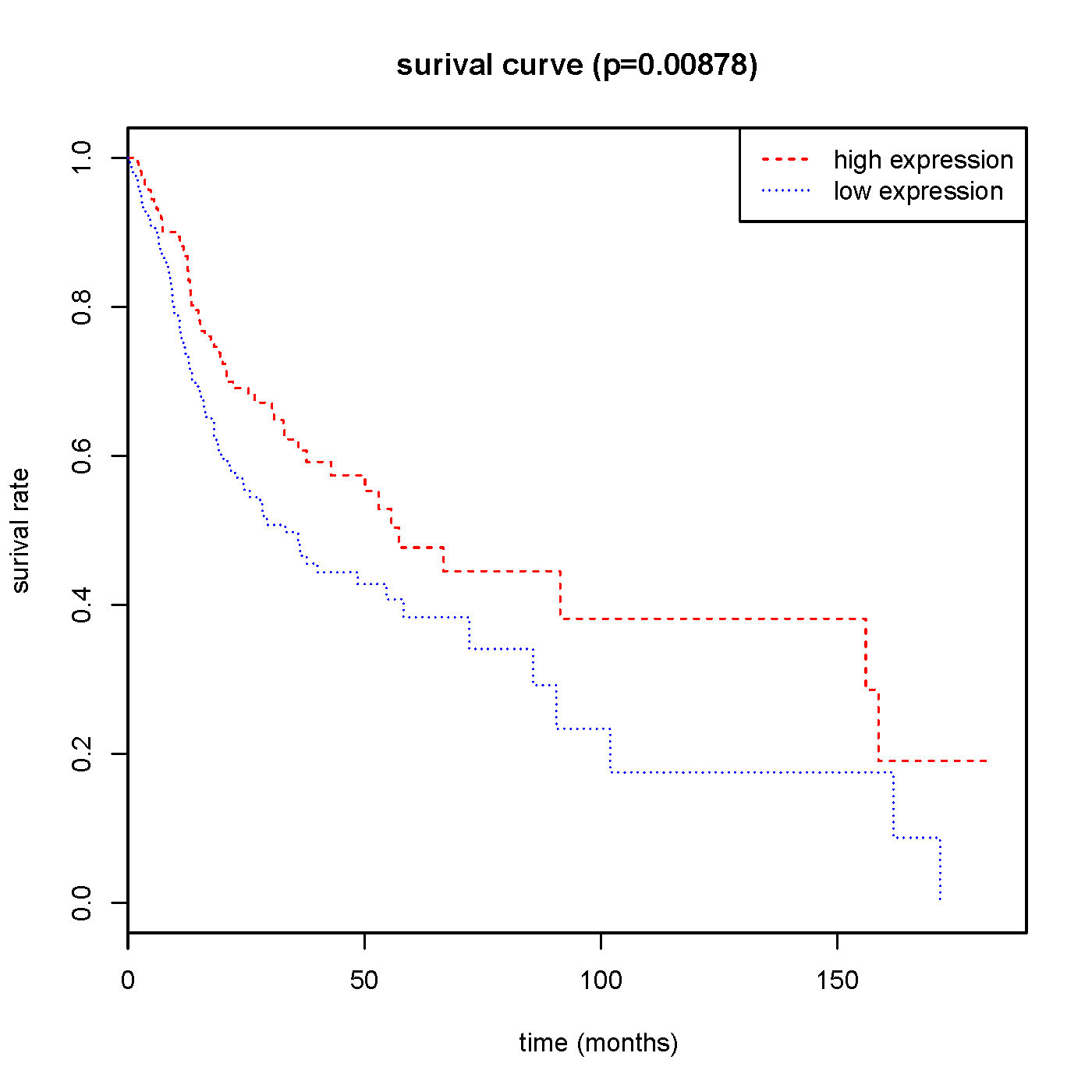

Supplement: Dataset S2 — Kaplan–Meier survival analysis with the log-rank was used to identify relationships between the above 2493 lncRNA signatures and OSCC patient survival. Then, we determined the levels of 151 lncRNA signatures that were significantly related to OS. [file peerj-06-5307-s006.zip › The result of Kaplan–Meier survival analyses and log-rank tests for OS in OSCC/RP11-674P19.2.jpg]

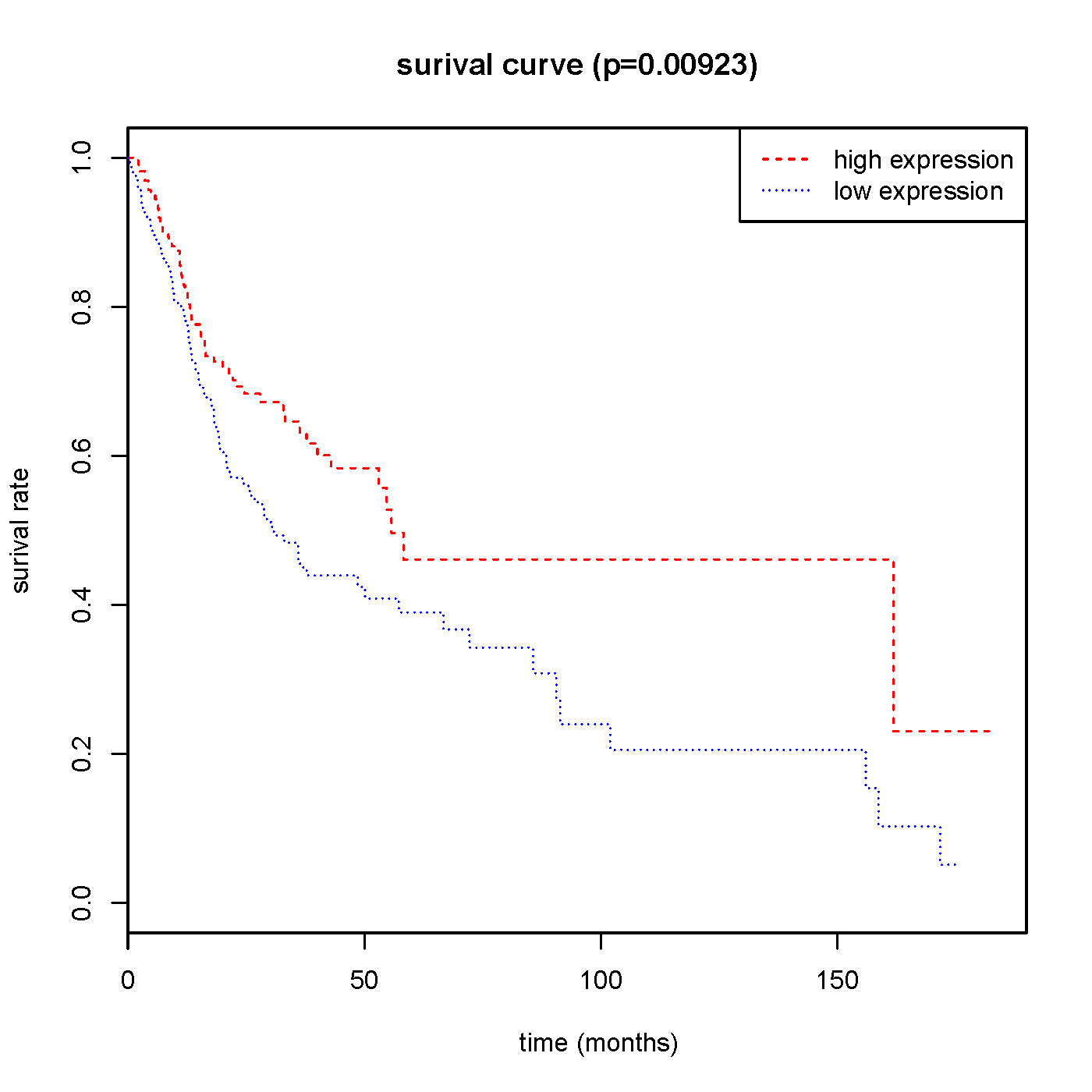

Supplement: Dataset S2 — Kaplan–Meier survival analysis with the log-rank was used to identify relationships between the above 2493 lncRNA signatures and OSCC patient survival. Then, we determined the levels of 151 lncRNA signatures that were significantly related to OS. [file peerj-06-5307-s006.zip › The result of Kaplan–Meier survival analyses and log-rank tests for OS in OSCC/RP11-677M14.3.jpg]

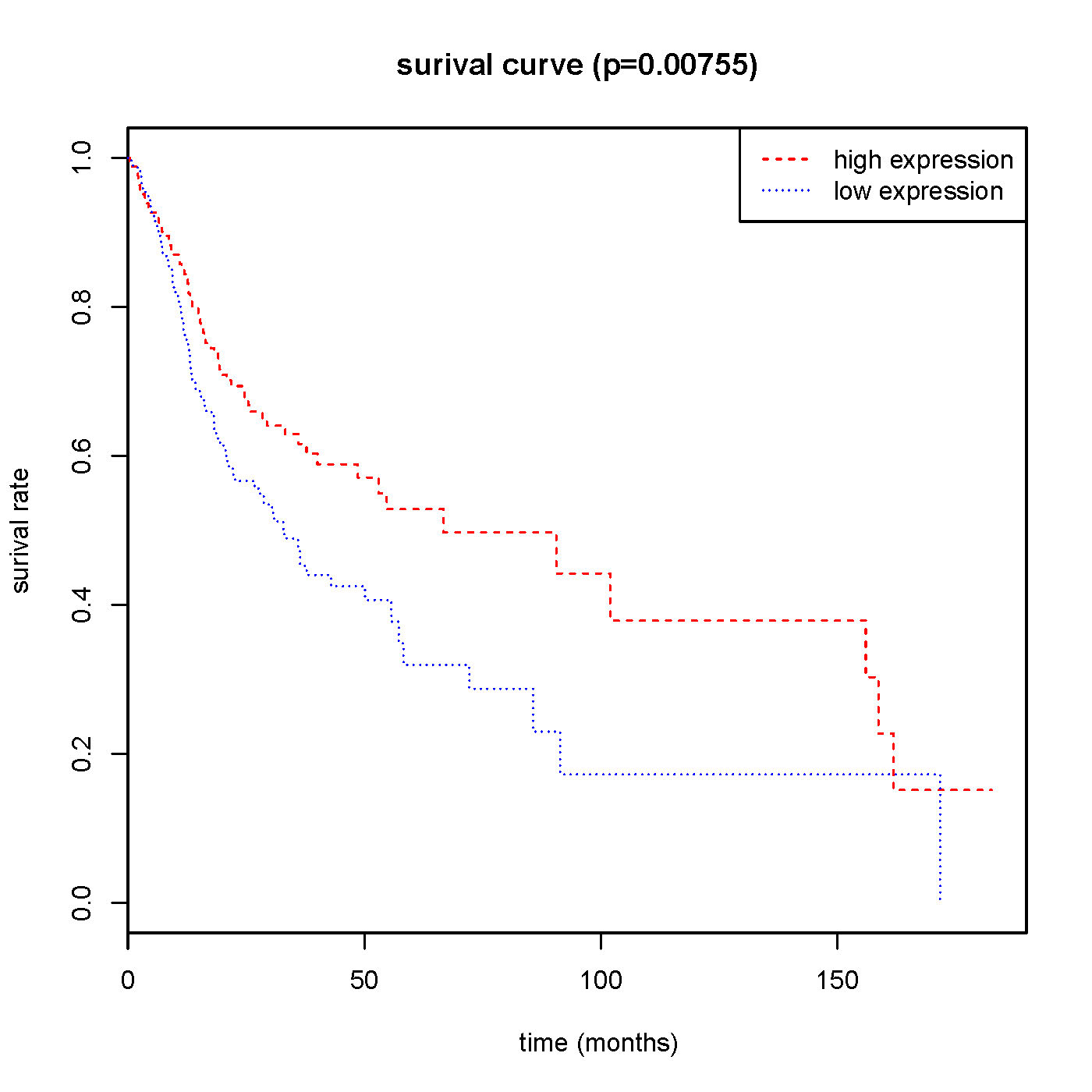

Supplement: Dataset S2 — Kaplan–Meier survival analysis with the log-rank was used to identify relationships between the above 2493 lncRNA signatures and OSCC patient survival. Then, we determined the levels of 151 lncRNA signatures that were significantly related to OS. [file peerj-06-5307-s006.zip › The result of Kaplan–Meier survival analyses and log-rank tests for OS in OSCC/RP11-69I8.3.jpg]

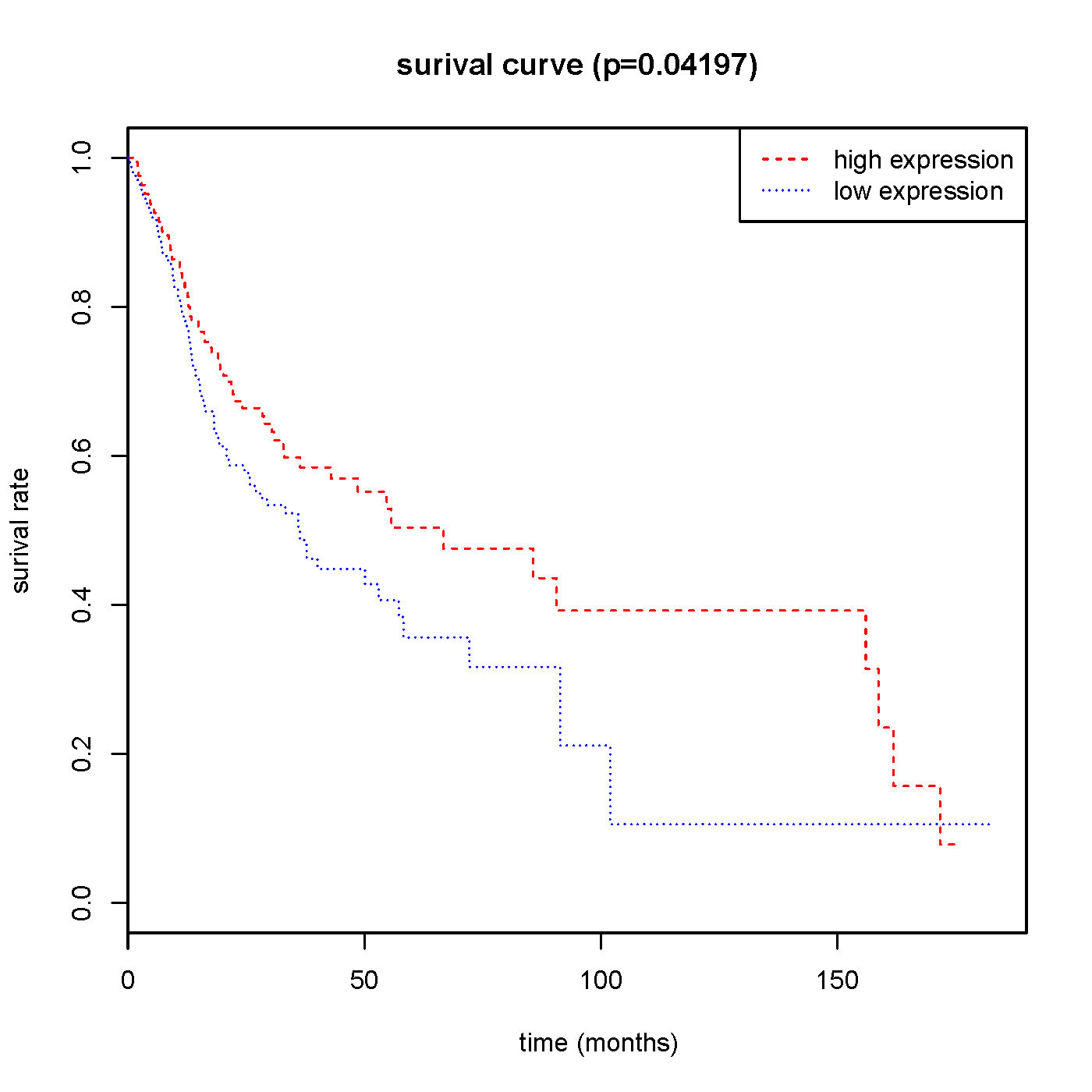

Supplement: Dataset S2 — Kaplan–Meier survival analysis with the log-rank was used to identify relationships between the above 2493 lncRNA signatures and OSCC patient survival. Then, we determined the levels of 151 lncRNA signatures that were significantly related to OS. [file peerj-06-5307-s006.zip › The result of Kaplan–Meier survival analyses and log-rank tests for OS in OSCC/RP11-700H6.1.jpg]

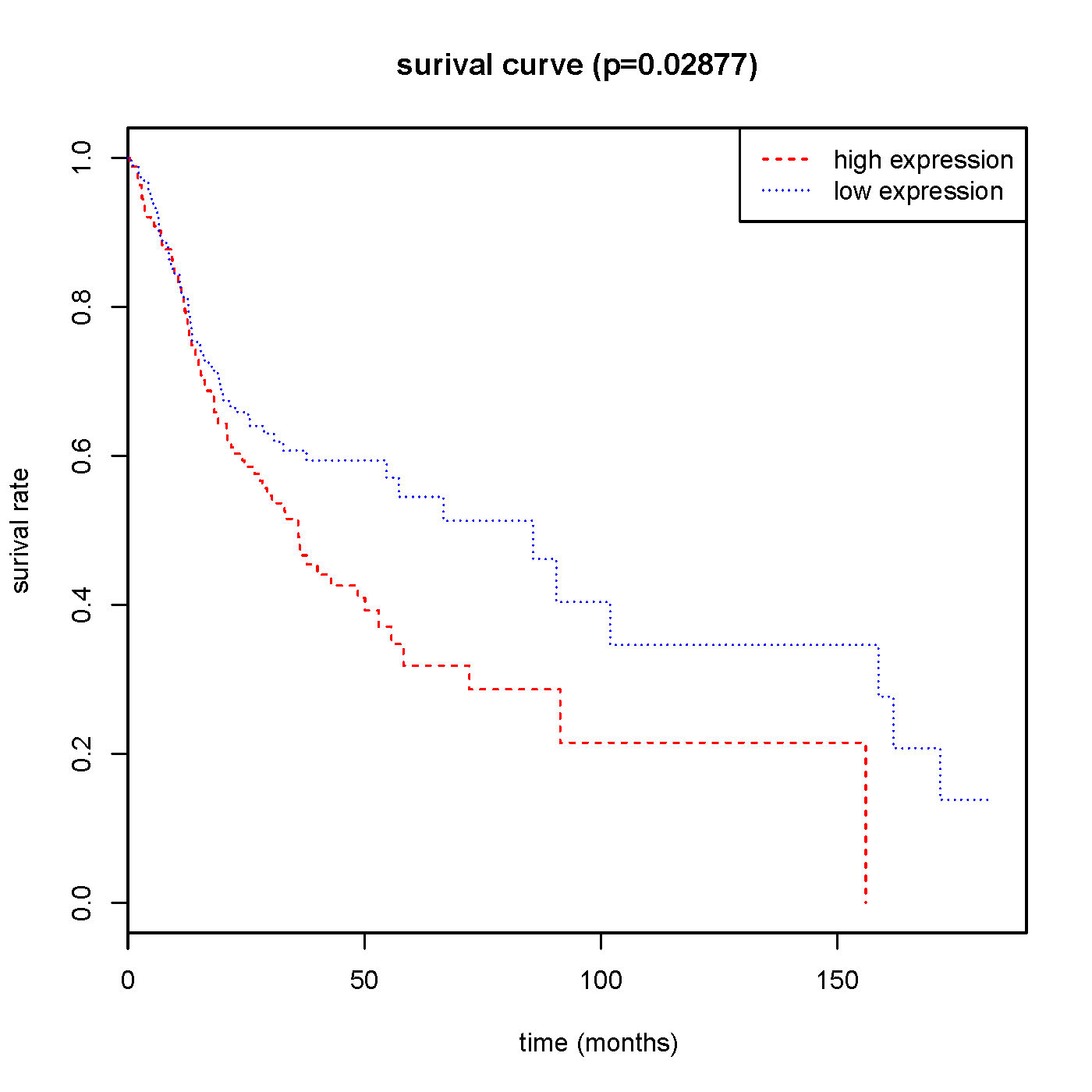

Supplement: Dataset S2 — Kaplan–Meier survival analysis with the log-rank was used to identify relationships between the above 2493 lncRNA signatures and OSCC patient survival. Then, we determined the levels of 151 lncRNA signatures that were significantly related to OS. [file peerj-06-5307-s006.zip › The result of Kaplan–Meier survival analyses and log-rank tests for OS in OSCC/RP11-715J22.6.jpg]

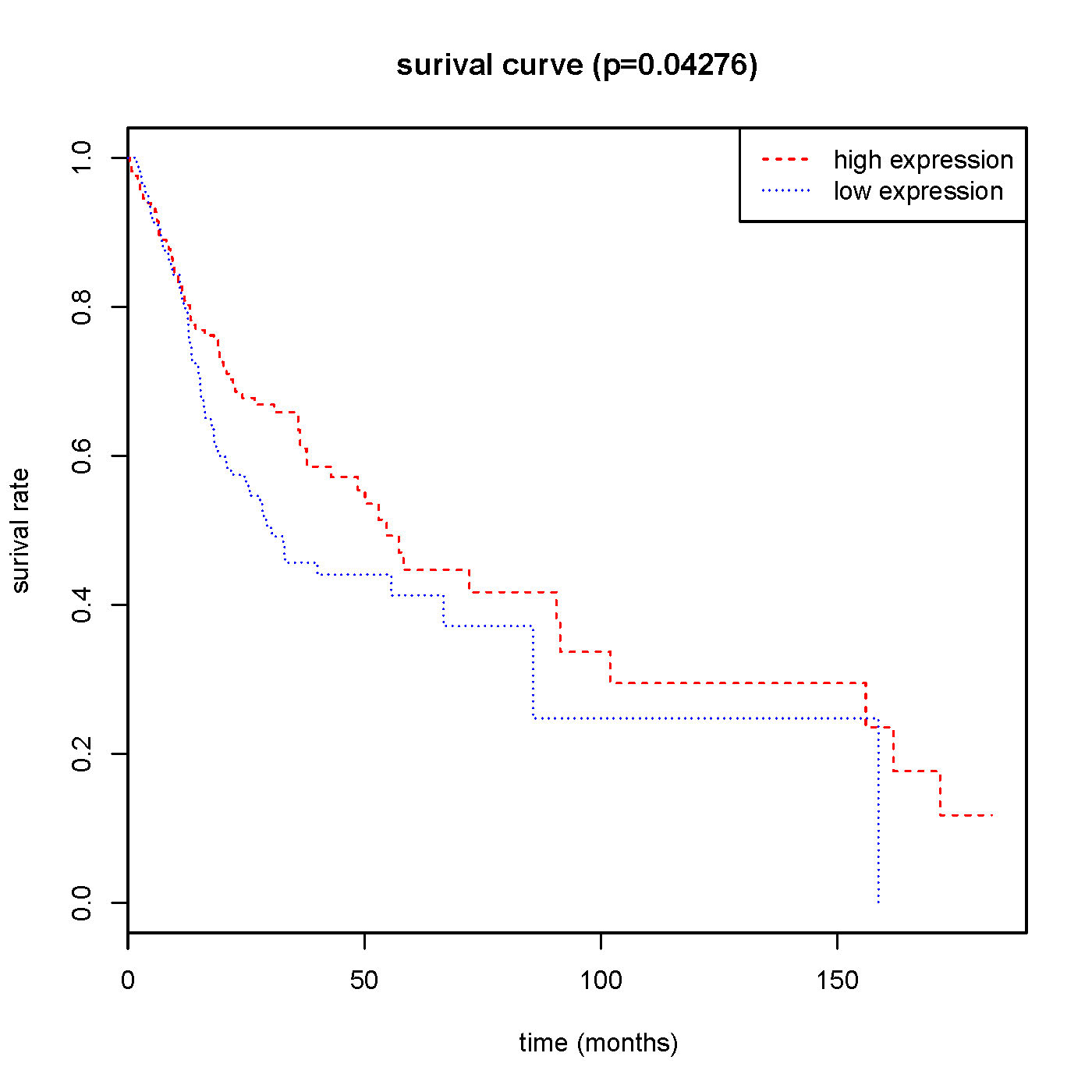

Supplement: Dataset S2 — Kaplan–Meier survival analysis with the log-rank was used to identify relationships between the above 2493 lncRNA signatures and OSCC patient survival. Then, we determined the levels of 151 lncRNA signatures that were significantly related to OS. [file peerj-06-5307-s006.zip › The result of Kaplan–Meier survival analyses and log-rank tests for OS in OSCC/RP11-796E2.4.jpg]

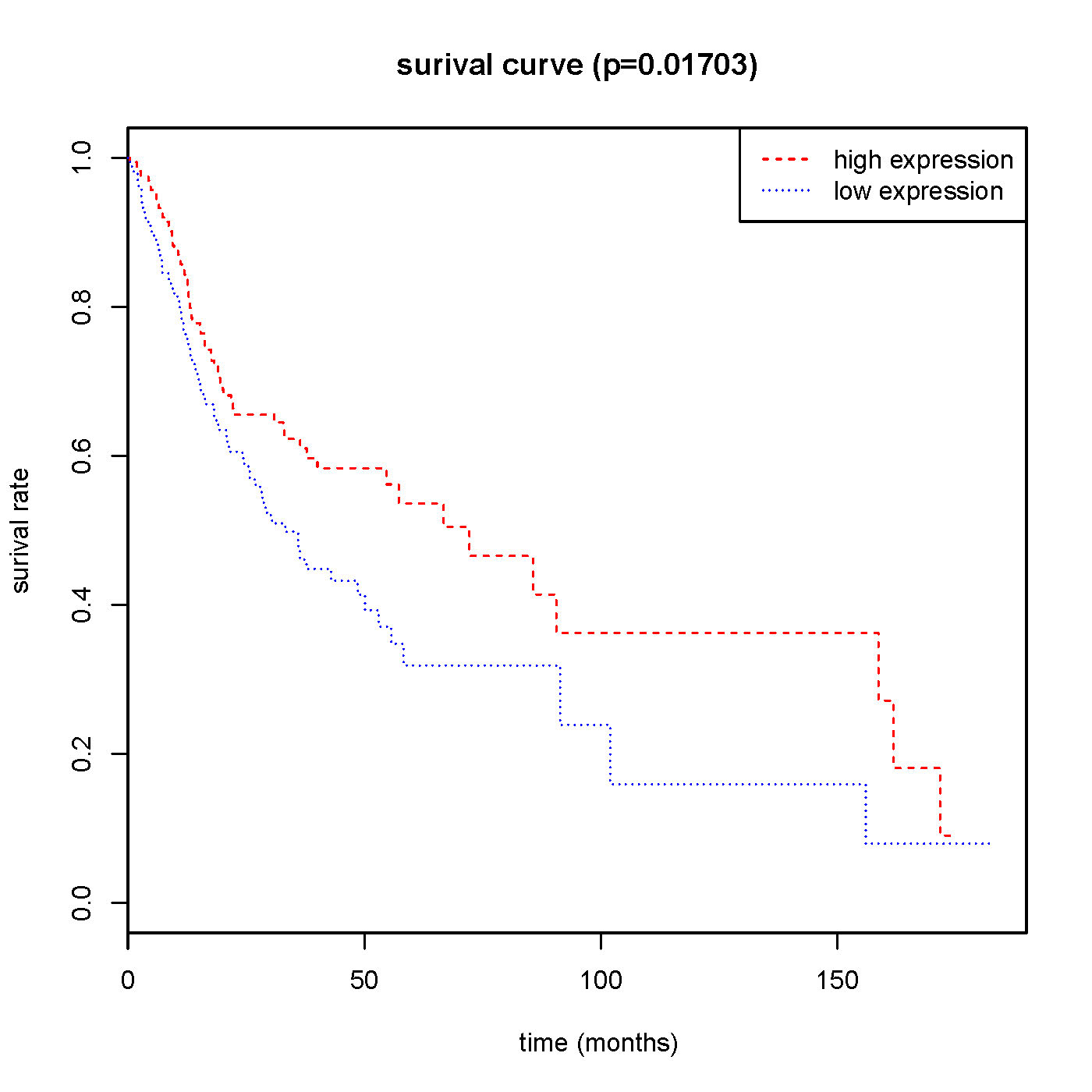

Supplement: Dataset S2 — Kaplan–Meier survival analysis with the log-rank was used to identify relationships between the above 2493 lncRNA signatures and OSCC patient survival. Then, we determined the levels of 151 lncRNA signatures that were significantly related to OS. [file peerj-06-5307-s006.zip › The result of Kaplan–Meier survival analyses and log-rank tests for OS in OSCC/RP11-7I15.4.jpg]

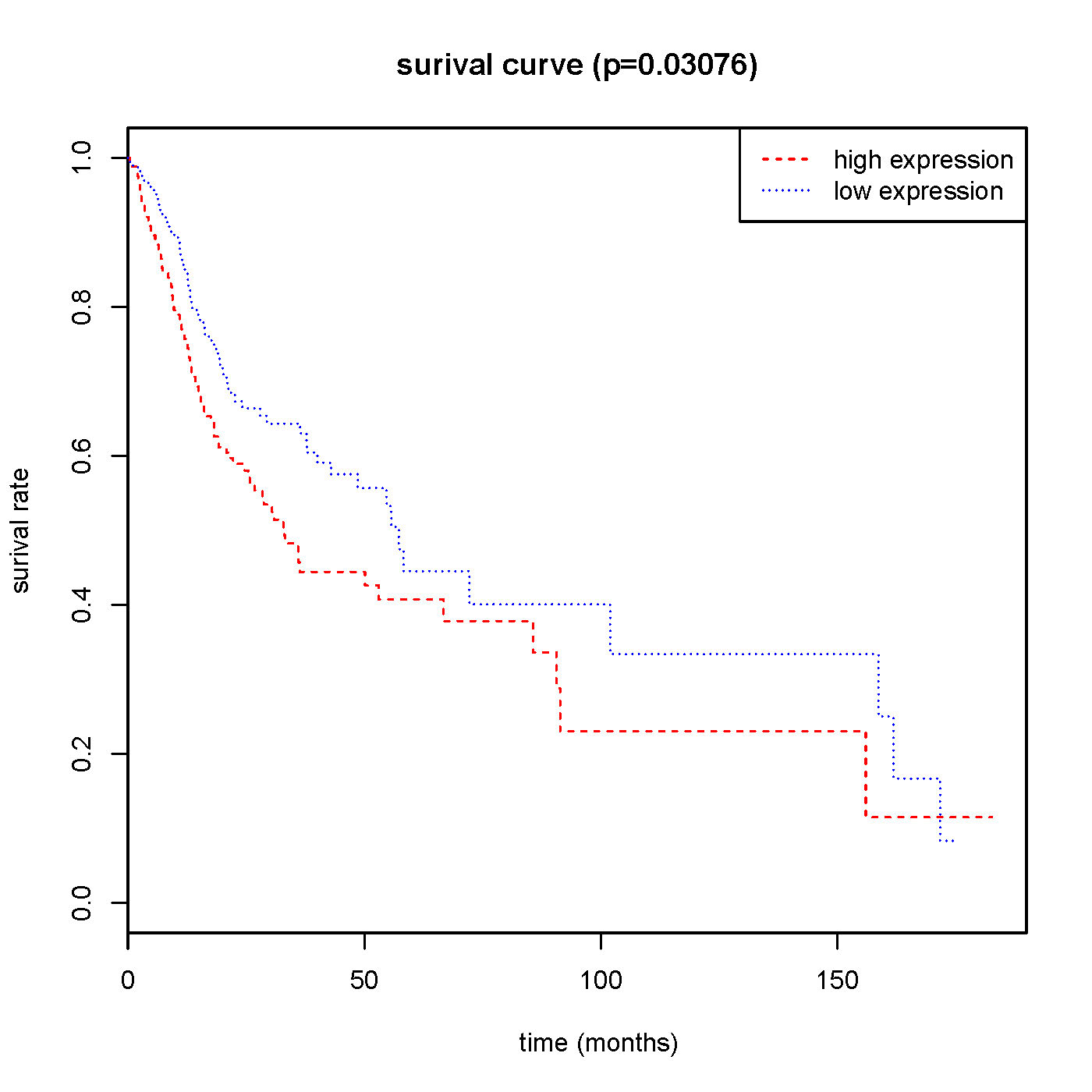

Supplement: Dataset S2 — Kaplan–Meier survival analysis with the log-rank was used to identify relationships between the above 2493 lncRNA signatures and OSCC patient survival. Then, we determined the levels of 151 lncRNA signatures that were significantly related to OS. [file peerj-06-5307-s006.zip › The result of Kaplan–Meier survival analyses and log-rank tests for OS in OSCC/RP11-817I4.2.jpg]

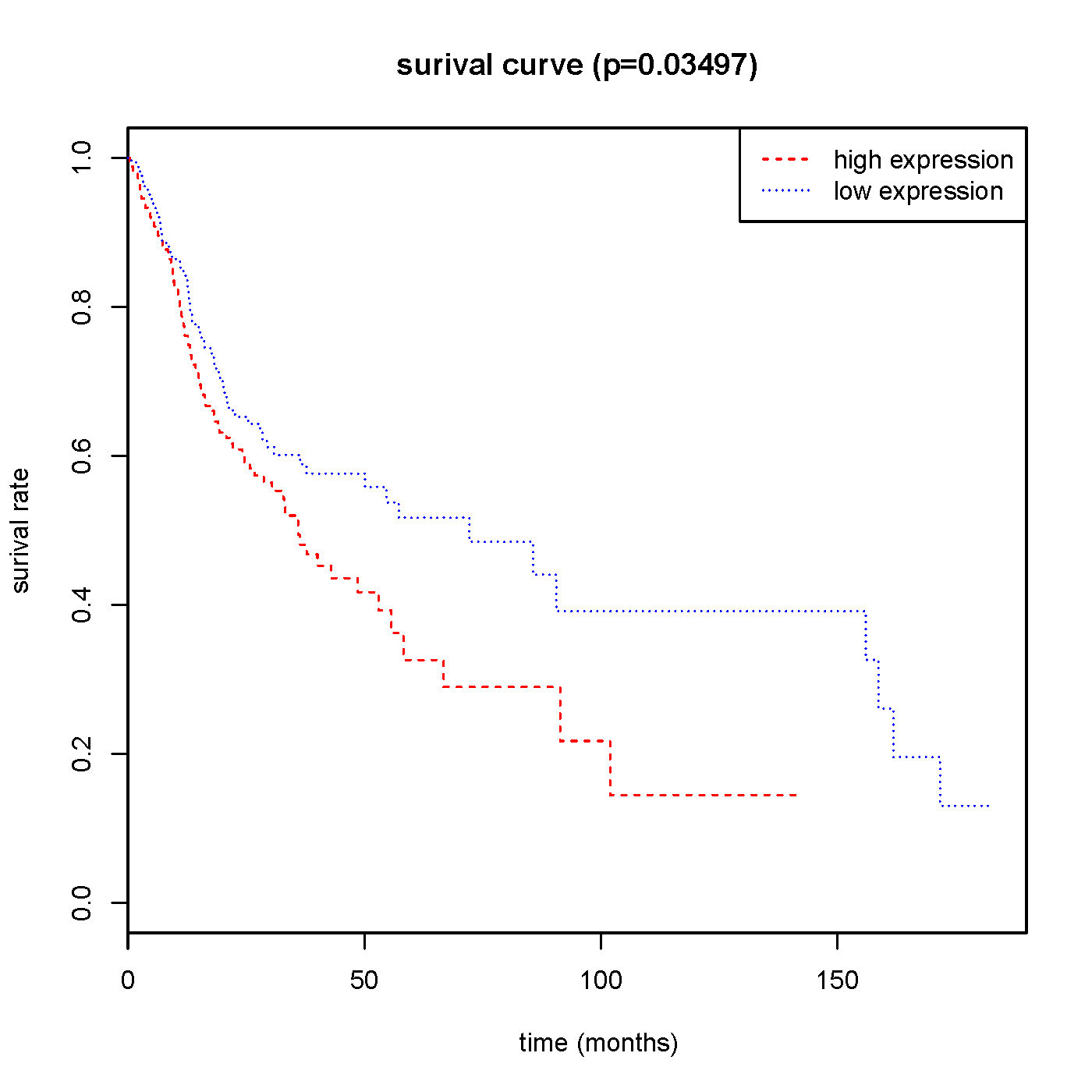

Supplement: Dataset S2 — Kaplan–Meier survival analysis with the log-rank was used to identify relationships between the above 2493 lncRNA signatures and OSCC patient survival. Then, we determined the levels of 151 lncRNA signatures that were significantly related to OS. [file peerj-06-5307-s006.zip › The result of Kaplan–Meier survival analyses and log-rank tests for OS in OSCC/RP11-85B7.2.jpg]

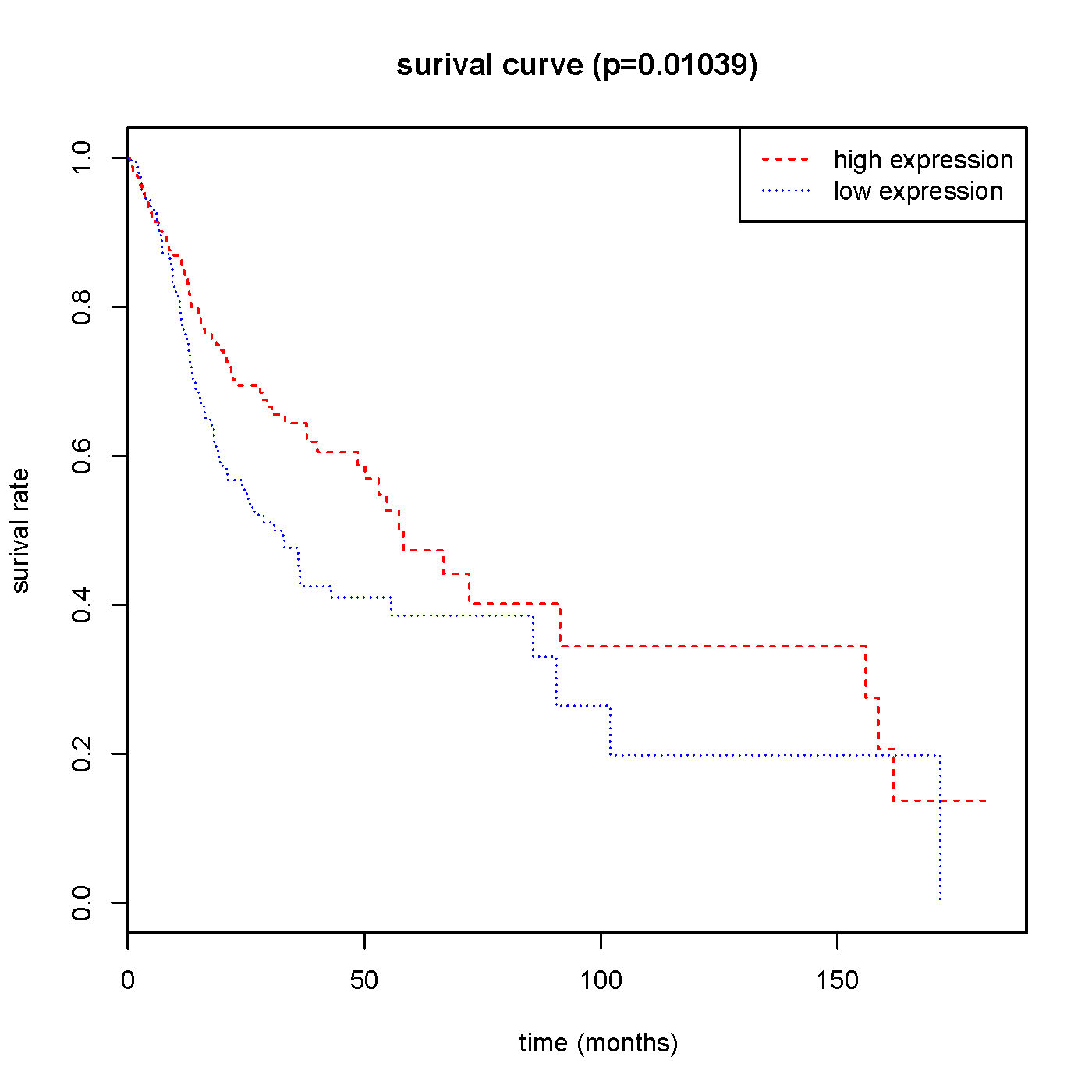

Supplement: Dataset S2 — Kaplan–Meier survival analysis with the log-rank was used to identify relationships between the above 2493 lncRNA signatures and OSCC patient survival. Then, we determined the levels of 151 lncRNA signatures that were significantly related to OS. [file peerj-06-5307-s006.zip › The result of Kaplan–Meier survival analyses and log-rank tests for OS in OSCC/RP11-863K10.7.jpg]

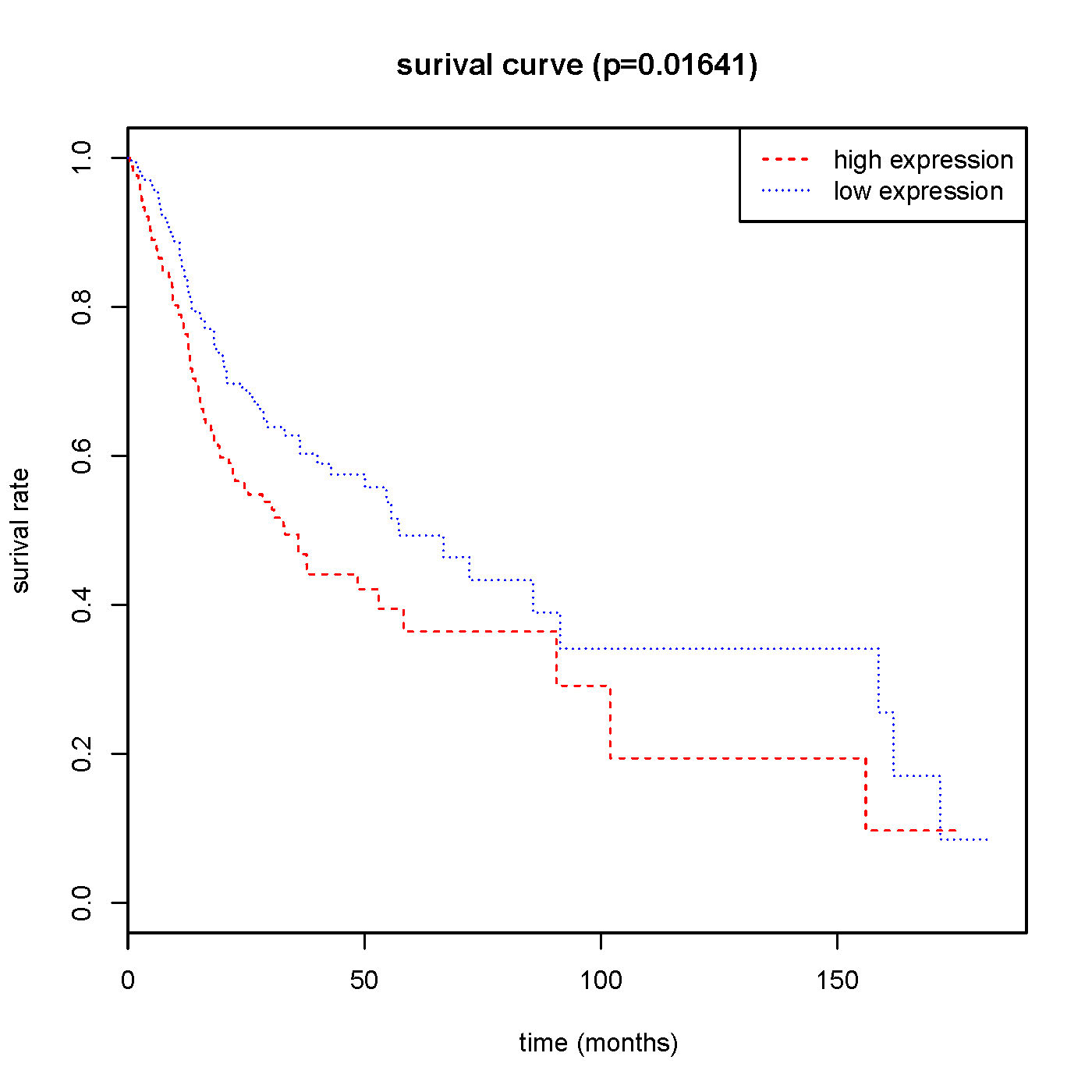

Supplement: Dataset S2 — Kaplan–Meier survival analysis with the log-rank was used to identify relationships between the above 2493 lncRNA signatures and OSCC patient survival. Then, we determined the levels of 151 lncRNA signatures that were significantly related to OS. [file peerj-06-5307-s006.zip › The result of Kaplan–Meier survival analyses and log-rank tests for OS in OSCC/RP11-865I6.2.jpg]

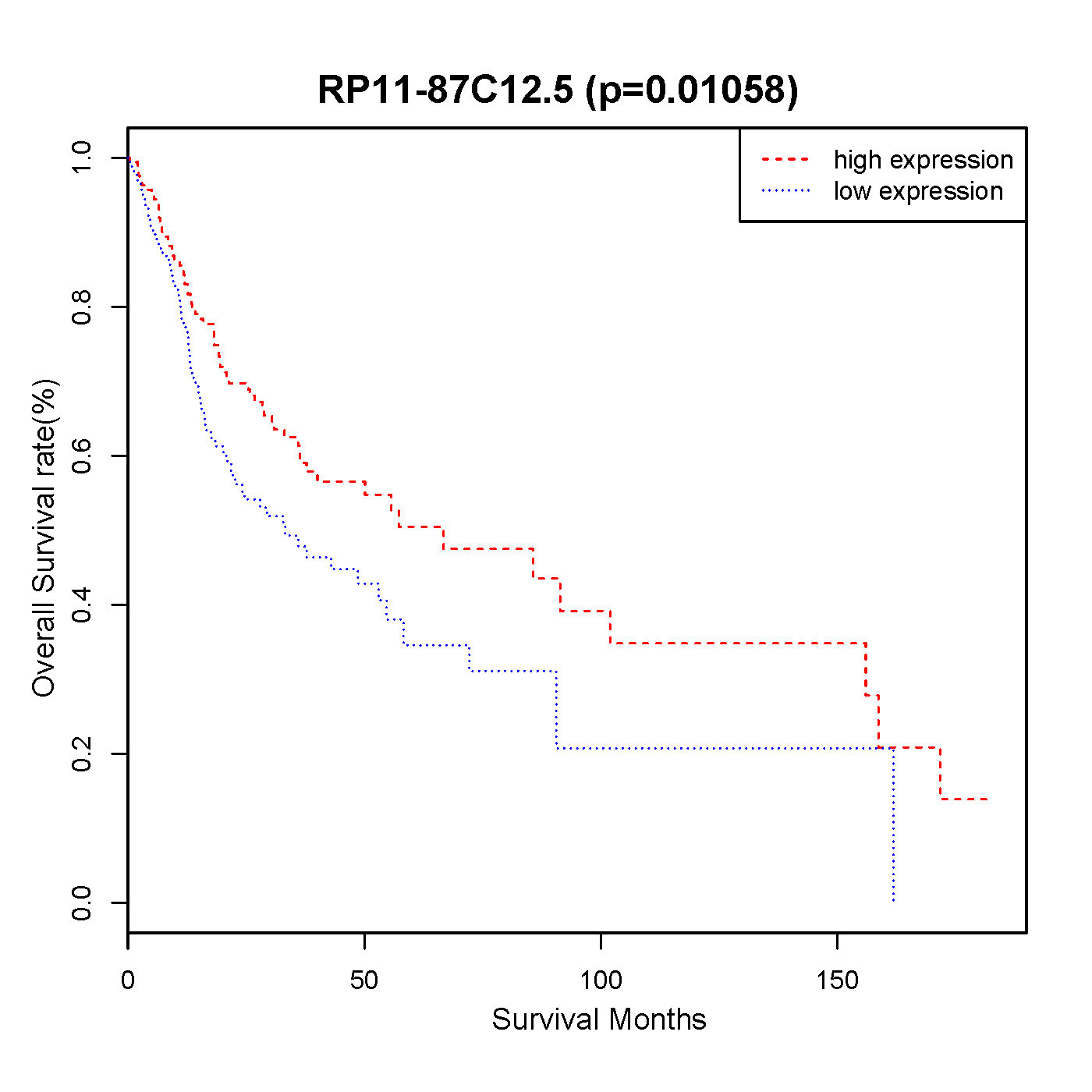

Supplement: Dataset S2 — Kaplan–Meier survival analysis with the log-rank was used to identify relationships between the above 2493 lncRNA signatures and OSCC patient survival. Then, we determined the levels of 151 lncRNA signatures that were significantly related to OS. [file peerj-06-5307-s006.zip › The result of Kaplan–Meier survival analyses and log-rank tests for OS in OSCC/RP11-87C12.5.jpg]

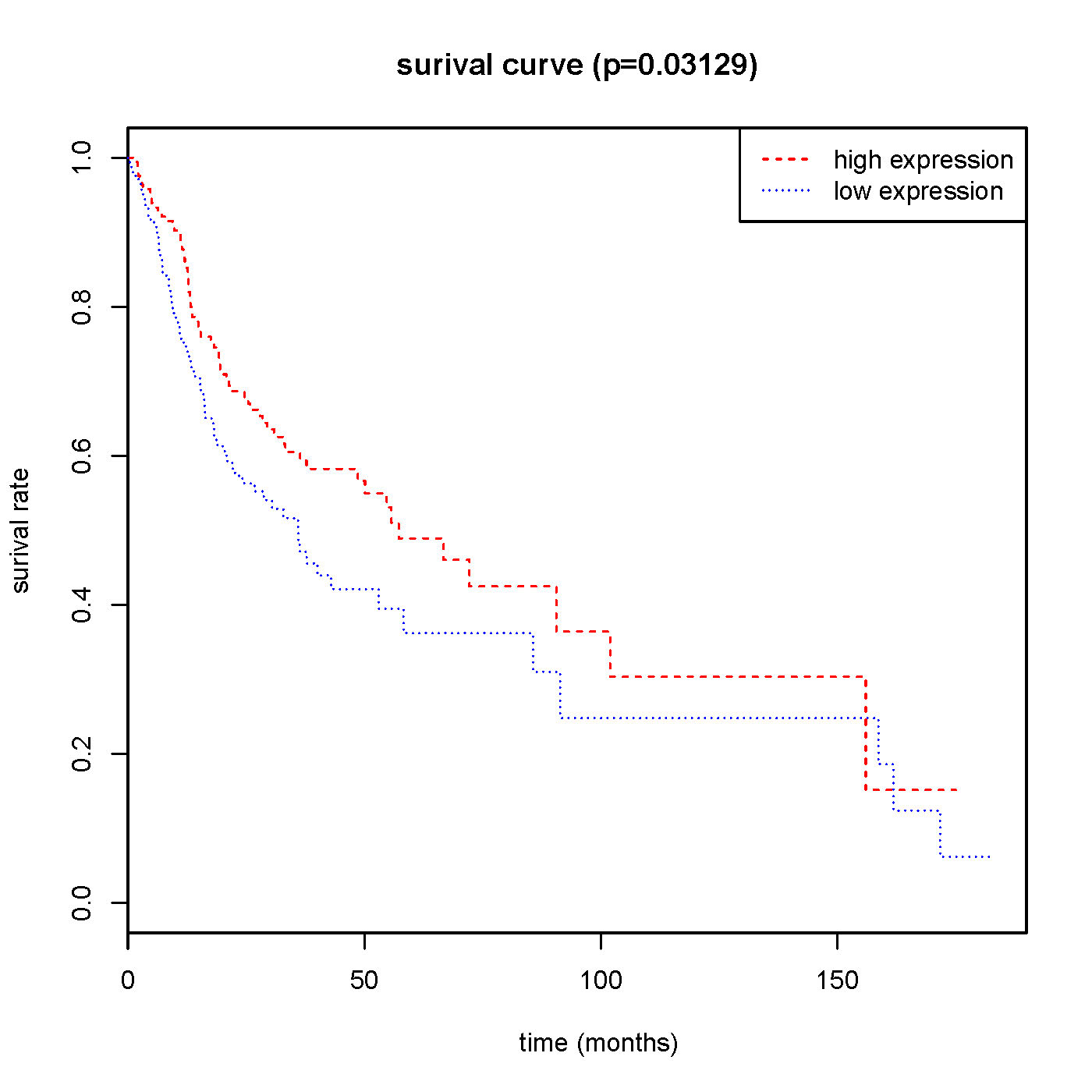

Supplement: Dataset S2 — Kaplan–Meier survival analysis with the log-rank was used to identify relationships between the above 2493 lncRNA signatures and OSCC patient survival. Then, we determined the levels of 151 lncRNA signatures that were significantly related to OS. [file peerj-06-5307-s006.zip › The result of Kaplan–Meier survival analyses and log-rank tests for OS in OSCC/RP11-897M7.1.jpg]

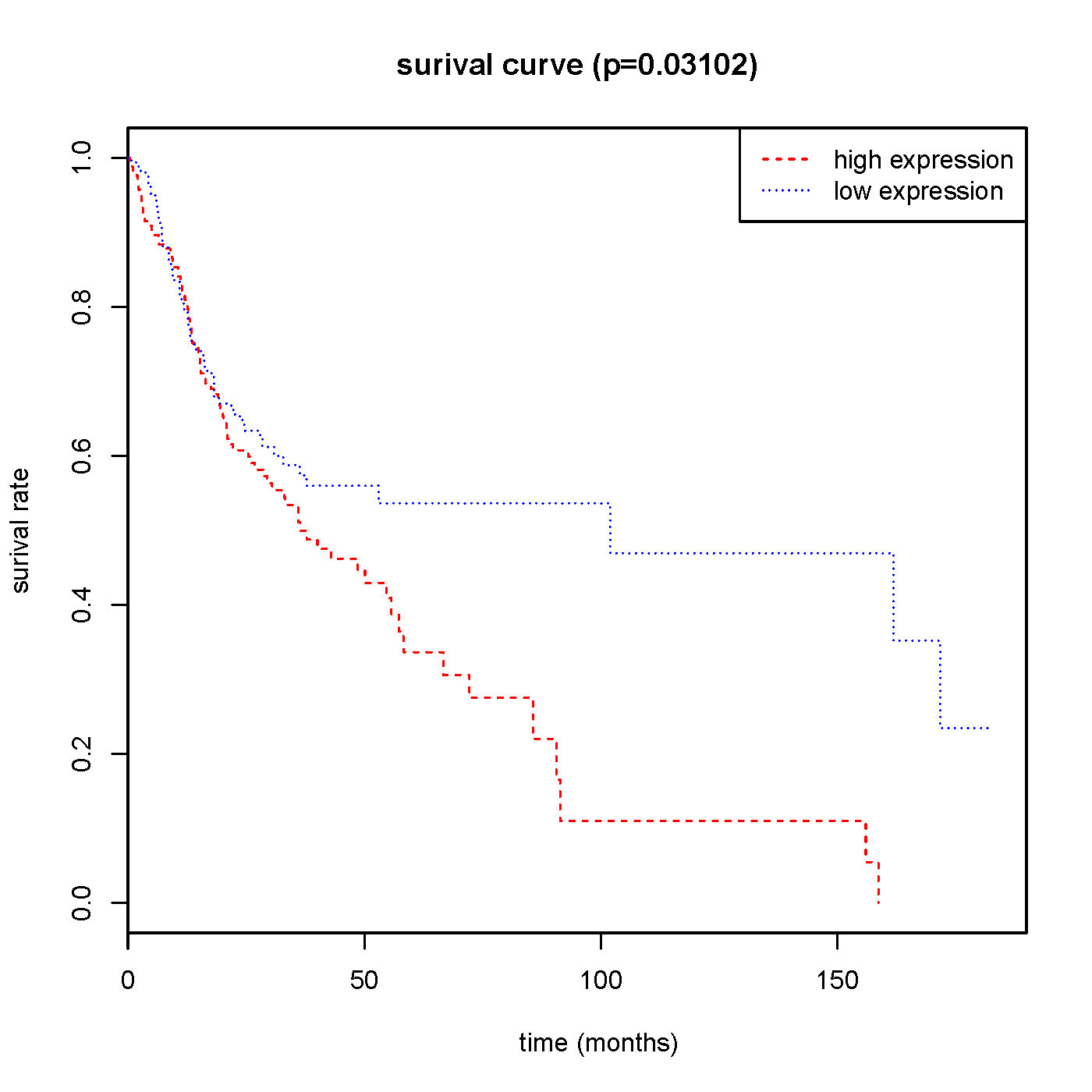

Supplement: Dataset S2 — Kaplan–Meier survival analysis with the log-rank was used to identify relationships between the above 2493 lncRNA signatures and OSCC patient survival. Then, we determined the levels of 151 lncRNA signatures that were significantly related to OS. [file peerj-06-5307-s006.zip › The result of Kaplan–Meier survival analyses and log-rank tests for OS in OSCC/RP11-8L2.1.jpg]

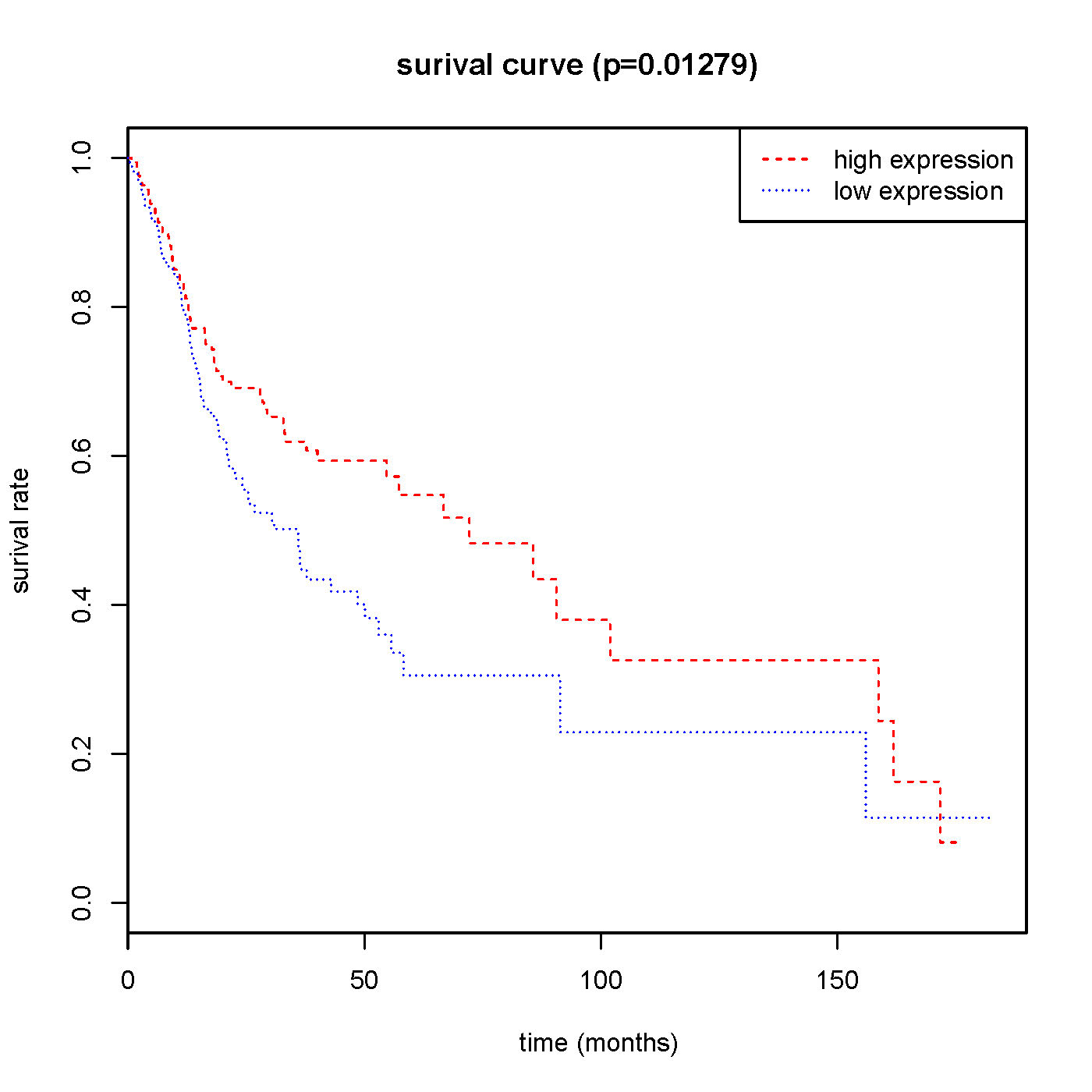

Supplement: Dataset S2 — Kaplan–Meier survival analysis with the log-rank was used to identify relationships between the above 2493 lncRNA signatures and OSCC patient survival. Then, we determined the levels of 151 lncRNA signatures that were significantly related to OS. [file peerj-06-5307-s006.zip › The result of Kaplan–Meier survival analyses and log-rank tests for OS in OSCC/RP11-93B14.9.jpg]

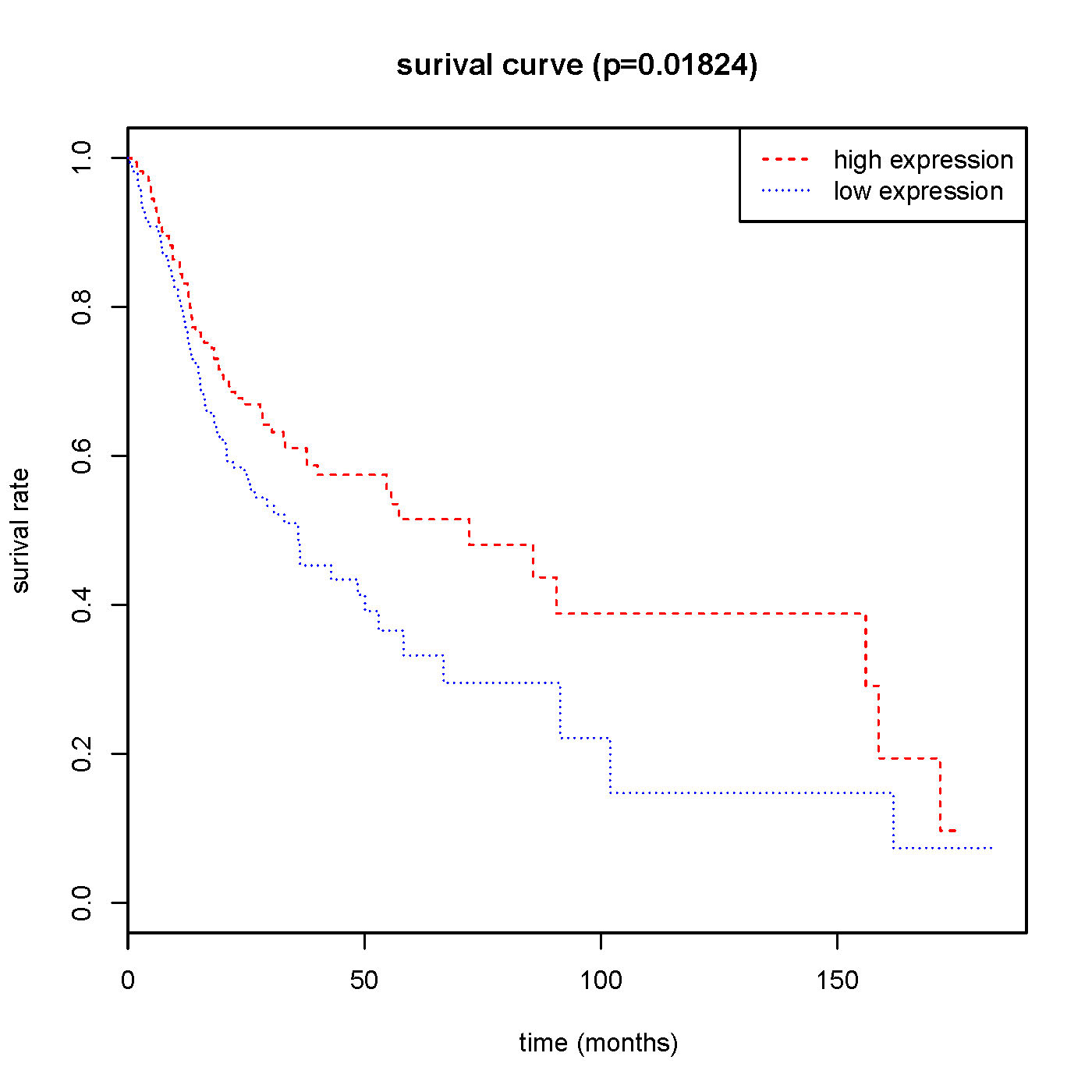

Supplement: Dataset S2 — Kaplan–Meier survival analysis with the log-rank was used to identify relationships between the above 2493 lncRNA signatures and OSCC patient survival. Then, we determined the levels of 151 lncRNA signatures that were significantly related to OS. [file peerj-06-5307-s006.zip › The result of Kaplan–Meier survival analyses and log-rank tests for OS in OSCC/RP11-96L7.2.jpg]

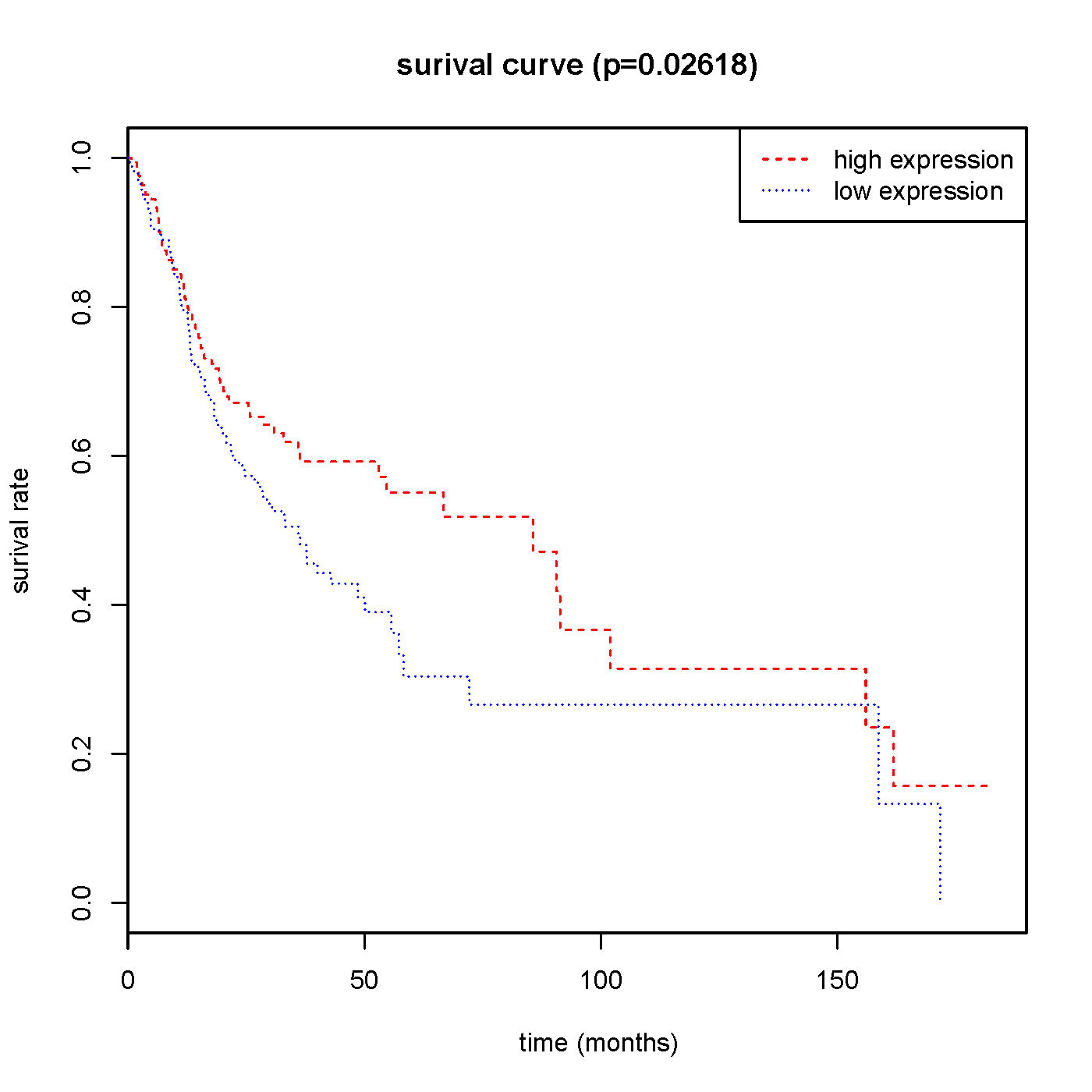

Supplement: Dataset S2 — Kaplan–Meier survival analysis with the log-rank was used to identify relationships between the above 2493 lncRNA signatures and OSCC patient survival. Then, we determined the levels of 151 lncRNA signatures that were significantly related to OS. [file peerj-06-5307-s006.zip › The result of Kaplan–Meier survival analyses and log-rank tests for OS in OSCC/RP11-993B23.3.jpg]

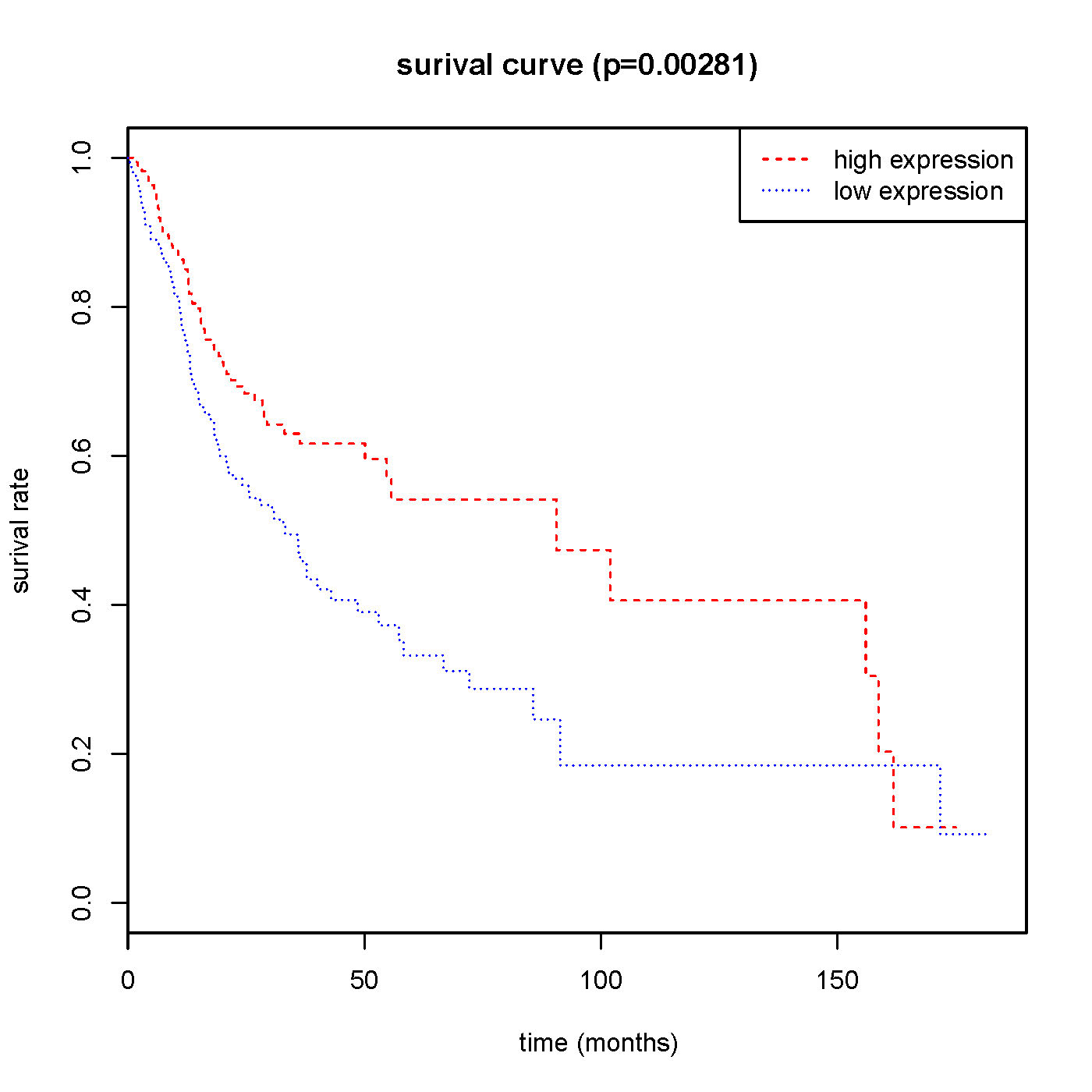

Supplement: Dataset S2 — Kaplan–Meier survival analysis with the log-rank was used to identify relationships between the above 2493 lncRNA signatures and OSCC patient survival. Then, we determined the levels of 151 lncRNA signatures that were significantly related to OS. [file peerj-06-5307-s006.zip › The result of Kaplan–Meier survival analyses and log-rank tests for OS in OSCC/RP3-416J7.4.jpg]

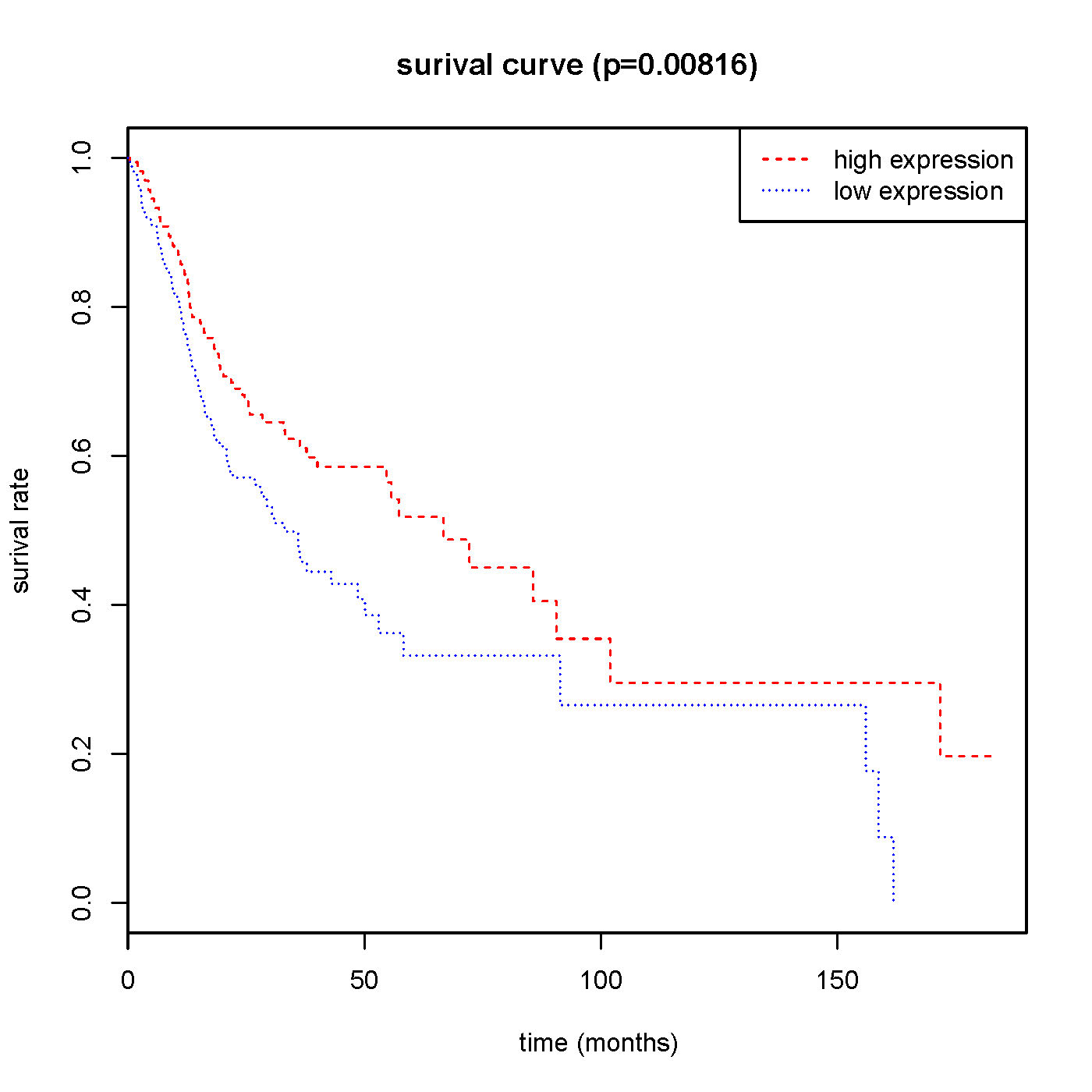

Supplement: Dataset S2 — Kaplan–Meier survival analysis with the log-rank was used to identify relationships between the above 2493 lncRNA signatures and OSCC patient survival. Then, we determined the levels of 151 lncRNA signatures that were significantly related to OS. [file peerj-06-5307-s006.zip › The result of Kaplan–Meier survival analyses and log-rank tests for OS in OSCC/RP3-429O6.1.jpg]

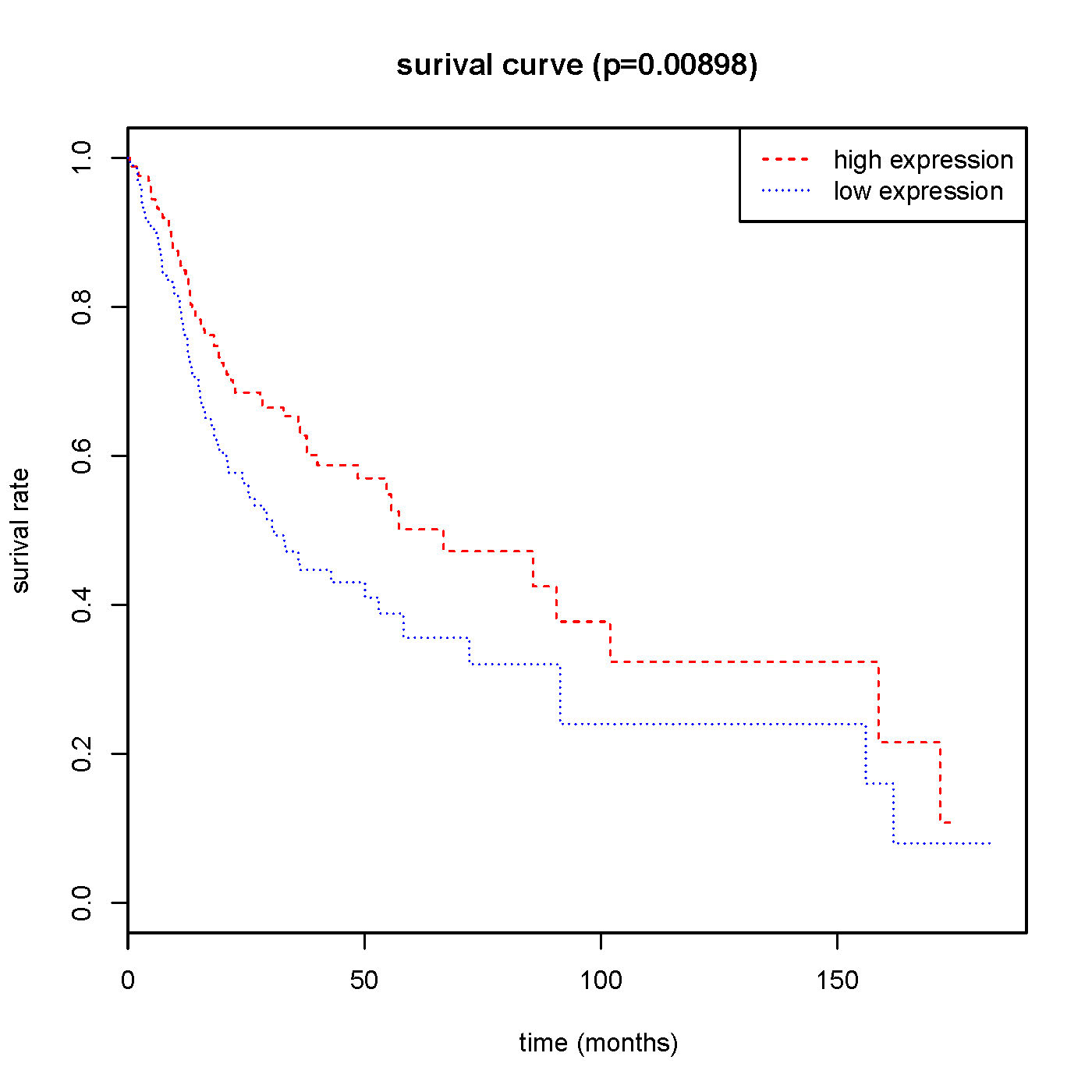

Supplement: Dataset S2 — Kaplan–Meier survival analysis with the log-rank was used to identify relationships between the above 2493 lncRNA signatures and OSCC patient survival. Then, we determined the levels of 151 lncRNA signatures that were significantly related to OS. [file peerj-06-5307-s006.zip › The result of Kaplan–Meier survival analyses and log-rank tests for OS in OSCC/RP4-547N15.3.jpg]

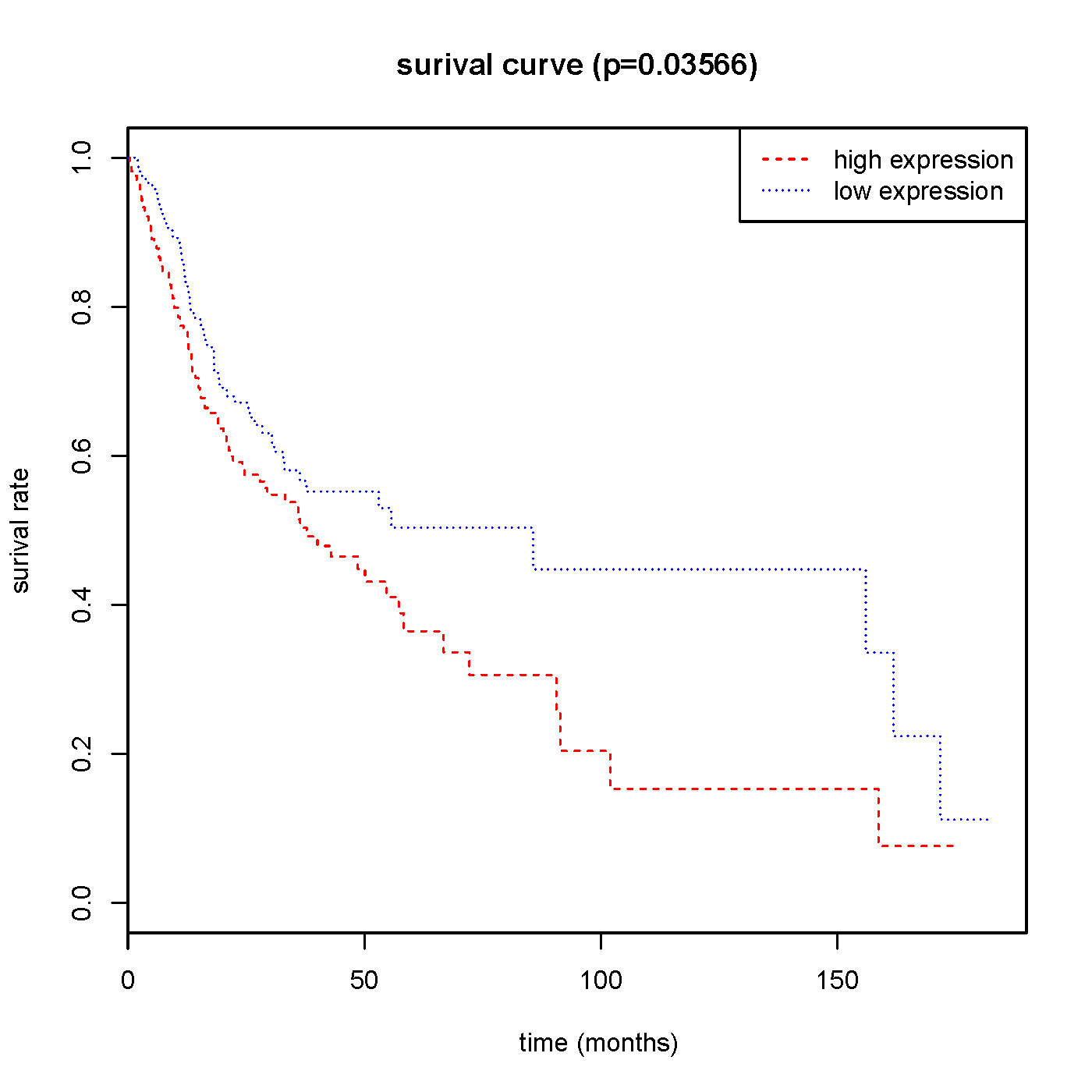

Supplement: Dataset S2 — Kaplan–Meier survival analysis with the log-rank was used to identify relationships between the above 2493 lncRNA signatures and OSCC patient survival. Then, we determined the levels of 151 lncRNA signatures that were significantly related to OS. [file peerj-06-5307-s006.zip › The result of Kaplan–Meier survival analyses and log-rank tests for OS in OSCC/RP4-594A5.1.jpg]

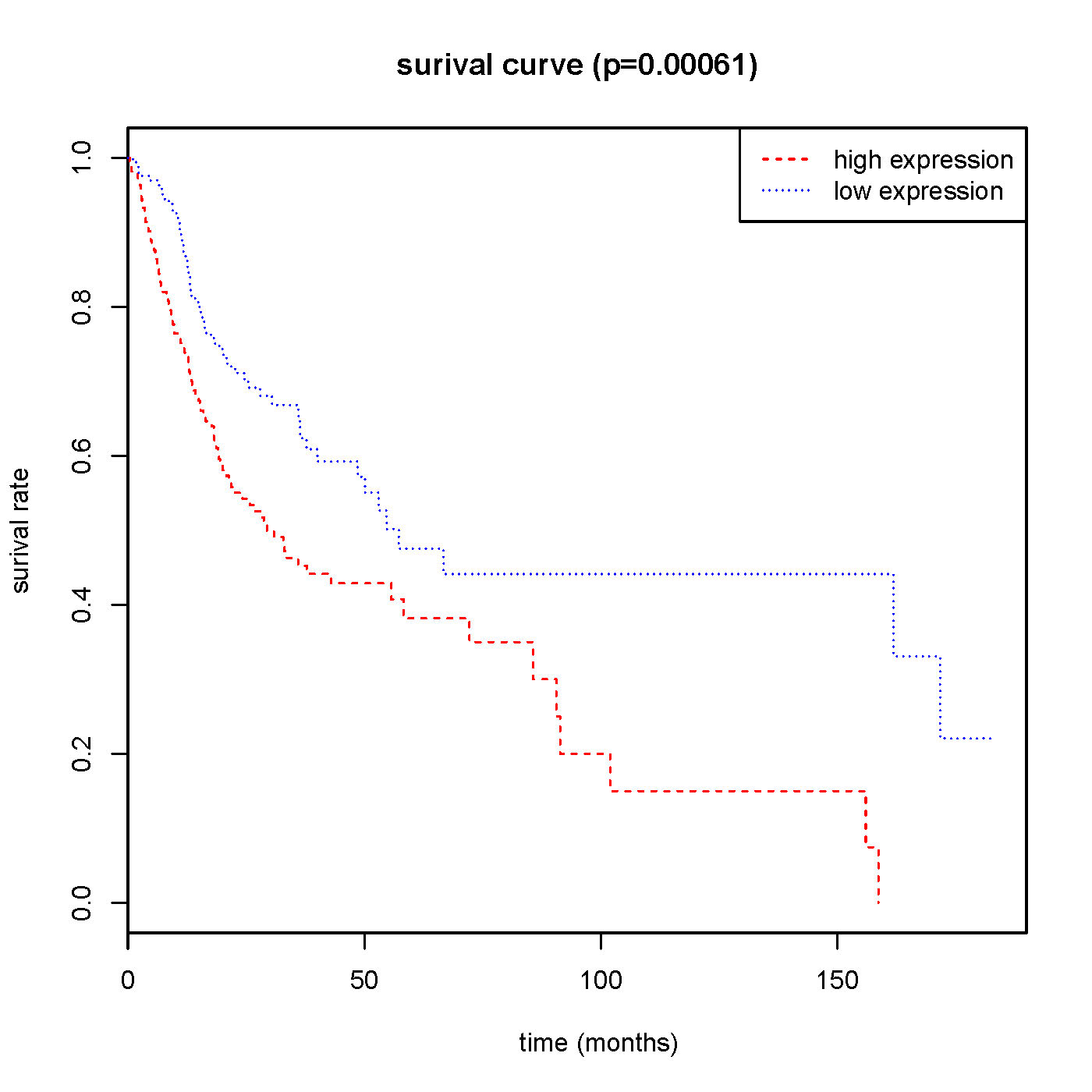

Supplement: Dataset S2 — Kaplan–Meier survival analysis with the log-rank was used to identify relationships between the above 2493 lncRNA signatures and OSCC patient survival. Then, we determined the levels of 151 lncRNA signatures that were significantly related to OS. [file peerj-06-5307-s006.zip › The result of Kaplan–Meier survival analyses and log-rank tests for OS in OSCC/RP5-1011O1.2.jpg]

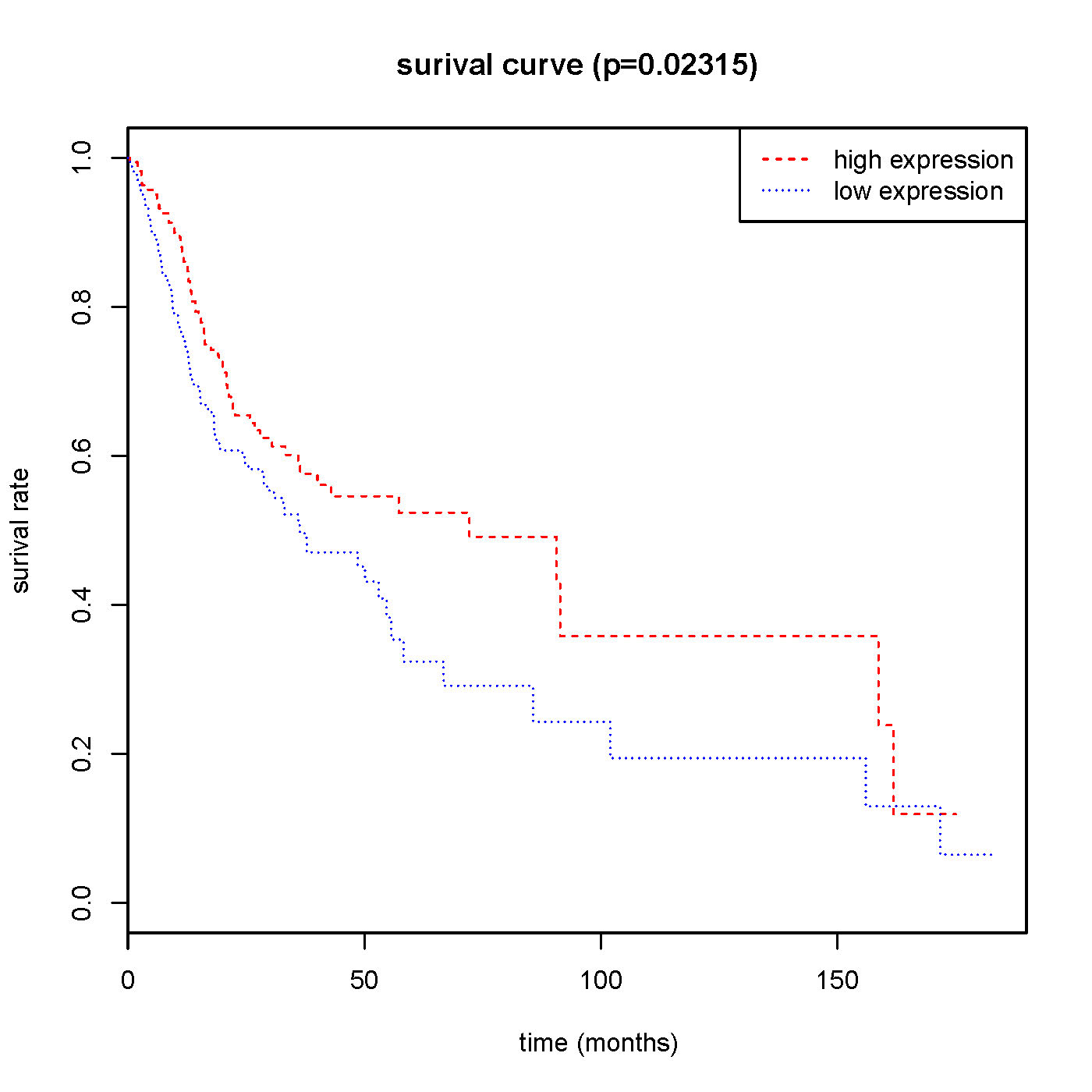

Supplement: Dataset S2 — Kaplan–Meier survival analysis with the log-rank was used to identify relationships between the above 2493 lncRNA signatures and OSCC patient survival. Then, we determined the levels of 151 lncRNA signatures that were significantly related to OS. [file peerj-06-5307-s006.zip › The result of Kaplan–Meier survival analyses and log-rank tests for OS in OSCC/RP5-1024N4.4.jpg]

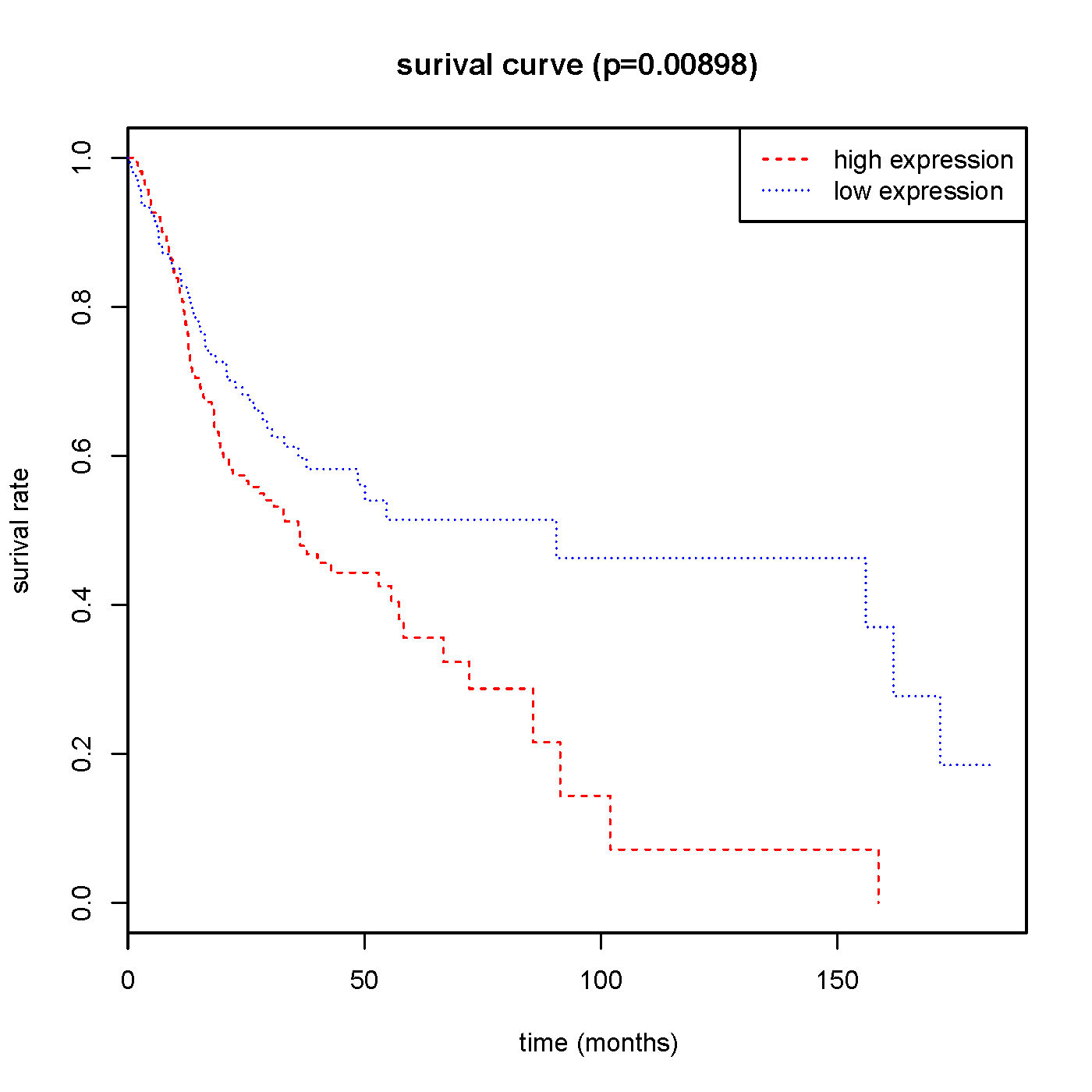

Supplement: Dataset S2 — Kaplan–Meier survival analysis with the log-rank was used to identify relationships between the above 2493 lncRNA signatures and OSCC patient survival. Then, we determined the levels of 151 lncRNA signatures that were significantly related to OS. [file peerj-06-5307-s006.zip › The result of Kaplan–Meier survival analyses and log-rank tests for OS in OSCC/RP5-991G20.1.jpg]

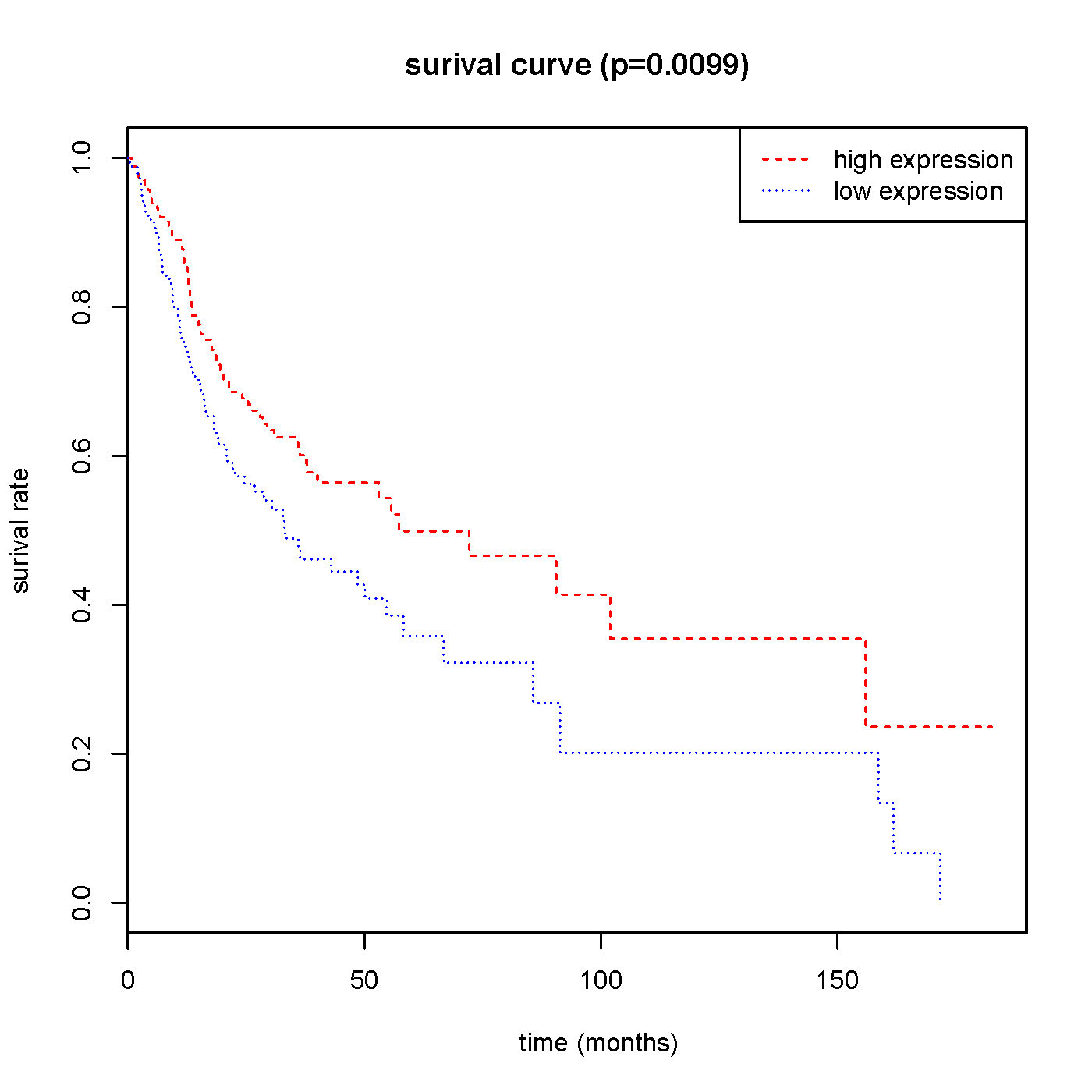

Supplement: Dataset S2 — Kaplan–Meier survival analysis with the log-rank was used to identify relationships between the above 2493 lncRNA signatures and OSCC patient survival. Then, we determined the levels of 151 lncRNA signatures that were significantly related to OS. [file peerj-06-5307-s006.zip › The result of Kaplan–Meier survival analyses and log-rank tests for OS in OSCC/STARD4-AS1.jpg]

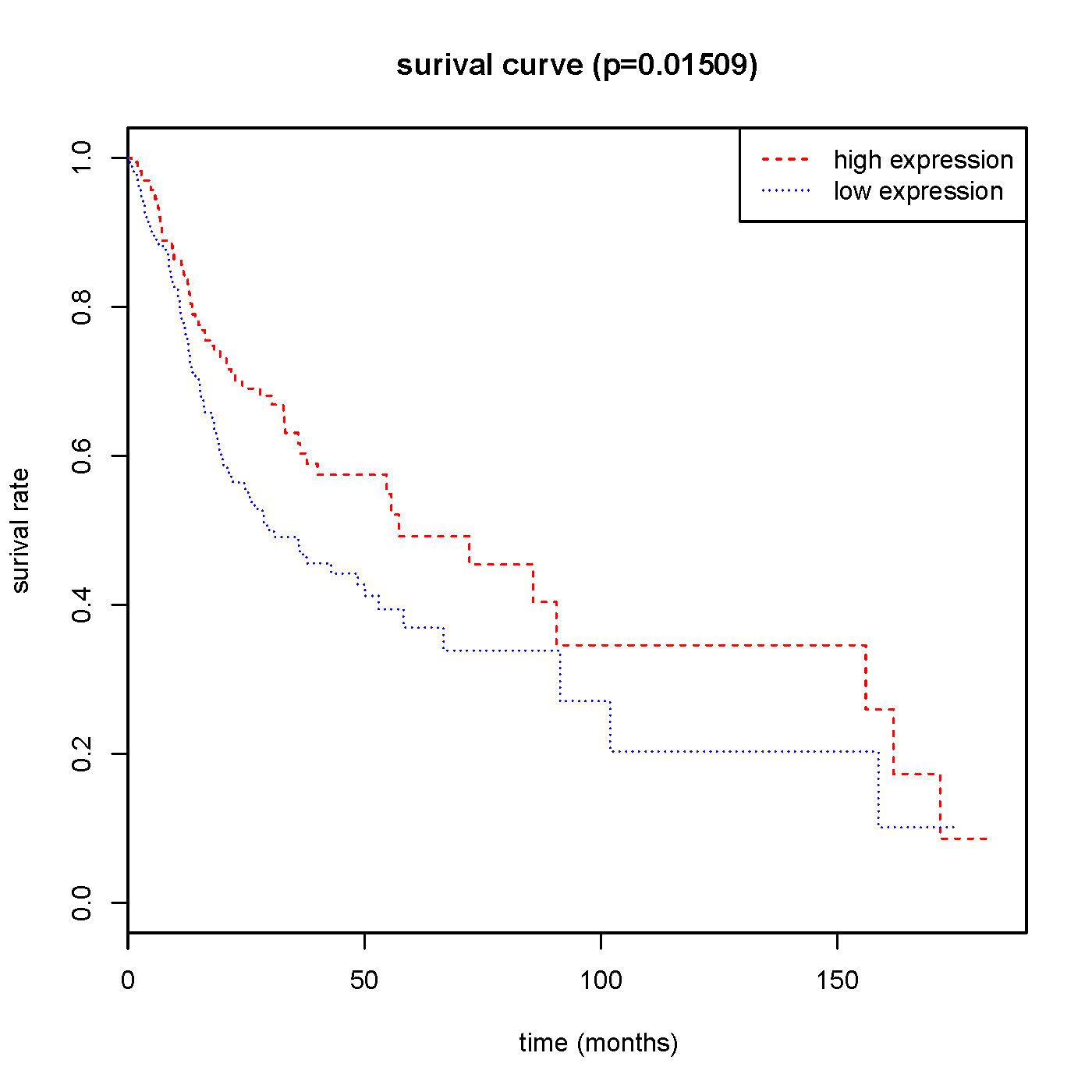

Supplement: Dataset S2 — Kaplan–Meier survival analysis with the log-rank was used to identify relationships between the above 2493 lncRNA signatures and OSCC patient survival. Then, we determined the levels of 151 lncRNA signatures that were significantly related to OS. [file peerj-06-5307-s006.zip › The result of Kaplan–Meier survival analyses and log-rank tests for OS in OSCC/TLX1NB.jpg]

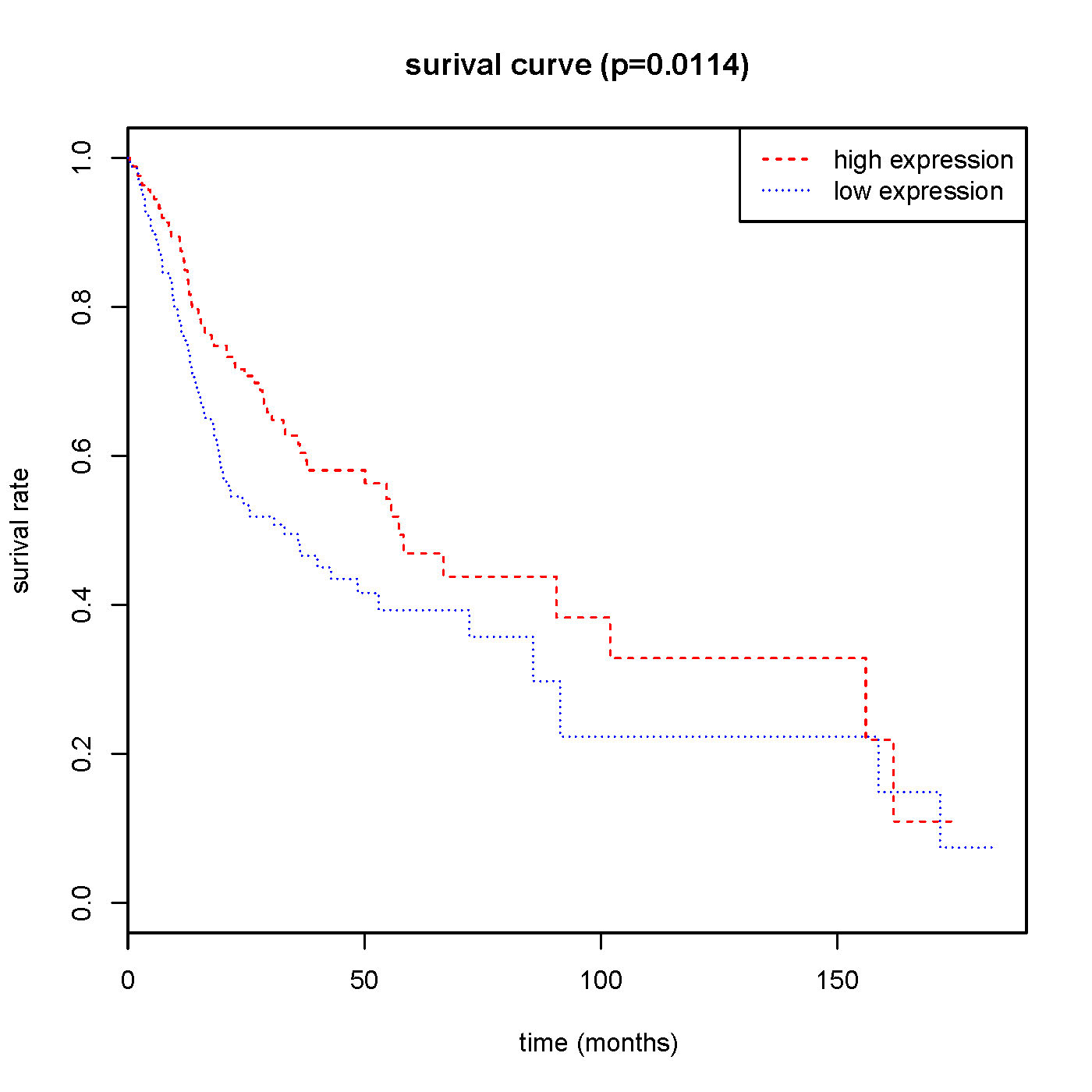

Supplement: Dataset S2 — Kaplan–Meier survival analysis with the log-rank was used to identify relationships between the above 2493 lncRNA signatures and OSCC patient survival. Then, we determined the levels of 151 lncRNA signatures that were significantly related to OS. [file peerj-06-5307-s006.zip › The result of Kaplan–Meier survival analyses and log-rank tests for OS in OSCC/TTC39A-AS1.jpg]

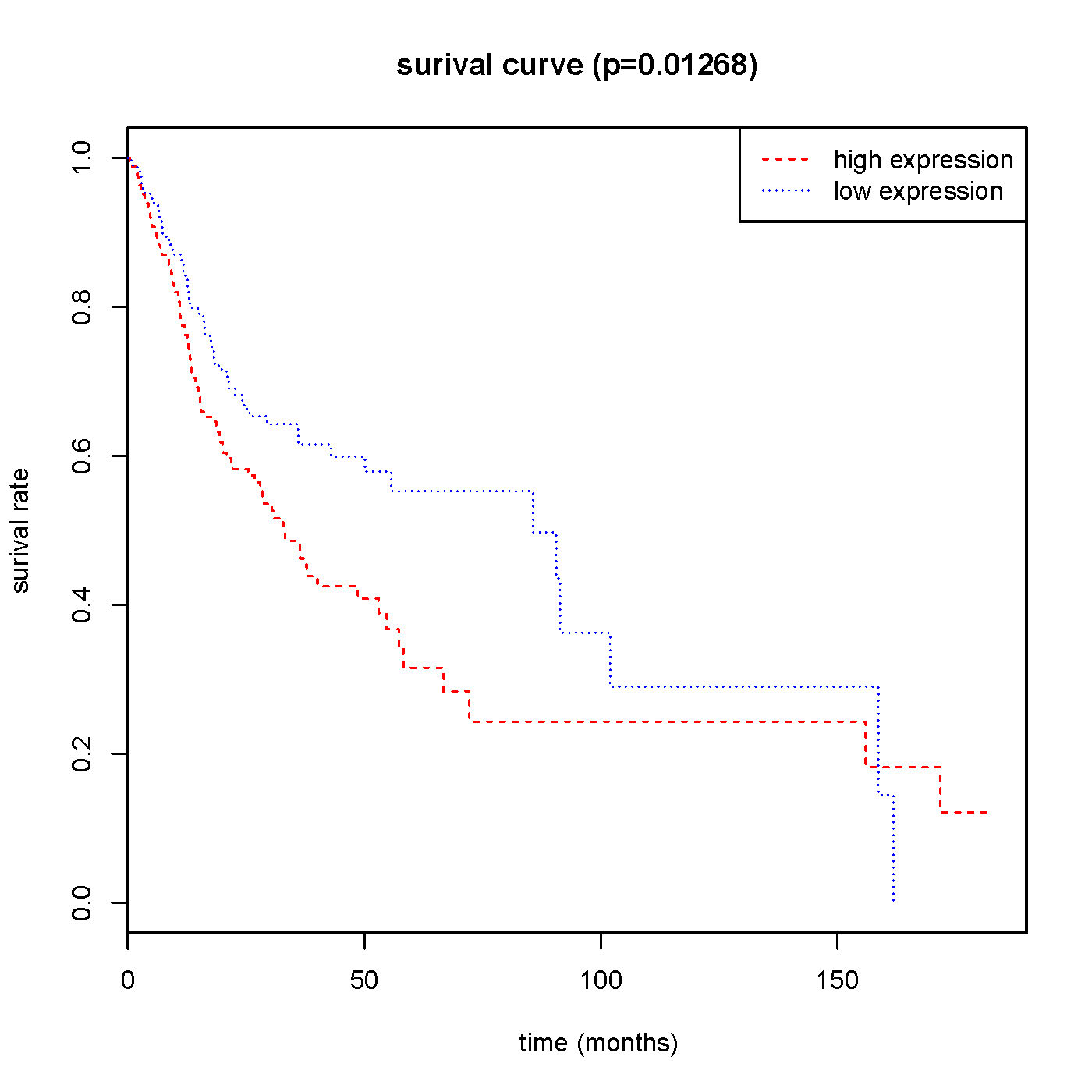

Supplement: Dataset S2 — Kaplan–Meier survival analysis with the log-rank was used to identify relationships between the above 2493 lncRNA signatures and OSCC patient survival. Then, we determined the levels of 151 lncRNA signatures that were significantly related to OS. [file peerj-06-5307-s006.zip › The result of Kaplan–Meier survival analyses and log-rank tests for OS in OSCC/WDFY3-AS2.jpg]

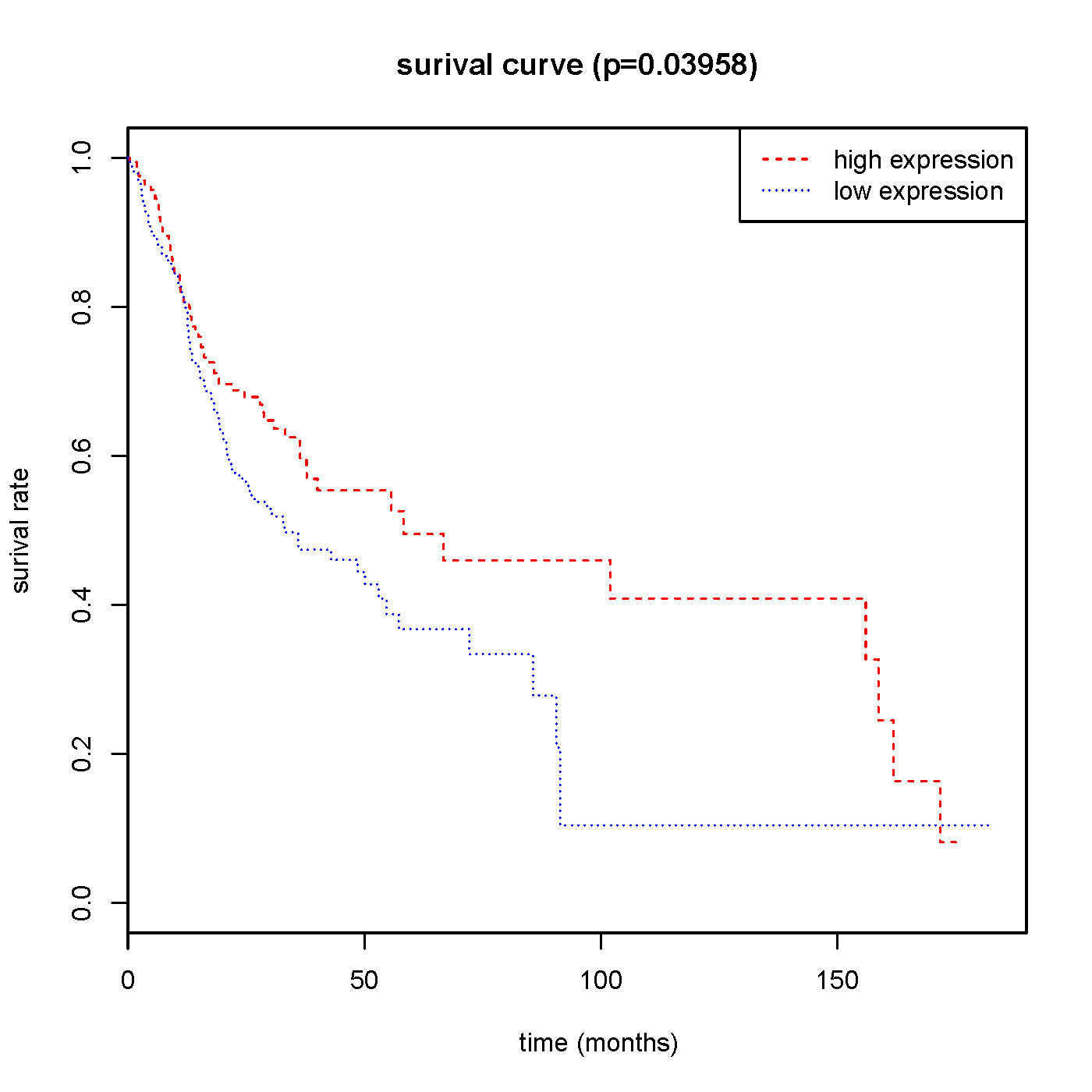

Supplement: Dataset S2 — Kaplan–Meier survival analysis with the log-rank was used to identify relationships between the above 2493 lncRNA signatures and OSCC patient survival. Then, we determined the levels of 151 lncRNA signatures that were significantly related to OS. [file peerj-06-5307-s006.zip › The result of Kaplan–Meier survival analyses and log-rank tests for OS in OSCC/ZNF503-AS1.jpg]
